# Elucidation of the acid reactivity of polyhedral orthoformates for the synthesis of carbasugar derivatives†

**DOI:** 10.1039/d5ra01049g

**Published:** 2025-06-19

**Authors:** Kazuki Usuguchi, Akira Takagi, Ippei Takashima, Kensuke Okuda

**Affiliations:** a Laboratory of Bioorganic & Natural Products Chemistry, Kobe Pharmaceutical University 4-19-1, Motoyamakita, Higashinada Kobe Hyogo 658-8558 Japan okuda@kobepharma-u.ac.jp

## Abstract

Carbasugar-containing natural products such as uvaridacol L have a variety of bioactivities, motivating chemists to develop methods for their synthesis. The conversion of *myo*-inositol is one of the most efficient methods for the synthesis of carbasugars. However, selective conversion of *myo*-inositol derivatives remains to be explored. In our synthesis of uvaridacol L derivatives, we found that the methoxy olefin derivatives of orthoester-protected *myo*-inositols, the key synthetic intermediates of our study, exhibit differing reaction selectivities depending on their geometric isomerism and substituents. Here we present new insights that contribute to the synthesis of carbasugar-type derivatives by elucidating the mechanism of the selectivity using density functional theory (DFT) calculations.

## Abstract 2

We present new insights that contribute to the synthesis of carbasugar derivatives by elucidating the acid reactivity of polyhedral orthoformates depending on their geometric isomerism and substituents using DFT calculations.

## Graphical abstract

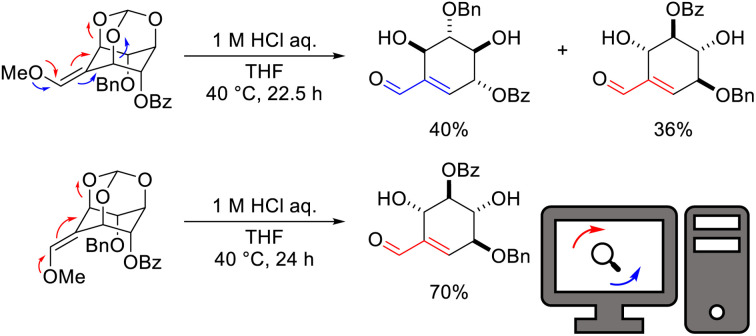

## 1 Introduction

**Fig. 1:**
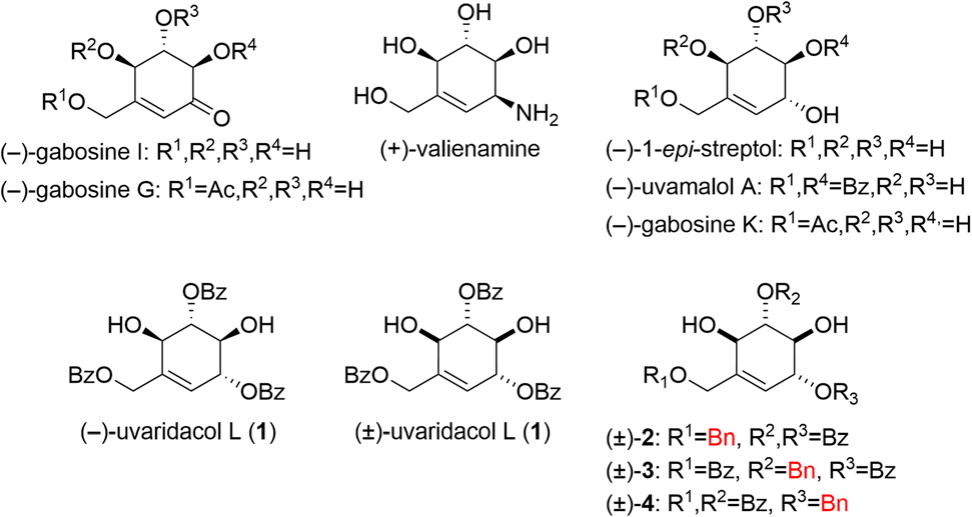
Carbasugar-type natural products and their derivatives.

Carbasugar-containing natural products^1–6^ such as gabosines, streptol, uvamalol, and valienamine have a variety of biological activities, including antimicrobial^7^ and antitumor activities^8^ (Fig. 1). Since carbasugars have roles as pseudosugars, they are at times incorporated into pharmaceuticals, the diabetes drug voglibose being a notable example. On the other hand, (−)-uvaridacol L (1), a carbasugar analogue isolated from the leaves of *Uvaria dac*, is known to exhibit selective cytotoxicity (50% preferential cytotoxicity, PC_50_ = 20.1 μM) toward the PANC-1 human pancreatic cancer cell line in nutrient deprived medium (NDM).^9^ Some types of cancer cells, including PANC-1, adapt to nutrient starvation – a characteristic of solid tumors – and resistance to existing conventional anticancer drugs such as gemcitabine.^10^ Therefore, uvaridacol L is attracting attention as a seed compound for novel drug discovery targeting the cancer-specific low nutrition environment.

Carbasugars have characteristic structures which consist of highly oxygenated cyclohex-1-en-1-ylmethanol and successive chiral centers.^11^ Also, hydroxy groups of carbasugar-type natural products are often functionalized by acyl groups such as benzoyl and/or acetyl moieties. Several synthetic methods are currently employed to synthesize carbasugars with these characteristics. One is the construction of new skeletons of carbasugars from sugars such as glucose.^11–15^ This strategy has some advantages such as the preexisting chiral hydroxy groups that are found in the starting material. However, the hemiacetal oxygen ether functionality must be replaced with a methylene group by a carbocyclization such as the Horner–Wadsworth–Emmons reaction with a suitable protection–deprotection strategy, which requires a multistep synthesis. The other is the semisynthetic approach by using shikimic acid that possesses the cyclohexene moiety and quinic acid (the hydrated form of shikimic acid) as starting materials.^12,13^ These starting materials derived from natural products already have similar chirality corresponding to the target carbasugars with a cyclohexene core, but they require the introduction of a hydroxy group at the 6-position with a possible need for stereo inversion of the 3,4,5-hydroxy groups. One of the other approaches uses *myo*-inositol.^14–16^ Although requiring *exo*-homologation at the 1-potision, the construction of carbasugars from *myo*-inositol provides many advantages particularly in regard to the stereochemistry at the 3,4,5,6-position hydroxy groups (all-*trans*) which perfectly match that of the target compound. Leveraging this advantage, we previously reported the first total synthesis of (±)-uvaridacol L (1) from *myo*-inositol in seven steps.^17^ We also showed that the benzoyl moiety on the primary hydroxy group is essential for the antiausterity property of (±)-1,^17^ and the potency of racemic (±)-1 is almost the same as that of natural (−)-uvaridacol L (1).^10^ This implies that the racemic compound can be used for structure activity relationship (SAR) studies.

For further SAR investigations, we were interested in how the ester carbonyl moiety affects the antiausterity properties. To explore the pharmacophore, deoxygenated derivatives ((±)-2–4, *i.e.* the benzoyloxy moieties of (±)-uvaridacol L substituted with benzyloxy groups) are necessary (Fig. 1). In this study, we aimed to establish a new synthetic route for and evaluate the antiausterity activities of uvaridacol L derivatives ((±)-2–4). Furthermore, we found that the direction of double bond migration in the ring-opening reaction of orthoesters for methoxy olefins changed depending on the substituent properties and we investigated the mechanism of this preference using a theoretical study.

## 2 Results and discussion

**Scheme 1:**
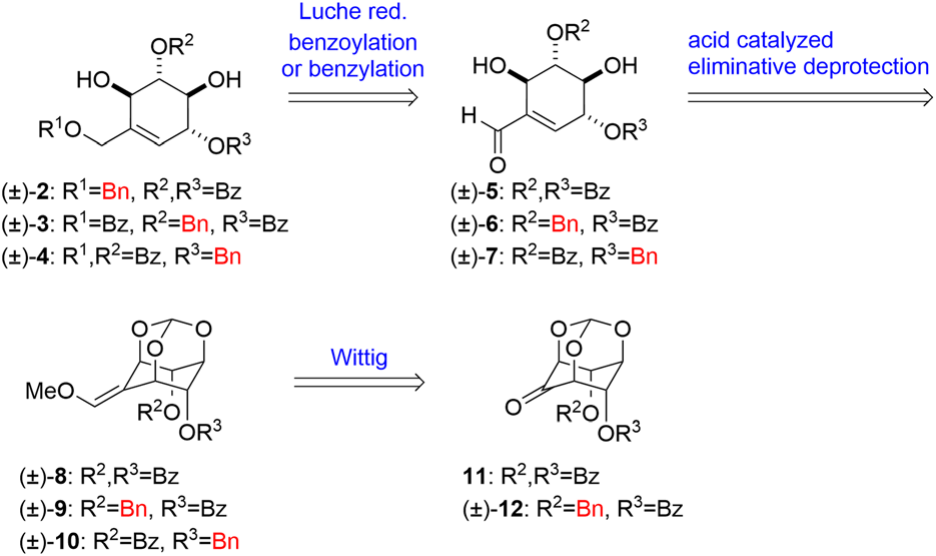
Retrosynthetic analysis of deoxygenated derivatives of (±)-uvaridacol L (2–4).

**Scheme 2:**
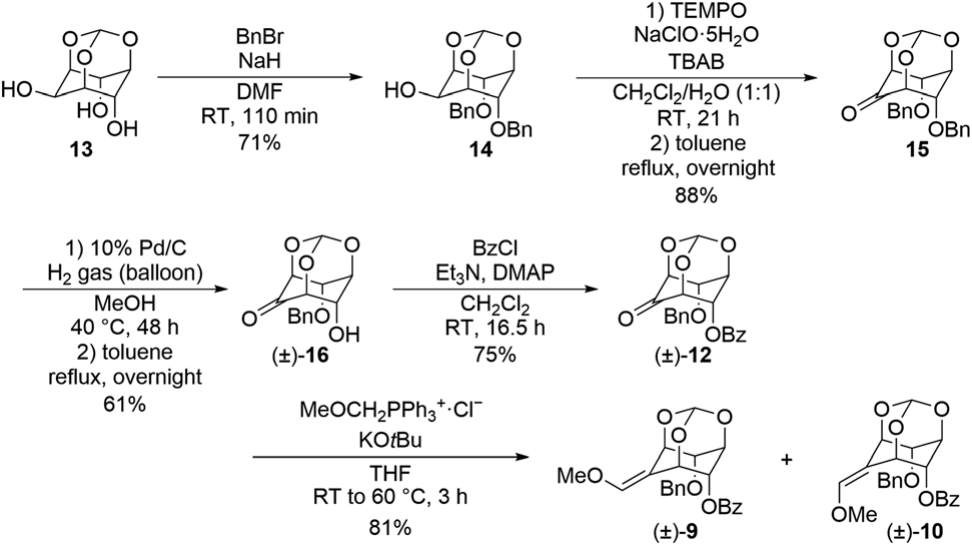
Synthesis of (±)-methoxy olefins (9 and 10).

To begin the study, we established an alternative synthetic route for the synthesis of (±)-2–4. Retrosynthetic analysis is as follows (Scheme 1). (±)-2–4 are accessible by Luche reduction of conjugated aldehydes (±)-5–7 followed by benzoylation or benzylation of the primary hydroxy group. The cyclohexene skeleton of (±)-5–7 can be constructed by Brønsted acid catalyzed eliminative deprotection of orthoesters (±)-8, (±)-9 and (±)-10. The methoxy olefin moieties of (±)-8, (±)-9 and (±)-10 can be obtained by Wittig reaction from the key intermediates 11 and (±)-12, which are accessible from *myo*-inositol orthoformate (13) in several steps. *E*/*Z*-selectivity of this Wittig reaction governs the yield of asymmetric (±)-9 and (±)-10, which also affects (±)-6 and (±)-7. We have previously shown the preparation of (±)-5 through (±)-8 and 11.^17^ To prepare the other key intermediates (±)-6 and (±)-7, first, using *myo*-inositol orthoformate 13 as the starting material, the two hydroxy groups at the axial positions of 13 were protected with benzyl groups to afford the dibenzyl product 14 in 71% yield. The remaining hydroxy group of 14 was oxidized by 2,2,6,6-tetramethylpiperidine 1-oxyl (TEMPO) to give ketone 15 in 88% yield. One of the benzyl groups of 15 was removed by Pd/C-catalyzed reduction to afford the monobenzyl product (±)-16 in 61% yield, and the resulting hydroxy group was benzoylated to afford the benzoylate (±)-12 in 75% yield. Then Wittig reaction of (±)-12 gave methoxy *E*-olefin (±)-9 and methoxy *Z*-olefin (±)-10 (*E* : *Z* = 7 : 10) in 81% total yield (Scheme 2).

**Scheme 3:**
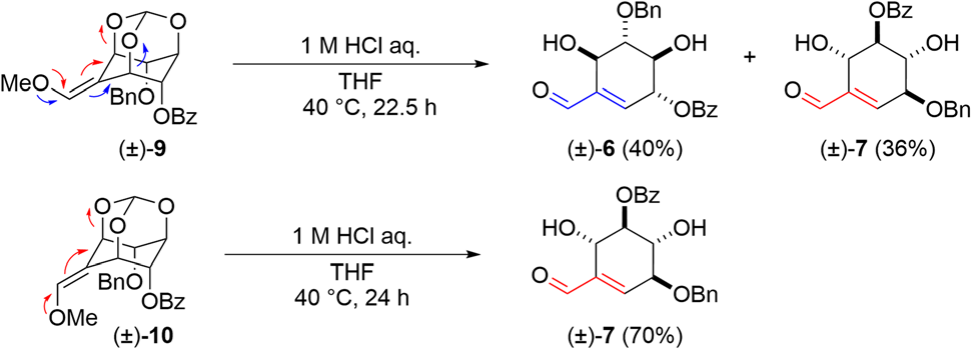
The ring-opening reaction of (±)-methoxy olefins (9 and 10).

The obtained (±)-9 and (±)-10 by further purification were treated with acid for the construction of the cyclohexene ring followed by cleavage of the orthoester, respectively (Scheme 3). Upon treating (±)-9 and (±)-10 with 1 : 1 1 M aq. HCl and THF, (±)-9 provided (±)-6 with the double bond formed on the benzoyloxy group side and (±)-7 with the double bond formed on the benzyloxy group side in 40% and 36% yields, respectively, while (±)-10 provided (±)-7 in 70% yield as a single product. These differences in selectivity are considered to be due to a match-mismatch between the two orientations of the substituents on the hydroxy group and the electronic effect of the methoxy group.

**Scheme 4:**
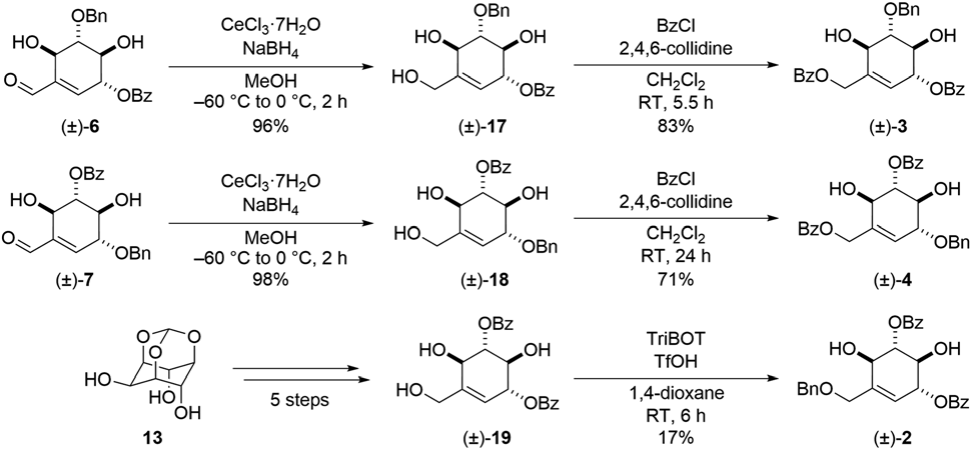
Synthesis of deoxygenated derivatives of (±)-uvaridacol L (2–4).

The obtained (±)-6 and (±)-7 were converted to the respective derivatives by the same synthetic process (Scheme 4). Luche reduction of (±)-6 and (±)-7 gave allylic alcohols (±)-17 and (±)-18 in 96% and 98% yields respectively. The deoxygenated derivatives of uvaridacol L ((±)-3 and (±)-4) were synthesized by benzoylation of the resulting primary alcohols with 2,4,6-collidine in 83% and 71% yields, respectively. Finally, allylic alcohol (±)-19 ^17^ was converted to benzyl ether (±)-2 in 17% yield by acidic benzylation with 2,4,6-tris(benzyloxy)-1,3,5-triazine (TriBOT).^18^

**Scheme 5:**
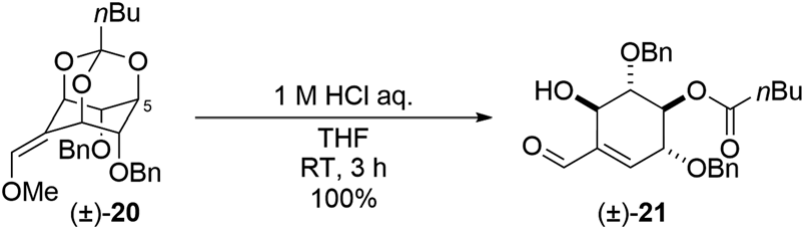
Related substrate (±)-20 for (±)-9 and (±)-10 and its product under acidic condition ((±)-21).

**Scheme 6:**
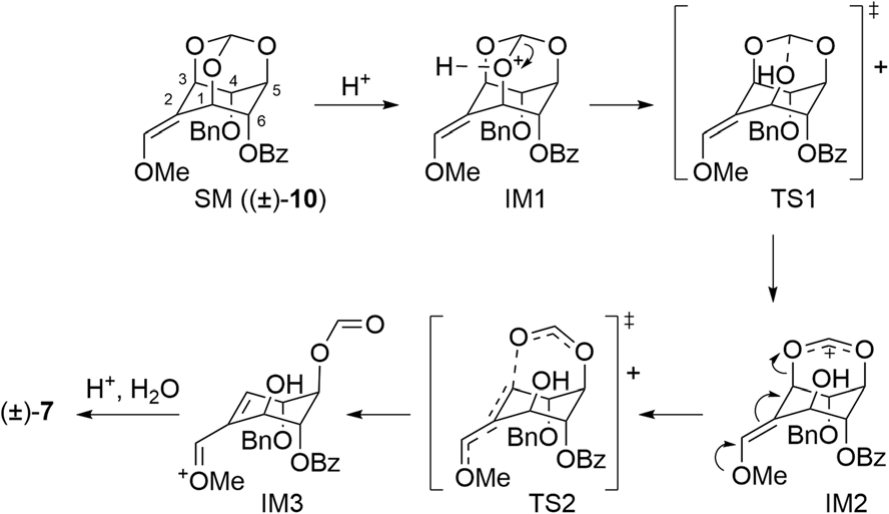
Mechanism of ring-opening reaction for (±)-10.

During these transformations, we were interested in understanding the mechanism that determines the regioselectivity of the construction of the cyclohexene skeleton ((±)-6 and (±)-7) from methoxy olefin orthoformates ((±)-9 and (±)-10). The direction of the cleavage is influenced by the type of substituent and the geometry of the methoxy olefin (Scheme 3). Controlling the regioselectivity of this cleavage would lead to the development of the selective synthesis of carbasugars using *myo*-inositol and we aimed to investigate the mechanism of this selectivity by computational chemistry. In a previous study, Sureshan and co-workers reported experiments using orthovalerate ((±)-20, Scheme 5), which gave a stable valerate ester after ring-opening rather than the acid-labile formate ester, as a substrate in the cleavage reaction of orthoesters to confirm the selectivity of the position activated by protonation.^14^ They confirmed that the ring-opening of the orthovalerate quantitatively provided a 5-*O*-acylated product ((±)-21). One can expect that the reaction mechanism is as follows: first, protonation at the orthoester O atom (intermediate (IM)1) of (±)-10 (starting material (SM)) leads to C–O bond cleavage to give IM2 followed by hydrolysis to yield (±)-7 (Scheme S1†). Also, one can assume that the protonation of each orthoester oxygen atom of (±)-9 and (±)-10 is reversible; however, putative density functional theory (DFT) calculations showed that every protonation triggered spontaneous generation of H-IM1 with more than 120 kcal mol^−1^ of stabilization compared to the SM, which suggests that the protonation is irreversible (Scheme S2†). Therefore, we considered this mechanism implausible. To continue our investigation of the mechanism, we then assumed that the reaction proceeds through a two-step transition state (Scheme 6).

First, the orthoester oxygen is activated by coordination to a proton, and the neighboring C–O bond is cleaved (transition state (TS)1). Next, electron donation from the lone pair of the methoxy group leads to olefin transfer, cleaving the C–O bond to form a formyl group (TS2). Based on the assumption that the reaction passes through these transition states, we investigated the mechanism of cleavage and the factors involved in the selectivity by using DFT calculations. All DFT optimization and transition state calculations were performed at the B3LYP/6-31G** level and in water using the SMD (solvation model based on density) model.^19^ Single-point energy calculations were performed at the B3LYP/def2-TZVP level and in water using the SMD model.

**Fig. 2:**
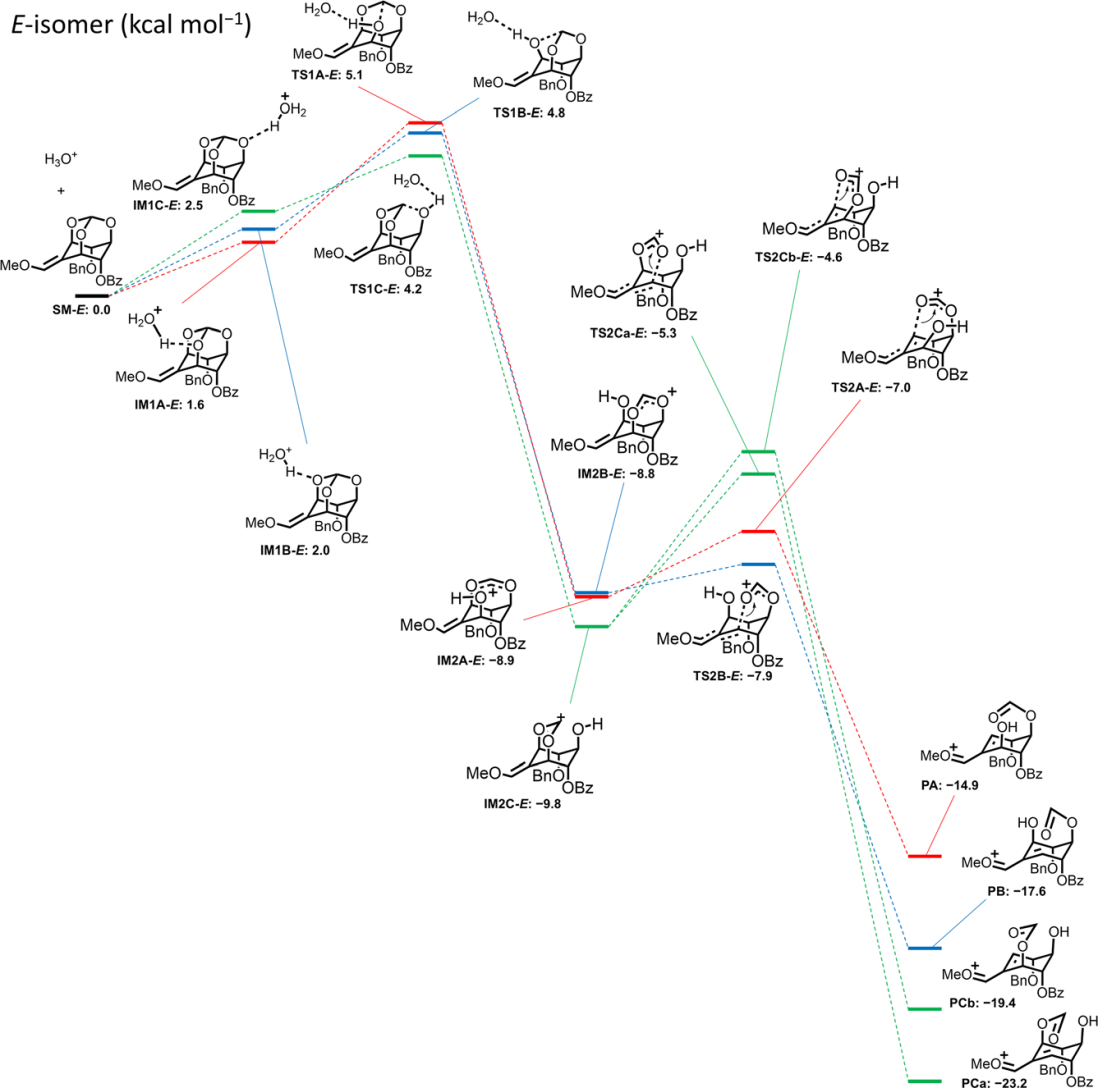
Energy profile for ring-opening reaction of *E*-isomer (±)-9 calculated by DFT (B3LYP/def2-TZVP//B3LYP/6-31G** [SMD = water]).

To analyze more detailed modelling of the protonation step, we calculated the structural stabilization using a model in which the hydronium ion was added. As a result, the structures of *E* isomer (±)-9 with 1-*O*, 3-*O* and 5-*O* coordinating to the hydrogen atoms of the oxonium ion were destabilized by 1.6 kcal mol^−1^ (1-*O*: IM1A-*E*), 2.0 kcal mol^−1^ (3-*O*: IM1B-*E*), and 2.5 kcal mol^−1^ (5-*O*: IM1C-*E*), respectively (Fig. 2). Then, the activation energies of the transition state (TS1) in the C–O bond cleavages of the orthoester were calculated from each hydrated oxonium ion adduct as the ground state. The activation energies of each transition state, TS1A-*E*, TS1B-*E*, and TS1C-*E*, were 3.5 kcal mol^−1^, 2.8 kcal mol^−1^, and 1.7 kcal mol^−1^, respectively. From these energy differences, it can be assumed that the proton transfer between the orthoester oxygen of the hydrated oxonium ion is reversible and that the route of C–O bond cleavage *via*TS1C-*E*, which requires the lowest activation energy, is the major pathway to producing the ring-opened products (±)-6 and (±)-7, followed by the route of C–O bond cleavage *via*TS1B-*E*. The double bond transfer and resulting orthoester cleavage at the 1-*O* and 3-*O* positions compete during the conversion through TS2C-*E* from IM2C-*E* to the product (PC). The activation energies (ΔΔ*G*^‡^) of the transition states providing the respective products were 4.5 kcal mol^−1^ for ring opening in the 1-*O* direction (TS2Ca-*E*) and 5.2 kcal mol^−1^ in the 3-*O* direction (TS2Cb-*E*). The difference between the two activation energies is 0.7 kcal mol^−1^ which suggests that (±)-6, resulting from PCa, is preferred slightly as the product compared to (±)-7 which is derived from PCb by this pathway. On the other hand, the route *via*IM2B-*E*, which requires the second lowest activation energy after that of IM2C-*E*, produces only PB to lead to (±)-6. These results suggest that the reaction with (±)-9 as the substrate has poor selectivity. This result corresponds to the experimental results in which the two products ((±)-6 and (±)-7) were obtained as a mixture.

**Fig. 3:**
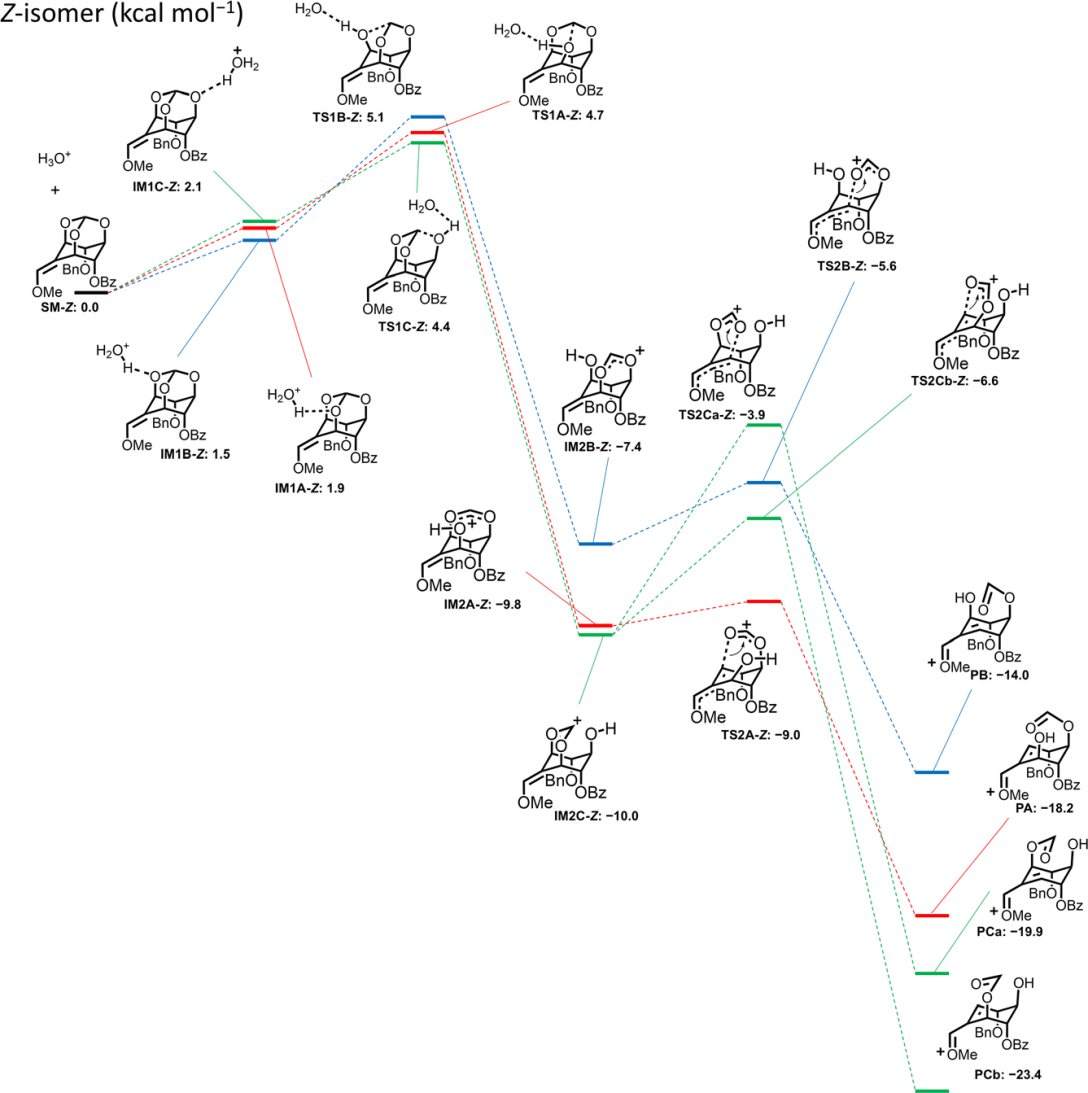
Energy profile for ring-opening reaction of *Z*-isomer (±)-10 calculated by DFT (B3LYP/def2-TZVP//B3LYP/6-31G** [SMD = water]).

The same calculation was applied for the *Z* isomer (±)-10, where the protonated forms (IM1A-*Z*, IM1B-*Z*, and IM1C-*Z*) were in equilibrium, and the two lower TS1 activation energies were observed when 5-*O* (TS1C-*Z*: ΔΔ*G*^‡^ = 2.3 kcal mol^−1^) or 1-*O* was protonated (TS1A-*Z*: ΔΔ*G*^‡^ = 2.8 kcal mol^−1^) (Fig. 3). The activation energies of each transition state giving products from IM2C-*Z* were estimated to be 6.1 kcal mol^−1^ for the ring-opening reaction in the 1-*O* direction (TS2Ca-*Z*) and 3.4 kcal mol^−1^ for the ring-opening reaction in the 3-*O* direction (TS2Cb-*Z*). The difference between the two activation energies was 2.7 kcal mol^−1^, suggesting that the reaction *via*TS2Cb-*Z* gives PCb to lead to (±)-7 as the more favorable product from (±)-10. Additionally, the route *via*TS1A-*Z* produces only PA to lead to (±)-7. These considerations of the mechanism correspond well with the experimental result that the reaction of (±)-10 gave exclusively (±)-7 in 70% yield.

From another perspective of the reactions converting (±)-9 and (±)-10 to (±)-6 and (±)-7, the sites of double bond formation tended to be on the side trans to the methoxy group with less preference for the side adjacent to the benzoyl moiety. To elucidate the mechanism of regioselectivity further, the electrostatic interactions which are important for the reactions of the dibenzoylated compound (±)-8 and dibenzylated compound (±)-22, in addition to (±)-9 and (±)-10, were calculated using second-order perturbation theory analysis of the Fock matrices in the natural bond orbital (NBO) analyses (Table 1).

**Table 1:** Second-order perturbation theory analysis of the Fock matrices in the NBO analyses (B3LYP/def2-TZVP//B3LYP/6-31G** [SMD = water])

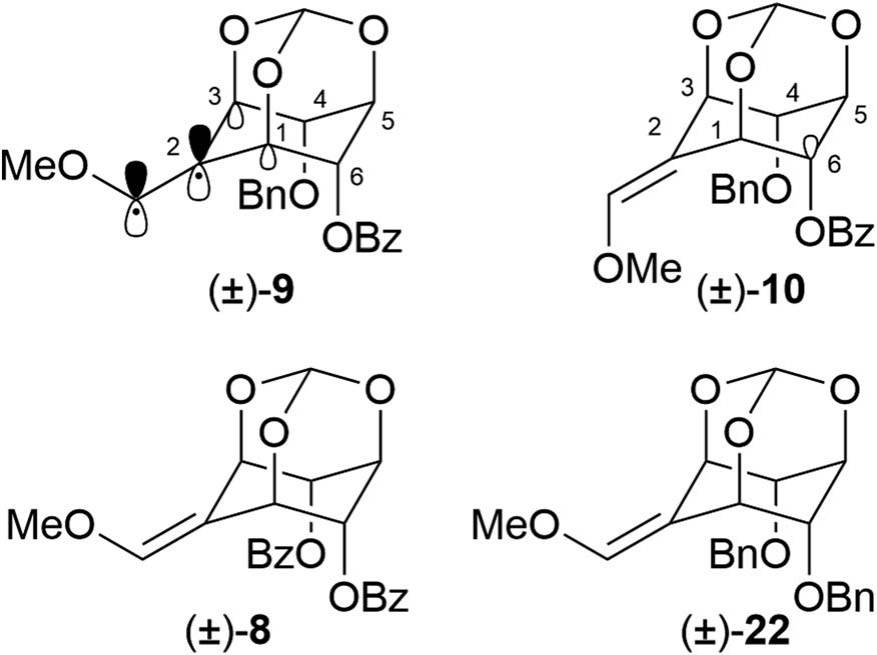			
NBO	Donor (BO)	Acceptor (ABO)	*E* (kcal mol^−1^)
*E*-Isomer (±)-9	C1–O	C6–O	2.1
	C3–O	C4–O	1.9
	C=C (*p*)	C1–O (*trans*)	5.5
	C=C (*p*)	C3–O	4.8
*Z*-Isomer (±)-10	C1–O	C6–O	2.1
	C3–O	C4–O	1.8
	C=C (*p*)	C1–O	5.1
	C=C (*p*)	C3–O (*trans*)	5.6
BzBz (±)-8	C1–O	C6–O	2.1
	C3–O	C4–O	1.9
	C=C (*p*)	C1–O (*trans*)	5.5
	C=C (*p*)	C3–O	4.8
BnBn (±)-22	C1–O	C6–O	2.0
	C3–O	C4–O	1.6
	C=C (*p*)	C1–O (*trans*)	5.4
	C=C (*p*)	C3–O	4.7

The greater the electron donation from the bonding orbital (BO) of the orthoester C–O bond to the antibonding orbital (ABO) of the neighbouring C–O bond, the greater the dissociation energy, making it more difficult to cleave the bond. Comparing the donation of electrons from the C1–O or C3–O BO to the C6–O or C4–O ABO in (±)-9 and (±)-10, the stabilization energy from the donation of energy to the ABO on the benzoyloxy group side is higher than that to the ABO on the benzyloxy group side. This result may be attributed to the increased electron acceptability of the antibonding orbital due to the electron-withdrawing property of the benzoyl group, which generates unfavorable effects on the C–O bond cleavage at the neighboring site. In addition, the donation of electrons from the π–electrons of the methoxy olefin double bond to the ABOs of the C1–O and C3–O bond may contribute to the advantageous delocalization of electrons.

To evaluate the methoxy olefin orientation effect simply, stabilization by electron donation from the π electrons of (±)-8 and (±)-22, which are protected by the same protecting group, to the ABO of the C1–O (*trans*) bond and C3–O (*cis*) bond were calculated, respectively. Protection with both benzoyl ((±)-8) or benzyl ((±)-22) groups showed strong preference for the π–electron donating orientation toward the *trans* position of the methoxy group. From these electrostatic effects, the cleavage of orthoester (±)-9 resulted in a mixture of (±)-6 and (±)-7 due to the competing substituent effects of the orientation of enol ether and ether/ester protection, whereas the reaction of (±)-10 resulted in (±)-7 as a single product due to collaborative substituent effects. We expect that these insights into the selectivity of the orthoester cleavage directed by the characteristics of the neighboring substituents will be useful for the molecular design and syntheses of other carbasugar derivatives from *myo*-inositol.

Finally, synthesized deoxygenated uvaridacol L derivatives (±)-2–4 were evaluated for their preferential cytotoxicity toward PANC-1 cells under nutrient deprived conditions by WST-8 assay. As (±)-2–4 are racemic mixtures, their stereochemistry may affect their potency. Therefore, we first evaluated the cytotoxicity of the (±)-uvaridacol L (1) racemic mixture, the natural enantiomer (−)-uvaridacol L (1), and the unnatural enantiomer (+)-uvaridacol L (1) in NDM compared to standard low glucose Dulbecco's modified Eagle medium (DMEM) supplemented with 10% fetal bovine serum (FBS) (+FBS) as well as FBS-free DMEM (−FBS) and FBS, sodium pyruvate, and glucose-free DMEM (−FBS, −SP & −Glc) over 24 h (Fig. S1,† and Table 2).

**Table 2:** Cytotoxicity of 1–4 toward PANC-1 cells. Each figure indicates concentration ± standard deviation (μM) at which 50% of the cells' metabolism was suppressed in each medium determined by WST-8 assay. The results are expressed as the mean ± standard deviation (*n* = 3–4)

Compound	+FBS	−FBS	−FBS, −SP, & −Glc	NDM
(±)-1	98.5 ± 10.6	51.1 ± 14.8	32.3 ± 1.2	33.5 ± 1.8
(−)-1^a^	81.8 ± 10.0	30.4 ± 2.0	24.6 ± 5.0	14.2 ± 1.1
(+)-1	102.9 ± 22.9	31.1 ± 1.9	26.7 ± 3.8	20.1 ± 8.3
(±)-2	108.8 ± 26.4	40.8 ± 17.9	27.7 ± 8.3	22.3 ± 11.7
(±)-3	138.5 ± 29.3	60.4 ± 34.4	43.9 ± 25.2	33.7 ± 8.4
(±)-4	141.4 ± 12.8	48.8 ± 20.7	35.6 ± 10.9	44.8 ± 25.8

a We have previously reported the cytotoxicity of (−)-1.^10^

We did not observe much difference between the enantiomers and the racemic mixture in each medium and they all showed preferential cytotoxicity in NDM. These data imply that the stereochemistries of 2–4, which are quite similar to 1, also have little effect on their potency. Then the evaluation of racemic (±)-2–4 was conducted to observe their cytotoxicities. As (±)-2–4 are derivatives of (±)-1, where one of the three benzoyl esters was converted to a benzyl ether, these results implied that the contribution of the ester carbonyl oxygen to their potency is small, contrary to the mono debenzoylated derivative (±)-19 which was found to be significantly less potent than (±)-1.^17^ These datapoints are useful for the design of more fine-tuned derivatives of uvaridacol L (1) as antiausterity compounds.

To explore the general preferential cytotoxicity of 1–4 in NDM toward cancer cells that have resistance to conventional anticancer drugs, we also evaluated the activity of (+)-1, (−)-1 and (±)-1–4 toward HT-29 colorectal cancer cells which, like PANC-1 cells, are also viable under nutrient starved conditions and resistant to gemcitabine.^10^ The WST-8 assay was performed in RPMI-1640 supplemented with 10% FBS (+FBS), FBS free RPMI-1640 medium (−FBS), FBS, sodium pyruvate, and glucose free RPMI-1640 medium (−FBS, −SP, and −Glc), and NDM. The trend with HT-29 cells (Fig. S2,† and Table 3) matched that of PANC-1 cells (Fig. S1,† and Table 2), giving evidence that 1–4 may act more generally on cancer cells under nutrient deprived conditions than currently known.

**Table 3:** Cytotoxicity of 1–4 toward HT-29 cells. Each figure indicates concentration ± standard deviation (μM) at which 50% of the cells' metabolism was suppressed in each medium determined by WST-8 assay. The results are expressed as the mean ± standard deviation (*n* = 3–6)

Compound	+FBS	−FBS	−FBS, −SP, & −Glc	NDM
(±)-1	67.9 ± 7.0	25.3 ± 11.5	28.7 ± 11.0	17.7 ± 5.6
(−)-1^a^	66.0 ± 12.0	23.3 ± 9.4	19.2 ± 6.3	13.3 ± 3.7
(+)-1	70.2 ± 11.3	43.0 ± 32.1	42.1 ± 34.7	17.4 ± 6.8
(±)-2	49.5 ± 23.7	17.1 ± 2.4	15.6 ± 2.2	13.0 ± 1.7
(±)-3	138.6 ± 50.5	89.2 ± 35.5	28.0 ± 6.3	19.8 ± 8.6
(±)-4	122.3 ± 23.3	46.4 ± 17.4	30.9 ± 3.4	28.5 ± 5.0

a We have previously reported the cytotoxicity of (−)-1.^10^

## 3 Conclusions

In summary, we have synthesized deoxygenated derivatives of uvaridacol L (±)-2–4 by building a cyclohex-1-en-1-ylmethanol skeleton from *myo*-inositol. Moreover, we discovered that the direction of acid-catalyzed double bond migration from methoxy olefin to a key intermediate in this synthesis depends on the type of adjacent substituent (ester or ether) and the geometric isomer of the intermediate methoxy olefin. We have found that the regioselectivity of migration of the double-bond depends on the electronic interactions of the BO and the ABO by using DFT calculations. These results provide useful guidance for the synthesis of carbasugars using *myo*-inositol as the starting material. SAR of antiausterity agent uvaridacol L (1) was also performed using (±)-2–4 to show that the ester carbonyl oxygen of uvaridacol L (1) is not essential for its antiausterity properties.

## 4 Experimental

### 4.1 General method

All reactions were carried out under an ambient atmosphere in a round bottom flask containing a stir-bar with a rubber septum except as described otherwise. Anhydrous tetrahydrofuran (THF) and CH_2_Cl_2_ were purchased from FUJIFILM Wako Pure Chemical Corporation (Osaka, Japan) and used without further purification. All other reagents were purchased from Tokyo Chemical Industry Co. (Tokyo, Japan), Kishida Chemical Co. (Osaka, Japan), Nacalai Tesque Inc. (Kyoto, Japan), or FUJIFILM Wako Pure Chemical Corporation and used without further purification. Silicycle Inc. (Quebec, Canada) silica gel (SiliaFlash® F60, 40–63 μm, #R10030B) or Chromatorex PSQ60B (Fuji Silysia Chemical Ltd, Kasugai, Japan) was used for flash column chromatography.

### 4.2 Analytical methods

All reactions were monitored by thin-layer chromatography with E. Merck silica gel 60 F_254_ pre-coated plates (0.25 mm, 1.05715.0001) and were visualized by UV (254 nm). ^1^H and ^13^C NMR spectra were recorded on a JEOL ECZ400S spectrometer (^1^H: 400 MHz, ^13^C: 100 MHz) instrument. Chemical shifts in ^1^H NMR are reported in ppm relative to the residual protons of deuterated solvents (acetone-*d*_6_: 2.04 ppm) or the internal standard tetramethylsilane (CDCl_3_: 0.00 ppm for ^1^H). Chemical shifts in ^13^C NMR are reported in ppm relative to the carbon of deuterated solvent (CDCl_3_: 77.0 ppm, acetone-*d*_6_: 29.8 ppm). The mass spectra were measured on a Thermo Fisher Scientific LTQ Orbitrap Discovery. Melting points were determined by Yanaco micro melting point apparatus MP-J3. Specific rotations were measured with JASCO DIP-370 digital polarimeter using the sodium D line and are reported as follows: [*α*]^t^_D_ (*c* = 10 mg mL^−1^, solvent). Yield refers to isolated yields of compounds greater than 95% purity as determined by ^1^H NMR analysis. New compounds ((±)-16, (±)-12, (±)-17, (±)-3, (±)-18, (±)-4, and (±)-2) were characterized by ^1^H NMR, ^13^C NMR, and HRMS. *E* or *Z*-isomers ((±)-9 and (±)-10) were identified unambiguously by ^1^H NMR, ^13^C NMR, COSY, and NOESY. (±)-6 and (±)-7 were identified unambiguously by ^1^H NMR, ^13^C NMR, COSY, HMBC, and HMQC. Known compounds (14,^20^15,^20^ and (±)-19 ^17^) were synthesized according to the literature. We have previously reported preparation of (+)-uvaridacol L ((+)-1)^10^ and its [*α*]^25^_D_ was +89.3 (*c* = 0.3, CHCl_3_).

### 4.3 Experimental procedure for synthesis

#### 4.3.1 Synthesis of (1*R*,3*R*,5*R*,6*R*,7*S*,8*R*,9*S*)-8,9-bis(benzyloxy)-2,4,10-trioxaadamantan-6-ol (14)

To a solution of 13 (190 mg, 1.0 mmol) in dry DMF (20 mL), sodium hydride (60% oil suspension, 160 mg, 4.0 mmol) was added and stirred at room temperature for 35 min under Ar atmosphere. To the mixture, benzyl bromide (240 μL, 2.0 mmol) was added dropwise by syringe over 5 min and the mixture was stirred at room temperature for 110 min. The reaction was quenched by the addition of H_2_O at room temperature and the mixture was extracted with hexane one time, hexane/EtOAc = 5 : 1 three times, and hexane/EtOAc = 3 : 1 two times. The combined organic layer was dried over anhydrous Na_2_SO_4_ and evaporated under reduced pressure. The residue was purified by column chromatography (CHCl_3_) to afford 14 (264 mg, 71%) as a colorless solid. Rf: 0.58 (hexane/EtOAc = 1 : 1). ^1^H NMR (400 MHz, CDCl_3_) *δ*: 3.00 (1H, d, *J* = 11.2 Hz), 4.19–4.24 (3H, m), 4.37 (2H, t, *J* = 3.6 Hz), 4.45–4.48 (1H, m), 4.59 (2H, d, *J* = 11.2 Hz), 4.67 (2H, d, *J* = 11.2 Hz), 5.47 (s, 1H), 7.28–7.30 (m, 10H). ^1^H NMR spectrum of 14 is consistent with the reported data.^21^

#### 4.3.2 Synthesis of (1*S*,3*S*,5*R*,7*S*,8*R*,9*S*)-8,9-bis(benzyloxy)-2,4,10-trioxaadamantan-6-one (15)

To a solution of 14 (411 mg, 1.1 mmol) and tetrabutylammonium bromide (TBAB, 35.5 mg, 0.11 mmol) in CH_2_Cl_2_/H_2_O (5.5 mL/5.5 mL), NaClO·5H_2_O (362 mg, 2.2 mmol) was added and stirred at room temperature. To the solution, TEMPO (17.2 mg, 0.11 mmol) was added and vigorously stirred at room temperature for 21 h. The mixture was diluted with 10% aqueous Na_2_SO_3_ and extracted with CHCl_3_ three times. The combined organic layer was dried over anhydrous Na_2_SO_4_ and evaporated under reduced pressure. The residue was purified by column chromatography (CHCl_3_) to afford 15 and its corresponding geminal diol. The afforded solid was dissolved in toluene and refluxed with Dean–Stark apparatus overnight. After cooling to room temperature, the solution was evaporated under reduced pressure to afford 15 (359 mg, 88%) as a pale yellow solid. Rf: 0.43 (CHCl_3_/MeOH = 20 : 1). ^1^H NMR (400 MHz, CDCl_3_) *δ*: 4.44–4.45 (2H, m), 4.55–4.59 (3H, m), 4.59 (2H, d, *J* = 12.0 Hz), 4.61 (2H, d, *J* = 12.0 Hz), 5.65 (1H, s), 7.23–7.30 (10H, m). ^1^H NMR spectrum of 15 is consistent with the reported data.^21^

#### 4.3.3 Synthesis of (1*S*,3*R*,5*R*,7*S*,8*R*,9*S*)-8-(benzyloxy)-9-hydroxy-2,4,10-trioxaadamantan-6-one ((±)-16)

To a suspension of 15 (146 mg, 0.40 mmol) in MeOH (8 mL), 10% Pd/C (42.0 mg, 0.04 mmol) was added and performed hydrogen displacement several times using a vacuum pump. The suspension was stirred at 40 °C for 24 h. After cooling to room temperature, hydrogen displacement was again performed several times, and the mixture was stirred at 40 °C for 24 h. After cooling to room temperature, the mixture was filtrated through a short pad of Celite. The filtrate was evaporated under reduced pressure. The residue was purified by column chromatography (hexane/EtOAc = 2 : 1 to 1 : 1) to afford (±)-16 and its corresponding geminal diol. The afforded oil was dissolved in toluene and refluxed with Dean–Stark apparatus overnight. After cooling to room temperature, the solution was evaporated under reduced pressure to afford (±)-16 (67.3 mg, 61%) as a pale yellow oil. Rf: 0.63 (CHCl_3_/MeOH = 20 : 1). ^1^H NMR (400 MHz, CDCl_3_) *δ*: 3.59 (1H, d, *J* = 11.2 Hz), 4.41 (1H, dt, *J* = 4.4, 2.0 Hz), 4.45 (1H, tt, *J* = 3.2, 2.0 Hz), 4.49 (1H, dt, *J* = 4.4, 2.0 Hz), 4.56 (1H, d, *J* = 11.6 Hz), 4.63 (1H, dd, *J* = 7.2, 3.2 Hz), 4.68 (1H, tt, *J* = 7.2, 3.2 Hz), 4.73 (1H, d, *J* = 11.6 Hz), 5.66 (1H, s), 7.28–7.30 (2H, m), 7.36–7.41 (3H, m); ^13^C NMR (100 MHz, CDCl_3_) *δ*: 68.5, 71.5, 72.7, 76.8, 77.4, 80.7, 102.1, 128.3 (2C), 128.9 (2C), 129.0, 135.2, 200.3; HR-ESI-MS *m*/*z* 301.0684 (calcd for C_14_H_14_O_6_Na [M + Na^+^]: 301.0683).

#### 4.3.4 Synthesis of (1*S*,3*S*,5*R*,6*S*,7*R*,8*R*)-8-(benzyloxy)-9-oxo-2,4,10-trioxaadamantan-6-yl benzoate ((±)-12)

To a solution of 16 (372 mg, 1.34 mmol), 4-dimethylaminopyridine (DMAP, 16.0 mg, 0.13 mmol) in dry CH_2_Cl_2_ (13 mL), triethylamine (700 μL, 5.02 mmol) was added, and stirred at room temperature for 10 min. Benzoyl chloride (280 μL, 2.41 mmol) was added and the mixture was stirred at room temperature for 16.5 h. The reaction was quenched by the addition of H_2_O at room temperature and the mixture was diluted with saturated aqueous NaHCO_3_ and extracted with CHCl_3_ three times. The combined organic layer was dried over anhydrous Na_2_SO_4_ and evaporated under reduced pressure. The residue was purified by column chromatography (hexane/EtOAc = 3 : 1) to afford (±)-12 (382 mg, 75%) as a colorless solid. Rf: 0.33 (hexane/EtOAc = 3 : 1). Mp: 114–116 °C. ^1^H NMR (400 MHz, acetone-*d*_6_) *δ*: 4.42 (1H, dt, *J* = 4.0, 2.0 Hz), 4.50 (1H, d, *J* = 11.6 Hz), 4.56 (1H, dt, *J* = 4.0, 2.0 Hz), 4.62 (1H, d, *J* = 11.6 Hz), 4.65 (1H, td, *J* = 3.6, 2.8 Hz), 4.79 (1H, tt, *J* = 3.6, 2.0 Hz), 5.74 (1H, td, *J* = 3.6, 2.8 Hz), 5.79 (1H, s), 7.09–7.13 (5H, m), 7.17 (2H, t, *J* = 8.0 Hz), 7.43 (1H, tt, *J* = 8.0, 1.6 Hz), 7.67 (2H, d, *J* = 8.0 Hz); ^13^C NMR (100 MHz, acetone-*d*_6_) *δ*: 68.7, 72.0, 72.7, 77.7, 77.9, 78.4, 103.4, 128.6, 128.7 (2C), 129.1 (2C), 129.3 (2C), 129.8, 130.5 (2C), 134.3, 138.1, 165.5, 200.0; HR-ESI-MS *m*/*z* 405.0948 (calcd for C_21_H_18_O_7_Na [M + Na^+^]: 405.0945).

#### 4.3.5 Synthesis of (1*R*,3*S*,5*S*,6*R*,7*R*,8*S*,*E*)-8-(benzyloxy)-9-(methoxymethylene)-2,4,10-trioxaadamantan-6-yl benzoate ((±)-9) and (1*R*,3*S*,5*S*,6*R*,7*R*,8*S*,*Z*)-8-(benzyloxy)-9-(methoxymethylene)-2,4,10-trioxaadamantan-6-yl benzoate ((±)-10)

To a suspension of (methoxymethyl)triphenylphosphonium chloride (216 mg, 0.63 mmol) in dry THF (6 mL), potassium *tert*-butoxide (67 mg, 0.60 mmol) was added and stirred at room temperature under Ar atmosphere. After 20 min, the color of solution changed to red-orange. (±)-12 (115 mg, 0.30 mmol) was added to the reaction mixture and stirred at 60 °C for 3 h. After cooling to room temperature, the reaction mixture was diluted with saturated aqueous NH_4_Cl and extracted with CHCl_3_ three times. The combined organic layer was dried over anhydrous Na_2_SO_4_ and evaporated under reduced pressure. The residue was purified by column chromatography (hexane/EtOAc = 2 : 1) to afford isomeric mixture of (±)-9 and (±)-10 (100 mg, 81%) as a colorless solid. The isomeric mixture was separated by column chromatography (hexane/EtOAc = 5 : 1) to afford (±)-9 (30 mg, 24%) and (±)-10 (48 mg, 39%) as a colorless solid.

##### 4.3.5.1 (±)-9 (*E*-isomer)

Rf: 0.50 (hexane/EtOAc = 2 : 1). Mp: 86–89 °C. ^1^H NMR (400 MHz, CDCl_3_) *δ*: 3.69 (3H, s), 4.38 (1H, td, *J* = 3.6, 0.8 Hz), 4.48 (1H, dt, *J* = 3.6, 2.0 Hz), 4.52 (1H, d, *J* = 11.2 Hz), 4.64 (1H, tt, *J* = 2.0, 3.6 Hz), 4.67 (1H, d, *J* = 11.2 Hz), 5.24–5.25 (1H, m), 5.59 (1H, td, *J* = 3.6, 0.8 Hz), 5.72 (1H, s), 6.26 (1H, s), 7.17 (2H, t, *J* = 8.0 Hz), 7.25–7.28 (5H, m), 7.45 (1H, tt, *J* = 8.0, 1.2 Hz), 7.84 (2H, dd, *J* = 8.0, 1.2 Hz); ^13^C NMR (100 MHz, CDCl_3_) *δ*: 60.3, 66.4, 68.0, 69.1, 70.5, 71.5, 73.4, 104.7, 106.3, 127.7, 127.9 (2C), 128.17 (2C), 128.25 (2C), 129.5, 129.8 (2C), 133.0, 137.5, 145.7, 165.5; HR-ESI-MS *m*/*z* 433.1259 (calcd for C_23_H_22_O_7_Na [M + Na^+^]: 433.1258).

##### 4.3.5.2 (±)-10 (*Z*-isomer)

Rf: 0.47 (hexane/EtOAc = 2 : 1). Mp: 89–91 °C. ^1^H NMR (400 MHz, CDCl_3_) *δ*: 3.57 (3H, s), 4.30 (1H, t, *J* = 3.2 Hz), 4.40 (1H, dt, *J* = 3.2, 1.6 Hz), 4.52 (1H, d, *J* = 12.0 Hz), 4.56 (1H, d, *J* = 12.0 Hz), 4.74 (1H, tt, *J* = 3.2, 1.6 Hz), 5.14 (1H, dd, *J* = 3.6, 1.6 Hz), 5.62 (1H, t, *J* = 3.6 Hz), 5.70 (1H, s), 6.23 (1H, s), 7.16–7.30 (7H, m), 7.50 (1H, t, *J* = 7.2 Hz), 7.94 (2H, d, *J* = 7.2 Hz); ^13^C NMR (100 MHz, CDCl_3_) *δ*: 60.2, 65.9, 67.8, 68.5, 71.4, 71.7, 73.9, 104.6, 106.0, 127.7 (2C), 127.8, 128.2 (2C), 128.3 (2C), 129.6, 129.9 (2C), 133.0, 137.5, 145.7, 165.6; HR-ESI-MS *m*/*z* 433.1260 (calcd for C_23_H_22_O_7_Na [M + Na^+^]: 433.1258).

#### 4.3.6 Synthesis of (1*R*,4*R*,5*S*,6*S*)-5-(benzyloxy)-3-formyl-4,6-dihydroxycyclohex-2-en-1-yl benzoate ((±)-6) and (1*S*,2*R*,5*R*,6*S*)-5-(benzyloxy)-3-formyl-2,6-dihydroxycyclohex-3-en-1-yl benzoate ((±)-7)

To a solution of isomeric mixtures (±)-9 and (±)-10 (155 mg, 0.38 mmol, *E* : *Z* = 36 : 64) in THF (3.8 mL), 1 M aqueous HCl (3.8 mL) was added and stirred at 40 °C for 25 h. After cooling to room temperature, the reaction mixture was diluted with H_2_O and extracted with EtOAc three times. The combined organic layer was dried over anhydrous Na_2_SO_4_ and evaporated under reduced pressure. The residue was purified by column chromatography (hexane/EtOAc = 1 : 1) to afford (±)-6 (38.6 mg, 28%) and (±)-7 (83.2 mg, 60%) as a colorless solid.

(±)-6 Rf: 0.67 (hexane/EtOAc = 1 : 1). Mp: 140–143 °C. ^1^H NMR (400 MHz, CDCl_3_) *δ*: 2.84 (1H, d, *J* = 1.6 Hz), 3.66 (1H, d, *J* = 2.4 Hz), 3.76 (1H, dd, *J* = 10.0, 6.8 Hz), 3.99 (1H, tt, *J* = 10.0, 1.6 Hz), 4.81 (1H, d, *J* = 11.6 Hz), 4.83–4.86 (1H, m), 5.13 (1H, d, *J* = 11.6 Hz), 5.90 (1H, dt, *J* = 8.0, 2.4 Hz), 6.67 (1H, s), 7.31–7.42 (5H, m), 7.46 (2H, t, *J* = 8.0 Hz), 7.60 (1H, tt, *J* = 8.0, 1.6 Hz), 8.07 (2H, dd, *J* = 8.0, 1.6 Hz), 9.53 (1H, s); ^13^C NMR (100 MHz, CDCl_3_) *δ*: 70.9, 71.1, 73.1, 75.1, 81.9, 128.1, 128.2 (2C), 128.5 (2C), 128.6 (2C), 129.1, 129.9 (2C), 133.6, 137.9, 140.6, 145.2, 166.0, 194.1; HR-ESI-MS *m*/*z*: 391.1150 (calcd for C_21_H_20_O_6_Na [M + Na^+^]: 391.1152).

(±)-7 Rf: 0.47 (hexane/EtOAc = 1 : 1). Mp: 121–123 °C. ^1^H NMR (400 MHz, CDCl_3_) *δ*: 2.65 (1H, d, *J* = 4.4 Hz), 3.57 (1H, d, *J* = 2.0 Hz), 4.02 (1H, ddd, *J* = 10.0, 7.6, 4.0 Hz), 4.41 (1H, dt, *J* = 7.6, 2.0 Hz), 4.85 (1H, d, *J* = 11.6 Hz), 4.87 (1H, d, *J* = 11.6 Hz), 4.85–4.88 (1H, m), 5.39 (1H, dd, *J* = 10.0, 7.6 Hz), 6.70–6.71 (1H, m), 7.32–7.42 (5H, m), 7.45 (2H, t, *J* = 7.6 Hz), 7.59 (1H, tt, *J* = 8.0, 1.2 Hz), 8.09 (2H, dd, *J* = 8.0, 1.2 Hz), 9.53 (1H, s); ^13^C NMR (100 MHz, CDCl_3_) *δ*: 68.4, 72.7, 73.5, 76.1, 78.3, 128.1 (2C), 128.3, 128.4 (2C), 128.7 (2C), 129.4, 130.0 (2C), 133.4, 137.2, 139.2, 147.0, 166.9, 193.6; HR-ESI-MS *m*/*z*: 391.1151 (calcd for C_21_H_20_O_6_Na [M + Na^+^]: 391.1152).

#### 4.3.7 The ring-opening reactions of each of the isolated isomers ((±)-9 and (±)-10)

##### 4.3.7.1 The reaction of (±)-9 (*E*-isomer)

To a solution of (±)-9 (31.0 mg, 0.076 mmol) in THF (0.7 mL), 1 M aqueous HCl (0.7 mL) was added and stirred at 40 °C for 22.5 h. After cooling to room temperature, the reaction mixture was diluted with H_2_O and extracted with EtOAc three times. The combined organic layer was dried over anhydrous Na_2_SO_4_ and evaporated under reduced pressure. The residue was purified by column chromatography (CHCl_3_) to afford (±)-6 (11 mg, 40%) and (±)-7 (10 mg, 36%) as a colorless solid.

##### 4.3.7.2 The reaction of (±)-10 (*Z*-isomer)

To a solution of (±)-10 (48.0 mg, 0.12 mmol) in THF (0.7 mL), 1 M aqueous HCl (0.7 mL) was added and stirred at 40 °C for 24 h. After cooling to room temperature, the reaction mixture was diluted with H_2_O and extracted with EtOAc three times. The combined organic layer was dried over anhydrous Na_2_SO_4_ and evaporated under reduced pressure. The residue was purified by column chromatography (CHCl_3_) to afford (±)-7 (30 mg, 70%) as a colorless solid.

#### 4.3.8 Synthesis of (1*R*,4*R*,5*S*,6*S*)-5-(benzyloxy)-4,6-dihydroxy-3-(hydroxymethyl)cyclohex-2-en-1-yl benzoate ((±)-17)

(±)-6 (29.0 mg, 0.079 mmol) and CeCl_3_·7H_2_O (32.0 mg, 0.086 mmol) were dissolved in MeOH (1 mL) and cooled to −60 °C. After 30 min, sodium borohydride (3.0 mg, 0.079 mmol) was added portionwise and stirred at 0 °C for 2 h. The reaction was quenched by the addition of saturated aqueous NH_4_Cl at 0 °C. The mixture was filtrated through a short pad of Celite. The filtrate was extracted with EtOAc three times. The combined organic layer was dried over anhydrous Na_2_SO_4_ and evaporated under reduced pressure. The residue was purified by column chromatography (CHCl_3_ to CHCl_3_/MeOH = 9 : 1) to afford (±)-17 (28 mg, 96%) as a colorless solid. Rf: 0.57 (CHCl_3_/MeOH = 9 : 1). Mp: 135–137 °C. ^1^H NMR (400 MHz, CDCl_3_) *δ*: 2.14 (1H, t, *J* = 2.0 Hz), 2.65 (1H, d, *J* = 4.0 Hz), 2.98 (1H, d, *J* = 2.4 Hz), 3.67 (1H, dd, *J* = 10.0, 8.0 Hz), 4.03 (1H, ddd, *J* = 10.0, 7.2, 2.4 Hz), 4.27 (2H, d, *J* = 4.0 Hz), 4.50 (1H, dd, *J* = 7.2, 2.0 Hz), 4.88 (1H, d, *J* = 12.0 Hz), 4.99 (1H, d, *J* = 12.0 Hz), 5.67 (1H, s), 5.68–5.70 (1H, m), 7.31–7.42 (5H, m), 7.45 (2H, t, *J* = 7.6 Hz), 7.58 (1H, tt, *J* = 7.6, 1.2 Hz), 8.06 (2H, dd, *J* = 7.6, 1.2 Hz); ^13^C NMR (100 MHz, CDCl_3_) *δ*: 64.0, 72.6, 73.5, 75.2, 75.4, 83.8, 121.9, 128.1 (2C), 128.2, 128.4 (2C), 128.8 (2C), 129.6, 129.8 (2C), 133.3, 138.2, 140.3, 166.8; HR-ESI-MS: *m*/*z* 393.1314 (calcd for C_21_H_22_O_6_Na [M + Na^+^]: 393.1309).

#### 4.3.9 Synthesis of [(3*R*,4*S*,5*S*,6*R*)-3-(benzoyloxy)-5-(benzyloxy)-4,6-dihydroxycyclohex-1-en-1-yl]methyl benzoate ((±)-3)

To a solution of (±)-17 (27.5 mg, 0.074 mmol) in dry CH_2_Cl_2_ (1.5 mL), 2,4,6-collidine (35 μL, 0.27 mmol) was added and stirred at room temperature under Ar atmosphere. After 20 min, benzoyl chloride (11 μL, 0.095 mmol) was added to the solution and stirred at room temperature for 5.5 h. The reaction was quenched by the addition of H_2_O, diluted with 1 M aqueous HCl and extracted with CHCl_3_ three times. The combined organic layer was dried over anhydrous Na_2_SO_4_ and evaporated under reduced pressure. The residue was purified by column chromatography (CHCl_3_) to afford (±)-3 (29.3 mg, 83%) as a colorless solid. Rf: 0.67 (CHCl_3_/MeOH = 9 : 1). Mp: 142–144 °C. ^1^H NMR (400 MHz, CDCl_3_) *δ*: 2.89 (1H, d, *J* = 2.4 Hz), 3.15 (1H, d, *J* = 4.8 Hz), 3.70 (1H, dd, *J* = 10.0, 7.6 Hz), 4.03 (1H, ddd, *J* = 10.0, 8.0, 2.4 Hz), 4.40–4.44 (1H, m), 4.70 (1H, d, *J* = 13.2 Hz), 4.93 (1H, d, *J* = 11.6 Hz), 4.99 (1H, d, *J* = 11.6 Hz), 5.22 (1H, d, *J* = 13.2 Hz), 5.70–5.72 (1H, m), 5.83 (1H, s), 7.30–7.42 (5H, m), 7.44 (2H, t, *J* = 8.0 Hz), 7.45 (2H, t, *J* = 7.6 Hz), 7.57 (1H, t, *J* = 7.6 Hz), 7.59 (1H, d, *J* = 8.0 Hz), 8.05 (2H, dd, *J* = 7.6, 1.2 Hz), 8.06 (2H, dd, *J* = 8.0, 1.6 Hz); ^13^C NMR (100 MHz, CDCl_3_) *δ*: 64.2, 71.4, 72.9, 74.5, 75.3, 83.4, 124.9, 128.1 (3C), 128.4 (2C), 128.5 (2C), 128.7 (2C), 129.5, 129.6, 129.8 (2C), 129.9 (2C), 133.3, 133.4, 137.0, 138.1, 166.5, 166.9; HR-ESI-MS: *m*/*z* 497.1575 (calcd for C_28_H_26_O_7_Na [M + Na^+^]: 497.1571).

#### 4.3.10 Synthesis of (1*S*,2*R*,5*R*,6*S*)-5-(benzyloxy)-2,6-dihydroxy-3-(hydroxymethyl)cyclohex-3-en-1-yl benzoate ((±)-18)

(±)-7 (69.0 mg, 0.19 mmol) and CeCl_3_·7H_2_O (76.6 mg, 0.21 mmol) were dissolved in MeOH (2 mL) and cooled to −60 °C. After 1 h, sodium borohydride (7.1 mg, 0.19 mmol) was added portionwise and stirred at 0 °C for 2 h. The reaction was quenched by the addition of saturated aqueous NH_4_Cl at 0 °C. The mixture was filtrated through a short pad of Celite. The filtrate was extracted with EtOAc three times. The combined organic layer was dried over anhydrous Na_2_SO_4_ and evaporated under reduced pressure. The residue was purified by column chromatography (CHCl_3_ to CHCl_3_/MeOH = 9 : 1) to afford (±)-18 (68 mg, 98%) as a hygroscopic colorless solid. Rf: 0.50 (CHCl_3_/MeOH = 9 : 1). ^1^H NMR (400 MHz, CDCl_3_) *δ*: 2.52 (1H, br s), 2.68 (1H, br s), 3.59 (1H, br s), 3.99 (1H, t, *J* = 8.8 Hz), 4.17 (1H, d, *J* = 8.0 Hz), 4.22 (2H, s), 4.56 (1H, d, *J* = 8.0 Hz), 4.71 (1H, d, *J* = 11.6 Hz), 4.76 (1H, d, *J* = 11.6 Hz), 5.19 (1H, dd, *J* = 11.6 Hz), 5.78 (1H, s), 7.28–7.39 (5H, m), 7.44 (2H, t, *J* = 8.0 Hz), 7.59 (1H, tt, *J* = 8.0, 1.2 Hz), 8.07 (2H, dd, *J* = 8.0, 1.2 Hz); ^13^C NMR (100 MHz, CDCl_3_) *δ*: 64.0, 72.0, 72.4, 73.0, 78.7, 78.8, 123.9, 127.9 (2C), 128.0, 128.49 (2C), 128.54 (2C), 129.3, 129.9 (2C), 133.6, 137.8, 138.2, 167.8; HR-ESI-MS: *m*/*z* 393.1312 (calcd for C_21_H_22_O_6_Na [M + Na^+^]: 393.1309).

#### 4.3.11 Synthesis of (1*S*,2*R*,5*R*,6*S*)-5-(benzyloxy)-2,6-dihydroxy-3-(hydroxymethyl)cyclohex-3-en-1-yl benzoate ((±)-4)

To a solution of (±)-18 (25.0 mg, 0.067 mmol) in dry CH_2_Cl_2_ (1.5 mL), 2,4,6-collidine (30 μL, 0.23 mmol) was added and stirred at room temperature under Ar atmosphere. After 5 min, benzoyl chloride (10 μL, 0.086 mmol) was added to the solution and stirred at room temperature for 24 h. The reaction was quenched by the addition of H_2_O, diluted with 1 M aqueous HCl and extracted with CHCl_3_ three times. The combined organic layer was dried over anhydrous Na_2_SO_4_ and evaporated under reduced pressure. The residue was purified by column chromatography (CHCl_3_ to hexane/EtOAc = 1 : 1) to afford (±)-4 (22.6 mg, 71%) as a colorless solid. Rf: 0.63 (hexane/EtOAc = 1 : 1). Mp: 41–44 °C. ^1^H NMR (400 MHz, CDCl_3_) *δ*: 2.47 (1H, d, *J* = 2.8 Hz), 3.33 (1H, d, *J* = 5.2 Hz), 4.06 (1H, ddd, *J* = 10.0, 8.0, 2.0 Hz), 4.22 (1H, d, *J* = 8.0 Hz), 4.52–4.56 (1H, m), 4.77 (2H, s), 4.79 (1H, d, *J* = 13.2 Hz), 5.15 (1H, d, *J* = 13.2 Hz), 5.26 (1H, dd, *J* = 10.0, 8.0 Hz), 5.93 (1H, s), 7.29–7.38 (5H, m), 7.46 (4H, t, *J* = 8.0 Hz), 7.59 (1H, t, *J* = 8.0 Hz), 7.60 (1H, t, *J* = 8.0 Hz), 8.05 (2H, dd, *J* = 8.0, 1.6 Hz), 8.10 (2H, dd, *J* = 8.0, 1.6 Hz); ^13^C NMR (100 MHz, CDCl_3_) *δ*: 64.2, 70.6, 72.6, 73.1, 78.3, 78.8, 126.5, 127.96 (2C), 127.99, 128.5 (4C), 128.6 (2C), 129.4, 129.7, 129.8 (2C), 130.0 (2C), 133.3, 133.5, 135.0, 137.8, 166.6, 167.6; HR-ESI-MS: *m*/*z* 497.1568 (calcd for C_28_H_26_O_7_Na [M + Na^+^]: 497.1571).

#### 4.3.12 Synthesis of (1*S*,2*S*,3*R*,6*R*)-5-[(benzyloxy)methyl]-2,6-dihydroxycyclohex-4-ene-1,3-diyl dibenzoate ((±)-2)

To a solution of (±)-19 ^17^ (230 mg, 0.60 mmol) and TfOH (20 μL, 0.23 mmol) in 1,4-dioxane (6 mL), TriBOT (96.0 mg, 0.24 mmol) was added and stirred at room temperature for 6 h. The reaction mixture was quenched by the addition of saturated aqueous NaHCO_3_ and extracted with CHCl_3_ three times. The combined organic layer was dried over anhydrous Na_2_SO_4_ and evaporated under reduced pressure. The residue was purified by column chromatography (hexane/EtOAc = 2 : 1) to afford (±)-2 (48 mg, 17%) as a colorless solid. Rf: 0.27 (hexane/EtOAc = 2 : 1). Mp: 42–44 °C. ^1^H NMR (400 MHz, CDCl_3_) *δ*: 3.06 (1H, d, *J* = 5.6 Hz), 3.23 (1H, d, *J* = 5.2 Hz), 4.14 (1H, d, *J* = 12.0 Hz), 4.24 (1H, dt, *J* = 9.2, 5.6 Hz), 4.28 (1H, d, *J* = 12.0 Hz), 4.56 (1H, d, *J* = 12.0 Hz), 4.59 (1H, d, *J* = 12.0 Hz), 4.59–4.62 (1H, m), 5.40 (1H, dd, *J* = 9.2, 6.8 Hz), 5.74 (1H, ddt, *J* = 6.8, 4.8, 2.0 Hz), 5.86–5.87 (1H, m), 7.28–7.35 (5H, m), 7.38 (2H, t, *J* = 8.0 Hz), 7.43 (2H, t, *J* = 8.0 Hz), 7.55 (1H, tt, *J* = 8.0, 1.2 Hz), 7.58 (1H, tt, *J* = 8.0, 1.2 Hz), 8.00 (2H, dd, *J* = 8.0, 1.2 Hz), 8.08 (2H, dd, *J* = 8.0, 1.2 Hz); ^13^C NMR (100 MHz, acetone-*d*_6_) *δ*: 70.1, 70.8, 72.0, 73.1, 76.1, 78.6, 121.9, 128.2, 128.5 (2C), 129.0 (2C), 129.2 (2C), 129.3 (2C), 130.3 (2C), 130.5 (2C), 131.2, 131.7, 133.7, 134.0, 139.6, 140.7, 166.4, 166.5; HR-ESI-MS: *m*/*z* 497.1571 (calcd for C_28_H_26_O_7_Na [M + Na^+^]: 497.1571).

### 4.4 Procedure of biological assay

#### 4.4.1 Cell culture

Human pancreatic cancer cell line PANC-1 was cultured in low glucose DMEM (Nacalai Tesque Inc., #08456-65 or Gibco #C11885500BT) supplemented with heat-inactivated 10% fetal bovine serum (FBS, PAN Biotech GmbH, Lot: P180803). Human colon cancer cell line HT-29 was cultured in RPMI-1640 medium (Nacalai Tesque Inc., #30264-85 or Gibco, #C11875500BT) supplemented with heat-inactivated 10% FBS (Gibco, lot: 42Q4173K). Every medium was supplemented with penicillin G potassium (50 units/mL, Meiji Seika Pharma Co), streptomycin sulfate (50 μg mL^−1^, Meiji Seika Pharma Co.), and kanamycin sulfate (50 μg mL^−1^, Meiji Seika Pharma Co.), and the cultured cells were maintained in a humidified atmosphere of 5% CO_2_ and 95% air at 37 °C. PANC-1 was grown in high glucose DMEM (Nacalai Tesque Inc., #08458-45 or Gibco #C11995500BT) supplemented with FBS and antibiotics described above for cell growth. A subculture was performed once or twice per week from subconfluent cultures using a trypsin–ethylenediaminetetraacetic acid (EDTA) solution (10 times diluted FUJIFILM Wako Pure Chemical Corporation, #208-17251 or 10 times diluted Nacalai Tesque Inc., #35556-44). Glucose and sodium pyruvate free DMEM (#09891-25), glucose and sodium pyruvate free RPMI-1640 (#09892-15) were purchased from Nacalai Tesque Inc. Nutrient deprived medium (NDM) to mimic tumor microenvironment is as follows: 25 mM *N*-(2-hydroxyethyl)-piperazine-*N*′-2-ethanesulfonic acid (HEPES) supplemented with 6.4 g L^−1^ NaCl, 700 mg L^−1^ NaHCO_3_, 400 mg L^−1^ KCl, 265 mg L^−1^ CaCl_2_·2H_2_O, 200 mg L^−1^ MgSO_4_·7H_2_O, 109 mg L^−1^ NaH_2_PO_4_, 0.1 mg L^−1^ Fe(NO_3_)·9H_2_O, 15 mg L^−1^ phenol red, 40 mL L^−1^ MEM vitamin solution (100×) (Gibco, Carlsbad, CA).

#### 4.4.2 Assay for growth inhibitory activity under nutrient deprived conditions

PANC-1 cells in low glucose DMEM with 10% FBS were seeded into each well of 96-well plates (2.0 × 10^4^ cells per well per 100 μL), cell culture 96-well plate, flat bottom (TPP Techno Plastic Products AG Trasadingen, Switzerland, #92696) then incubated for 24 h in a humidified atmosphere of 5% CO_2_ and 95% air at 37 °C. After removal of the medium, the cells in each well were rinsed with 100 μL of phosphate buffered saline (PBS(−)). Then, the plates were incubated in either (a) low glucose DMEM with 10% FBS (+FBS), (b) FBS-free low glucose DMEM (−FBS), (c) FBS, sodium pyruvate and glucose-free DMEM (−FBS, −SP, and −Glc), or (d) NDM with the test compounds (1% DMSO) for 24 h. After the incubation, 10% WST-8 cell counting kit solution (Kishida Chemical Co., #260-96160) in low glucose DMEM with 10% FBS (100 μL) was added to the each well. After 2–4 h incubation, each absorbance at 450 nm (Abs_450_) to quantify metabolite formazan and at 650 nm (Abs_650_) as background was measured (Molecular Devices Inc. SpectraMax iD5 multiplate reader). Cell viability was calculated from the mean values of two wells by using the following equation:
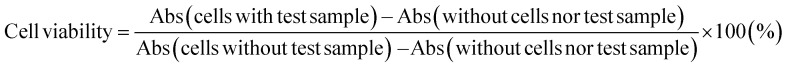
Abs = Abs_450_ − Abs_650_

Each experiment was performed in duplicate and repeated independently. The WST-8 assay on HT-29 cells was also performed according to the method on PANC-1 cells except that the medium was changed to RPMI-1640 medium.

#### 4.4.3 Statistical analysis

Data were analyzed using Prism software (ver. 9.5.1, GraphPad Software, Inc.). Data presented are expressed as the mean ± standard error of the mean (SEM). Statistical significance was calculated using a two-way ANOVA followed by the Tukey's comparison test for multiple comparisons with a significance level of *P* < 0.05.

## Author contributions

KO conceived the project. All authors designed the experiments. KU, AT and KO performed the experiments and analyzed the data. All authors contributed to manuscript preparation and revision.

## Conflicts of interest

There are no conflicts of interest to declare.

## Note added after first publication

This article replaces the version published on 19 June 2025, which included an incorrectly placed equation. The text of the article remains unchanged.

## Supplementary Material

RA-015-D5RA01049G-s001

## Data Availability

The data underlying this study are available in the published article and its ESI.†
