# Supplementary material for: Elucidation of the acid reactivity of polyhedral orthoformates for the synthesis of carbasugar derivatives
Source: RSC Adv. 2025 Jun 19;15(26):20734–44. doi: 10.1039/d5ra01049g (PMC12178235; doi:10.1039/d5ra01049g)
Supplement: RA-015-D5RA01049G-s001 [file RA-015-D5RA01049G-s001.pdf]

Supplementary Information (SI) for RSC Advances.
This journal is © The Royal Society of Chemistry 2025

Supplementary Information

Elucidation of the Acid Reactivity of Polyhedral Orthoformates for the Synthesis of Carbasugar Derivatives

Kazuki Usuguchi, Akira Takagi, Ippei Takashima,† and Kensuke Okuda*

Laboratory of Bioorganic & Natural Products Chemistry, Kobe Pharmaceutical University, 4–19–1 Motoyamakita, Higashinada, Kobe 658–8558, Japan.

†present address: National Institute of Technology, Hakodate College, 14-1, Tokurachou, Hakodate, Hokkaido, 042-8501, Japan

* Correspondence phone/facsimile: +81-78-441-7545, e-mail: okuda@kobepharma-u.ac.jp (K.O.)

Table of Contents

Scheme S1 S2

Scheme S2 S2

Fig. S1 S3

Fig. S2 S3

Spectra of Compounds S4

Cartesian coordinates and total energies for the calculated structures (B3LYP/6-31G** [SMD = water]) S40

Cartesian coordinates and total energies for all of the calculated structures (B3LYP/def2-TZVP//B3LYP/6-31G** [SMD = water]) S53

Second order perturbation theory analysis of the Fock matrices in the NBO analyses for the DFT calculated structures S103

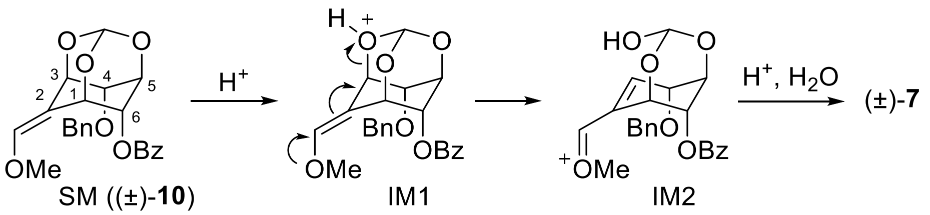

Scheme S1 First assumed mechanism of ring-opening reaction for (±)-**10**

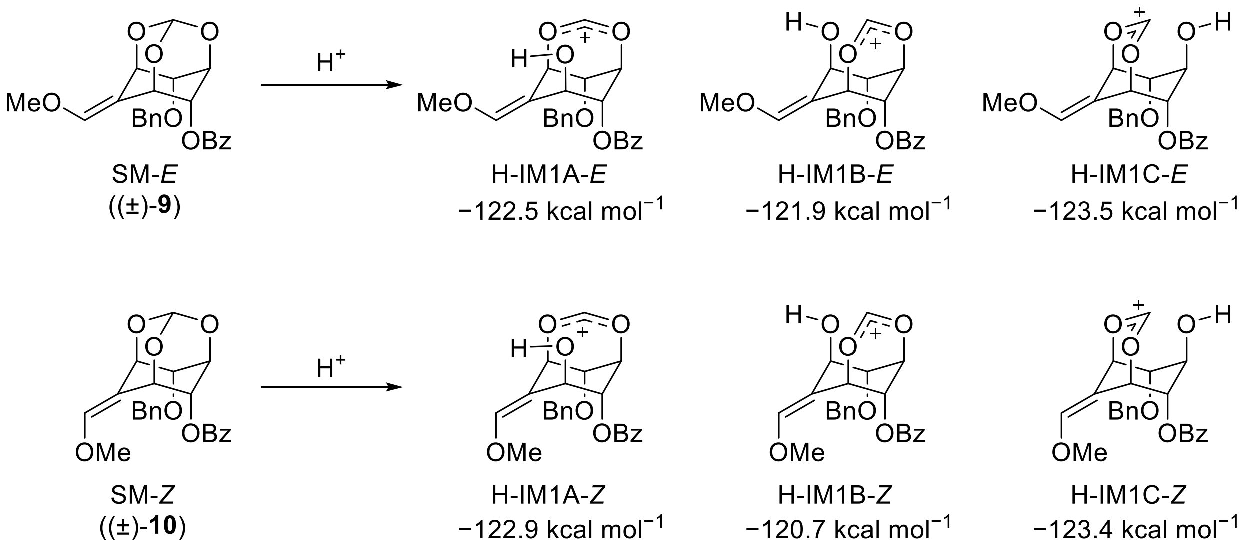

Scheme S2 Energy profile for-protonation of (±)-**9** and (±)-**10** calculated by DFT (B3LYP/6-31G** [SMD = water])

Structure of SM-*E* and SM-*Z* as starting materials were obtained from the optimized structure of **8** and **22** based on B3LYP/6-31G** [SMD = water].

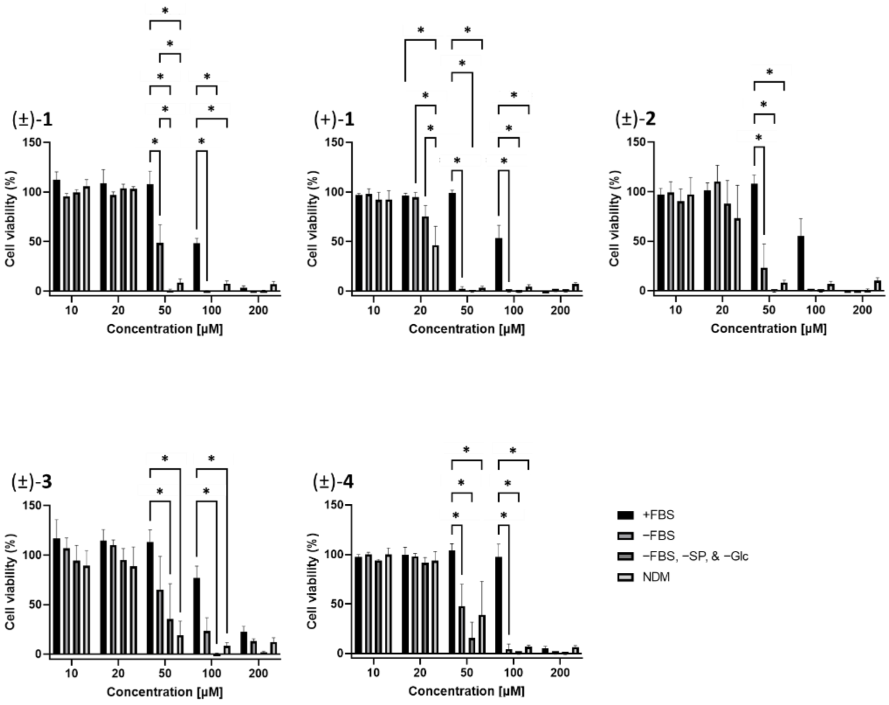

Fig. S1 Preferential cytotoxic activity of **1**–**4** toward PANC-1 cells in each medium determined by WST-8 Assay. +FBS: DMEM supplemented with 10% FBS, −FBS: FBS free DMEM, −FBS, −SP, and −Glc: FBS, sodium pyruvate, and glucose free DMEM, NDM: nutrient deprived medium. The results are expressed as the means ± standard error of the mean (*n*= 3–4). *P < 0.05.

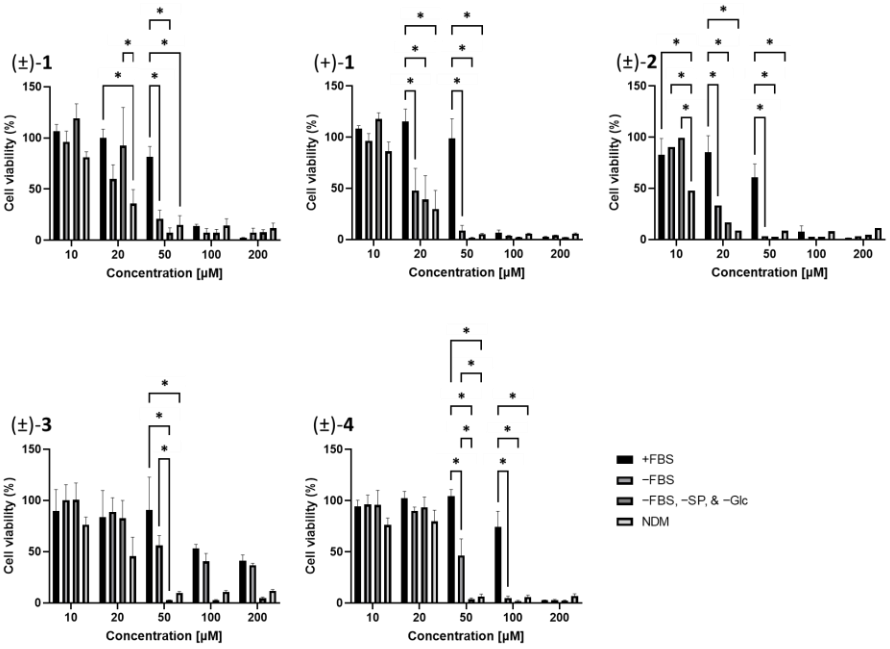

Fig. S2 Preferential cytotoxic activity of **1**–**4** toward HT-29 cells in each medium determined by WST-8 Assay. +FBS: RPMI-1640 supplemented with 10% FBS, −FBS: FBS free RPMI-1640, −FBS, −SP, and −Glc: FBS, sodium pyruvate, and glucose free RPMI-1640, NDM: nutrient deprived medium. The results are expressed as the means ± standard error of the mean (*n*= 3–6). *P < 0.05.

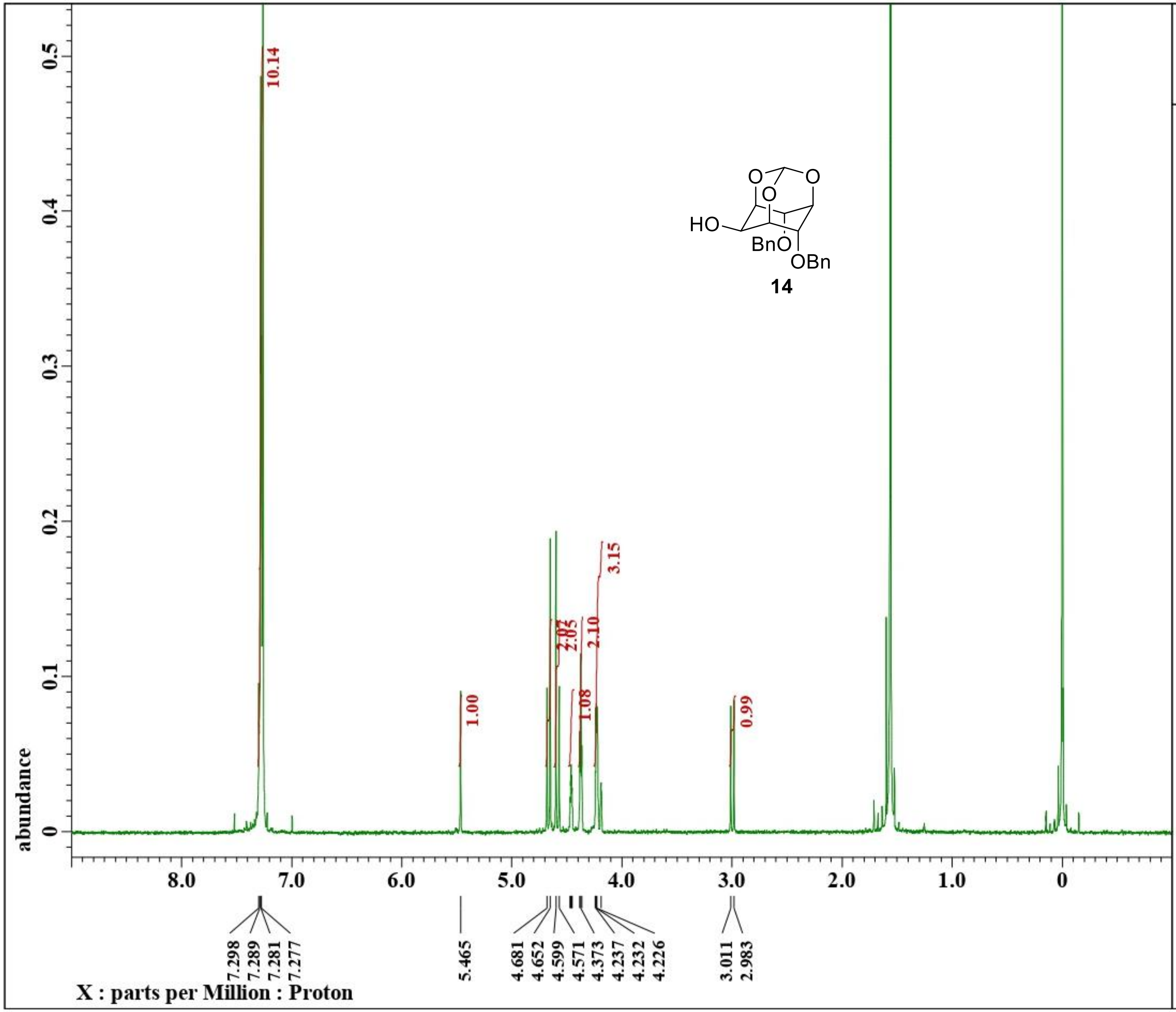

JEOL

Filename	= 18kus-diBn_Proton-
Author	= delta
Experiment	= proton_auto.jxp
Sample_Id	= 24RHT1-4
Solvent	= CHLOROFORM-D
Actual_Start_Time	= 29-AUG-2024 17:01:
Revision_Time	= 26-SEP-2024 23:00:
Comment	= single_pulse
Data_Format	= 1D COMPLEX
Dim_Size	= 13107
X_Domain	= Proton
Dim_Title	= Proton
Dim_Units	= [ppm]
Dimensions	= X
Spectrometer	= JNM-ECZ400S/L1
Field_Strength	= 9.389766[T] (400[M
X_Acq_Duration	= 2.18628096[s]
X_Domain	= Proton
X_Freq	= 399.78219838[MHz]
X_Offset	= 5[ppm]
X_Points	= 16384
X_Prescans	= 1
X_Resolution	= 0.45739775[Hz]
X_Sweep	= 7.4940048[kHz]
X_Sweep_Clipped	= 5.99520384[kHz]
Irr_Domain	= Proton
Irr_Freq	= 399.78219838[MHz]
Irr_Offset	= 5[ppm]
Tri_Domain	= Proton
Tri_Freq	= 399.78219838[MHz]
Tri_Offset	= 5[ppm]
Blanking	= 2.0[us]
Clipped	= FALSE
Scans	= 8
Total_Scans	= 8
Relaxation_Delay	= 5[s]
Recvr_Gain	= 66
Temp_Get	= 24.4[dC]
X_90_Width	= 6.6[us]
X_Acq_Time	= 2.18628096[s]
X_Angle	= 45[deg]
X_Atn	= 6.5[dB]
X_Pulse	= 3.3[us]
Irr_Mode	= Off
Tri_Mode	= Off
Dante_Loop	= 500
Dante_Presat	= FALSE
Decimation_Rate	= 0
Experiment_Path	= C:\Program Files\J
Initial_Wait	= 1[s]

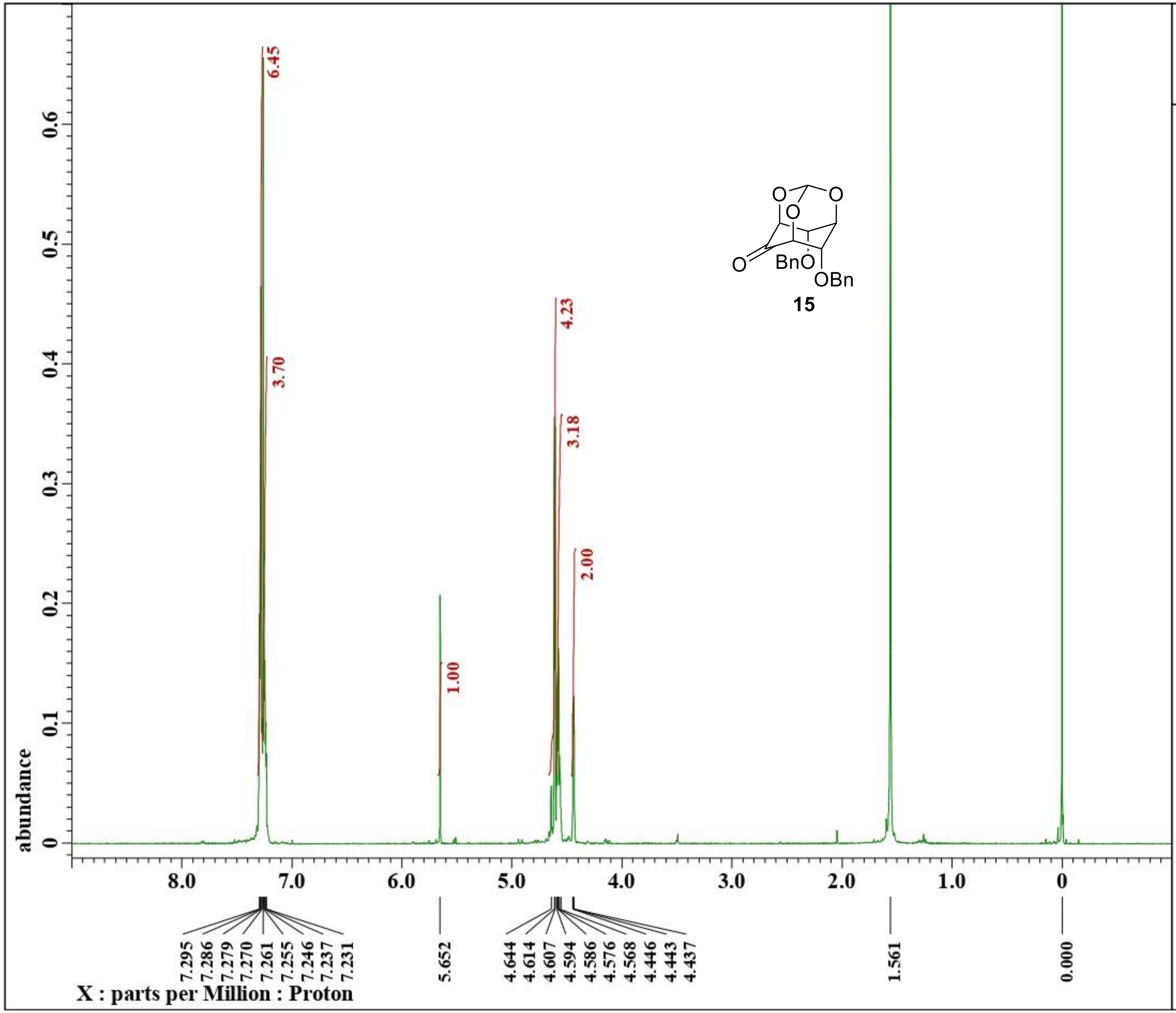

Filename	= 18kus17-38-1_Proto
Author	= delta
Experiment	= proton_auto.jxp
Sample_Id	= 18kus17-38-1
Solvent	= CHLOROFORM-D
Actual_Start_Time	= 25-SEP-2024 21:19:
Revision_Time	= 2-OCT-2024 19:16:
Comment	= single_pulse
Data_Format	= 1D COMPLEX
Dim_Size	= 13107
X_Domain	= Proton
Dim_Title	= Proton
Dim_Units	= [ppm]
Dimensions	= X
Spectrometer	= JNM-ECZ400S/L1
Field_Strength	= 9.389766[T] (400[M
X_Acq_Duration	= 2.18628096[s]
X_Domain	= Proton
X_Freq	= 399.78219838[MHz]
X_Offset	= 5[ppm]
X_Points	= 16384
X_Prescans	= 1
X_Resolution	= 0.45739775[Hz]
X_Sweep	= 7.4940048[kHz]
X_Sweep_Clipped	= 5.99520384[kHz]
Irr_Domain	= Proton
Irr_Freq	= 399.78219838[MHz]
Irr_Offset	= 5[ppm]
Tri_Domain	= Proton
Tri_Freq	= 399.78219838[MHz]
Tri_Offset	= 5[ppm]
Blanking	= 2.0[us]
Clipped	= FALSE
Scans	= 8
Total_Scans	= 8
Relaxation_Delay	= 5[s]
Recvr_Gain	= 56
Temp_Get	= 24.9[dC]
X_90_Width	= 6.6[us]
X_Acq_Time	= 2.18628096[s]
X_Angle	= 45[deg]
X_Atn	= 6.5[dB]
X_Pulse	= 3.3[us]
Irr_Mode	= Off
Tri_Mode	= Off
Dante_Loop	= 500
Dante_Presat	= FALSE
Decimation_Rate	= 0
Experiment_Path	= C:\Program Files\J
Initial_Wait	= 1[s]

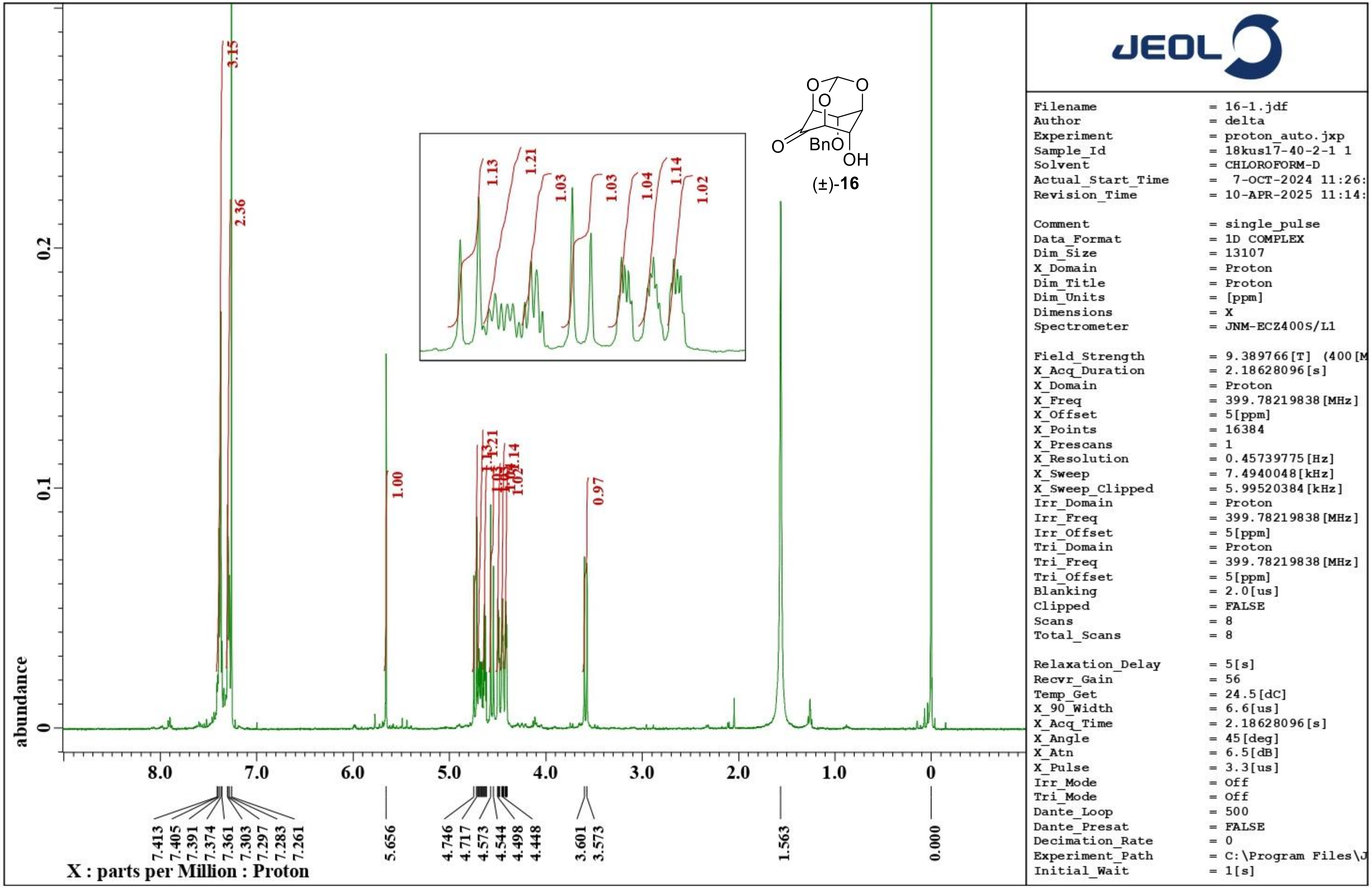

JEOL
(±)-16
BnO
OH
O
abundance
0.2
0.1
0
X : parts per Million : Proton
8.0
7.0
6.0
5.0
4.0
3.0
2.0
1.0
0
3.15
2.36
1.00
0.97
1.13
1.21
1.03
1.03
1.04
1.14
1.02
7.413
7.405
7.391
7.374
7.361
7.303
7.297
7.283
7.261
5.656
4.746
4.717
4.573
4.544
4.498
4.448
3.601
3.573
1.563
0.000
Filename = 16-1.jdf
Author = delta
Experiment = proton_auto.jxp
Sample_Id = 18kus17-40-2-1 1
Solvent = CHLOROFORM-D
Actual_Start_Time = 7-OCT-2024 11:26:
Revision_Time = 10-APR-2025 11:14:
Comment = single_pulse
Data_Format = 1D COMPLEX
Dim_Size = 13107
X_Domain = Proton
Dim_Title = Proton
Dim_Units = [ppm]
Dimensions = X
Spectrometer = JNM-ECZ400S/L1
Field_Strength = 9.389766[T] (400[M
X_Acq_Duration = 2.18628096[s]
X_Domain = Proton
X_Freq = 399.78219838[MHz]
X_Offset = 5[ppm]
X_Points = 16384
X_Prescans = 1
X_Resolution = 0.45739775[Hz]
X_Sweep = 7.4940048[kHz]
X_Sweep_Clipped = 5.99520384[kHz]
Irr_Domain = Proton
Irr_Freq = 399.78219838[MHz]
Irr_Offset = 5[ppm]
Tri_Domain = Proton
Tri_Freq = 399.78219838[MHz]
Tri_Offset = 5[ppm]
Blanking = 2.0[us]
Clipped = FALSE
Scans = 8
Total_Scans = 8
Relaxation_Delay = 5[s]
Recvr_Gain = 56
Temp_Get = 24.5[dC]
X_90_Width = 6.6[us]
X_Acq_Time = 2.18628096[s]
X_Angle = 45[deg]
X_Atn = 6.5[dB]
X_Pulse = 3.3[us]
Irr_Mode = Off
Tri_Mode = Off
Dante_Loop = 500
Dante_Presat = FALSE
Decimation_Rate = 0
Experiment_Path = C:\Program Files\J
Initial_Wait = 1[s]

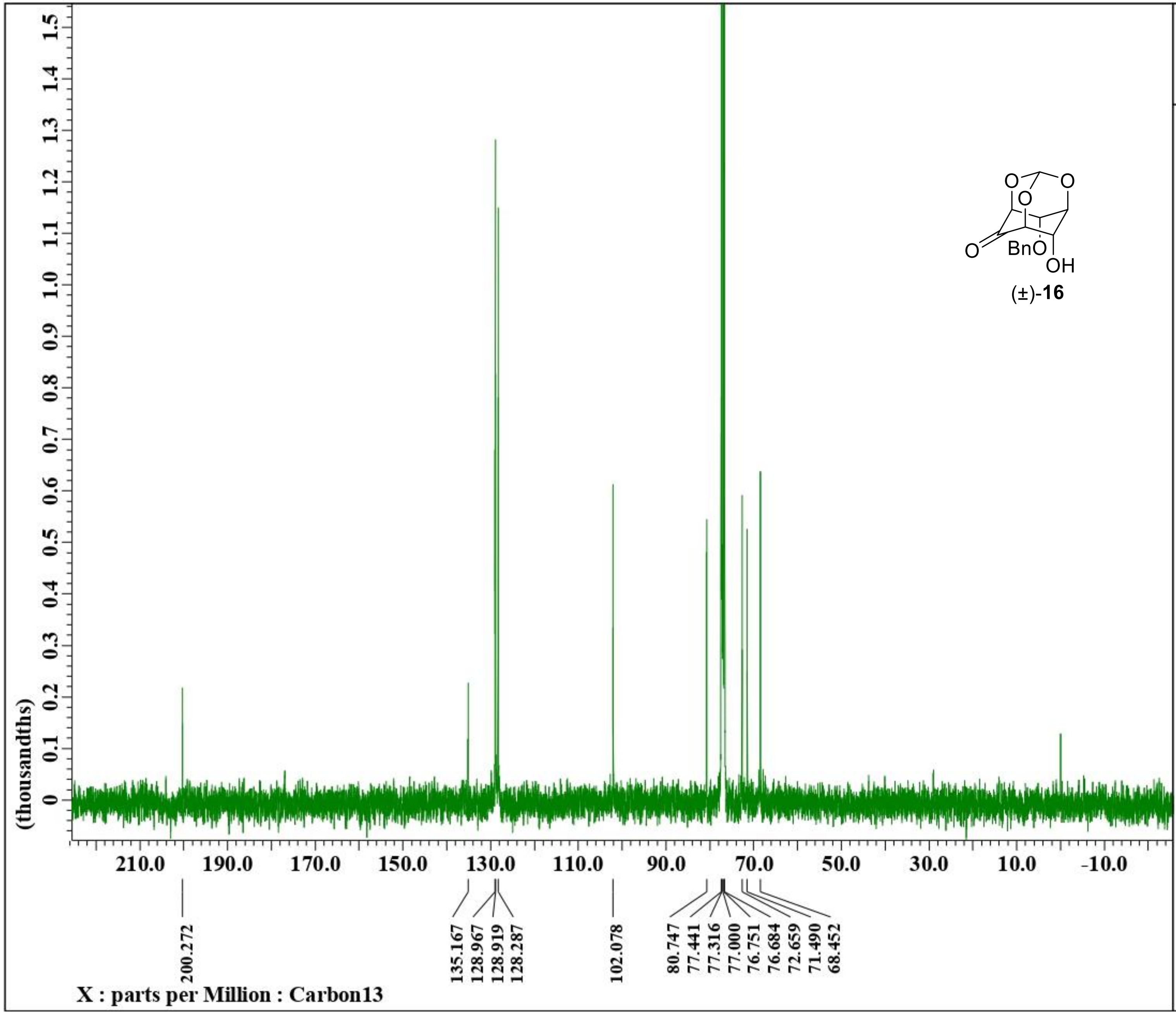

Filename	= 18kus17-40-2-1_
Author	= delta
Experiment	= carbon_auto.jxp
Sample_Id	= 18kus17-40-2-1
Solvent	= CHLOROFORM-D
Actual_Start_Time	= 4-OCT-2024 21:
Revision_Time	= 8-OCT-2024 00:
Comment	= single pulse de
Data_Format	= 1D COMPLEX
Dim_Size	= 26214
X_Domain	= Carbon13
Dim_Title	= Carbon13
Dim_Units	= [ppm]
Dimensions	= X
Spectrometer	= JNM-ECZ400S/L1
Field_Strength	= 9.389766[T] (40
X_Acq_Duration	= 1.03809024[s]
X_Domain	= Carbon13
X_Freq	= 100.52530333[MH
X_Offset	= 100[ppm]
X_Points	= 32768
X_Prescans	= 4
X_Resolution	= 0.96330739[Hz]
X_Sweep	= 31.56565657[kHz
X_Sweep_Clipped	= 25.25252525[kHz
Irr_Domain	= Proton
Irr_Freq	= 399.78219838[MH
Irr_Offset	= 5[ppm]
Blanking	= 5.0[us]
Clipped	= FALSE
Scans	= 2048
Total_Scans	= 2048
Relaxation_Delay	= 2[s]
Recvr_Gain	= 50
Temp_Get	= 24.3[dC]
X_90_Width	= 10.5[us]
X_Acq_Time	= 1.03809024[s]
X_Angle	= 30[deg]
X_Atn	= 8.2[dB]
X_Pulse	= 3.5[us]
Irr_Atn_Dec	= 31.323[dB]
Irr_Atn_Dec_Calc	= 31.323[dB]
Irr_Atn_Dec_Default_Calc	= 31.323[dB]
Irr_Atn_Noe	= 31.323[dB]
Irr_Dec_Bandwidth_Hz	= 4.7826087[kHz]
Irr_Dec_Bandwidth_Ppm	= 11.96303566[ppm
Irr_Dec_Freq	= 399.78219838[MH
Irr_Dec_Merit_Factor	= 2.2
Irr_Decoupling	= TRUE
Irr_Noe	= TRUE

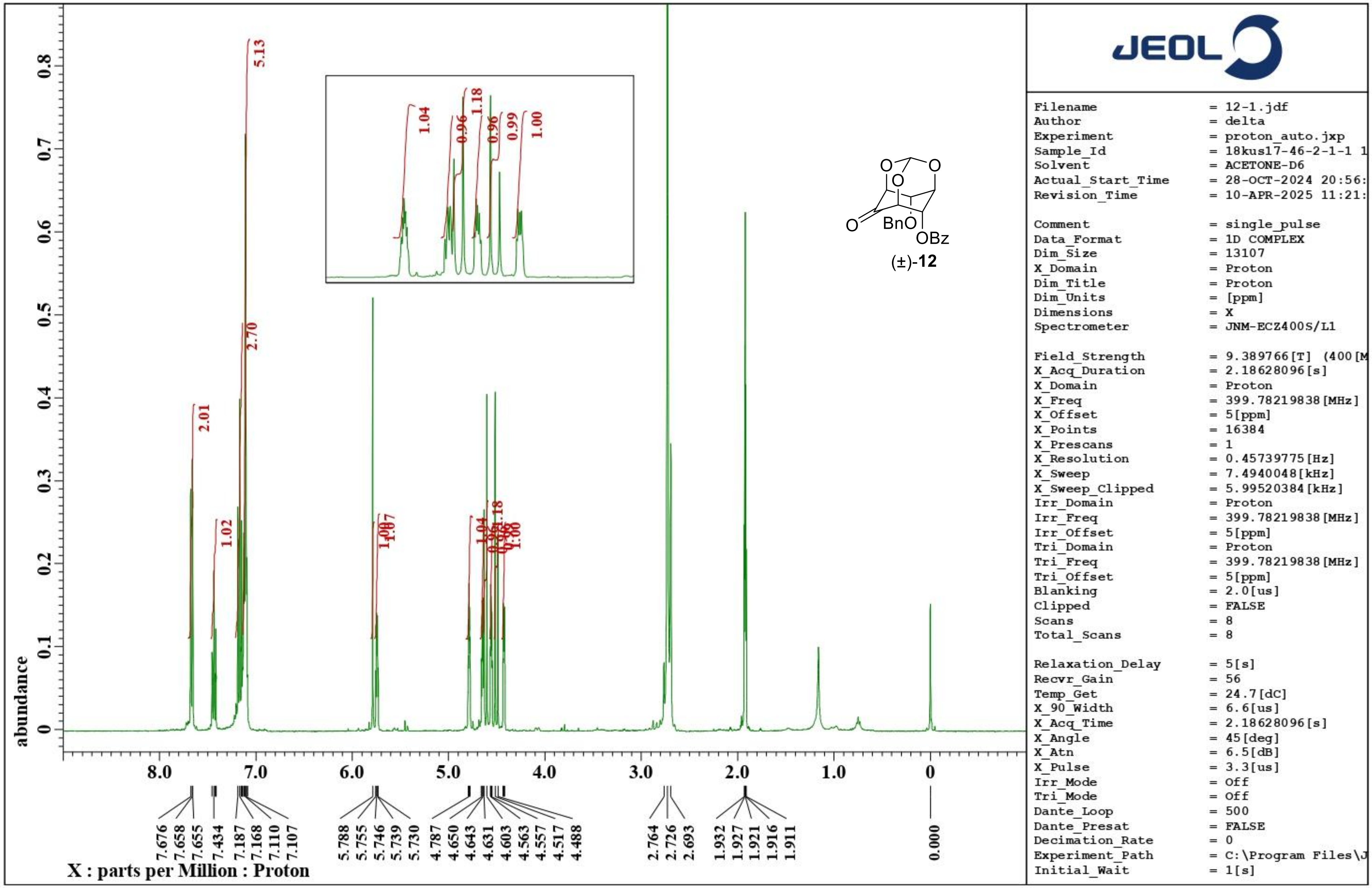

Filename	= 12-1.jdf
Author	= delta
Experiment	= proton_auto.jxp
Sample_Id	= 18kus17-46-2-1-1 1
Solvent	= ACETONE-D6
Actual_Start_Time	= 28-OCT-2024 20:56:
Revision_Time	= 10-APR-2025 11:21:
Comment	= single_pulse
Data_Format	= 1D COMPLEX
Dim_Size	= 13107
X_Domain	= Proton
Dim_Title	= Proton
Dim_Units	= [ppm]
Dimensions	= X
Spectrometer	= JNM-ECZ400S/L1
Field_Strength	= 9.389766[T] (400[M
X_Acq_Duration	= 2.18628096[s]
X_Domain	= Proton
X_Freq	= 399.78219838[MHz]
X_Offset	= 5[ppm]
X_Points	= 16384
X_Prescans	= 1
X_Resolution	= 0.45739775[Hz]
X_Sweep	= 7.4940048[kHz]
X_Sweep_Clipped	= 5.99520384[kHz]
Irr_Domain	= Proton
Irr_Freq	= 399.78219838[MHz]
Irr_Offset	= 5[ppm]
Tri_Domain	= Proton
Tri_Freq	= 399.78219838[MHz]
Tri_Offset	= 5[ppm]
Blanking	= 2.0[us]
Clipped	= FALSE
Scans	= 8
Total_Scans	= 8
Relaxation_Delay	= 5[s]
Recvr_Gain	= 56
Temp_Get	= 24.7[dC]
X_90_Width	= 6.6[us]
X_Acq_Time	= 2.18628096[s]
X_Angle	= 45[deg]
X_Atn	= 6.5[dB]
X_Pulse	= 3.3[us]
Irr_Mode	= Off
Tri_Mode	= Off
Dante_Loop	= 500
Dante_Presat	= FALSE
Decimation_Rate	= 0
Experiment_Path	= C:\Program Files\J
Initial_Wait	= 1[s]

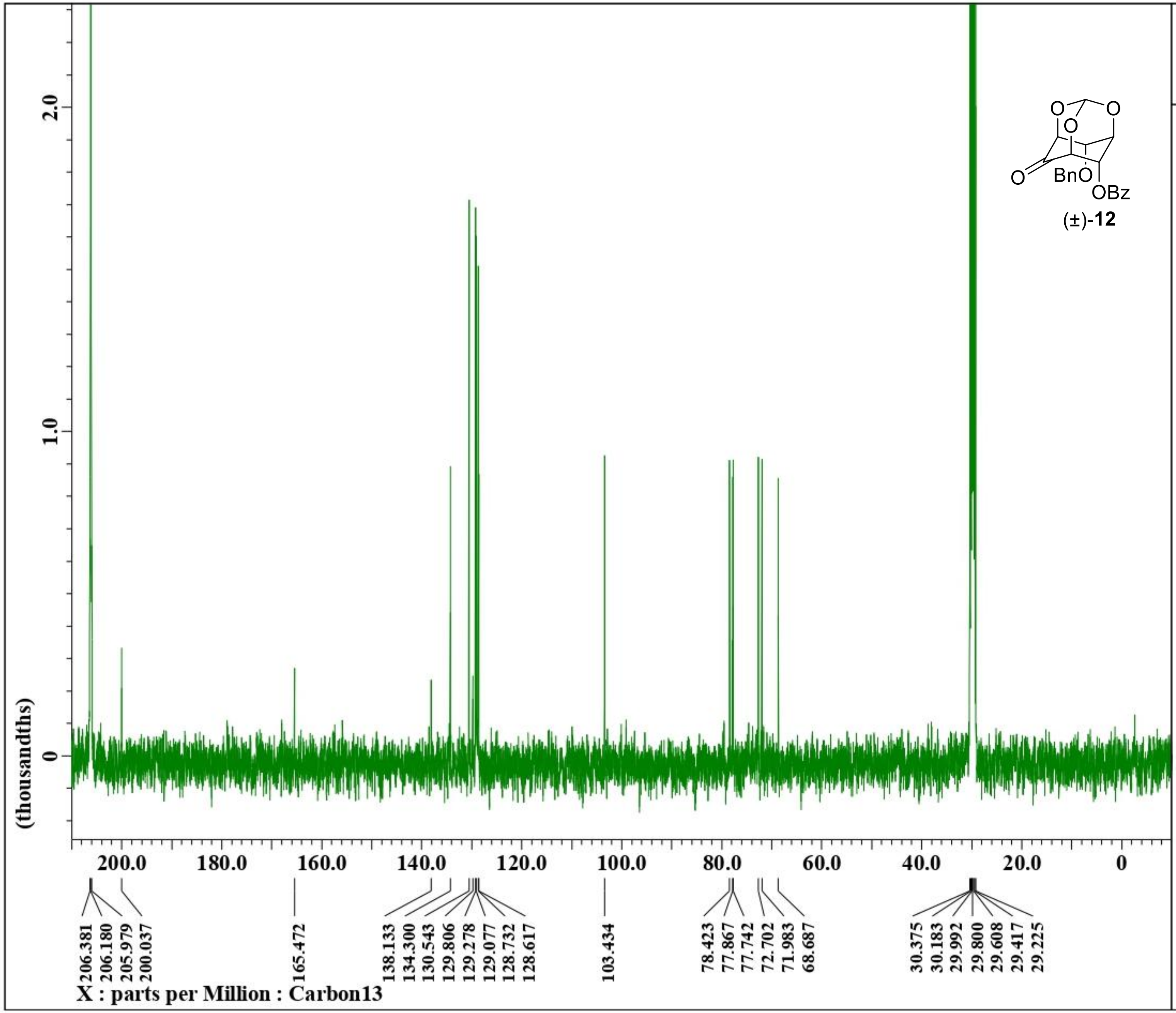

Filename = 18kus17-46-2-1-
Author = delta
Experiment = carbon_auto.jxp
Sample_Id = 18kus17-46-2-1-
Solvent = ACETONE-D6
Actual_Start_Time = 28-OCT-2024 20:
Revision_Time = 28-OCT-2024 21:

Comment = single pulse de
Data_Format = 1D COMPLEX
Dim_Size = 26214
X_Domain = Carbon13
Dim_Title = Carbon13
Dim_Units = [ppm]
Dimensions = X
Spectrometer = JNM-ECZ400S/L1

Field_Strength = 9.389766[T] (40
X_Acq_Duration = 1.03809024[s]
X_Domain = Carbon13
X_Freq = 100.52530333[MH
X_Offset = 100[ppm]
X_Points = 32768
X_Prescans = 4
X_Resolution = 0.96330739[Hz]
X_Sweep = 31.56565657[kHz
X_Sweep_Clipped = 25.25252525[kHz
Irr_Domain = Proton
Irr_Freq = 399.78219838[MH
Irr_Offset = 5[ppm]
Blanking = 5.0[us]
Clipped = FALSE
Incomplete_Copy = TRUE
Scans = 390
Total_Scans = 390

Relaxation_Delay = 2[s]
Recvr_Gain = 50
Temp_Get = 24.4[dC]
X_90_Width = 10.5[us]
X_Acq_Time = 1.03809024[s]
X_Angle = 30[deg]
X_Atn = 8.2[dB]
X_Pulse = 3.5[us]
Irr_Atn_Dec = 31.323[dB]
Irr_Atn_Dec_Calc = 31.323[dB]
Irr_Atn_Dec_Default_Calc = 31.323[dB]
Irr_Atn_Noe = 31.323[dB]
Irr_Dec_Bandwidth_Hz = 4.7826087[kHz]
Irr_Dec_Bandwidth_Ppm = 11.96303566[ppm
Irr_Dec_Freq = 399.78219838[MH
Irr_Dec_Merit_Factor = 2.2
Irr_Decoupling = TRUE

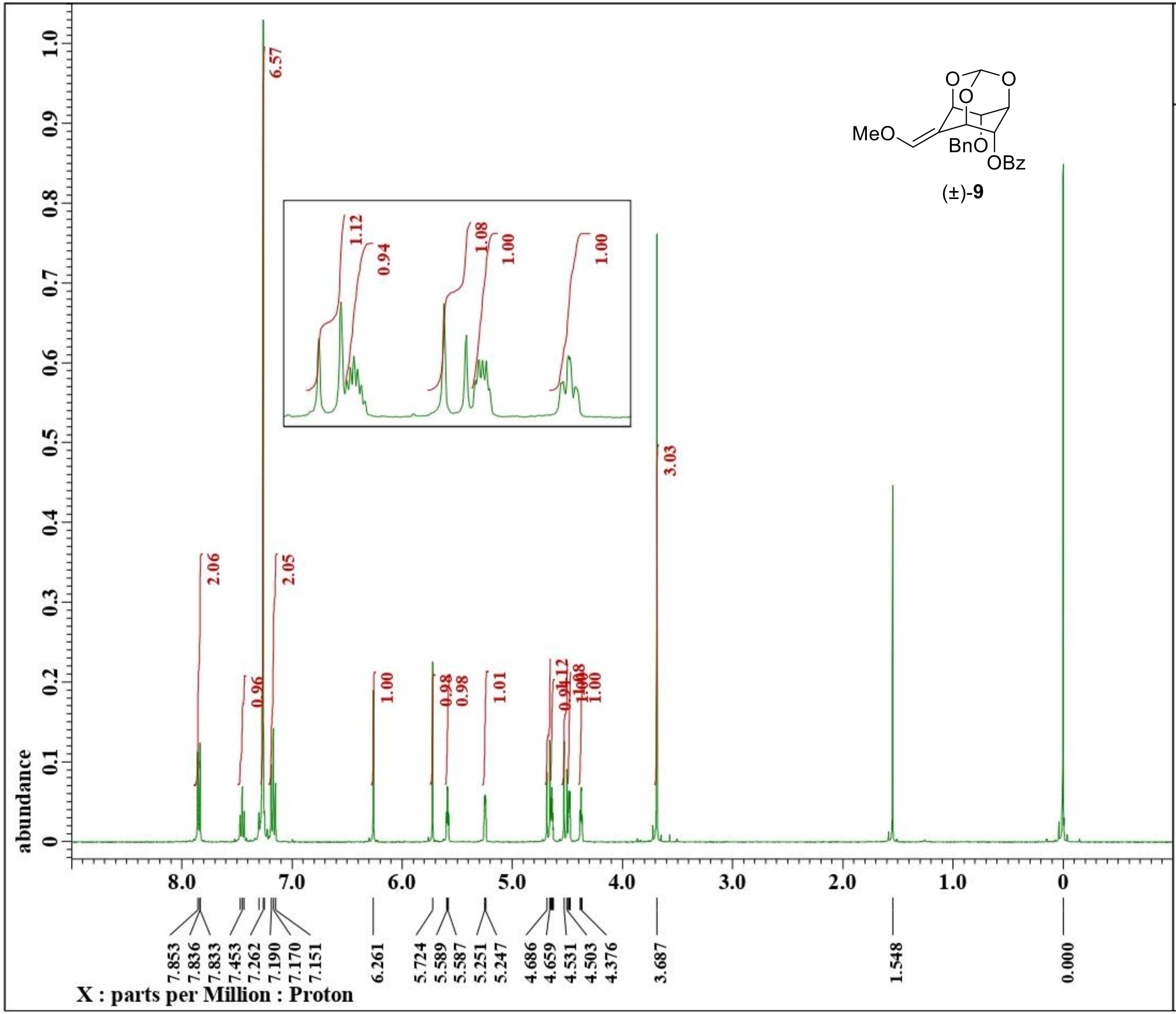

JEOL

Filename	= 9 (2)-1.jdf
Author	= delta
Experiment	= proton_auto.jxp
Sample_Id	= 18kus9-92-1
Solvent	= CHLOROFORM-D
Actual_Start_Time	= 28-OCT-2022 12:01:
Revision_Time	= 10-APR-2025 11:28:
Comment	= single_pulse
Data_Format	= 1D COMPLEX
Dim_Size	= 13107
X_Domain	= Proton
Dim_Title	= Proton
Dim_Units	= [ppm]
Dimensions	= X
Spectrometer	= JNM-ECZ400S/L1
Field_Strength	= 9.389766[T] (400[M
X_Acq_Duration	= 2.18628096[s]
X_Domain	= Proton
X_Freq	= 399.78219838[MHz]
X_Offset	= 5[ppm]
X_Points	= 16384
X_Prescans	= 1
X_Resolution	= 0.45739775[Hz]
X_Sweep	= 7.4940048[kHz]
X_Sweep_Clipped	= 5.99520384[kHz]
Irr_Domain	= Proton
Irr_Freq	= 399.78219838[MHz]
Irr_Offset	= 5[ppm]
Tri_Domain	= Proton
Tri_Freq	= 399.78219838[MHz]
Tri_Offset	= 5[ppm]
Blanking	= 2.0[us]
Clipped	= FALSE
Scans	= 8
Total_Scans	= 8
Relaxation_Delay	= 5[s]
Recvr_Gain	= 56
Temp_Get	= 22.6[dC]
X_90_Width	= 6.6[us]
X_Acq_Time	= 2.18628096[s]
X_Angle	= 45[deg]
X_Atn	= 6.5[dB]
X_Pulse	= 3.3[us]
Irr_Mode	= Off
Tri_Mode	= Off
Dante_Loop	= 500
Dante_Presat	= FALSE
Decimation_Rate	= 0
Experiment_Path	= C:\Program Files\J
Initial_Wait	= 1[s]

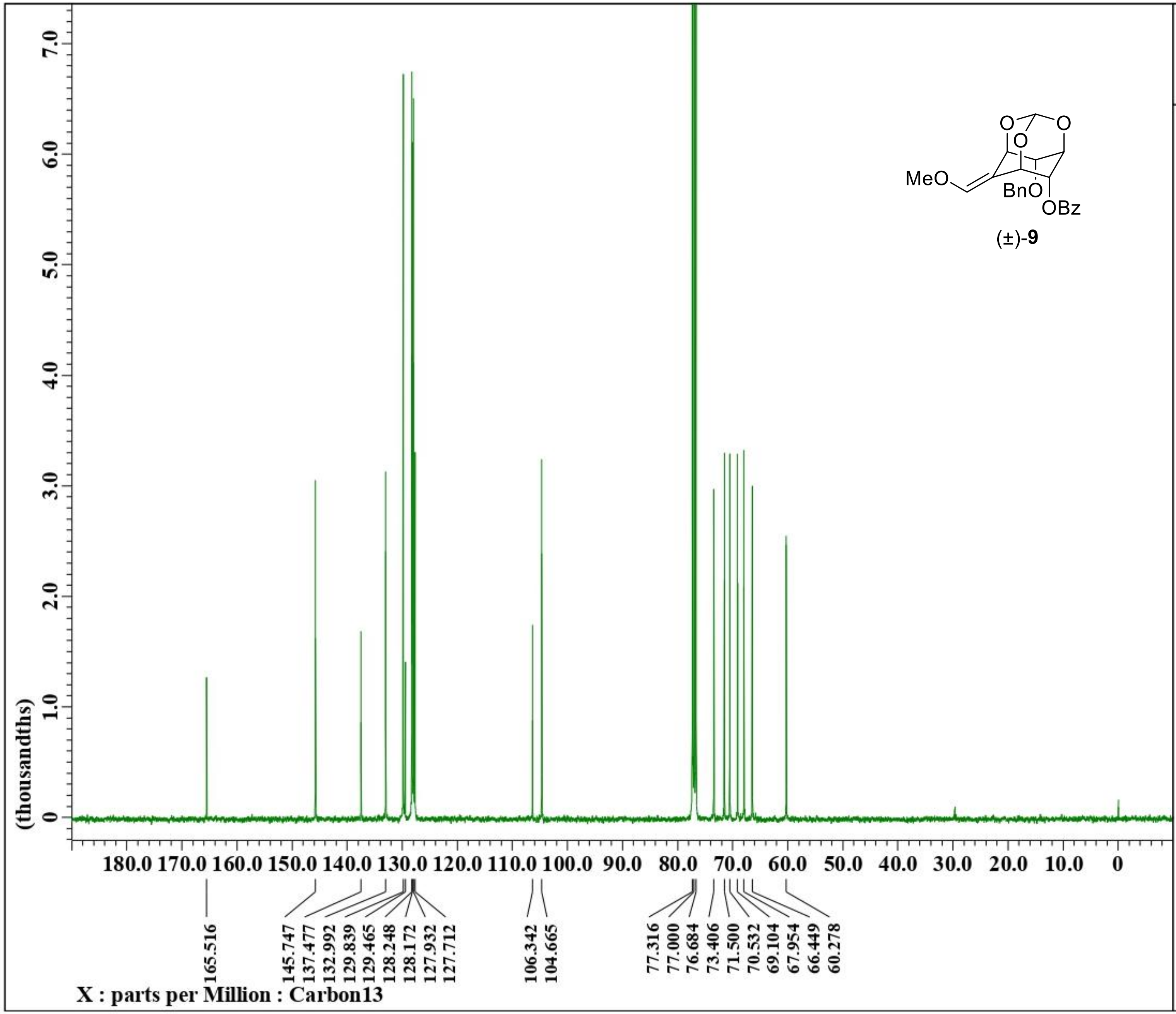

(±)-**9**

JEOL

Filename	= 18kus9-92-1-1p_
Author	= delta
Experiment	= carbon_auto.jxp
Sample_Id	= 18kus9-92-1-1p
Solvent	= CHLOROFORM-D
Actual_Start_Time	= 16-SEP-2022 21:
Revision_Time	= 2-OCT-2024 21:
Comment	= single pulse de
Data_Format	= 1D COMPLEX
Dim_Size	= 26214
X_Domain	= Carbon13
Dim_Title	= Carbon13
Dim_Units	= [ppm]
Dimensions	= X
Spectrometer	= JNM-ECZ400S/L1
Field_Strength	= 9.389766[T] (40
X_Acq_Duration	= 1.03809024[s]
X_Domain	= Carbon13
X_Freq	= 100.52530333[MH
X_Offset	= 100[ppm]
X_Points	= 32768
X_Prescans	= 4
X_Resolution	= 0.96330739[Hz]
X_Sweep	= 31.56565657[kHz
X_Sweep_Clipped	= 25.25252525[kHz
Irr_Domain	= Proton
Irr_Freq	= 399.78219838[MH
Irr_Offset	= 5[ppm]
Blanking	= 5.0[us]
Clipped	= FALSE
Scans	= 4000
Total_Scans	= 4000
Relaxation_Delay	= 2[s]
Recvr_Gain	= 50
Temp_Get	= 22.2[dC]
X_90_Width	= 10.5[us]
X_Acq_Time	= 1.03809024[s]
X_Angle	= 30[deg]
X_Atn	= 8.2[dB]
X_Pulse	= 3.5[us]
Irr_Atn_Dec	= 31.323[dB]
Irr_Atn_Dec_Calc	= 31.323[dB]
Irr_Atn_Dec_Default_Calc	= 31.323[dB]
Irr_Atn_Noe	= 31.323[dB]
Irr_Dec_Bandwidth_Hz	= 4.7826087[kHz]
Irr_Dec_Bandwidth_Ppm	= 11.96303566[ppm
Irr_Dec_Freq	= 399.78219838[MH
Irr_Dec_Merit_Factor	= 2.2
Irr_Decoupling	= TRUE
Irr_Noe	= TRUE

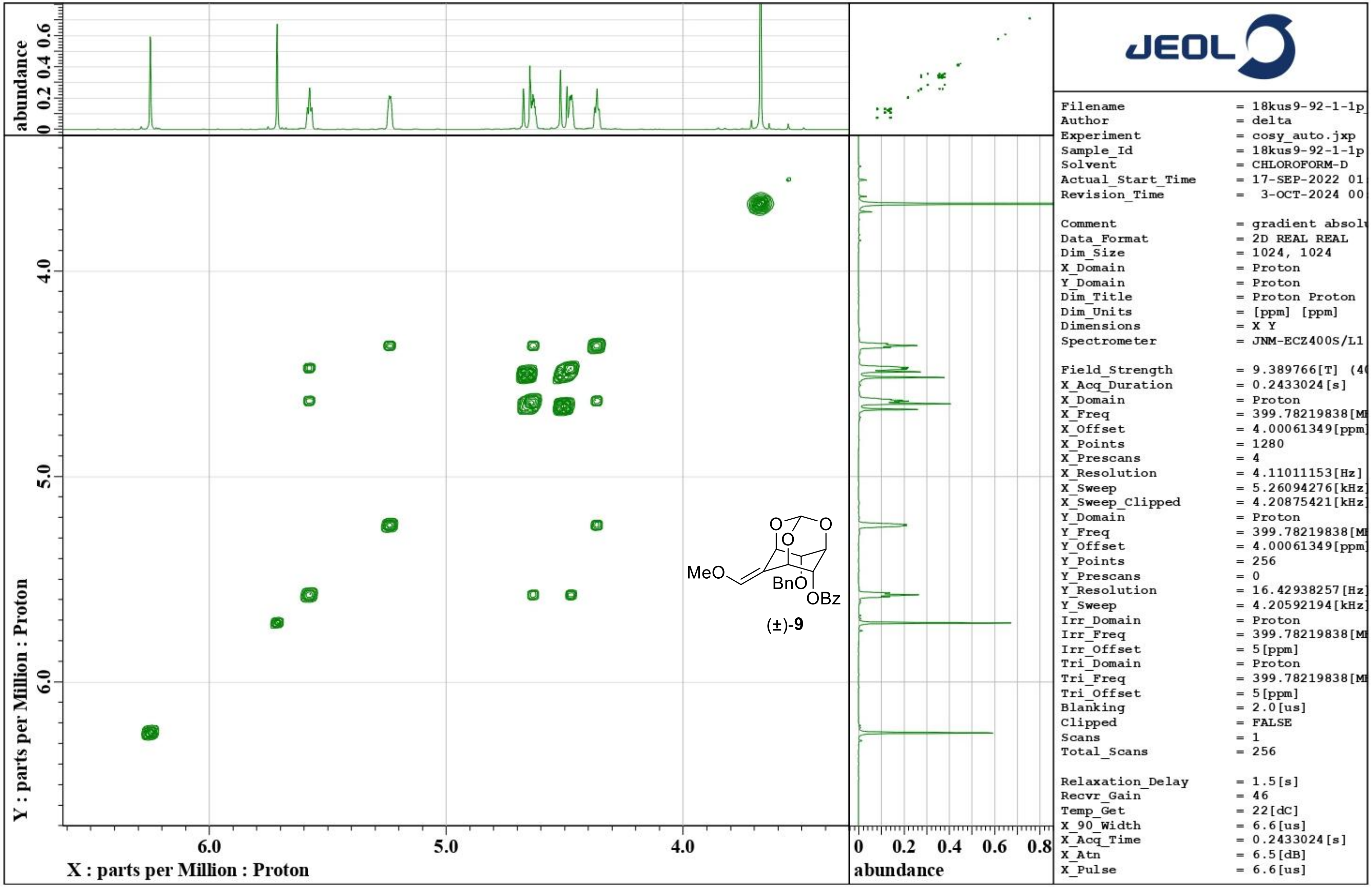
abundance
0 0.2 0.4 0.6
Y : parts per Million : Proton
4.0
5.0
6.0
X : parts per Million : Proton
6.0
5.0
4.0
0 0.2 0.4 0.6 0.8
abundance
MeO
O
O
O
BnO
OBz
(±)-9
Filename = 18kus9-92-1-1p_
Author = delta
Experiment = cosy_auto.jxp
Sample_Id = 18kus9-92-1-1p
Solvent = CHLOROFORM-D
Actual_Start_Time = 17-SEP-2022 01:
Revision_Time = 3-OCT-2024 00:
Comment = gradient absolu
Data_Format = 2D REAL REAL
Dim_Size = 1024, 1024
X_Domain = Proton
Y_Domain = Proton
Dim_Title = Proton Proton
Dim_Units = [ppm] [ppm]
Dimensions = X Y
Spectrometer = JNM-ECZ400S/L1
Field_Strength = 9.389766[T] (40
X_Acq_Duration = 0.2433024[s]
X_Domain = Proton
X_Freq = 399.78219838[MH
X_Offset = 4.00061349[ppm]
X_Points = 1280
X_Prescans = 4
X_Resolution = 4.11011153[Hz]
X_Sweep = 5.26094276[kHz]
X_Sweep_Clipped = 4.20875421[kHz]
Y_Domain = Proton
Y_Freq = 399.78219838[MH
Y_Offset = 4.00061349[ppm]
Y_Points = 256
Y_Prescans = 0
Y_Resolution = 16.42938257[Hz]
Y_Sweep = 4.20592194[kHz]
Irr_Domain = Proton
Irr_Freq = 399.78219838[MH
Irr_Offset = 5[ppm]
Tri_Domain = Proton
Tri_Freq = 399.78219838[MH
Tri_Offset = 5[ppm]
Blanking = 2.0[us]
Clipped = FALSE
Scans = 1
Total_Scans = 256
Relaxation_Delay = 1.5[s]
Recvr_Gain = 46
Temp_Get = 22[dC]
X_90_Width = 6.6[us]
X_Acq_Time = 0.2433024[s]
X_Atn = 6.5[dB]
X_Pulse = 6.6[us]

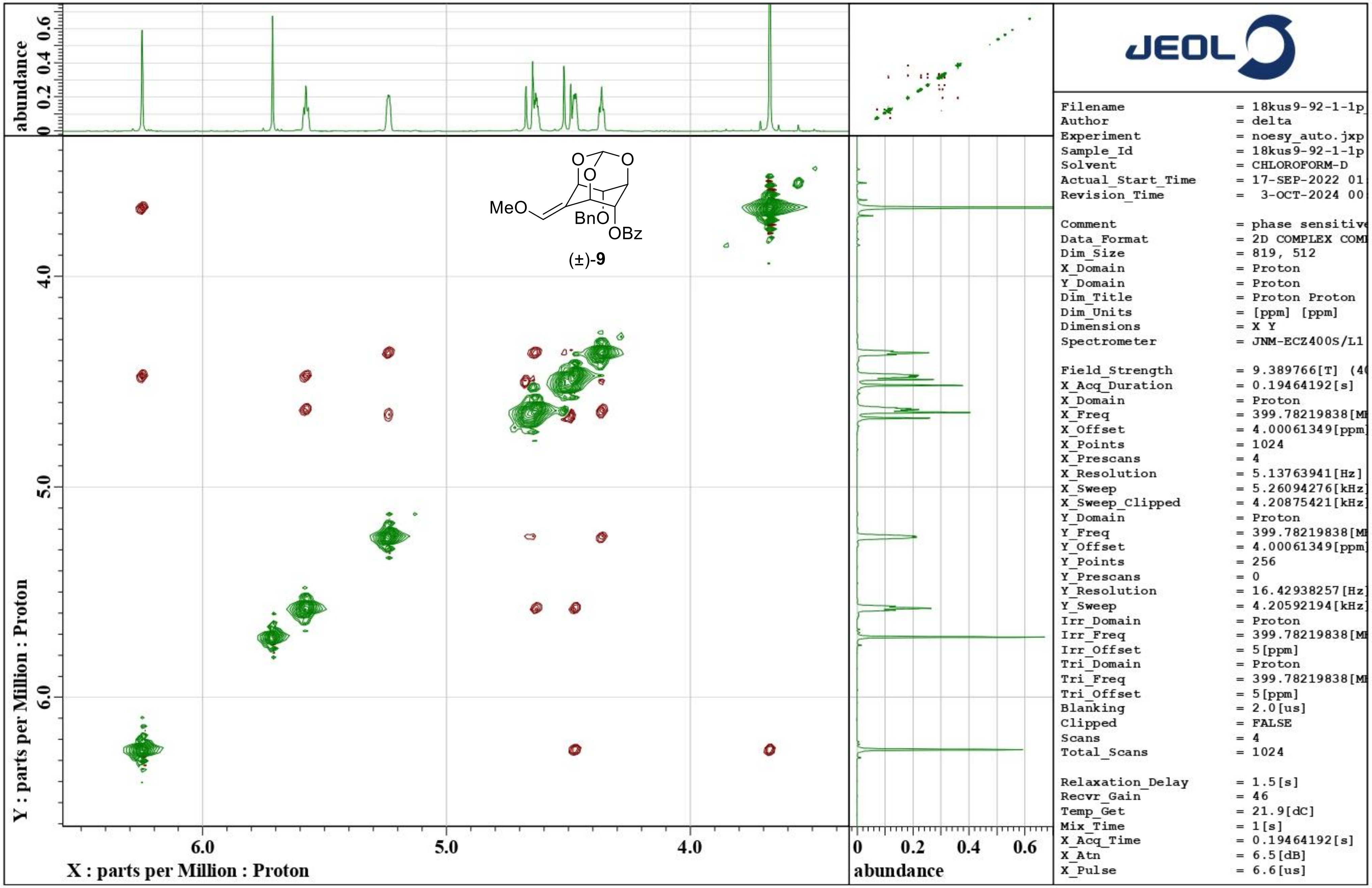

Filename	= 18kus9-92-1-1p_
Author	= delta
Experiment	= noesy_auto.jxp
Sample_Id	= 18kus9-92-1-1p
Solvent	= CHLOROFORM-D
Actual_Start_Time	= 17-SEP-2022 01
Revision_Time	= 3-OCT-2024 00
Comment	= phase sensitive
Data_Format	= 2D COMPLEX COMI
Dim_Size	= 819, 512
X_Domain	= Proton
Y_Domain	= Proton
Dim_Title	= Proton Proton
Dim_Units	= [ppm] [ppm]
Dimensions	= X Y
Spectrometer	= JNM-ECZ400S/L1
Field_Strength	= 9.389766[T] (4(
X_Acq_Duration	= 0.19464192[s]
X_Domain	= Proton
X_Freq	= 399.78219838[MI
X_Offset	= 4.00061349[ppm]
X_Points	= 1024
X_Prescans	= 4
X_Resolution	= 5.13763941[Hz]
X_Sweep	= 5.26094276[kHz]
X_Sweep_Clipped	= 4.20875421[kHz]
Y_Domain	= Proton
Y_Freq	= 399.78219838[MI
Y_Offset	= 4.00061349[ppm]
Y_Points	= 256
Y_Prescans	= 0
Y_Resolution	= 16.42938257[Hz]
Y_Sweep	= 4.20592194[kHz]
Irr_Domain	= Proton
Irr_Freq	= 399.78219838[MI
Irr_Offset	= 5[ppm]
Tri_Domain	= Proton
Tri_Freq	= 399.78219838[MI
Tri_Offset	= 5[ppm]
Blanking	= 2.0[us]
Clipped	= FALSE
Scans	= 4
Total_Scans	= 1024
Relaxation_Delay	= 1.5[s]
Recvr_Gain	= 46
Temp_Get	= 21.9[dC]
Mix_Time	= 1[s]
X_Acq_Time	= 0.19464192[s]
X_Atn	= 6.5[dB]
X_Pulse	= 6.6[us]

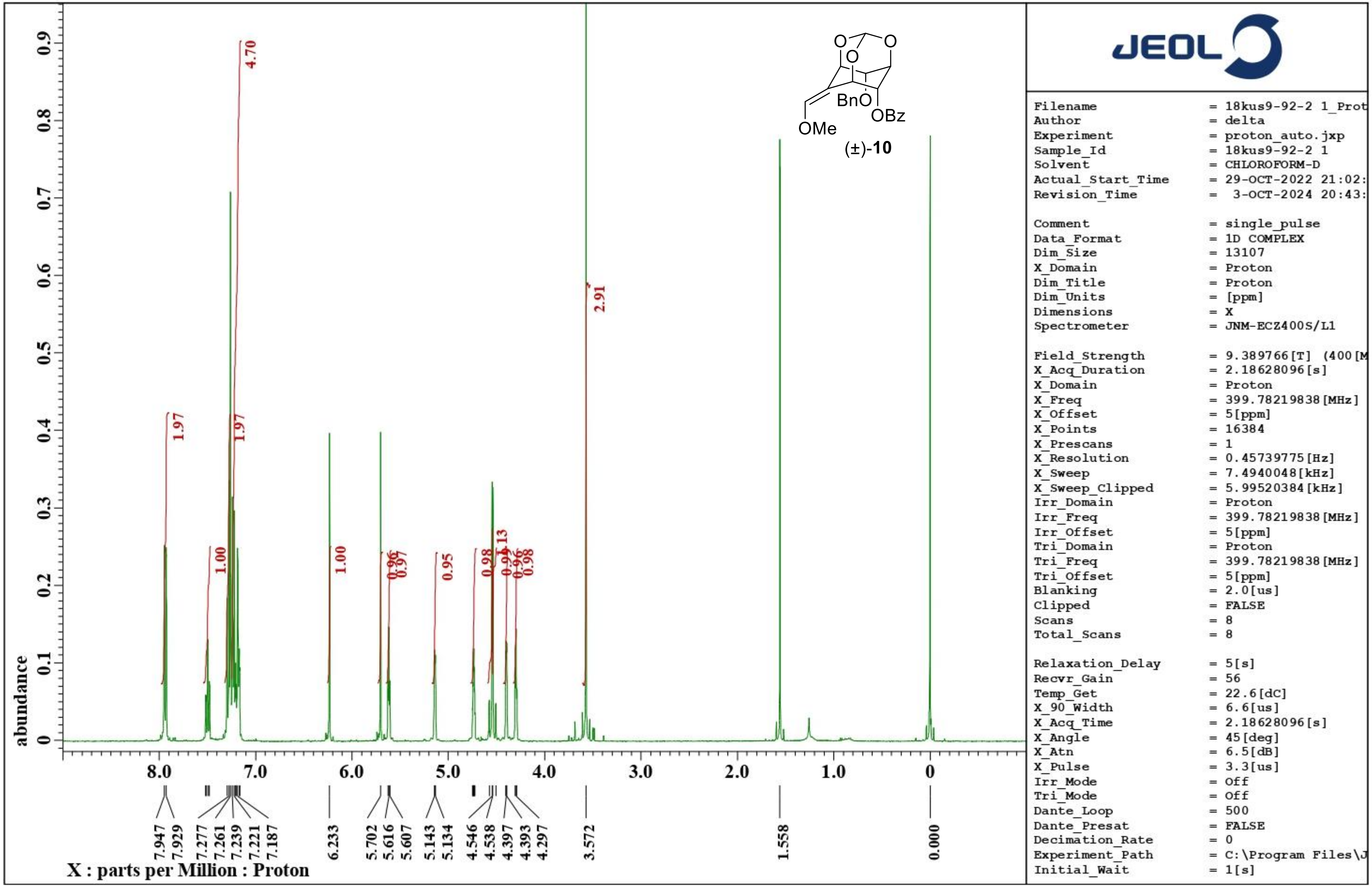

OMe
BnO
OBz
(±)-10
abundance
X : parts per Million : Proton
8.0
7.0
6.0
5.0
4.0
3.0
2.0
1.0
0
1.97
4.70
1.97
1.00
1.00
0.96
0.97
0.95
0.98
1.13
2.91
7.947
7.929
7.277
7.261
7.239
7.221
7.187
6.233
5.702
5.616
5.607
5.143
5.134
4.546
4.538
4.397
4.393
4.297
3.572
1.558
0.000
JEOL
Filename = 18kus9-92-2 1_Prot
Author = delta
Experiment = proton_auto.jxp
Sample_Id = 18kus9-92-2 1
Solvent = CHLOROFORM-D
Actual_Start_Time = 29-OCT-2022 21:02:
Revision_Time = 3-OCT-2024 20:43:
Comment = single_pulse
Data_Format = 1D COMPLEX
Dim_Size = 13107
X_Domain = Proton
Dim_Title = Proton
Dim_Units = [ppm]
Dimensions = X
Spectrometer = JNM-ECZ400S/L1
Field_Strength = 9.389766[T] (400[M
X_Acq_Duration = 2.18628096[s]
X_Domain = Proton
X_Freq = 399.78219838[MHz]
X_Offset = 5[ppm]
X_Points = 16384
X_Prescans = 1
X_Resolution = 0.45739775[Hz]
X_Sweep = 7.4940048[kHz]
X_Sweep_Clipped = 5.99520384[kHz]
Irr_Domain = Proton
Irr_Freq = 399.78219838[MHz]
Irr_Offset = 5[ppm]
Tri_Domain = Proton
Tri_Freq = 399.78219838[MHz]
Tri_Offset = 5[ppm]
Blanking = 2.0[us]
Clipped = FALSE
Scans = 8
Total_Scans = 8
Relaxation_Delay = 5[s]
Recvr_Gain = 56
Temp_Get = 22.6[dC]
X_90_Width = 6.6[us]
X_Acq_Time = 2.18628096[s]
X_Angle = 45[deg]
X_Atn = 6.5[dB]
X_Pulse = 3.3[us]
Irr_Mode = Off
Tri_Mode = Off
Dante_Loop = 500
Dante_Presat = FALSE
Decimation_Rate = 0
Experiment_Path = C:\Program Files\J
Initial_Wait = 1[s]

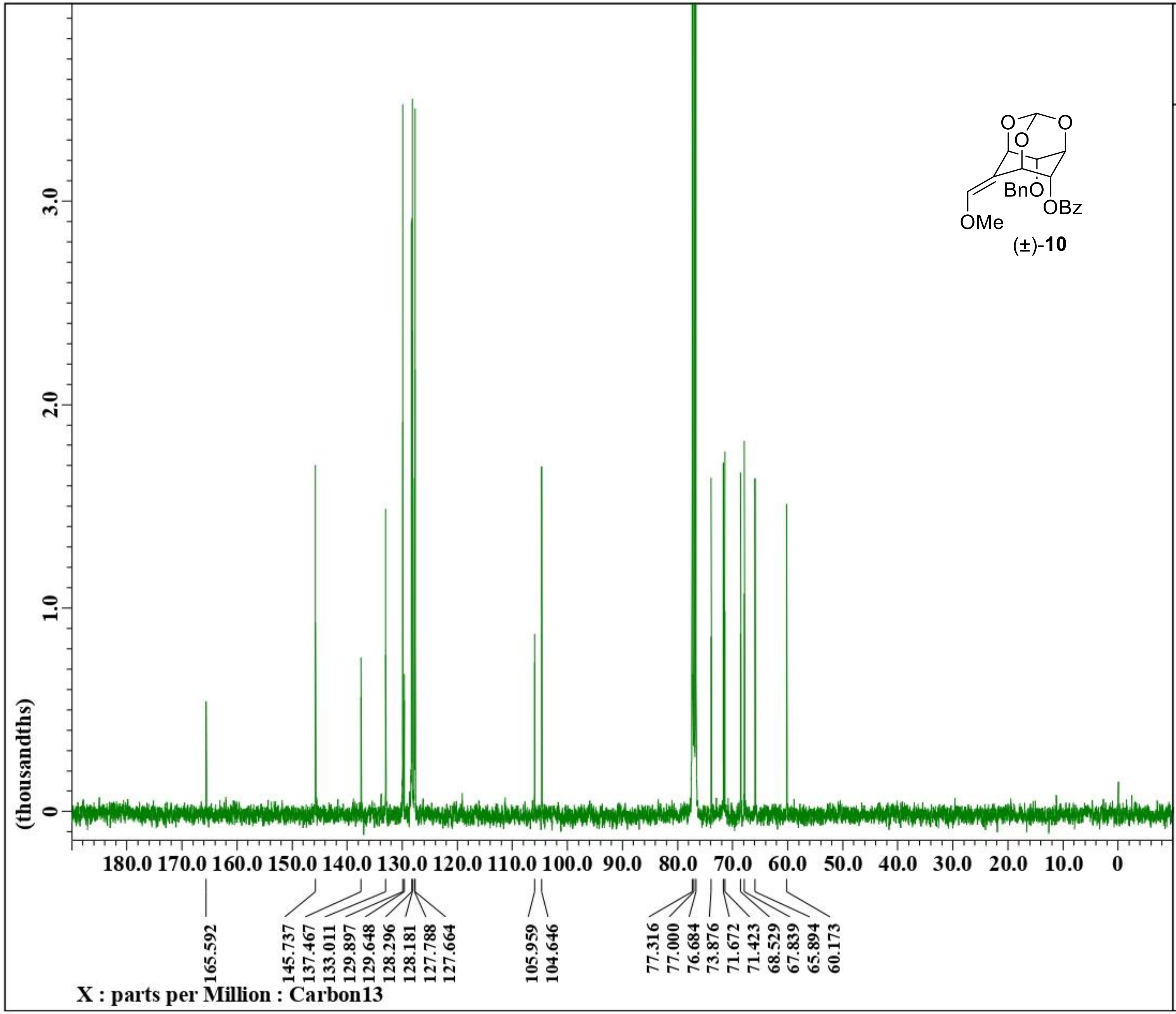

Filename	= 18kus7-11-1-2-1
Author	= delta
Experiment	= carbon_auto.jxp
Sample_Id	= 18kus7-11-1-2-1
Solvent	= CHLOROFORM-D
Actual_Start_Time	= 17-NOV-2021 23:
Revision_Time	= 3-OCT-2024 22:
Comment	= single pulse de
Data_Format	= 1D COMPLEX
Dim_Size	= 26214
X_Domain	= Carbon13
Dim_Title	= Carbon13
Dim_Units	= [ppm]
Dimensions	= X
Spectrometer	= JNM-ECZ400S/L1
Field_Strength	= 9.389766[T] (40
X_Acq_Duration	= 1.03809024[s]
X_Domain	= Carbon13
X_Freq	= 100.52530333[MH
X_Offset	= 100[ppm]
X_Points	= 32768
X_Prescans	= 4
X_Resolution	= 0.96330739[Hz]
X_Sweep	= 31.56565657[kHz
X_Sweep_Clipped	= 25.25252525[kHz
Irr_Domain	= Proton
Irr_Freq	= 399.78219838[MH
Irr_Offset	= 5[ppm]
Blanking	= 5.0[us]
Clipped	= FALSE
Scans	= 1024
Total_Scans	= 1024
Relaxation_Delay	= 2[s]
Recvr_Gain	= 50
Temp_Get	= 21.8[dC]
X_90_Width	= 10.5[us]
X_Acq_Time	= 1.03809024[s]
X_Angle	= 30[deg]
X_Atn	= 8.2[dB]
X_Pulse	= 3.5[us]
Irr_Atn_Dec	= 31.323[dB]
Irr_Atn_Dec_Calc	= 31.323[dB]
Irr_Atn_Dec_Default_Calc	= 31.323[dB]
Irr_Atn_Noe	= 31.323[dB]
Irr_Dec_Bandwidth_Hz	= 4.7826087[kHz]
Irr_Dec_Bandwidth_Ppm	= 11.96303566[ppm
Irr_Dec_Freq	= 399.78219838[MH
Irr_Dec_Merit_Factor	= 2.2
Irr_Decoupling	= TRUE
Irr_Noe	= TRUE

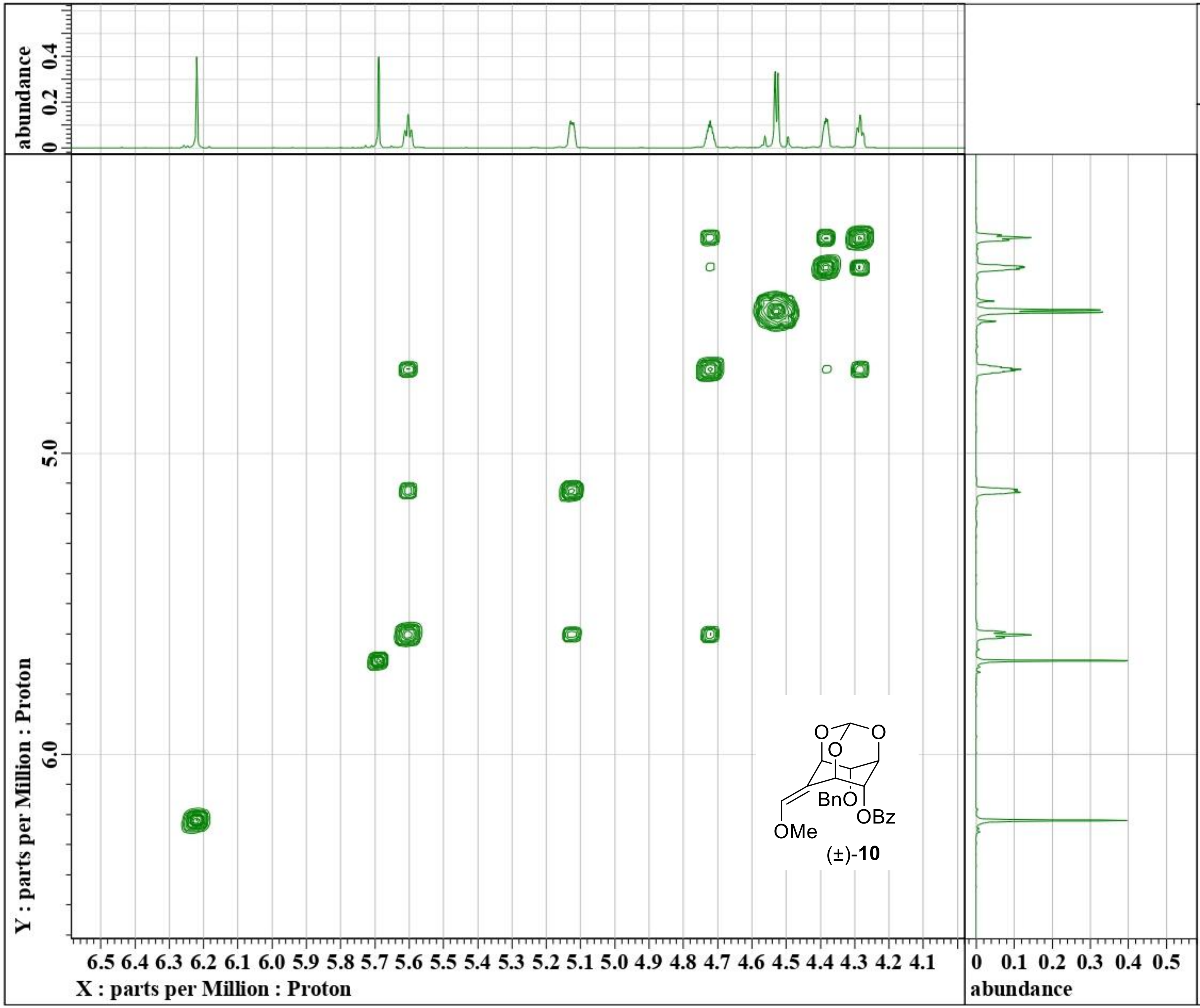

Filename	= 18kus9-92-2 1_(
Author	= delta
Experiment	= cosy_auto.jxp
Sample_Id	= 18kus9-92-2 1
Solvent	= CHLOROFORM-D
Actual_Start_Time	= 29-OCT-2022 21:
Revision_Time	= 3-OCT-2024 20:
Comment	= gradient absolu
Data_Format	= 2D REAL REAL
Dim_Size	= 1024, 1024
X_Domain	= Proton
Y_Domain	= Proton
Dim_Title	= Proton Proton
Dim_Units	= [ppm] [ppm]
Dimensions	= X Y
Spectrometer	= JNM-ECZ400S/L1
Field_Strength	= 9.389766[T] (4(
X_Acq_Duration	= 0.2420736[s]
X_Domain	= Proton
X_Freq	= 399.78219838[MH
X_Offset	= 3.90450763[ppm]
X_Points	= 1280
X_Prescans	= 4
X_Resolution	= 4.13097504[Hz]
X_Sweep	= 5.28764805[kHz]
X_Sweep_Clipped	= 4.23011844[kHz]
Y_Domain	= Proton
Y_Freq	= 399.78219838[MH
Y_Offset	= 3.90450763[ppm]
Y_Points	= 256
Y_Prescans	= 0
Y_Resolution	= 16.51132809[Hz]
Y_Sweep	= 4.22689999[kHz]
Irr_Domain	= Proton
Irr_Freq	= 399.78219838[MH
Irr_Offset	= 5[ppm]
Tri_Domain	= Proton
Tri_Freq	= 399.78219838[MH
Tri_Offset	= 5[ppm]
Blanking	= 2.0[us]
Clipped	= FALSE
Scans	= 1
Total_Scans	= 256
Relaxation_Delay	= 1.5[s]
Recvr_Gain	= 56
Temp_Get	= 22.5[dC]
X_90_Width	= 6.6[us]
X_Acq_Time	= 0.2420736[s]
X_Atn	= 6.5[dB]
X_Pulse	= 6.6[us]

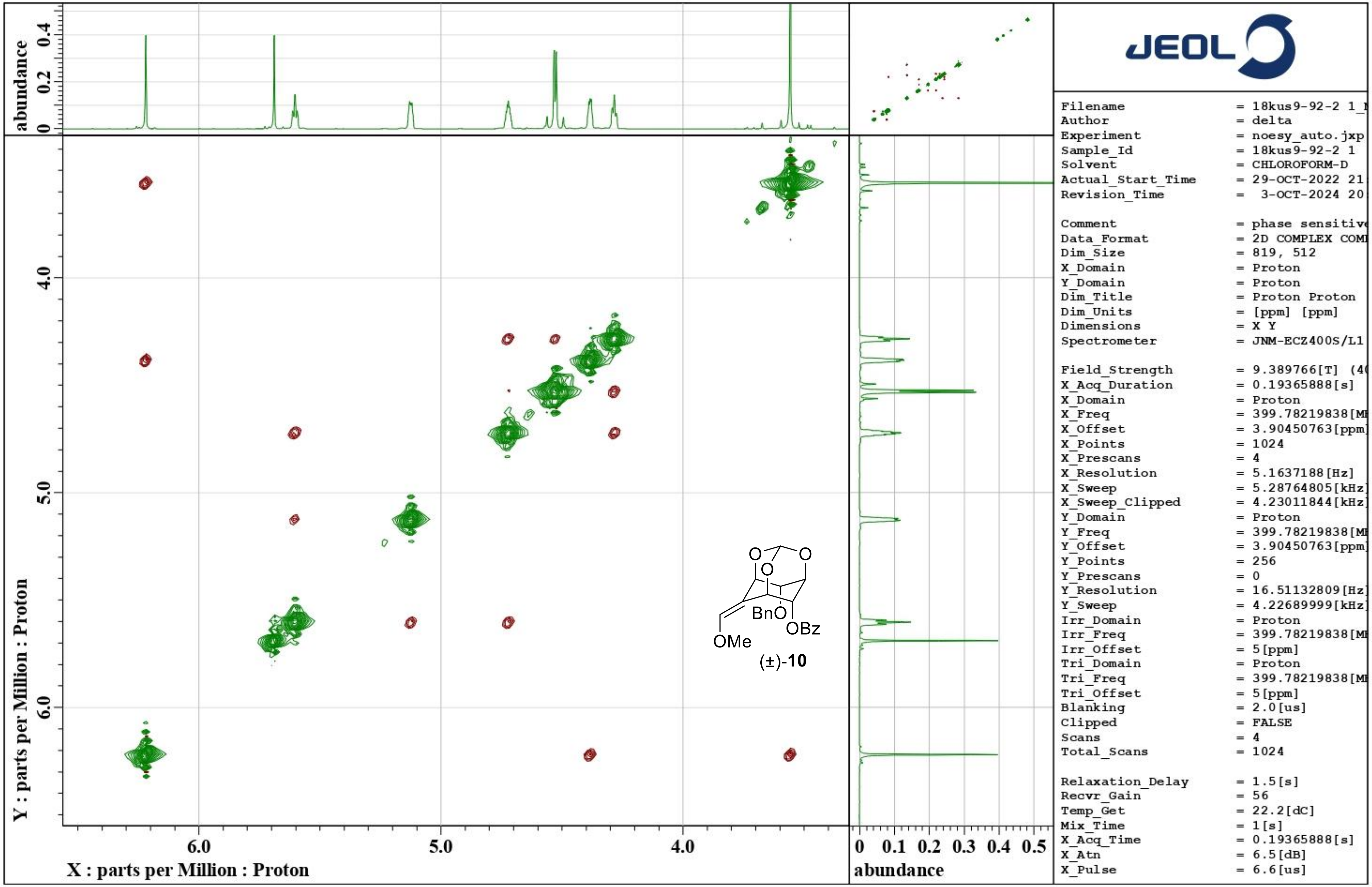
abundance
0.4
0.2
0
4.0
5.0
6.0
Y : parts per Million : Proton
6.0
5.0
4.0
X : parts per Million : Proton
0
0.1
0.2
0.3
0.4
0.5
abundance
JEOL
BnO
OBz
OMe
O
(±)-10
Filename = 18kus9-92-2 1_
Author = delta
Experiment = noesy_auto.jxp
Sample_Id = 18kus9-92-2 1
Solvent = CHLOROFORM-D
Actual_Start_Time = 29-OCT-2022 21
Revision_Time = 3-OCT-2024 20
Comment = phase sensitive
Data_Format = 2D COMPLEX COM
Dim_Size = 819, 512
X_Domain = Proton
Y_Domain = Proton
Dim_Title = Proton Proton
Dim_Units = [ppm] [ppm]
Dimensions = X Y
Spectrometer = JNM-ECZ400S/L1
Field_Strength = 9.389766[T] (4
X_Acq_Duration = 0.19365888[s]
X_Domain = Proton
X_Freq = 399.78219838[M
X_Offset = 3.90450763[ppm
X_Points = 1024
X_Prescans = 4
X_Resolution = 5.1637188[Hz]
X_Sweep = 5.28764805[kHz
X_Sweep_Clipped = 4.23011844[kHz
Y_Domain = Proton
Y_Freq = 399.78219838[M
Y_Offset = 3.90450763[ppm
Y_Points = 256
Y_Prescans = 0
Y_Resolution = 16.51132809[Hz
Y_Sweep = 4.22689999[kHz
Irr_Domain = Proton
Irr_Freq = 399.78219838[M
Irr_Offset = 5[ppm]
Tri_Domain = Proton
Tri_Freq = 399.78219838[M
Tri_Offset = 5[ppm]
Blanking = 2.0[us]
Clipped = FALSE
Scans = 4
Total_Scans = 1024
Relaxation_Delay = 1.5[s]
Recvr_Gain = 56
Temp_Get = 22.2[dC]
Mix_Time = 1[s]
X_Acq_Time = 0.19365888[s]
X_Atn = 6.5[dB]
X_Pulse = 6.6[us]

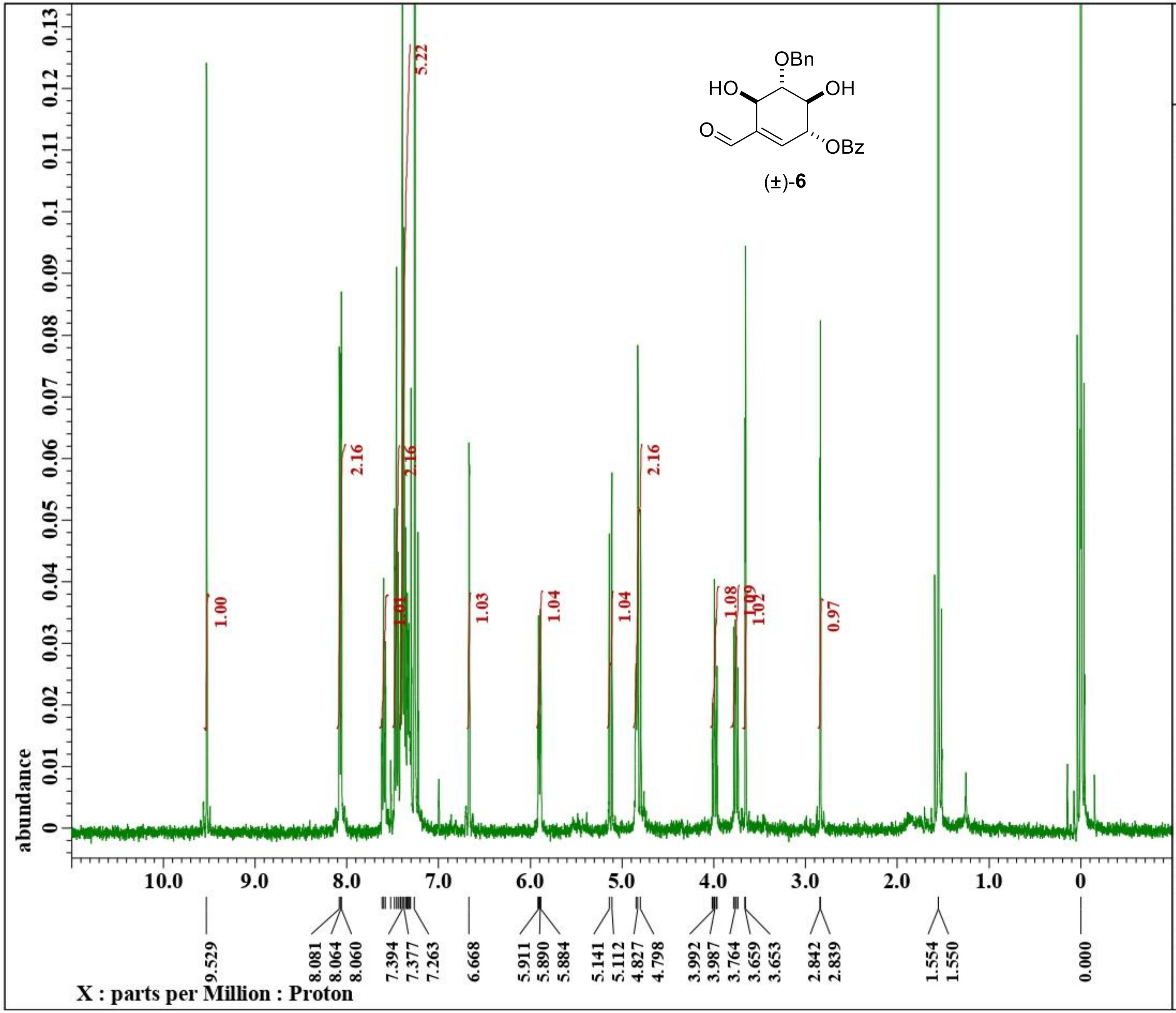

Filename	= 18kus10-49-1 1_Pro
Author	= delta
Experiment	= proton_auto.jxp
Sample_Id	= 18kus10-49-1 1
Solvent	= CHLOROFORM-D
Actual_Start_Time	= 9-DEC-2022 20:07:
Revision_Time	= 4-OCT-2024 14:51:
Comment	= single_pulse
Data_Format	= 1D COMPLEX
Dim_Size	= 13107
X_Domain	= Proton
Dim_Title	= Proton
Dim_Units	= [ppm]
Dimensions	= X
Spectrometer	= JNM-ECZ400S/L1
Field_Strength	= 9.389766[T] (400[M
X_Acq_Duration	= 2.18628096[s]
X_Domain	= Proton
X_Freq	= 399.78219838[MHz]
X_Offset	= 5[ppm]
X_Points	= 16384
X_Prescans	= 1
X_Resolution	= 0.45739775[Hz]
X_Sweep	= 7.4940048[kHz]
X_Sweep_Clipped	= 5.99520384[kHz]
Irr_Domain	= Proton
Irr_Freq	= 399.78219838[MHz]
Irr_Offset	= 5[ppm]
Tri_Domain	= Proton
Tri_Freq	= 399.78219838[MHz]
Tri_Offset	= 5[ppm]
Blanking	= 2.0[us]
Clipped	= FALSE
Scans	= 8
Total_Scans	= 8
Relaxation_Delay	= 5[s]
Recvr_Gain	= 66
Temp_Get	= 19.6[dC]
X_90_Width	= 6.6[us]
X_Acq_Time	= 2.18628096[s]
X_Angle	= 45[deg]
X_Atn	= 6.5[dB]
X_Pulse	= 3.3[us]
Irr_Mode	= Off
Tri_Mode	= Off
Dante_Loop	= 500
Dante_Presat	= FALSE
Decimation_Rate	= 0
Experiment_Path	= C:\Program Files\J
Initial_Wait	= 1[s]

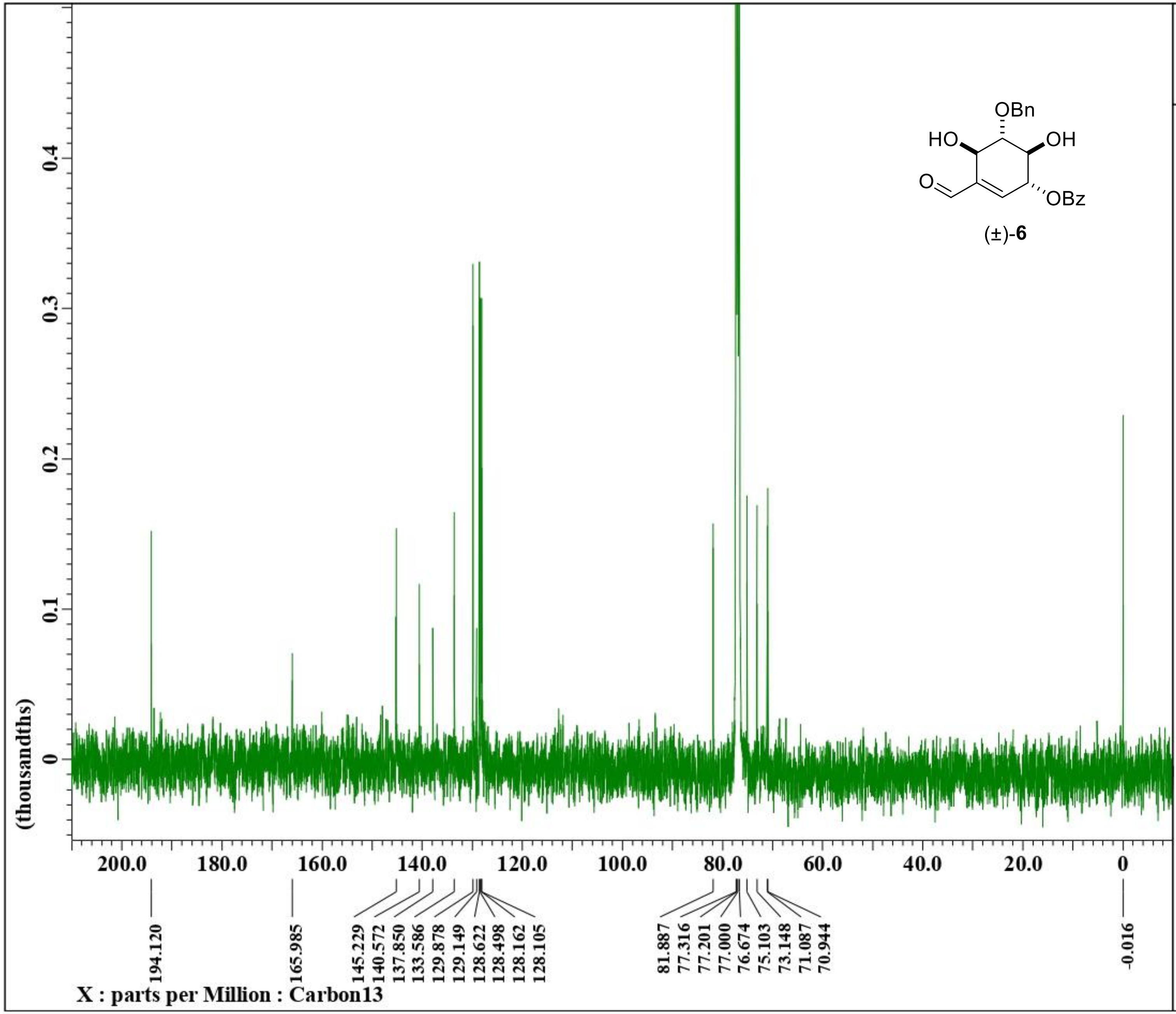

JEOL

Filename	= 18kus10-49-1_Ca
Author	= delta
Experiment	= carbon_auto.jxp
Sample_Id	= 18kus10-49-1
Solvent	= CHLOROFORM-D
Actual_Start_Time	= 8-DEC-2022 02:
Revision_Time	= 4-OCT-2024 15:
Comment	= single pulse de
Data_Format	= 1D COMPLEX
Dim_Size	= 26214
X_Domain	= Carbon13
Dim_Title	= Carbon13
Dim_Units	= [ppm]
Dimensions	= X
Spectrometer	= JNM-ECZ400S/L1
Field_Strength	= 9.389766[T] (40
X_Acq_Duration	= 1.03809024[s]
X_Domain	= Carbon13
X_Freq	= 100.52530333[MH
X_Offset	= 100[ppm]
X_Points	= 32768
X_Prescans	= 4
X_Resolution	= 0.96330739[Hz]
X_Sweep	= 31.56565657[kHz
X_Sweep_Clipped	= 25.25252525[kHz
Irr_Domain	= Proton
Irr_Freq	= 399.78219838[MH
Irr_Offset	= 5[ppm]
Blanking	= 5.0[us]
Clipped	= FALSE
Scans	= 6144
Total_Scans	= 6144
Relaxation_Delay	= 2[s]
Recvr_Gain	= 50
Temp_Get	= 18[dC]
X_90_Width	= 10.5[us]
X_Acq_Time	= 1.03809024[s]
X_Angle	= 30[deg]
X_Atn	= 8.2[dB]
X_Pulse	= 3.5[us]
Irr_Atn_Dec	= 31.323[dB]
Irr_Atn_Dec_Calc	= 31.323[dB]
Irr_Atn_Dec_Default_Calc	= 31.323[dB]
Irr_Atn_Noe	= 31.323[dB]
Irr_Dec_Bandwidth_Hz	= 4.7826087[kHz]
Irr_Dec_Bandwidth_Ppm	= 11.96303566[ppm
Irr_Dec_Freq	= 399.78219838[MH
Irr_Dec_Merit_Factor	= 2.2
Irr_Decoupling	= TRUE
Irr_Noe	= TRUE

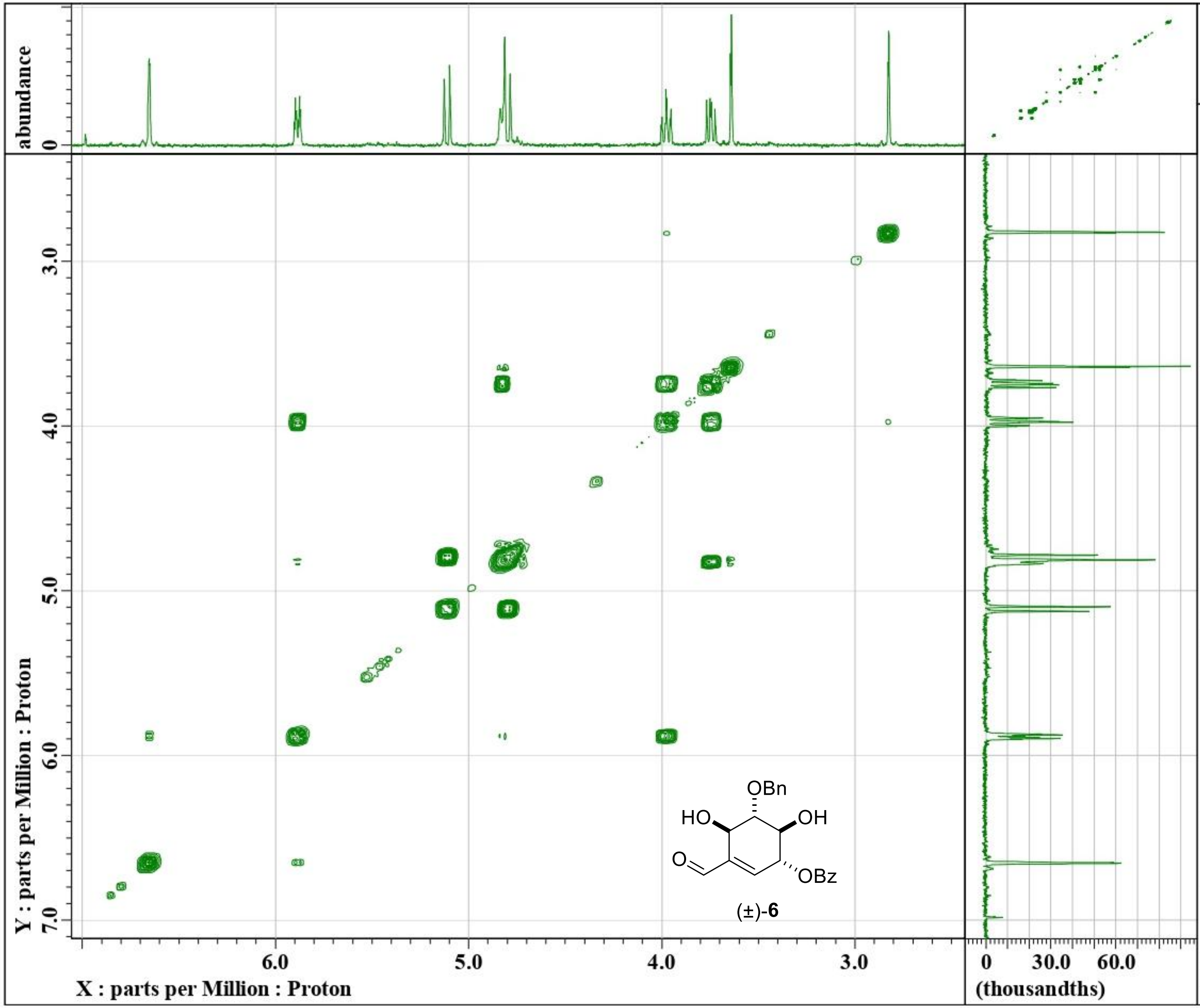

Filename	=	18kus10-49-1_C(
Author	=	delta
Experiment	=	cosy_auto.jxp
Sample_Id	=	18kus10-49-1
Solvent	=	CHLOROFORM-D
Actual_Start_Time	=	8-DEC-2022 07
Revision_Time	=	4-OCT-2024 11
Comment	=	gradient absolu
Data_Format	=	2D REAL REAL
Dim_Size	=	1024, 1024
X_Domain	=	Proton
Y_Domain	=	Proton
Dim_Title	=	Proton Proton
Dim_Units	=	[ppm] [ppm]
Dimensions	=	X Y
Spectrometer	=	JNM-ECZ400S/L1
Field_Strength	=	9.389766[T] (4(
X_Acq_Duration	=	0.2011136[s]
X_Domain	=	Proton
X_Freq	=	399.78219838[MI
X_Offset	=	4.72598389[ppm]
X_Points	=	1280
X_Prescans	=	4
X_Resolution	=	4.97231415[Hz]
X_Sweep	=	6.36456212[kHz]
X_Sweep_Clipped	=	5.09164969[kHz]
Y_Domain	=	Proton
Y_Freq	=	399.78219838[MI
Y_Offset	=	4.72598389[ppm]
Y_Points	=	256
Y_Prescans	=	0
Y_Resolution	=	19.85690321[Hz]
Y_Sweep	=	5.08336722[kHz]
Irr_Domain	=	Proton
Irr_Freq	=	399.78219838[MI
Irr_Offset	=	5[ppm]
Tri_Domain	=	Proton
Tri_Freq	=	399.78219838[MI
Tri_Offset	=	5[ppm]
Blanking	=	2.0[us]
Clipped	=	FALSE
Scans	=	1
Total_Scans	=	256
Relaxation_Delay	=	1.5[s]
Recvr_Gain	=	56
Temp_Get	=	17.9[dC]
X_90_Width	=	6.6[us]
X_Acq_Time	=	0.2011136[s]
X_Atn	=	6.5[dB]
X_Pulse	=	6.6[us]

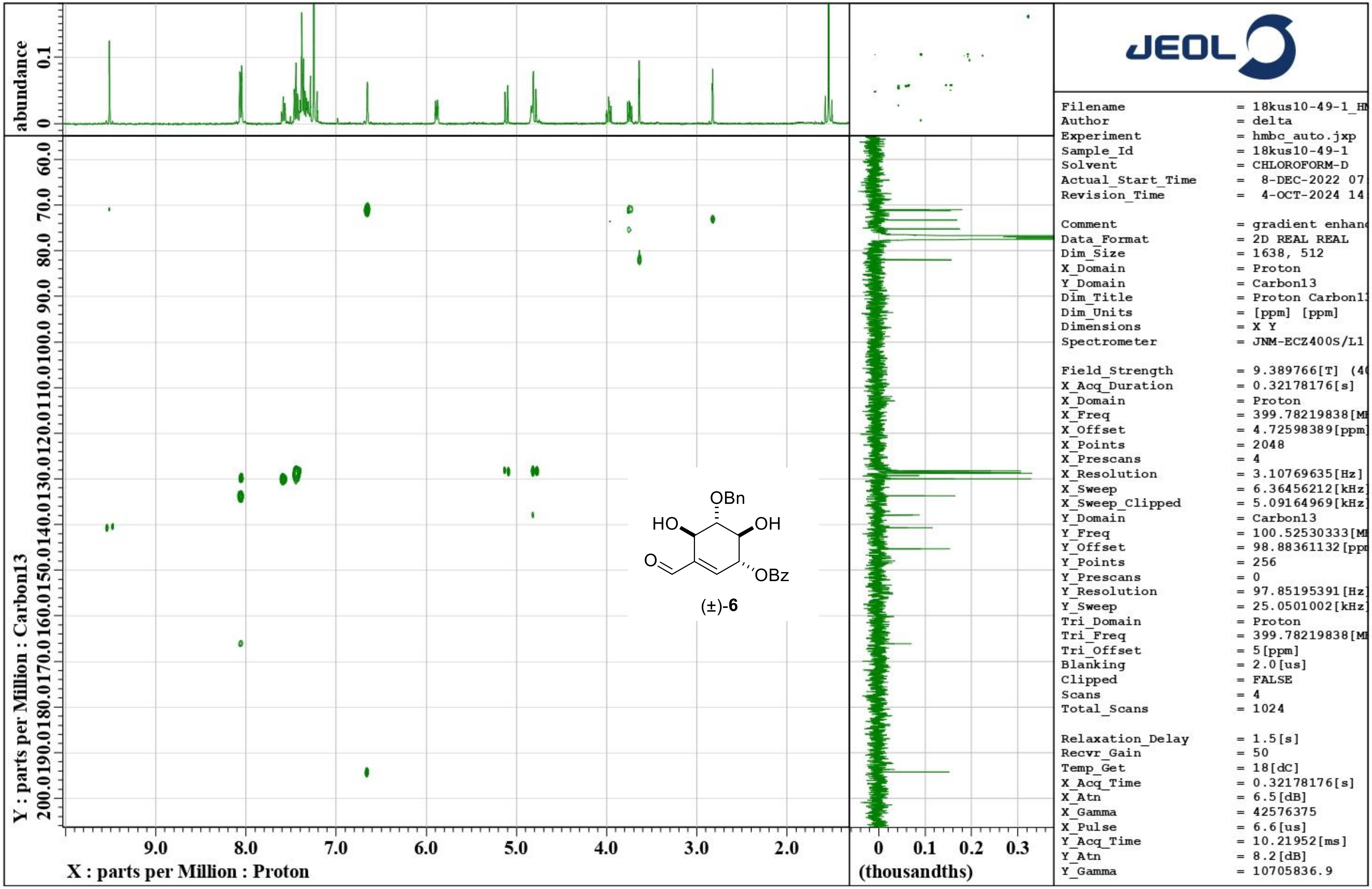

Filename	= 18kus10-49-1_HM
Author	= delta
Experiment	= hmbc_auto.jxp
Sample_Id	= 18kus10-49-1
Solvent	= CHLOROFORM-D
Actual_Start_Time	= 8-DEC-2022 07
Revision_Time	= 4-OCT-2024 14
Comment	= gradient enhan
Data_Format	= 2D REAL REAL
Dim_Size	= 1638, 512
X_Domain	= Proton
Y_Domain	= Carbon13
Dim_Title	= Proton Carbon1
Dim_Units	= [ppm] [ppm]
Dimensions	= X Y
Spectrometer	= JNM-ECZ400S/L1
Field_Strength	= 9.389766[T] (4
X_Acq_Duration	= 0.32178176[s]
X_Domain	= Proton
X_Freq	= 399.78219838[M
X_Offset	= 4.72598389[ppm]
X_Points	= 2048
X_Prescans	= 4
X_Resolution	= 3.10769635[Hz]
X_Sweep	= 6.36456212[kHz]
X_Sweep_Clipped	= 5.09164969[kHz]
Y_Domain	= Carbon13
Y_Freq	= 100.52530333[M
Y_Offset	= 98.88361132[pp
Y_Points	= 256
Y_Prescans	= 0
Y_Resolution	= 97.85195391[Hz]
Y_Sweep	= 25.0501002[kHz]
Tri_Domain	= Proton
Tri_Freq	= 399.78219838[M
Tri_Offset	= 5[ppm]
Blanking	= 2.0[us]
Clipped	= FALSE
Scans	= 4
Total_Scans	= 1024
Relaxation_Delay	= 1.5[s]
Recvr_Gain	= 50
Temp_Get	= 18[dC]
X_Acq_Time	= 0.32178176[s]
X_Atn	= 6.5[dB]
X_Gamma	= 42576375
X_Pulse	= 6.6[us]
Y_Acq_Time	= 10.21952[ms]
Y_Atn	= 8.2[dB]
Y_Gamma	= 10705836.9

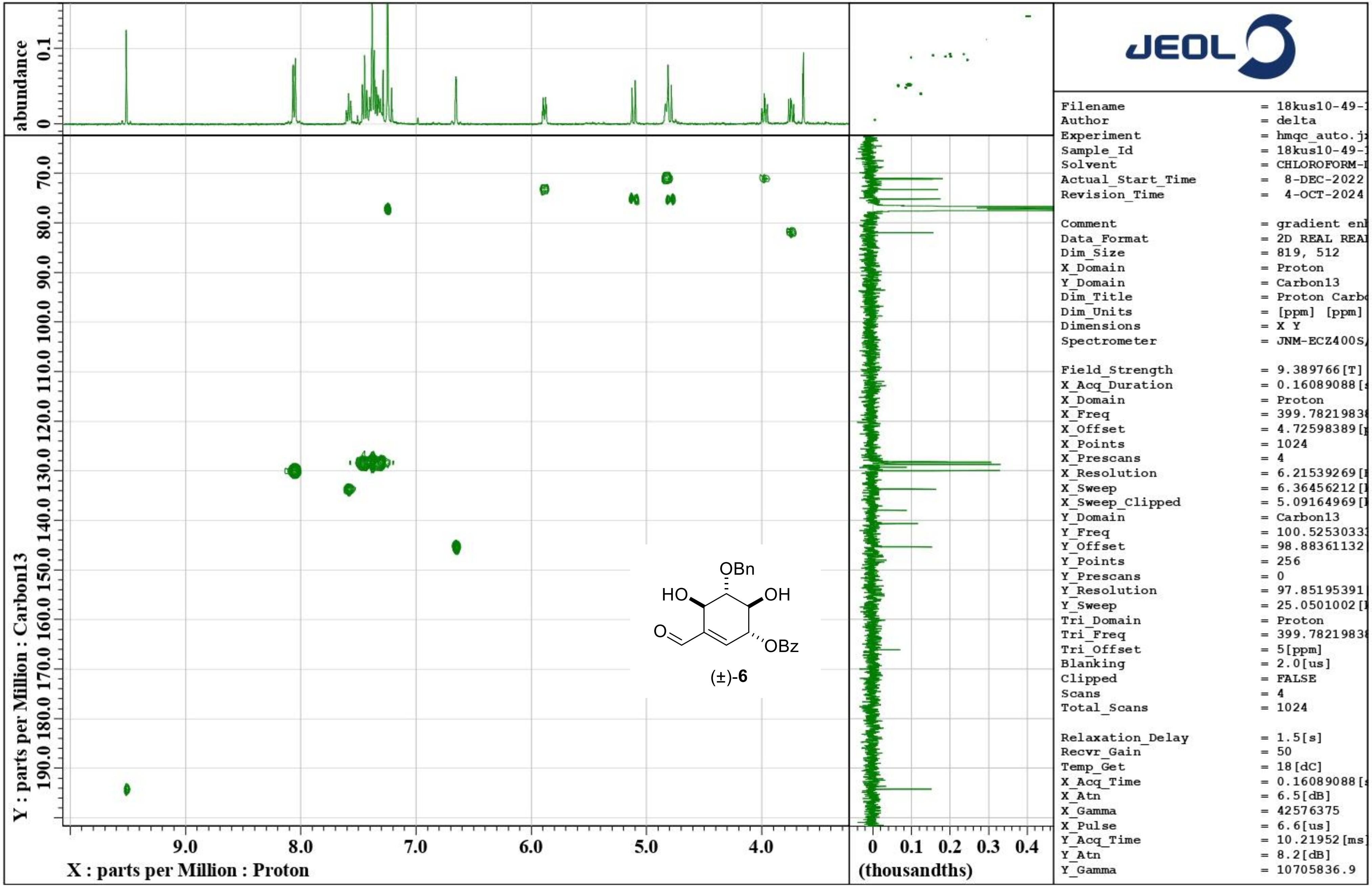
JEOL
abundance
0.1
0
Y : parts per Million : Carbon13
70.0
80.0
90.0
100.0
110.0
120.0
130.0
140.0
150.0
160.0
170.0
180.0
190.0
9.0
8.0
7.0
6.0
5.0
4.0
X : parts per Million : Proton
0
0.1
0.2
0.3
0.4
(thousandths)
OBn
HO
OH
O
OBz
(±)-6
Filename = 18kus10-49-
Author = delta
Experiment = hmqc_auto.j
Sample_Id = 18kus10-49-
Solvent = CHLOROFORM-
Actual_Start_Time = 8-DEC-2022
Revision_Time = 4-OCT-2024
Comment = gradient en
Data_Format = 2D REAL REA
Dim_Size = 819, 512
X_Domain = Proton
Y_Domain = Carbon13
Dim_Title = Proton Carb
Dim_Units = [ppm] [ppm]
Dimensions = X Y
Spectrometer = JNM-ECZ400S
Field_Strength = 9.389766[T]
X_Acq_Duration = 0.16089088[
X_Domain = Proton
X_Freq = 399.7821983
X_Offset = 4.72598389[
X_Points = 1024
X_Prescans = 4
X_Resolution = 6.21539269[
X_Sweep = 6.36456212[
X_Sweep_Clipped = 5.09164969[
Y_Domain = Carbon13
Y_Freq = 100.5253033
Y_Offset = 98.88361132
Y_Points = 256
Y_Prescans = 0
Y_Resolution = 97.85195391
Y_Sweep = 25.0501002[
Tri_Domain = Proton
Tri_Freq = 399.7821983
Tri_Offset = 5[ppm]
Blanking = 2.0[us]
Clipped = FALSE
Scans = 4
Total_Scans = 1024
Relaxation_Delay = 1.5[s]
Recvr_Gain = 50
Temp_Get = 18[dC]
X_Acq_Time = 0.16089088[
X_Atn = 6.5[dB]
X_Gamma = 42576375
X_Pulse = 6.6[us]
Y_Acq_Time = 10.21952[ms]
Y_Atn = 8.2[dB]
Y_Gamma = 10705836.9

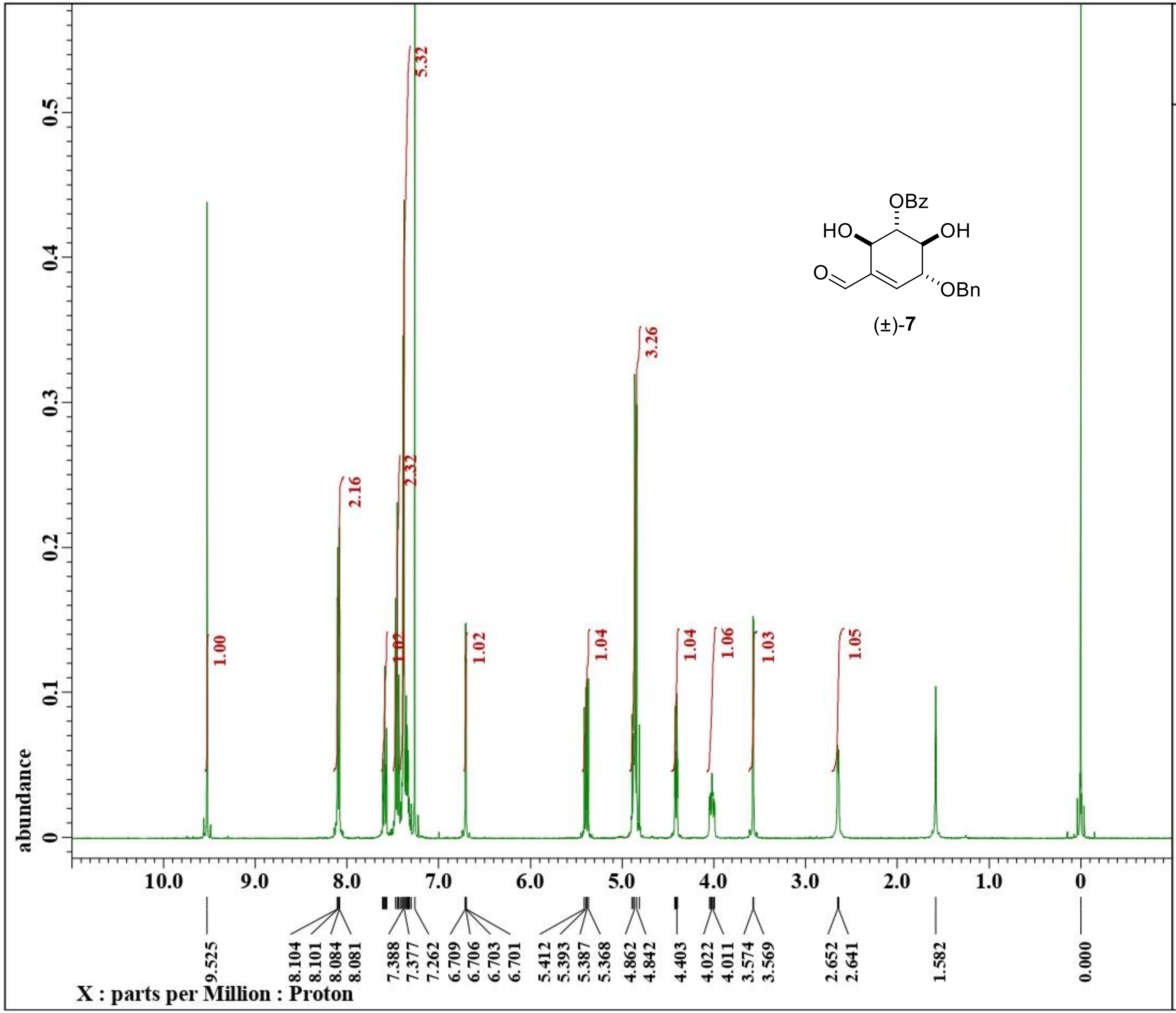

JEOL

Filename	= 18kus10-49-2_Proto
Author	= delta
Experiment	= proton_auto.jxp
Sample_Id	= 18kus10-49-2
Solvent	= CHLOROFORM-D
Actual_Start_Time	= 8-DEC-2022 20:35:
Revision_Time	= 4-OCT-2024 20:21:
Comment	= single_pulse
Data_Format	= 1D COMPLEX
Dim_Size	= 13107
X_Domain	= Proton
Dim_Title	= Proton
Dim_Units	= [ppm]
Dimensions	= X
Spectrometer	= JNM-ECZ400S/L1
Field_Strength	= 9.389766[T] (400[M
X_Acq_Duration	= 2.18628096[s]
X_Domain	= Proton
X_Freq	= 399.78219838[MHz]
X_Offset	= 5[ppm]
X_Points	= 16384
X_Prescans	= 1
X_Resolution	= 0.45739775[Hz]
X_Sweep	= 7.4940048[kHz]
X_Sweep_Clipped	= 5.99520384[kHz]
Irr_Domain	= Proton
Irr_Freq	= 399.78219838[MHz]
Irr_Offset	= 5[ppm]
Tri_Domain	= Proton
Tri_Freq	= 399.78219838[MHz]
Tri_Offset	= 5[ppm]
Blanking	= 2.0[us]
Clipped	= FALSE
Scans	= 8
Total_Scans	= 8
Relaxation_Delay	= 5[s]
Recvr_Gain	= 56
Temp_Get	= 19.5[dC]
X_90_Width	= 6.6[us]
X_Acq_Time	= 2.18628096[s]
X_Angle	= 45[deg]
X_Atn	= 6.5[dB]
X_Pulse	= 3.3[us]
Irr_Mode	= Off
Tri_Mode	= Off
Dante_Loop	= 500
Dante_Presat	= FALSE
Decimation_Rate	= 0
Experiment_Path	= C:\Program Files\J
Initial_Wait	= 1[s]

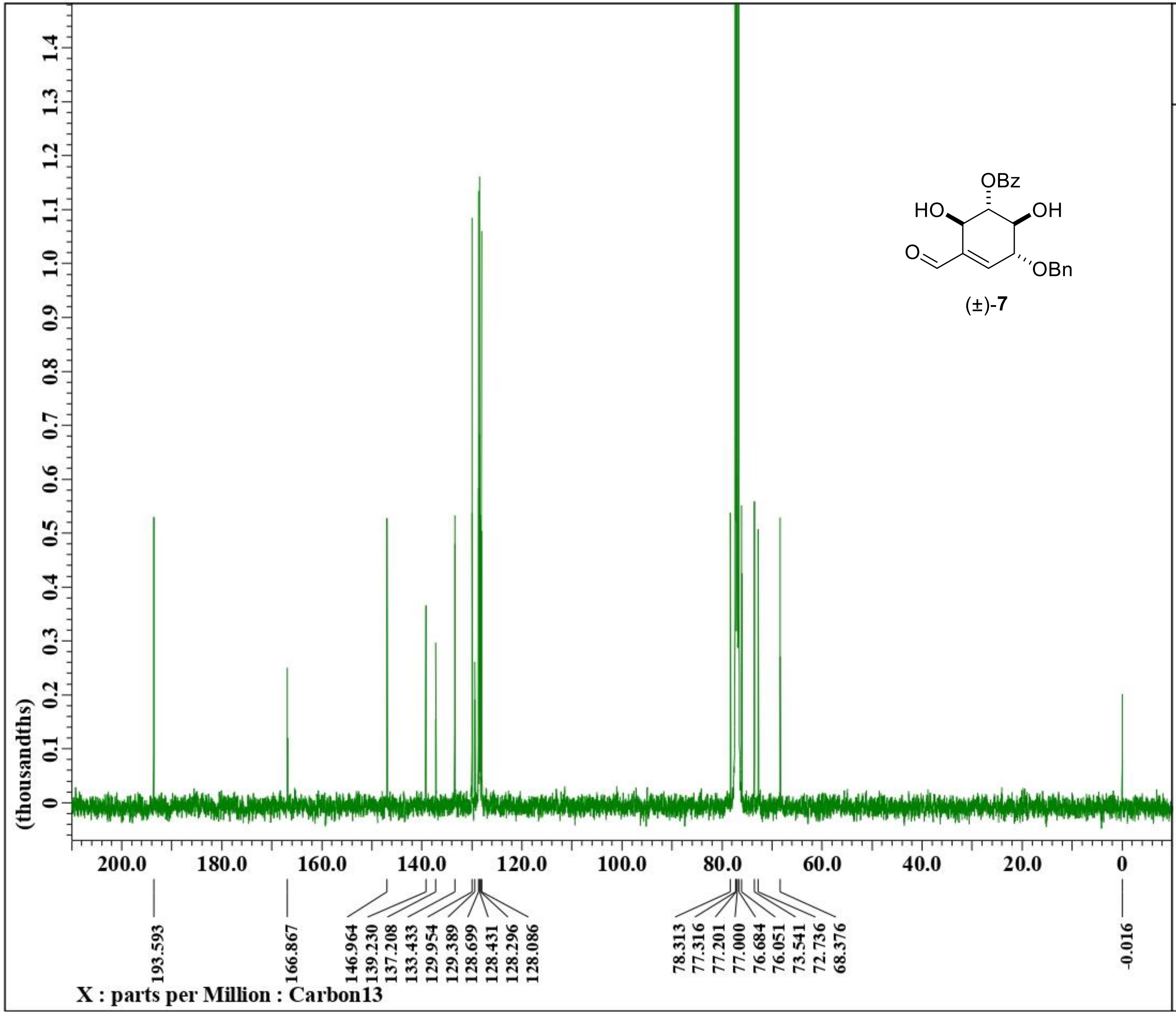

(±)-**7**

JEOL

Filename	= 18kus10-49-2_Ca
Author	= delta
Experiment	= carbon_auto.jxp
Sample_Id	= 18kus10-49-2
Solvent	= CHLOROFORM-D
Actual_Start_Time	= 9-DEC-2022 02:
Revision_Time	= 4-OCT-2024 20:
Comment	= single pulse de
Data_Format	= 1D COMPLEX
Dim_Size	= 26214
X_Domain	= Carbon13
Dim_Title	= Carbon13
Dim_Units	= [ppm]
Dimensions	= X
Spectrometer	= JNM-ECZ400S/L1
Field_Strength	= 9.389766[T] (40
X_Acq_Duration	= 1.03809024[s]
X_Domain	= Carbon13
X_Freq	= 100.52530333[MH
X_Offset	= 100[ppm]
X_Points	= 32768
X_Prescans	= 4
X_Resolution	= 0.96330739[Hz]
X_Sweep	= 31.56565657[kHz
X_Sweep_Clipped	= 25.25252525[kHz
Irr_Domain	= Proton
Irr_Freq	= 399.78219838[MH
Irr_Offset	= 5[ppm]
Blanking	= 5.0[us]
Clipped	= FALSE
Scans	= 6144
Total_Scans	= 6144
Relaxation_Delay	= 2[s]
Recvr_Gain	= 50
Temp_Get	= 18.1[dC]
X_90_Width	= 10.5[us]
X_Acq_Time	= 1.03809024[s]
X_Angle	= 30[deg]
X_Atn	= 8.2[dB]
X_Pulse	= 3.5[us]
Irr_Atn_Dec	= 31.323[dB]
Irr_Atn_Dec_Calc	= 31.323[dB]
Irr_Atn_Dec_Default_Calc	= 31.323[dB]
Irr_Atn_Noe	= 31.323[dB]
Irr_Dec_Bandwidth_Hz	= 4.7826087[kHz]
Irr_Dec_Bandwidth_Ppm	= 11.96303566[ppm
Irr_Dec_Freq	= 399.78219838[MH
Irr_Dec_Merit_Factor	= 2.2
Irr_Decoupling	= TRUE
Irr_Noe	= TRUE

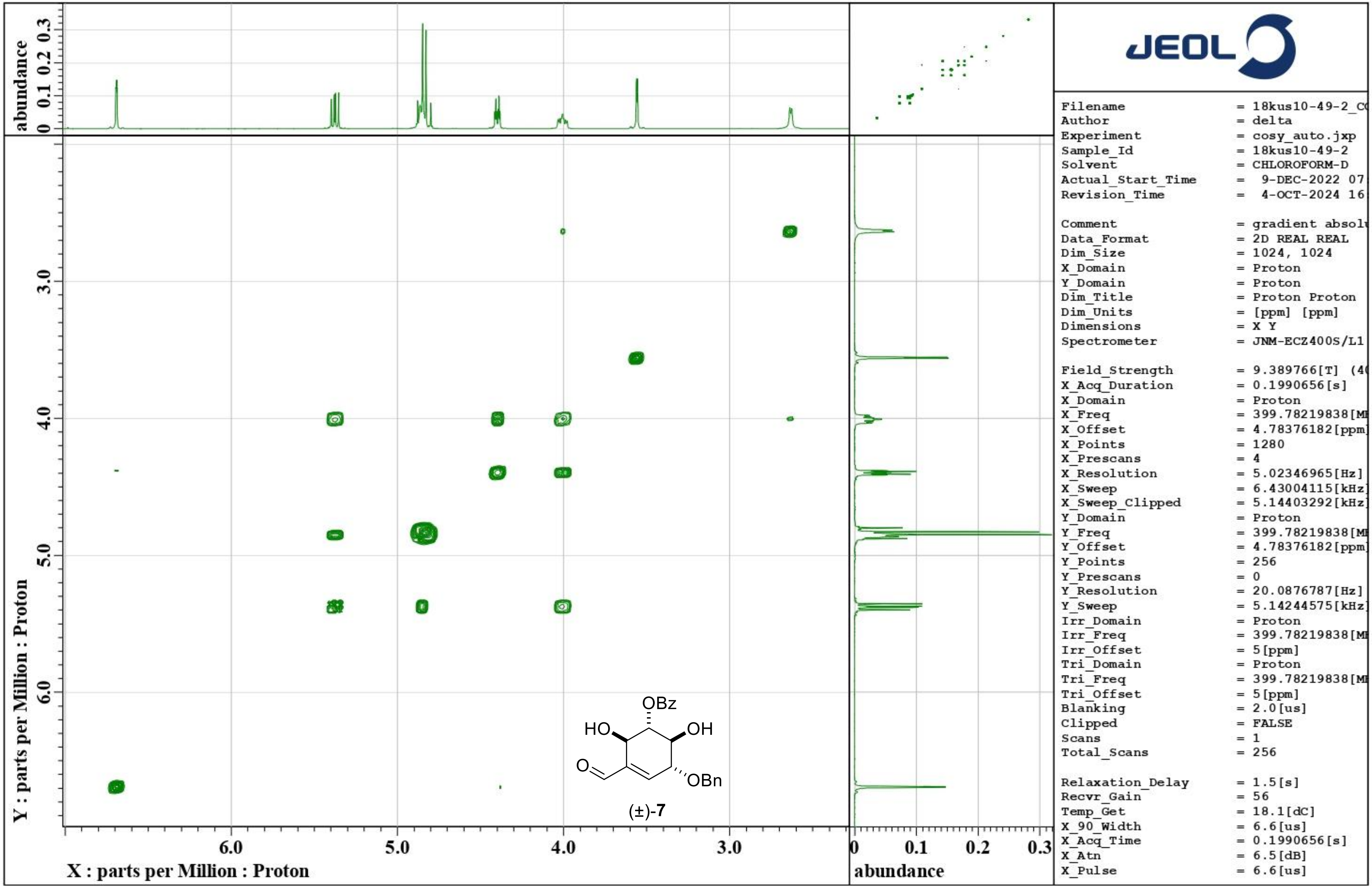

Filename	=	18kus10-49-2_CC
Author	=	delta
Experiment	=	cosy_auto.jxp
Sample_Id	=	18kus10-49-2
Solvent	=	CHLOROFORM-D
Actual_Start_Time	=	9-DEC-2022 07:
Revision_Time	=	4-OCT-2024 16:
Comment	=	gradient absolu
Data_Format	=	2D REAL REAL
Dim_Size	=	1024, 1024
X_Domain	=	Proton
Y_Domain	=	Proton
Dim_Title	=	Proton Proton
Dim_Units	=	[ppm] [ppm]
Dimensions	=	X Y
Spectrometer	=	JNM-ECZ400S/L1
Field_Strength	=	9.389766[T] (40
X_Acq_Duration	=	0.1990656[s]
X_Domain	=	Proton
X_Freq	=	399.78219838[MH
X_Offset	=	4.78376182[ppm]
X_Points	=	1280
X_Prescans	=	4
X_Resolution	=	5.02346965[Hz]
X_Sweep	=	6.43004115[kHz]
X_Sweep_Clipped	=	5.14403292[kHz]
Y_Domain	=	Proton
Y_Freq	=	399.78219838[MH
Y_Offset	=	4.78376182[ppm]
Y_Points	=	256
Y_Prescans	=	0
Y_Resolution	=	20.0876787[Hz]
Y_Sweep	=	5.14244575[kHz]
Irr_Domain	=	Proton
Irr_Freq	=	399.78219838[MH
Irr_Offset	=	5[ppm]
Tri_Domain	=	Proton
Tri_Freq	=	399.78219838[MH
Tri_Offset	=	5[ppm]
Blanking	=	2.0[us]
Clipped	=	FALSE
Scans	=	1
Total_Scans	=	256
Relaxation_Delay	=	1.5[s]
Recvr_Gain	=	56
Temp_Get	=	18.1[dC]
X_90_Width	=	6.6[us]
X_Acq_Time	=	0.1990656[s]
X_Atn	=	6.5[dB]
X_Pulse	=	6.6[us]

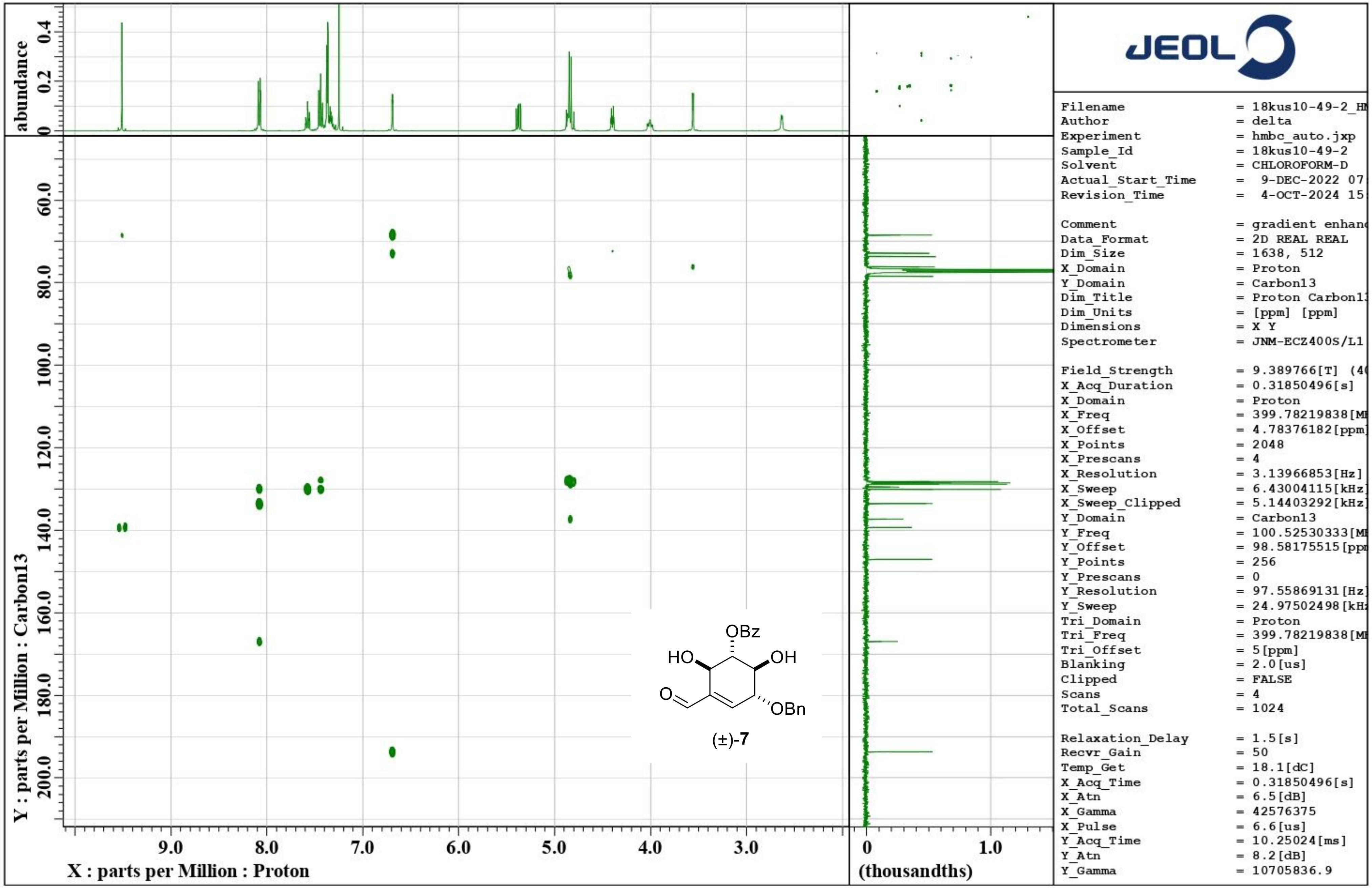

(±)-**7**

JEOL

Filename	= 18kus10-49-2_HM
Author	= delta
Experiment	= hmbc_auto.jxp
Sample_Id	= 18kus10-49-2
Solvent	= CHLOROFORM-D
Actual_Start_Time	= 9-DEC-2022 07:
Revision_Time	= 4-OCT-2024 15:
Comment	= gradient enhanc
Data_Format	= 2D REAL REAL
Dim_Size	= 1638, 512
X_Domain	= Proton
Y_Domain	= Carbon13
Dim_Title	= Proton Carbon13
Dim_Units	= [ppm] [ppm]
Dimensions	= X Y
Spectrometer	= JNM-ECZ400S/L1
Field_Strength	= 9.389766[T] (40
X_Acq_Duration	= 0.31850496[s]
X_Domain	= Proton
X_Freq	= 399.78219838[MH
X_Offset	= 4.78376182[ppm]
X_Points	= 2048
X_Prescans	= 4
X_Resolution	= 3.13966853[Hz]
X_Sweep	= 6.43004115[kHz]
X_Sweep_Clipped	= 5.14403292[kHz]
Y_Domain	= Carbon13
Y_Freq	= 100.52530333[MH
Y_Offset	= 98.58175515[ppm
Y_Points	= 256
Y_Prescans	= 0
Y_Resolution	= 97.55869131[Hz]
Y_Sweep	= 24.97502498[kHz
Tri_Domain	= Proton
Tri_Freq	= 399.78219838[MH
Tri_Offset	= 5[ppm]
Blanking	= 2.0[us]
Clipped	= FALSE
Scans	= 4
Total_Scans	= 1024
Relaxation_Delay	= 1.5[s]
Recvr_Gain	= 50
Temp_Get	= 18.1[dC]
X_Acq_Time	= 0.31850496[s]
X_Atn	= 6.5[dB]
X_Gamma	= 42576375
X_Pulse	= 6.6[us]
Y_Acq_Time	= 10.25024[ms]
Y_Atn	= 8.2[dB]
Y_Gamma	= 10705836.9

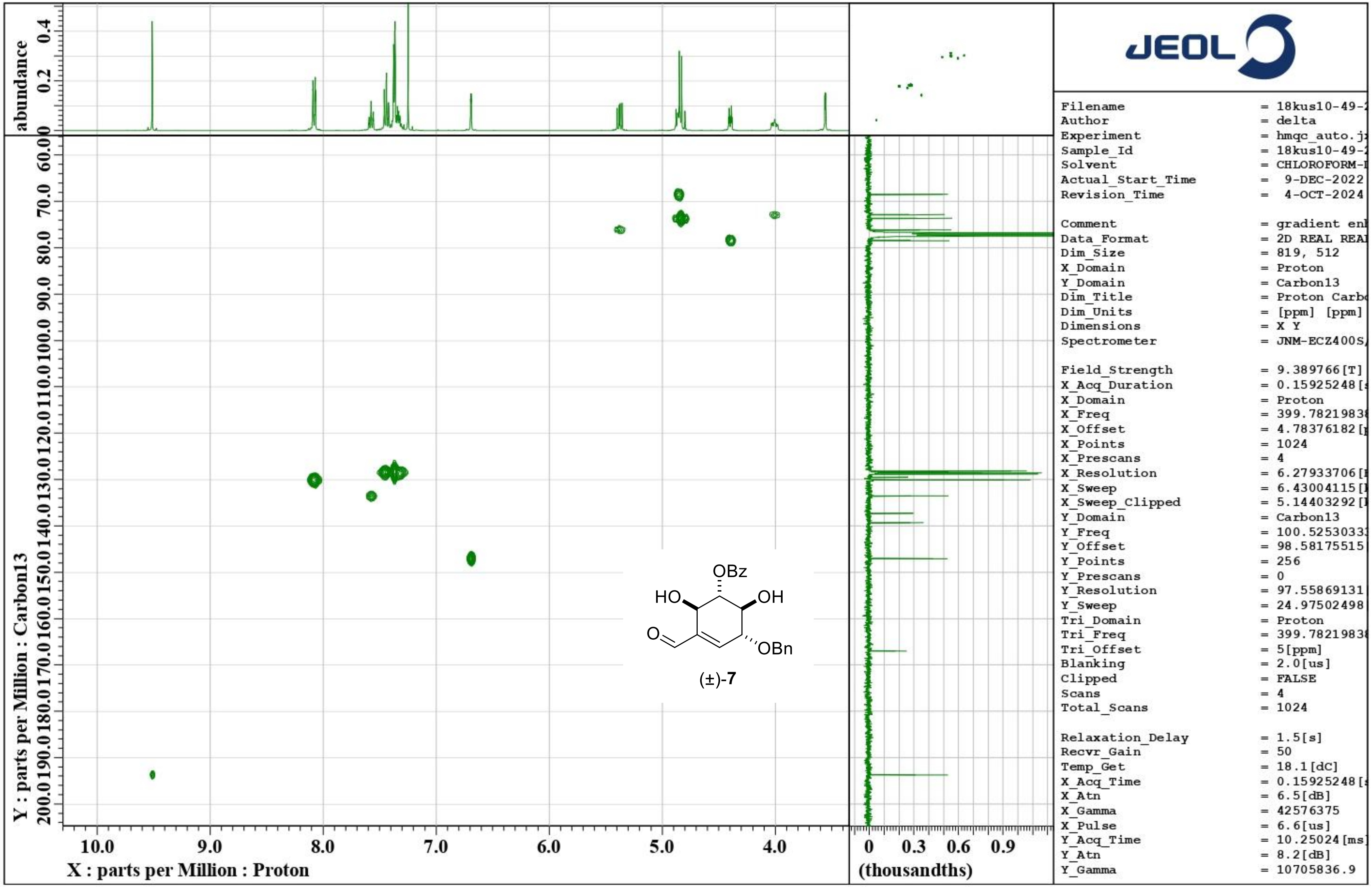
JEOL
abundance
Y : parts per Million : Carbon13
X : parts per Million : Proton
(thousandths)
OBz
HO
OH
O
OBn
(±)-7
Filename = 18kus10-49-
Author = delta
Experiment = hmqc_auto.j
Sample_Id = 18kus10-49-
Solvent = CHLOROFORM-
Actual_Start_Time = 9-DEC-2022
Revision_Time = 4-OCT-2024
Comment = gradient en
Data_Format = 2D REAL REA
Dim_Size = 819, 512
X_Domain = Proton
Y_Domain = Carbon13
Dim_Title = Proton Carb
Dim_Units = [ppm] [ppm]
Dimensions = X Y
Spectrometer = JNM-ECZ400S
Field_Strength = 9.389766[T]
X_Acq_Duration = 0.15925248[
X_Domain = Proton
X_Freq = 399.7821983
X_Offset = 4.78376182[
X_Points = 1024
X_Prescans = 4
X_Resolution = 6.27933706[
X_Sweep = 6.43004115[
X_Sweep_Clipped = 5.14403292[
Y_Domain = Carbon13
Y_Freq = 100.5253033
Y_Offset = 98.58175515
Y_Points = 256
Y_Prescans = 0
Y_Resolution = 97.55869131
Y_Sweep = 24.97502498
Tri_Domain = Proton
Tri_Freq = 399.7821983
Tri_Offset = 5[ppm]
Blanking = 2.0[us]
Clipped = FALSE
Scans = 4
Total_Scans = 1024
Relaxation_Delay = 1.5[s]
Recvr_Gain = 50
Temp_Get = 18.1[dC]
X_Acq_Time = 0.15925248[
X_Atn = 6.5[dB]
X_Gamma = 42576375
X_Pulse = 6.6[us]
Y_Acq_Time = 10.25024[ms
Y_Atn = 8.2[dB]
Y_Gamma = 10705836.9

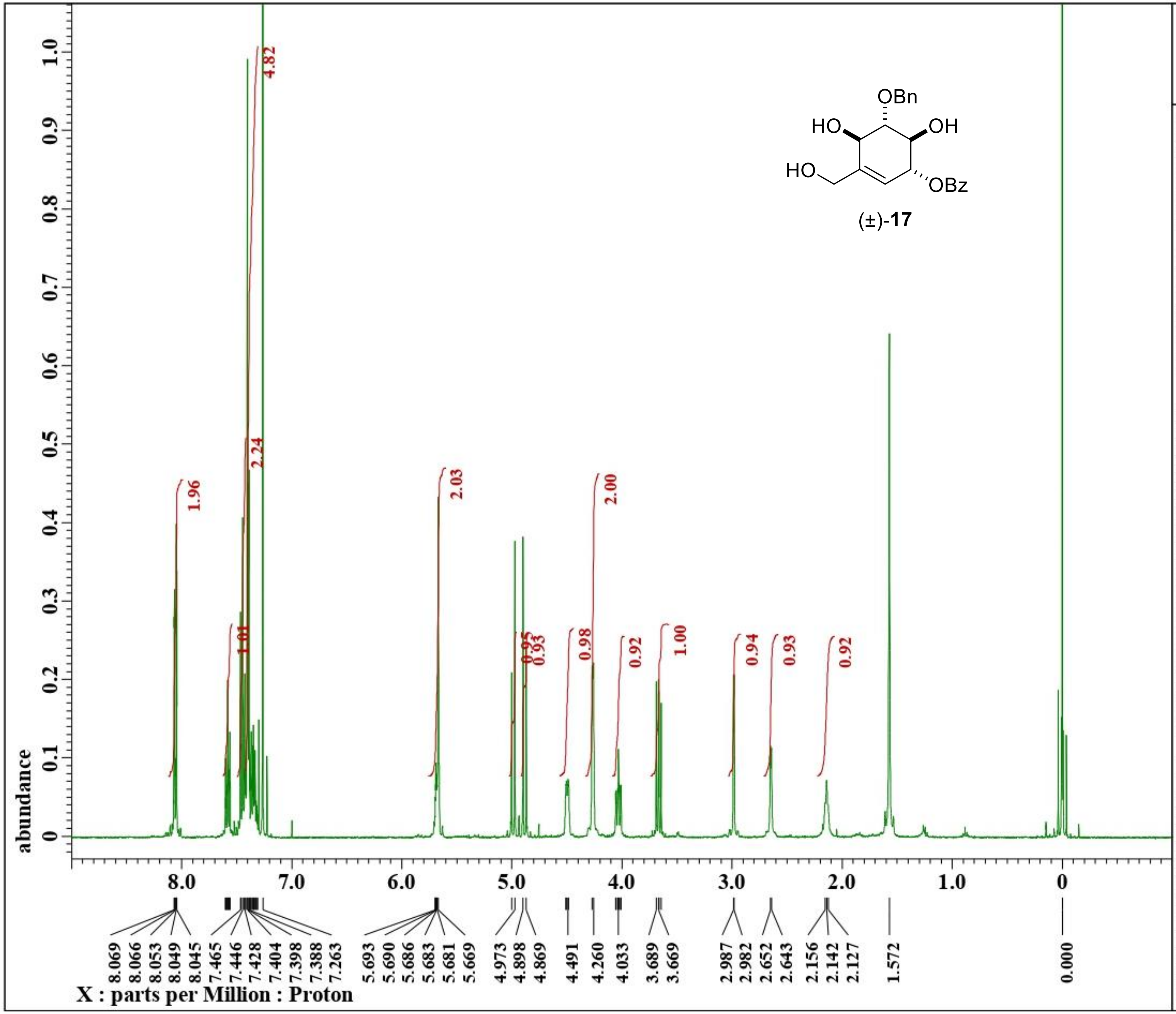

Filename	= 18kus10-94-1-1_Pro
Author	= delta
Experiment	= proton_auto.jxp
Sample_Id	= 18kus10-94-1-1
Solvent	= CHLOROFORM-D
Actual_Start_Time	= 13-JAN-2023 22:02:
Revision_Time	= 4-OCT-2024 23:12:
Comment	= single_pulse
Data_Format	= 1D COMPLEX
Dim_Size	= 13107
X_Domain	= Proton
Dim_Title	= Proton
Dim_Units	= [ppm]
Dimensions	= X
Spectrometer	= JNM-ECZ400S/L1
Field_Strength	= 9.389766[T] (400[M
X_Acq_Duration	= 2.18628096[s]
X_Domain	= Proton
X_Freq	= 399.78219838[MHz]
X_Offset	= 5[ppm]
X_Points	= 16384
X_Prescans	= 1
X_Resolution	= 0.45739775[Hz]
X_Sweep	= 7.4940048[kHz]
X_Sweep_Clipped	= 5.99520384[kHz]
Irr_Domain	= Proton
Irr_Freq	= 399.78219838[MHz]
Irr_Offset	= 5[ppm]
Tri_Domain	= Proton
Tri_Freq	= 399.78219838[MHz]
Tri_Offset	= 5[ppm]
Blanking	= 2.0[us]
Clipped	= FALSE
Scans	= 8
Total_Scans	= 8
Relaxation_Delay	= 5[s]
Recvr_Gain	= 66
Temp_Get	= 18.5[dC]
X_90_Width	= 6.6[us]
X_Acq_Time	= 2.18628096[s]
X_Angle	= 45[deg]
X_Atn	= 6.5[dB]
X_Pulse	= 3.3[us]
Irr_Mode	= Off
Tri_Mode	= Off
Dante_Loop	= 500
Dante_Presat	= FALSE
Decimation_Rate	= 0
Experiment_Path	= C:\Program Files\J
Initial_Wait	= 1[s]

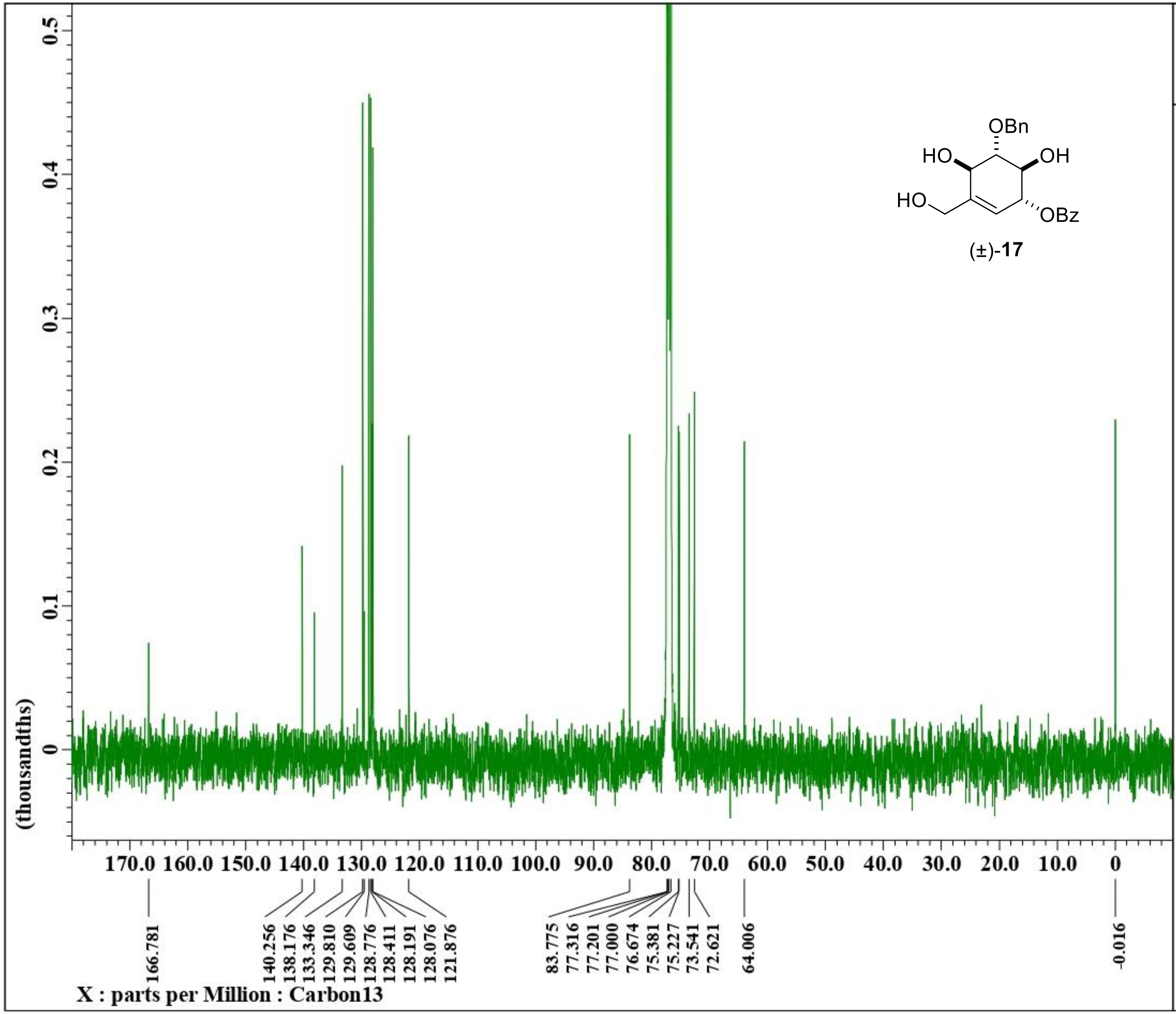

JEOL

Filename	= 18kus10-94-1-1_
Author	= delta
Experiment	= carbon_auto.jxp
Sample_Id	= 18kus10-94-1-1
Solvent	= CHLOROFORM-D
Actual_Start_Time	= 13-JAN-2023 22:
Revision_Time	= 4-OCT-2024 23:
Comment	= single pulse de
Data_Format	= 1D COMPLEX
Dim_Size	= 26214
X_Domain	= Carbon13
Dim_Title	= Carbon13
Dim_Units	= [ppm]
Dimensions	= X
Spectrometer	= JNM-ECZ400S/L1
Field_Strength	= 9.389766[T] (40
X_Acq_Duration	= 1.03809024[s]
X_Domain	= Carbon13
X_Freq	= 100.52530333[MH
X_Offset	= 100[ppm]
X_Points	= 32768
X_Prescans	= 4
X_Resolution	= 0.96330739[Hz]
X_Sweep	= 31.56565657[kHz
X_Sweep_Clipped	= 25.25252525[kHz
Irr_Domain	= Proton
Irr_Freq	= 399.78219838[MH
Irr_Offset	= 5[ppm]
Blanking	= 5.0[us]
Clipped	= FALSE
Scans	= 6000
Total_Scans	= 6000
Relaxation_Delay	= 2[s]
Recvr_Gain	= 50
Temp_Get	= 18.1[dC]
X_90_Width	= 10.5[us]
X_Acq_Time	= 1.03809024[s]
X_Angle	= 30[deg]
X_Atn	= 8.2[dB]
X_Pulse	= 3.5[us]
Irr_Atn_Dec	= 31.323[dB]
Irr_Atn_Dec_Calc	= 31.323[dB]
Irr_Atn_Dec_Default_Calc	= 31.323[dB]
Irr_Atn_Noe	= 31.323[dB]
Irr_Dec_Bandwidth_Hz	= 4.7826087[kHz]
Irr_Dec_Bandwidth_Ppm	= 11.96303566[ppm
Irr_Dec_Freq	= 399.78219838[MH
Irr_Dec_Merit_Factor	= 2.2
Irr_Decoupling	= TRUE
Irr_Noe	= TRUE

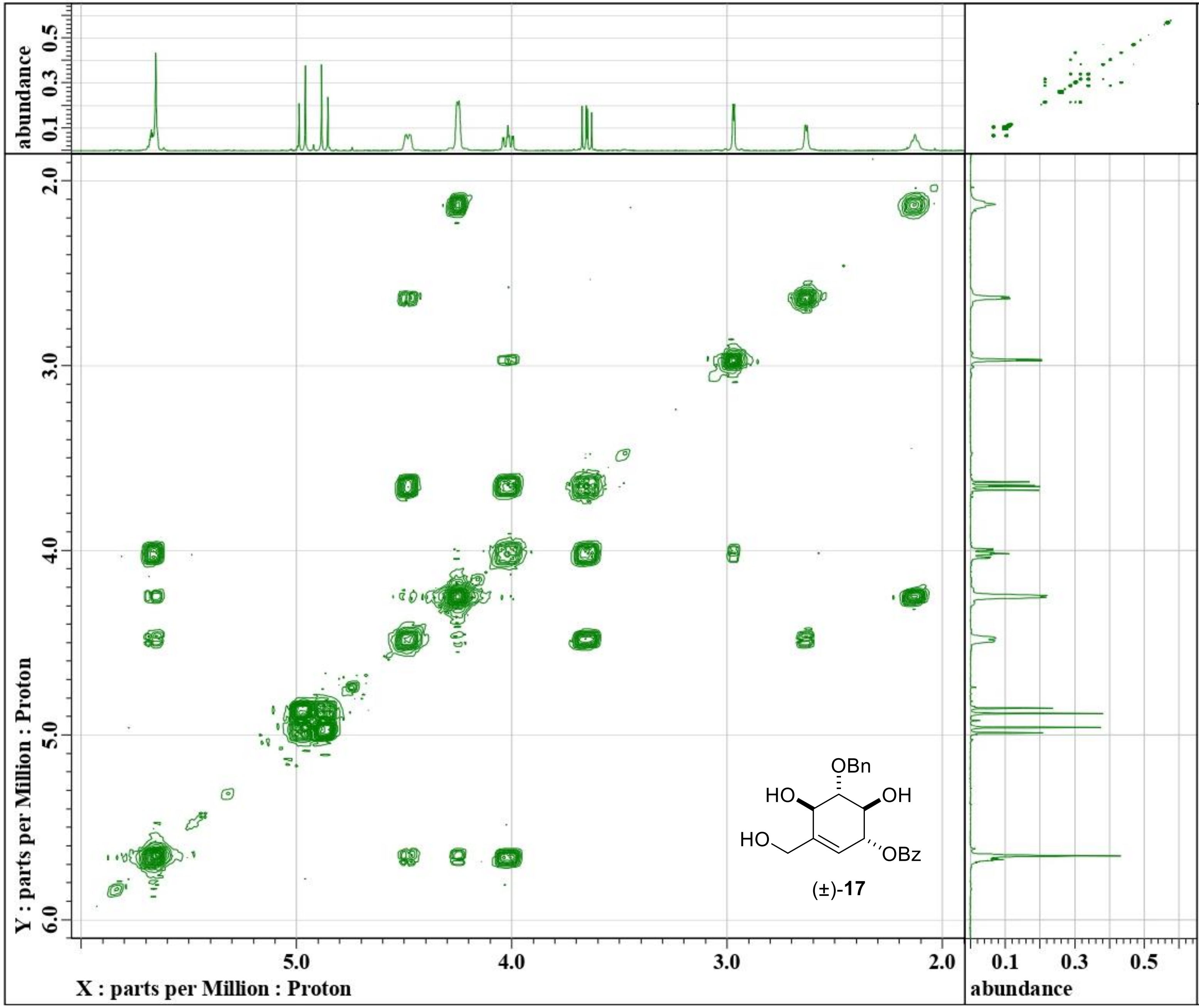

Filename	=	18kus10-94-1-1_
Author	=	delta
Experiment	=	cosy_auto.jxp
Sample_Id	=	18kus10-94-1-1
Solvent	=	CHLOROFORM-D
Actual_Start_Time	=	14-JAN-2023 03:
Revision_Time	=	4-OCT-2024 21:
Comment	=	gradient absolu
Data_Format	=	2D REAL REAL
Dim_Size	=	1024, 1024
X_Domain	=	Proton
Y_Domain	=	Proton
Dim_Title	=	Proton Proton
Dim_Units	=	[ppm] [ppm]
Dimensions	=	X Y
Spectrometer	=	JNM-ECZ400S/L1
Field_Strength	=	9.389766[T] (40
X_Acq_Duration	=	0.2379776[s]
X_Domain	=	Proton
X_Freq	=	399.78219838[MH
X_Offset	=	3.97315467[ppm]
X_Points	=	1280
X_Prescans	=	4
X_Resolution	=	4.20207616[Hz]
X_Sweep	=	5.37865749[kHz]
X_Sweep_Clipped	=	4.30292599[kHz]
Y_Domain	=	Proton
Y_Freq	=	399.78219838[MH
Y_Offset	=	3.97315467[ppm]
Y_Points	=	256
Y_Prescans	=	0
Y_Resolution	=	16.80107527[Hz]
Y_Sweep	=	4.30107527[kHz]
Irr_Domain	=	Proton
Irr_Freq	=	399.78219838[MH
Irr_Offset	=	5[ppm]
Tri_Domain	=	Proton
Tri_Freq	=	399.78219838[MH
Tri_Offset	=	5[ppm]
Blanking	=	2.0[us]
Clipped	=	FALSE
Scans	=	1
Total_Scans	=	256
Relaxation_Delay	=	1.5[s]
Recvr_Gain	=	66
Temp_Get	=	18[dC]
X_90_Width	=	6.6[us]
X_Acq_Time	=	0.2379776[s]
X_Atn	=	6.5[dB]
X_Pulse	=	6.6[us]

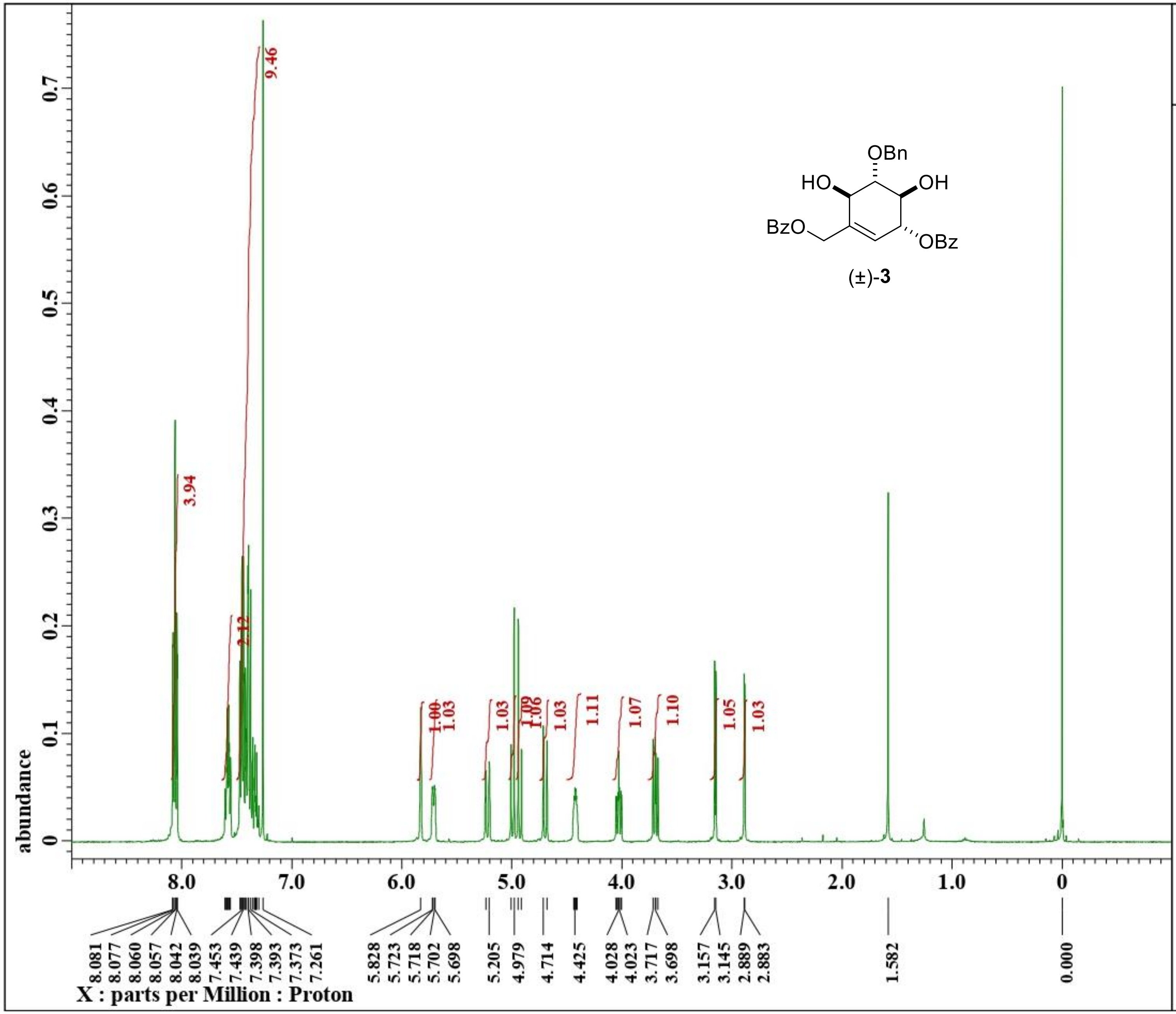

Filename	= 18kus7-93-1-5_Prot
Author	= delta
Experiment	= proton_auto.jxp
Sample_Id	= 18kus7-93-1-5
Solvent	= CHLOROFORM-D
Actual_Start_Time	= 27-FEB-2022 21:02:
Revision_Time	= 5-OCT-2024 00:32:
Comment	= single_pulse
Data_Format	= 1D COMPLEX
Dim_Size	= 13107
X_Domain	= Proton
Dim_Title	= Proton
Dim_Units	= [ppm]
Dimensions	= X
Spectrometer	= JNM-ECZ400S/L1
Field_Strength	= 9.389766[T] (400[M
X_Acq_Duration	= 2.18628096[s]
X_Domain	= Proton
X_Freq	= 399.78219838[MHz]
X_Offset	= 5[ppm]
X_Points	= 16384
X_Prescans	= 1
X_Resolution	= 0.45739775[Hz]
X_Sweep	= 7.4940048[kHz]
X_Sweep_Clipped	= 5.99520384[kHz]
Irr_Domain	= Proton
Irr_Freq	= 399.78219838[MHz]
Irr_Offset	= 5[ppm]
Tri_Domain	= Proton
Tri_Freq	= 399.78219838[MHz]
Tri_Offset	= 5[ppm]
Blanking	= 2.0[us]
Clipped	= FALSE
Scans	= 8
Total_Scans	= 8
Relaxation_Delay	= 5[s]
Recvr_Gain	= 56
Temp_Get	= 19.5[dC]
X_90_Width	= 6.6[us]
X_Acq_Time	= 2.18628096[s]
X_Angle	= 45[deg]
X_Atn	= 6.5[dB]
X_Pulse	= 3.3[us]
Irr_Mode	= Off
Tri_Mode	= Off
Dante_Loop	= 500
Dante_Presat	= FALSE
Decimation_Rate	= 0
Experiment_Path	= C:\Program Files\J
Initial_Wait	= 1[s]

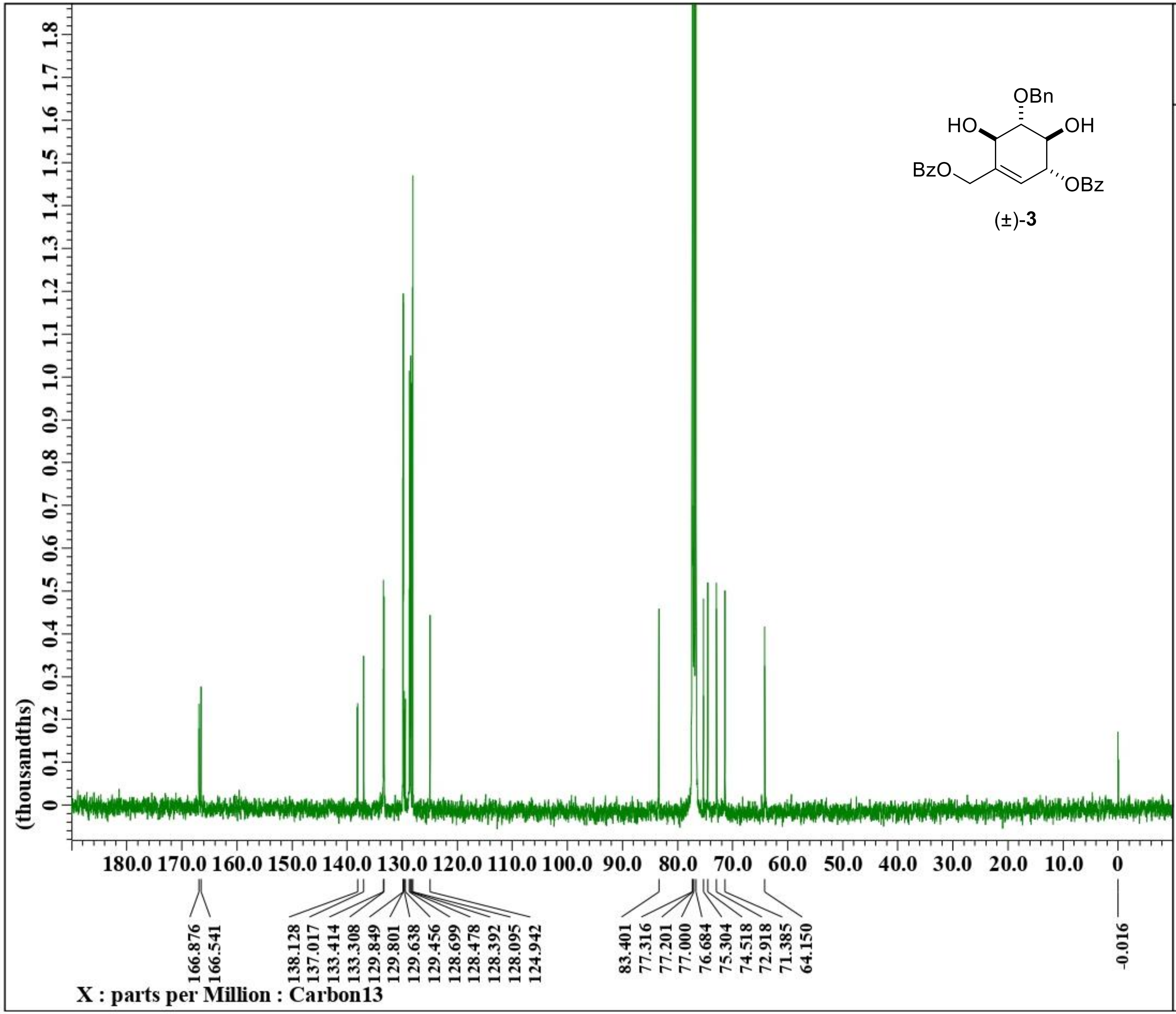

Filename	= 18kus7-93-1-5_C
Author	= delta
Experiment	= carbon_auto.jxp
Sample_Id	= 18kus7-93-1-5
Solvent	= CHLOROFORM-D
Actual_Start_Time	= 27-FEB-2022 21:
Revision_Time	= 6-OCT-2024 15:
Comment	= single pulse de
Data_Format	= 1D COMPLEX
Dim_Size	= 26214
X_Domain	= Carbon13
Dim_Title	= Carbon13
Dim_Units	= [ppm]
Dimensions	= X
Spectrometer	= JNM-ECZ400S/L1
Field_Strength	= 9.389766[T] (40
X_Acq_Duration	= 1.03809024[s]
X_Domain	= Carbon13
X_Freq	= 100.52530333[MH
X_Offset	= 100[ppm]
X_Points	= 32768
X_Prescans	= 4
X_Resolution	= 0.96330739[Hz]
X_Sweep	= 31.56565657[kHz
X_Sweep_Clipped	= 25.25252525[kHz
Irr_Domain	= Proton
Irr_Freq	= 399.78219838[MH
Irr_Offset	= 5[ppm]
Blanking	= 5.0[us]
Clipped	= FALSE
Scans	= 5000
Total_Scans	= 5000
Relaxation_Delay	= 2[s]
Recvr_Gain	= 50
Temp_Get	= 19.6[dC]
X_90_Width	= 10.5[us]
X_Acq_Time	= 1.03809024[s]
X_Angle	= 30[deg]
X_Atn	= 8.2[dB]
X_Pulse	= 3.5[us]
Irr_Atn_Dec	= 31.323[dB]
Irr_Atn_Dec_Calc	= 31.323[dB]
Irr_Atn_Dec_Default_Calc	= 31.323[dB]
Irr_Atn_Noe	= 31.323[dB]
Irr_Dec_Bandwidth_Hz	= 4.7826087[kHz]
Irr_Dec_Bandwidth_Ppm	= 11.96303566[ppm
Irr_Dec_Freq	= 399.78219838[MH
Irr_Dec_Merit_Factor	= 2.2
Irr_Decoupling	= TRUE
Irr_Noe	= TRUE

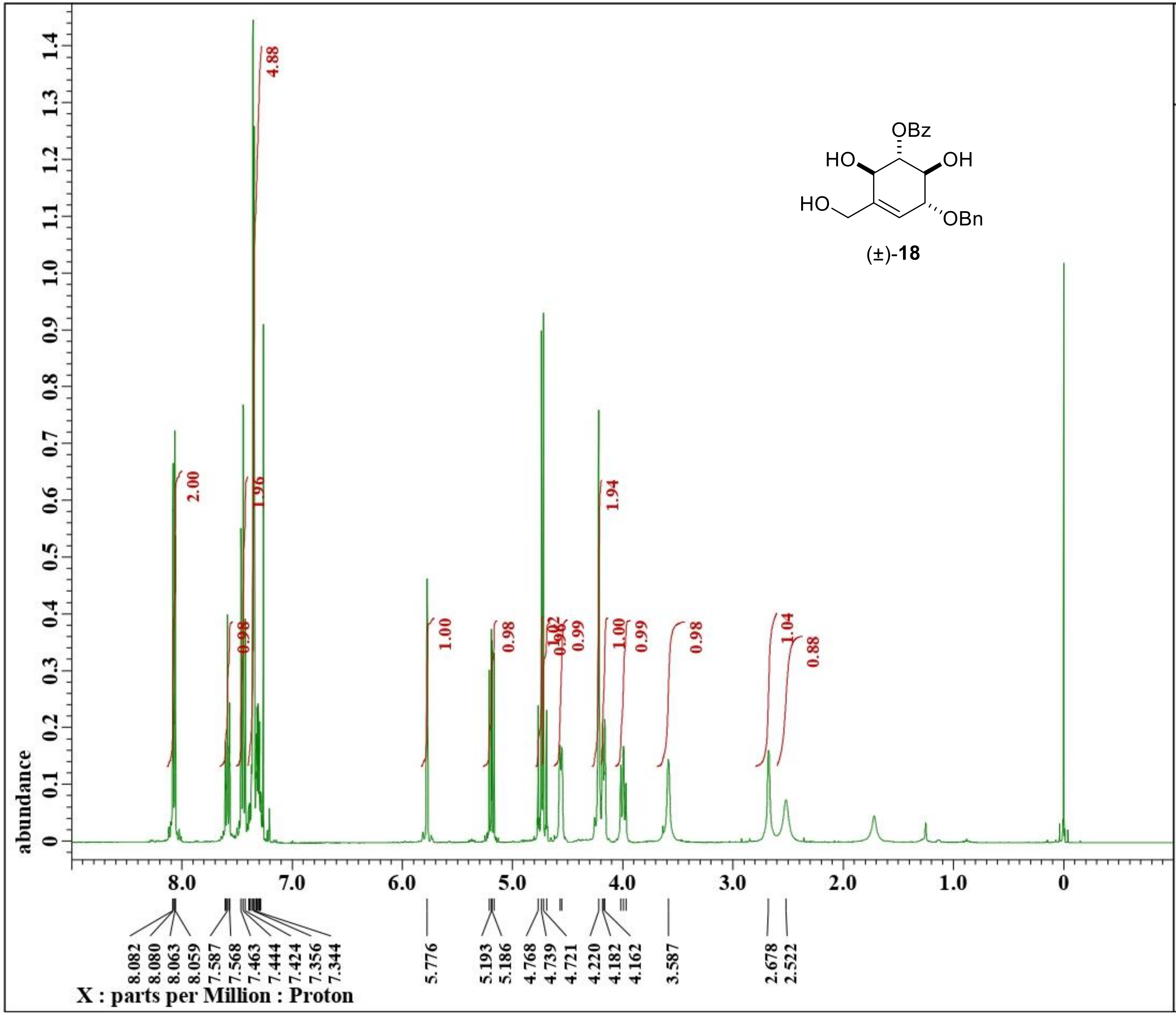

Filename	= 18kus10-71-1-4_Pro
Author	= delta
Experiment	= proton_auto.jxp
Sample_Id	= 18kus10-71-1-4
Solvent	= CHLOROFORM-D
Actual_Start_Time	= 22-DEC-2022 02:02:
Revision_Time	= 6-OCT-2024 18:43:
Comment	= single_pulse
Data_Format	= 1D COMPLEX
Dim_Size	= 13107
X_Domain	= Proton
Dim_Title	= Proton
Dim_Units	= [ppm]
Dimensions	= X
Spectrometer	= JNM-ECZ400S/L1
Field_Strength	= 9.389766[T] (400[M
X_Acq_Duration	= 2.18628096[s]
X_Domain	= Proton
X_Freq	= 399.78219838[MHz]
X_Offset	= 5[ppm]
X_Points	= 16384
X_Prescans	= 1
X_Resolution	= 0.45739775[Hz]
X_Sweep	= 7.4940048[kHz]
X_Sweep_Clipped	= 5.99520384[kHz]
Irr_Domain	= Proton
Irr_Freq	= 399.78219838[MHz]
Irr_Offset	= 5[ppm]
Tri_Domain	= Proton
Tri_Freq	= 399.78219838[MHz]
Tri_Offset	= 5[ppm]
Blanking	= 2.0[us]
Clipped	= FALSE
Scans	= 8
Total_Scans	= 8
Relaxation_Delay	= 5[s]
Recvr_Gain	= 56
Temp_Get	= 17.2[dC]
X_90_Width	= 6.6[us]
X_Acq_Time	= 2.18628096[s]
X_Angle	= 45[deg]
X_Atn	= 6.5[dB]
X_Pulse	= 3.3[us]
Irr_Mode	= Off
Tri_Mode	= Off
Dante_Loop	= 500
Dante_Presat	= FALSE
Decimation_Rate	= 0
Experiment_Path	= C:\Program Files\J
Initial_Wait	= 1[s]

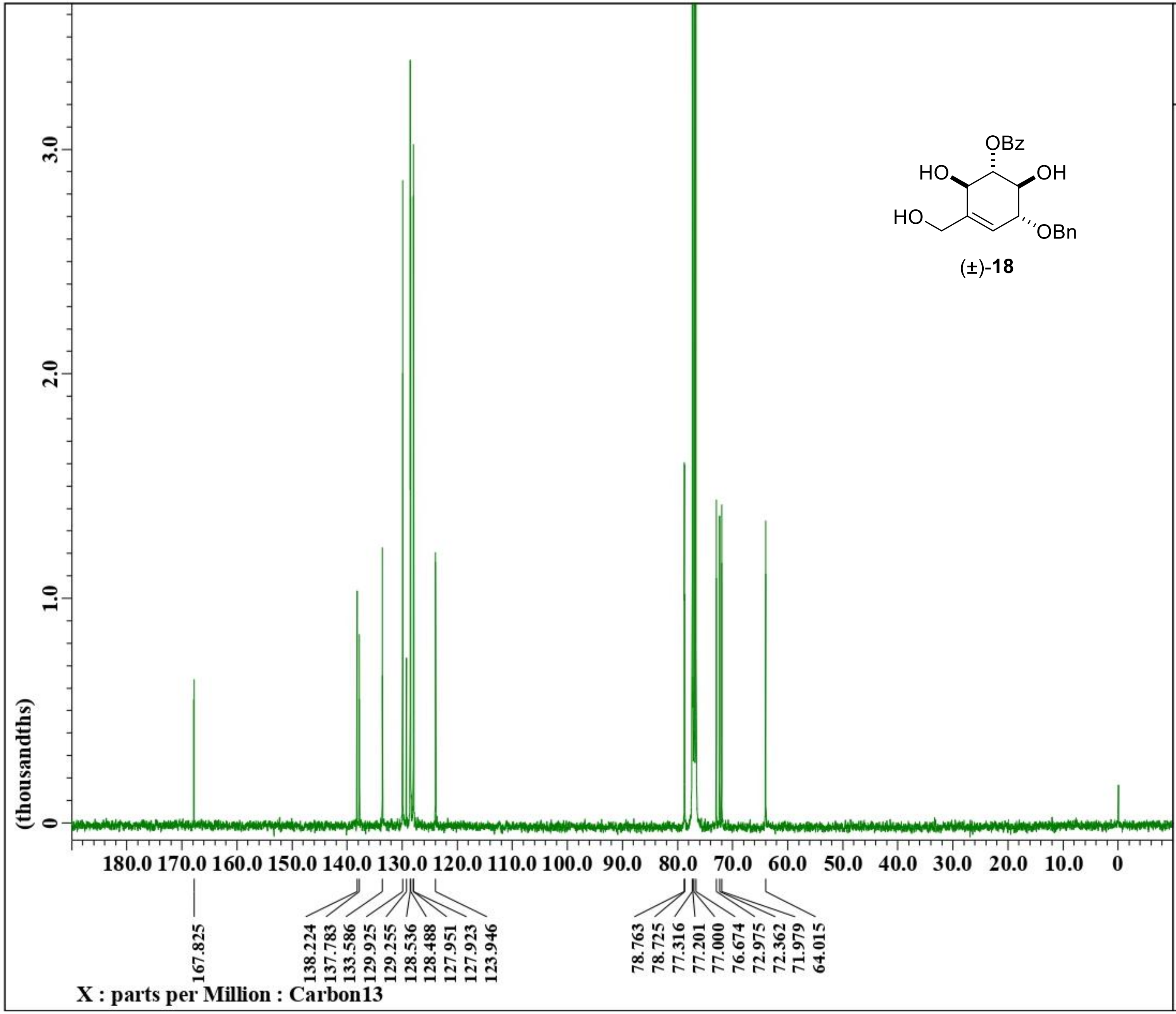

JEOL

```
Filename                    = 18kus10-71-1-4_
Author                      = delta
Experiment                  = carbon_auto.jxp
Sample_Id                   = 18kus10-71-1-4
Solvent                     = CHLOROFORM-D
Actual_Start_Time           = 22-DEC-2022 02:
Revision_Time               =  6-OCT-2024 18:

Comment                     = single pulse de
Data_Format                 = 1D COMPLEX
Dim_Size                    = 26214
X_Domain                    = Carbon13
Dim_Title                   = Carbon13
Dim_Units                   = [ppm]
Dimensions                  = X
Spectrometer                = JNM-ECZ400S/L1

Field_Strength              = 9.389766[T] (40
X_Acq_Duration              = 1.03809024[s]
X_Domain                    = Carbon13
X_Freq                      = 100.52530333[MH
X_Offset                    = 100[ppm]
X_Points                    = 32768
X_Prescans                  = 4
X_Resolution                = 0.96330739[Hz]
X_Sweep                     = 31.56565657[kHz
X_Sweep_Clipped             = 25.25252525[kHz
Irr_Domain                  = Proton
Irr_Freq                    = 399.78219838[MH
Irr_Offset                  = 5[ppm]
Blanking                    = 5.0[us]
Clipped                     = FALSE
Scans                       = 5600
Total_Scans                 = 5600

Relaxation_Delay            = 2[s]
Recvr_Gain                  = 50
Temp_Get                    = 17.1[dC]
X_90_Width                  = 10.5[us]
X_Acq_Time                  = 1.03809024[s]
X_Angle                     = 30[deg]
X_Atn                       = 8.2[dB]
X_Pulse                     = 3.5[us]
Irr_Atn_Dec                 = 31.323[dB]
Irr_Atn_Dec_Calc            = 31.323[dB]
Irr_Atn_Dec_Default_Calc    = 31.323[dB]
Irr_Atn_Noe                 = 31.323[dB]
Irr_Dec_Bandwidth_Hz        = 4.7826087[kHz]
Irr_Dec_Bandwidth_Ppm       = 11.96303566[ppm
Irr_Dec_Freq                = 399.78219838[MH
Irr_Dec_Merit_Factor        = 2.2
Irr_Decoupling              = TRUE
Irr_Noe                     = TRUE
```

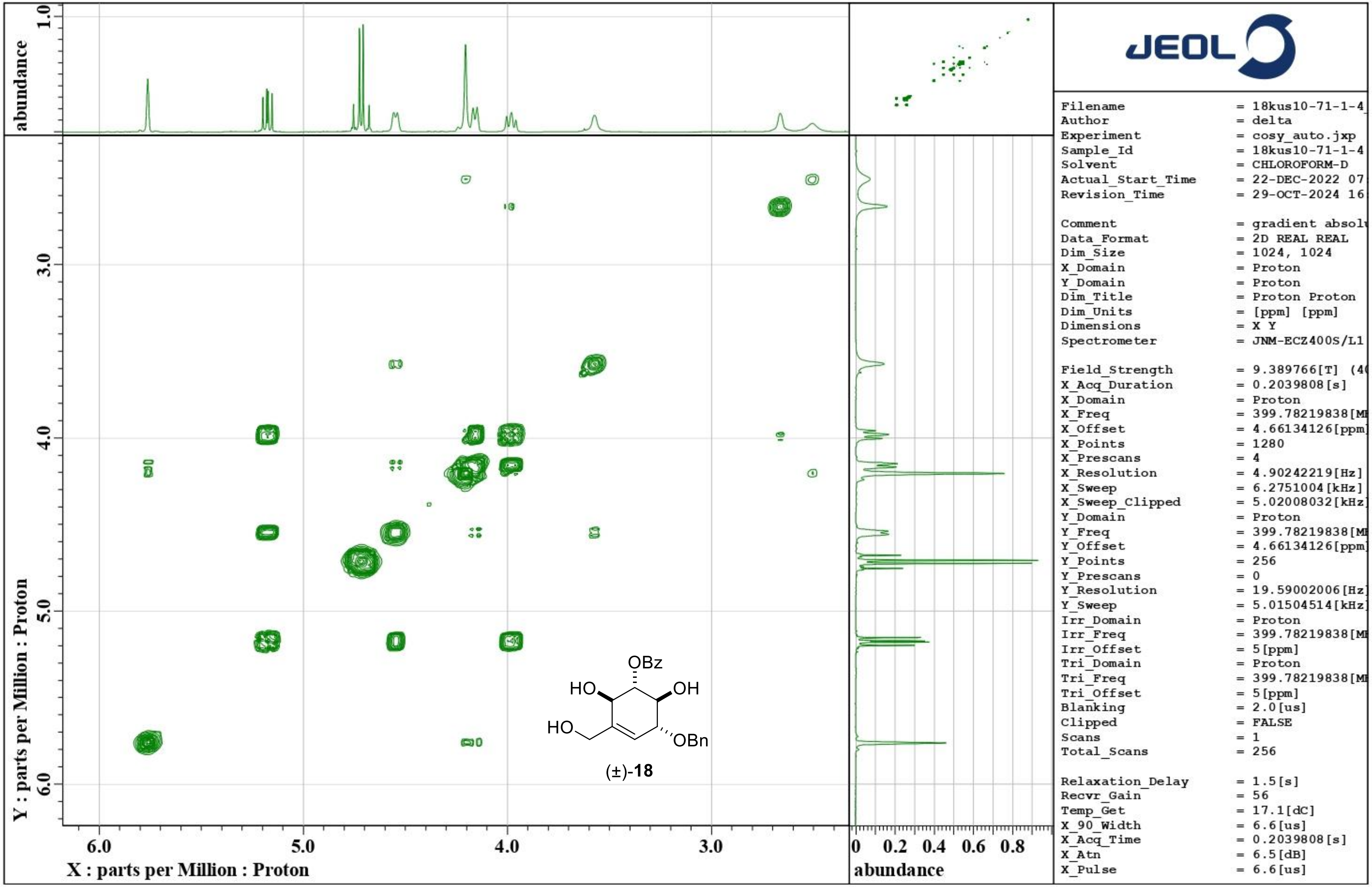

JEOL
abundance
1.0
Y : parts per Million : Proton
3.0
4.0
5.0
6.0
X : parts per Million : Proton
6.0
5.0
4.0
3.0
0 0.2 0.4 0.6 0.8
abundance
OBz
HO
OH
HO
OBn
(±)-18
Filename = 18kus10-71-1-4_
Author = delta
Experiment = cosy_auto.jxp
Sample_Id = 18kus10-71-1-4
Solvent = CHLOROFORM-D
Actual_Start_Time = 22-DEC-2022 07:
Revision_Time = 29-OCT-2024 16:
Comment = gradient absol
Data_Format = 2D REAL REAL
Dim_Size = 1024, 1024
X_Domain = Proton
Y_Domain = Proton
Dim_Title = Proton Proton
Dim_Units = [ppm] [ppm]
Dimensions = X Y
Spectrometer = JNM-ECZ400S/L1
Field_Strength = 9.389766[T] (4
X_Acq_Duration = 0.2039808[s]
X_Domain = Proton
X_Freq = 399.78219838[M
X_Offset = 4.66134126[ppm]
X_Points = 1280
X_Prescans = 4
X_Resolution = 4.90242219[Hz]
X_Sweep = 6.2751004[kHz]
X_Sweep_Clipped = 5.02008032[kHz]
Y_Domain = Proton
Y_Freq = 399.78219838[M
Y_Offset = 4.66134126[ppm]
Y_Points = 256
Y_Prescans = 0
Y_Resolution = 19.59002006[Hz]
Y_Sweep = 5.01504514[kHz]
Irr_Domain = Proton
Irr_Freq = 399.78219838[M
Irr_Offset = 5[ppm]
Tri_Domain = Proton
Tri_Freq = 399.78219838[M
Tri_Offset = 5[ppm]
Blanking = 2.0[us]
Clipped = FALSE
Scans = 1
Total_Scans = 256
Relaxation_Delay = 1.5[s]
Recvr_Gain = 56
Temp_Get = 17.1[dC]
X_90_Width = 6.6[us]
X_Acq_Time = 0.2039808[s]
X_Atn = 6.5[dB]
X_Pulse = 6.6[us]

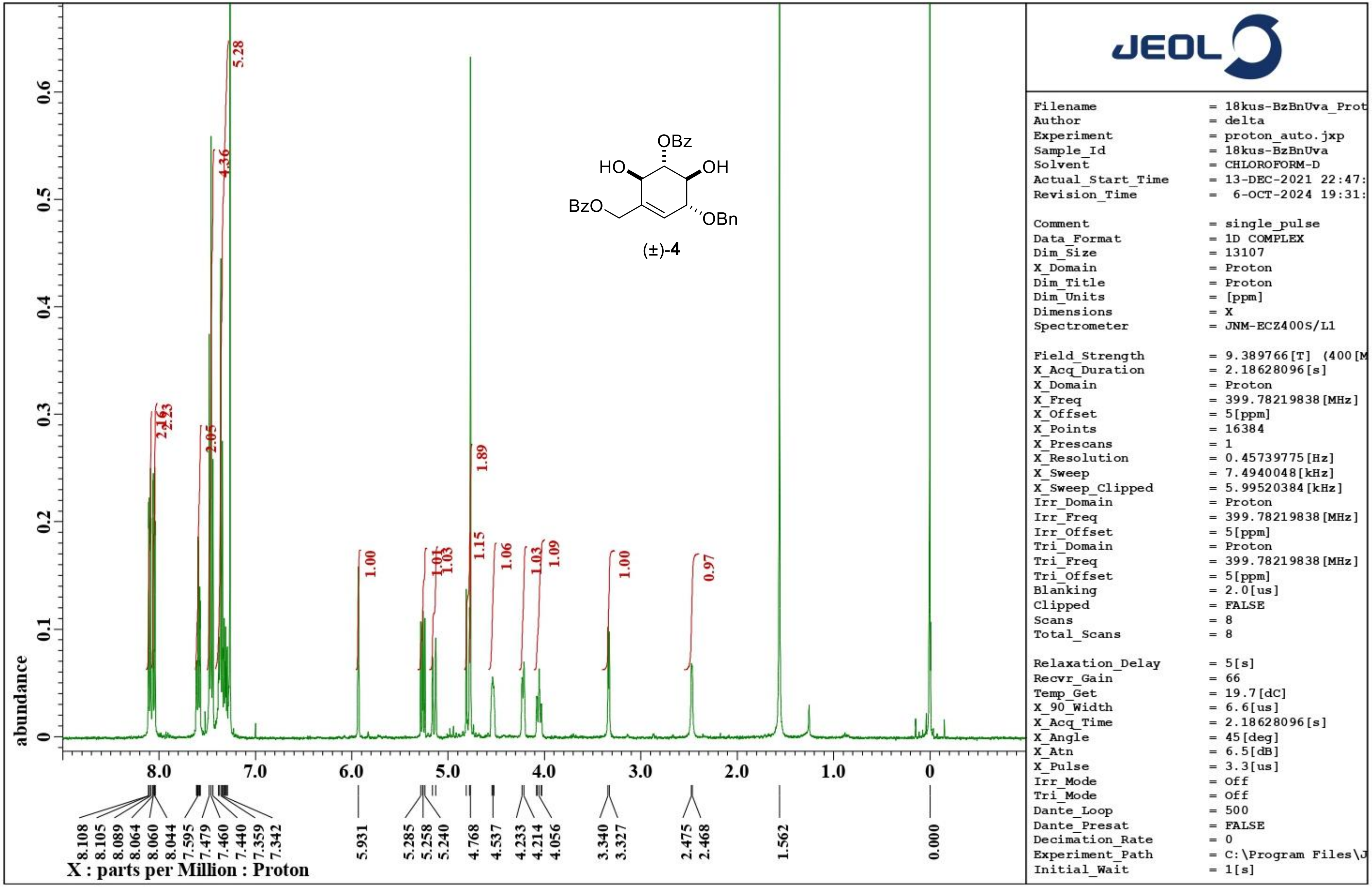

JEOL

Filename	= 18kus-BzBnUva_Prot
Author	= delta
Experiment	= proton_auto.jxp
Sample_Id	= 18kus-BzBnUva
Solvent	= CHLOROFORM-D
Actual_Start_Time	= 13-DEC-2021 22:47:
Revision_Time	= 6-OCT-2024 19:31:
Comment	= single_pulse
Data_Format	= 1D COMPLEX
Dim_Size	= 13107
X_Domain	= Proton
Dim_Title	= Proton
Dim_Units	= [ppm]
Dimensions	= X
Spectrometer	= JNM-ECZ400S/L1
Field_Strength	= 9.389766[T] (400[M
X_Acq_Duration	= 2.18628096[s]
X_Domain	= Proton
X_Freq	= 399.78219838[MHz]
X_Offset	= 5[ppm]
X_Points	= 16384
X_Prescans	= 1
X_Resolution	= 0.45739775[Hz]
X_Sweep	= 7.4940048[kHz]
X_Sweep_Clipped	= 5.99520384[kHz]
Irr_Domain	= Proton
Irr_Freq	= 399.78219838[MHz]
Irr_Offset	= 5[ppm]
Tri_Domain	= Proton
Tri_Freq	= 399.78219838[MHz]
Tri_Offset	= 5[ppm]
Blanking	= 2.0[us]
Clipped	= FALSE
Scans	= 8
Total_Scans	= 8
Relaxation_Delay	= 5[s]
Recvr_Gain	= 66
Temp_Get	= 19.7[dC]
X_90_Width	= 6.6[us]
X_Acq_Time	= 2.18628096[s]
X_Angle	= 45[deg]
X_Atn	= 6.5[dB]
X_Pulse	= 3.3[us]
Irr_Mode	= Off
Tri_Mode	= Off
Dante_Loop	= 500
Dante_Presat	= FALSE
Decimation_Rate	= 0
Experiment_Path	= C:\Program Files\J
Initial_Wait	= 1[s]

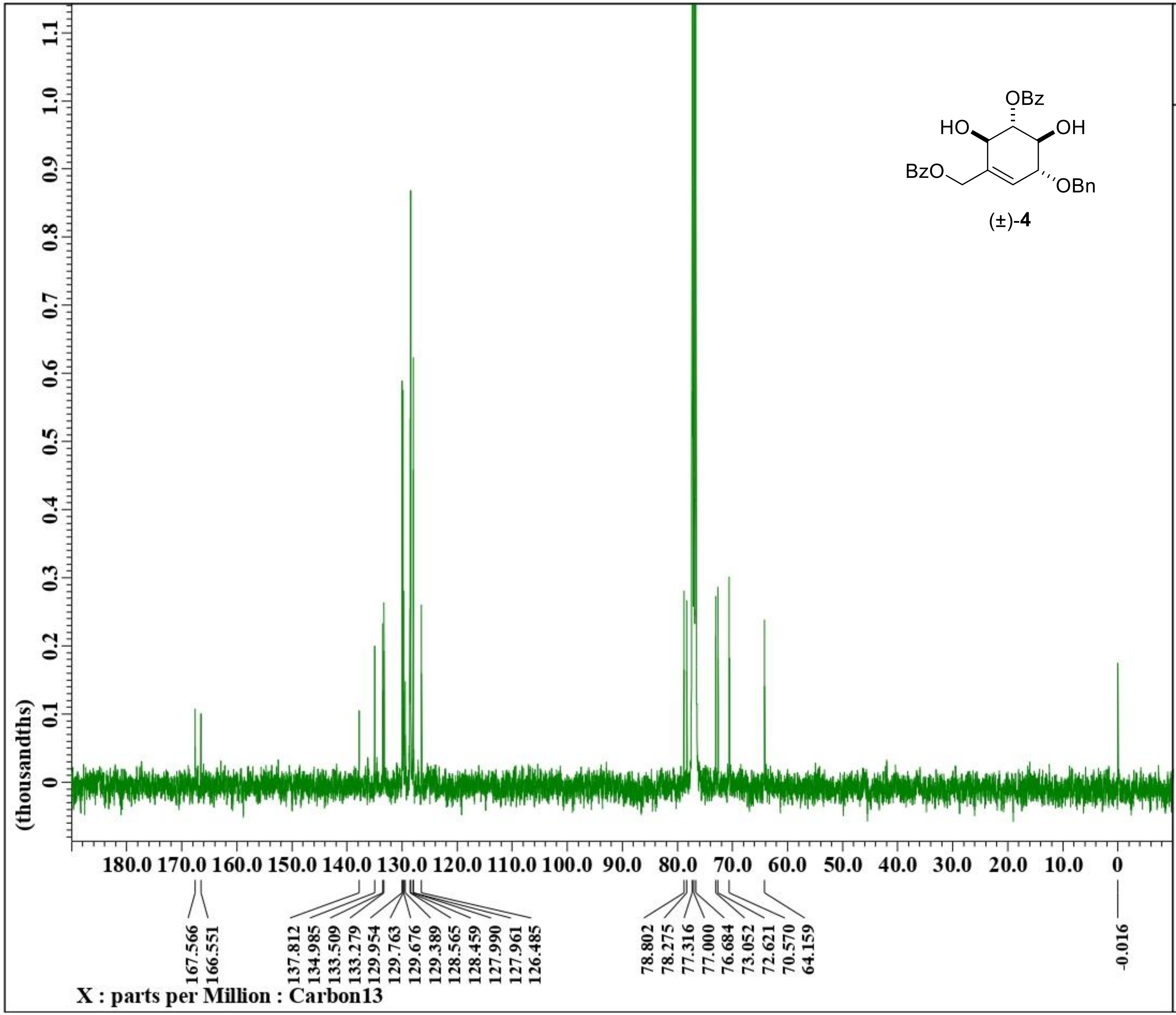

JEOL

Filename	=	18kus7-25-1_Car
Author	=	delta
Experiment	=	carbon_auto.jxp
Sample_Id	=	18kus7-25-1
Solvent	=	CHLOROFORM-D
Actual_Start_Time	=	8-DEC-2024 06:
Revision_Time	=	10-DEC-2024 23:
Comment	=	single pulse de
Data_Format	=	1D COMPLEX
Dim_Size	=	26214
X_Domain	=	Carbon13
Dim_Title	=	Carbon13
Dim_Units	=	[ppm]
Dimensions	=	X
Spectrometer	=	JNM-ECZ400S/L1
Field_Strength	=	9.389766[T] (40
X_Acq_Duration	=	1.03809024[s]
X_Domain	=	Carbon13
X_Freq	=	100.52530333[MH
X_Offset	=	100[ppm]
X_Points	=	32768
X_Prescans	=	4
X_Resolution	=	0.96330739[Hz]
X_Sweep	=	31.56565657[kHz
X_Sweep_Clipped	=	25.25252525[kHz
Irr_Domain	=	Proton
Irr_Freq	=	399.78219838[MH
Irr_Offset	=	5[ppm]
Blanking	=	5.0[us]
Clipped	=	FALSE
Scans	=	4096
Total_Scans	=	4096
Relaxation_Delay	=	2[s]
Recvr_Gain	=	50
Temp_Get	=	20.3[dC]
X_90_Width	=	10.5[us]
X_Acq_Time	=	1.03809024[s]
X_Angle	=	30[deg]
X_Atn	=	8.2[dB]
X_Pulse	=	3.5[us]
Irr_Atn_Dec	=	31.323[dB]
Irr_Atn_Dec_Calc	=	31.323[dB]
Irr_Atn_Dec_Default_Calc	=	31.323[dB]
Irr_Atn_Noe	=	31.323[dB]
Irr_Dec_Bandwidth_Hz	=	4.7826087[kHz]
Irr_Dec_Bandwidth_Ppm	=	11.96303566[ppm
Irr_Dec_Freq	=	399.78219838[MH
Irr_Dec_Merit_Factor	=	2.2
Irr_Decoupling	=	TRUE
Irr_Noe	=	TRUE

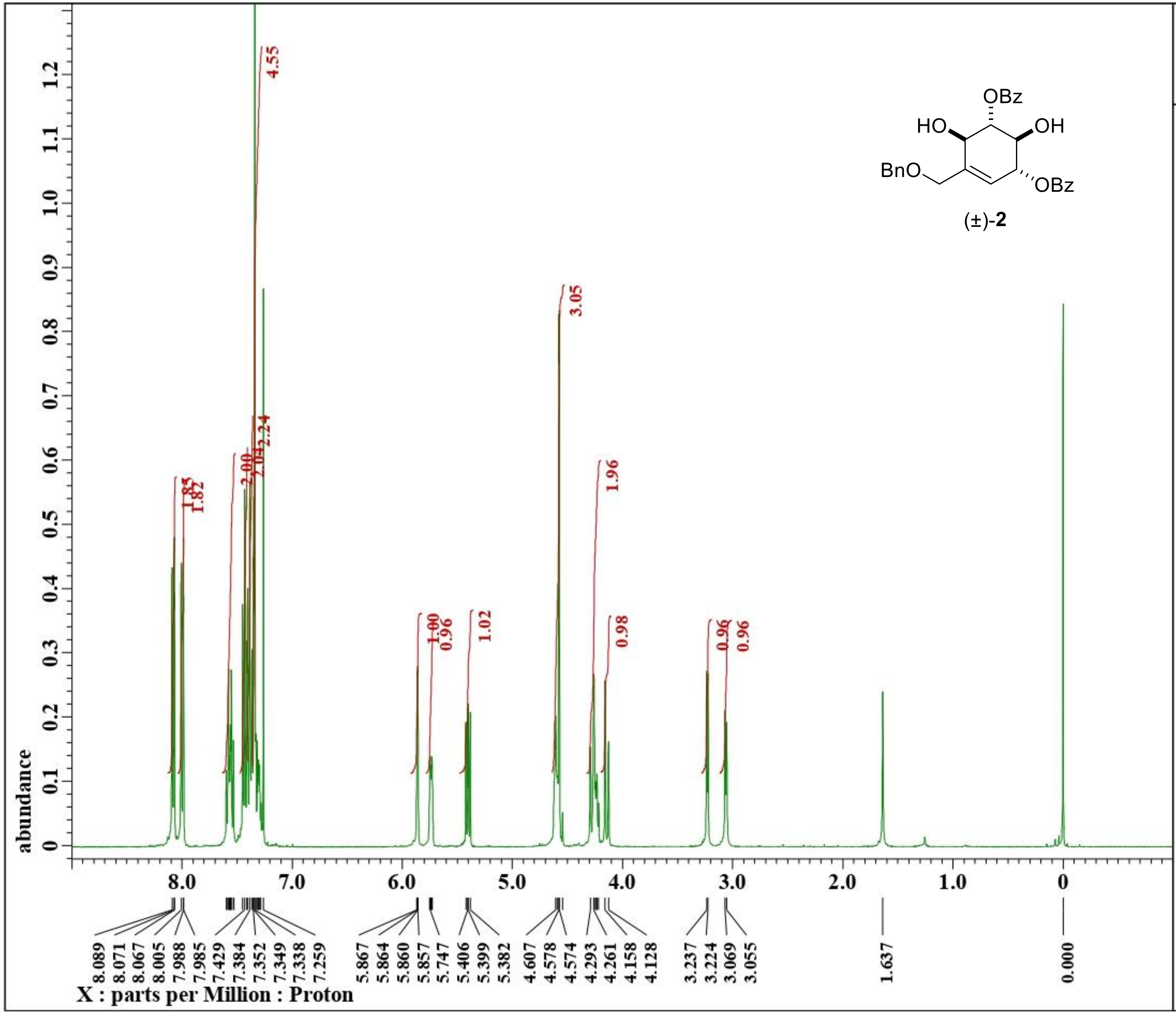

Filename	= 18kus7-75-BnUva_Pr
Author	= delta
Experiment	= proton_auto.jxp
Sample_Id	= 18kus7-75-BnUva
Solvent	= CHLOROFORM-D
Actual_Start_Time	= 13-FEB-2022 21:02:
Revision_Time	= 7-OCT-2024 13:57:
Comment	= single_pulse
Data_Format	= 1D COMPLEX
Dim_Size	= 13107
X_Domain	= Proton
Dim_Title	= Proton
Dim_Units	= [ppm]
Dimensions	= X
Spectrometer	= JNM-ECZ400S/L1
Field_Strength	= 9.389766[T] (400[M
X_Acq_Duration	= 2.18628096[s]
X_Domain	= Proton
X_Freq	= 399.78219838[MHz]
X_Offset	= 5[ppm]
X_Points	= 16384
X_Prescans	= 1
X_Resolution	= 0.45739775[Hz]
X_Sweep	= 7.4940048[kHz]
X_Sweep_Clipped	= 5.99520384[kHz]
Irr_Domain	= Proton
Irr_Freq	= 399.78219838[MHz]
Irr_Offset	= 5[ppm]
Tri_Domain	= Proton
Tri_Freq	= 399.78219838[MHz]
Tri_Offset	= 5[ppm]
Blanking	= 2.0[us]
Clipped	= FALSE
Scans	= 8
Total_Scans	= 8
Relaxation_Delay	= 5[s]
Recvr_Gain	= 56
Temp_Get	= 19.8[dC]
X_90_Width	= 6.6[us]
X_Acq_Time	= 2.18628096[s]
X_Angle	= 45[deg]
X_Atn	= 6.5[dB]
X_Pulse	= 3.3[us]
Irr_Mode	= Off
Tri_Mode	= Off
Dante_Loop	= 500
Dante_Presat	= FALSE
Decimation_Rate	= 0
Experiment_Path	= C:\Program Files\J
Initial_Wait	= 1[s]

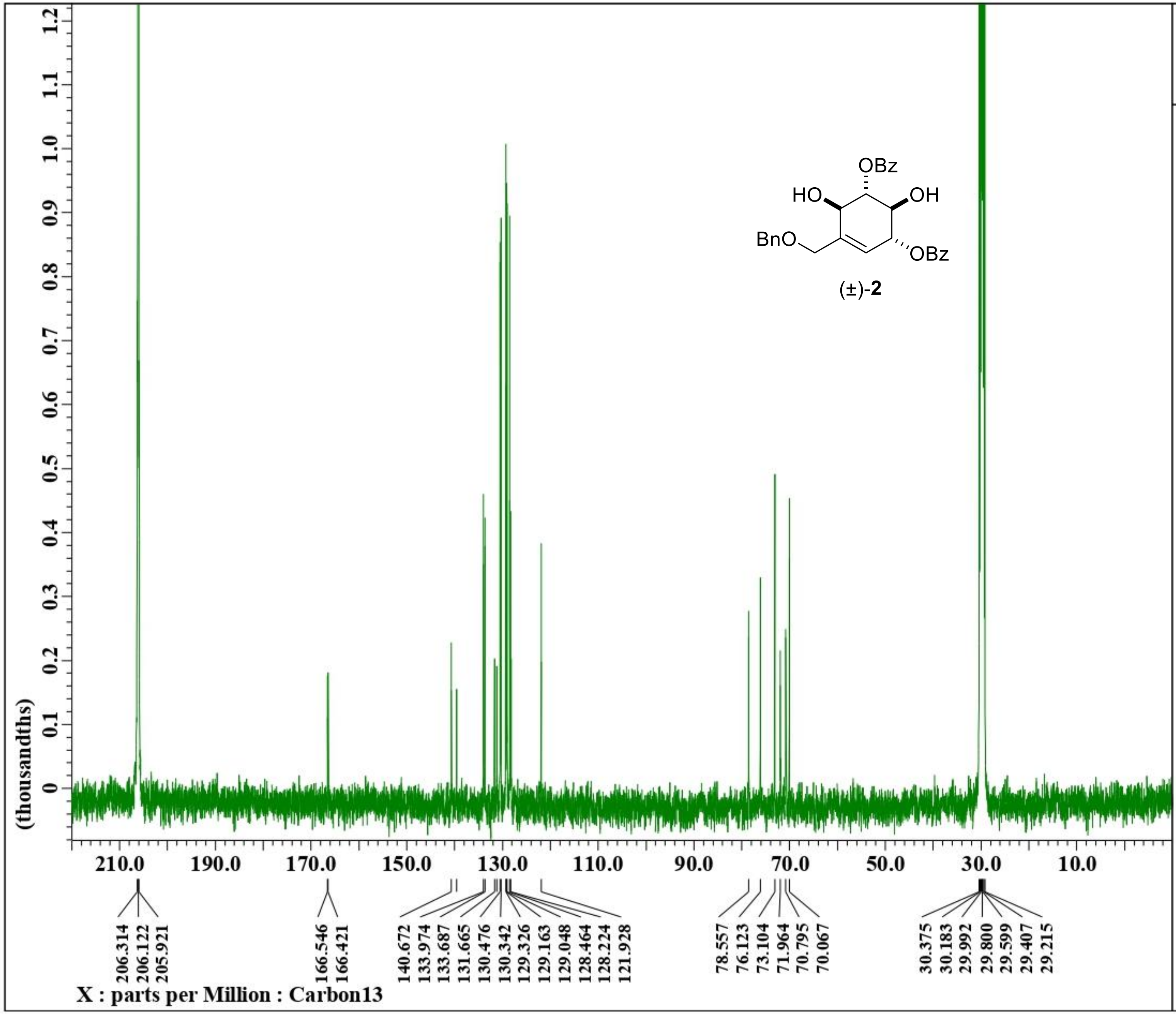

JEOL

Filename	= 18kus7-75-bnuva
Author	= delta
Experiment	= carbon_auto.jxp
Sample_Id	= 18kus7-75-bnuva
Solvent	= ACETONE-D6
Actual_Start_Time	= 8-OCT-2024 00:
Revision_Time	= 8-OCT-2024 13:
Comment	= single pulse de
Data_Format	= 1D COMPLEX
Dim_Size	= 26214
X_Domain	= Carbon13
Dim_Title	= Carbon13
Dim_Units	= [ppm]
Dimensions	= X
Spectrometer	= JNM-ECZ400S/L1
Field_Strength	= 9.389766[T] (40
X_Acq_Duration	= 1.03809024[s]
X_Domain	= Carbon13
X_Freq	= 100.52530333[MH
X_Offset	= 100[ppm]
X_Points	= 32768
X_Prescans	= 4
X_Resolution	= 0.96330739[Hz]
X_Sweep	= 31.56565657[kHz
X_Sweep_Clipped	= 25.25252525[kHz
Irr_Domain	= Proton
Irr_Freq	= 399.78219838[MH
Irr_Offset	= 5[ppm]
Blanking	= 5.0[us]
Clipped	= FALSE
Scans	= 3072
Total_Scans	= 3072
Relaxation_Delay	= 2[s]
Recvr_Gain	= 50
Temp_Get	= 24.3[dC]
X_90_Width	= 10.5[us]
X_Acq_Time	= 1.03809024[s]
X_Angle	= 30[deg]
X_Atn	= 8.2[dB]
X_Pulse	= 3.5[us]
Irr_Atn_Dec	= 31.323[dB]
Irr_Atn_Dec_Calc	= 31.323[dB]
Irr_Atn_Dec_Default_Calc	= 31.323[dB]
Irr_Atn_Noe	= 31.323[dB]
Irr_Dec_Bandwidth_Hz	= 4.7826087[kHz]
Irr_Dec_Bandwidth_Ppm	= 11.96303566[ppm
Irr_Dec_Freq	= 399.78219838[MH
Irr_Dec_Merit_Factor	= 2.2
Irr_Decoupling	= TRUE
Irr_Noe	= TRUE

Cartesian coordinates and total energies for all of the calculated structures (B3LYP/6-31G** [SMD = water])

H^+

Zero-point correction= 0.000000 (Hartree/Particle)
Thermal correction to Energy= 0.001416
Thermal correction to Enthalpy= 0.002360
Thermal correction to Gibbs Free Energy= -0.010000
Sum of electronic and thermal Free Energies= -0.225180

H	0.00000	0.00000	0.00000

SM-*E*

Zero-point correction= 0.418553 (Hartree/Particle)
Thermal correction to Energy= 0.443518
Thermal correction to Enthalpy= 0.444462
Thermal correction to Gibbs Free Energy= 0.359890
Sum of electronic and thermal Free Energies= -1415.904622

C	2.56005	-1.28933	0.18131
H	2.44709	-2.27143	0.63827
C	4.24995	0.09924	-0.72050
H	5.31530	0.14203	-0.94396
C	2.12028	0.20823	-1.75065
H	1.66065	0.33508	-2.73241
C	1.79791	1.39581	-0.82770
H	2.15913	2.31486	-1.29117
C	2.16445	-0.16477	1.09825
C	2.53558	1.16529	0.50931
H	2.38523	1.99923	1.19584
C	1.50857	-0.28882	2.25675
O	0.37674	1.48380	-0.63271
O	0.39921	-1.33178	-1.01009
O	3.55732	0.25294	-1.94582
O	3.95501	1.15636	0.16590
O	3.98106	-1.15443	-0.13853
C	-0.13194	2.69834	-0.31527
C	-0.02916	-2.70951	-0.95275
H	0.21578	-3.20529	-1.90064

H	0.50164	-3.2306	-0.14701
C	-1.60371	2.66778	-0.12607
C	-2.26022	3.87768	0.14645
C	-2.33902	1.47486	-0.20350
C	-3.63946	3.89485	0.33549
H	-1.68261	4.79358	0.20633
C	-3.71879	1.49798	-0.01014
H	-1.83318	0.53905	-0.40853
C	-4.36975	2.70522	0.25786
H	-4.14504	4.83237	0.54437
H	-4.28374	0.57283	-0.06634
H	-5.44511	2.71926	0.40775
C	-1.51355	-2.73430	-0.70009
C	-2.01139	-2.45336	0.58098
C	-2.41462	-3.00805	-1.73584
C	-3.38593	-2.44852	0.82049
H	-1.31508	-2.24171	1.38855
C	-3.79113	-3.00897	-1.49745
H	-2.03503	-3.22457	-2.73087
C	-4.27888	-2.72799	-0.21941
H	-3.76110	-2.23493	1.81703
H	-4.48021	-3.22804	-2.30775
H	-5.34855	-2.73164	-0.03147
O	0.56062	3.69984	-0.20186
C	1.80075	-1.18048	-1.16300
H	2.19778	-1.93227	-1.85718
O	1.09226	-1.49670	2.72933
C	0.63079	-1.48771	4.09072
H	-0.18486	-0.76858	4.21863
H	0.26744	-2.49438	4.29871
H	1.44929	-1.24344	4.77549
H	1.25285	0.57774	2.86514

H-IM1A-*E*

Zero-point correction= 0.427778 (Hartree/Particle)

Thermal correction to Energy= 0.454513

Thermal correction to Enthalpy= 0.455457

Thermal correction to Gibbs Free Energy= 0.366279

Sum of electronic and thermal Free Energies= -1416.325037

C	2.71574	-0.90223	-0.27293
H	2.91806	-1.93951	-0.02288
C	4.07291	0.48925	-1.72396
H	5.04503	0.80991	-2.09221
C	1.68699	0.74642	-1.77180
H	1.05573	0.95842	-2.63260
C	1.37397	1.71145	-0.61335
H	1.59402	2.73087	-0.93471
C	2.39954	-0.0553	0.90708
C	2.18332	1.41901	0.67048
H	1.64640	1.85905	1.51802
C	2.29768	-0.57298	2.14751
O	-0.03326	1.57723	-0.37116
O	0.40692	-1.12820	-1.02239
O	3.05732	1.08481	-2.23300
O	3.43634	2.10521	0.46548
O	4.05596	-0.44936	-0.86847
C	-0.69462	2.68287	0.06444
C	0.18809	-2.56304	-1.04345
H	0.47390	-2.94349	-2.03192
H	0.81492	-3.04710	-0.28714
C	-2.14427	2.43747	0.25134
C	-2.93754	3.49995	0.71118
C	-2.73038	1.19068	-0.01787
C	-4.30451	3.31693	0.90002
H	-2.47413	4.45884	0.91604
C	-4.09898	1.01412	0.17387
H	-2.12058	0.36855	-0.37300
C	-4.88613	2.07417	0.63193
H	-4.91607	4.14001	1.25576
H	-4.54883	0.04830	-0.03387
H	-5.95236	1.93224	0.78045
C	-1.26858	-2.81347	-0.76283
C	-1.68465	-3.27808	0.49059
C	-2.23109	-2.55679	-1.75018
C	-3.03978	-3.49012	0.75406
H	-0.94292	-3.47513	1.25989
C	-3.58584	-2.76274	-1.48770
H	-1.91418	-2.19958	-2.72637

C	-3.99217	-3.23163	-0.23410
H	-3.35079	-3.85529	1.72831
H	-4.32295	-2.56705	-2.26076
H	-5.04594	-3.39917	-0.03186
O	-0.13245	3.74670	0.27170
C	1.70171	-0.74182	-1.41557
H	2.03800	-1.31090	-2.29235
O	2.50991	-1.87440	2.41229
C	2.38969	-2.25703	3.79784
H	1.38322	-2.04162	4.16748
H	2.57465	-3.33032	3.83120
H	3.13405	-1.73321	4.40412
H	2.01617	0.05557	2.99152
H	4.02215	1.84200	1.19172

H-IM1B-*E*

Zero-point correction= 0.427391 (Hartree/Particle)

Thermal correction to Energy= 0.454228

Thermal correction to Enthalpy= 0.455172

Thermal correction to Gibbs Free Energy= 0.366000

Sum of electronic and thermal Free Energies= -1416.324134

C	2.32114	-1.40047	0.27636
H	1.83709	-2.10048	0.96425
C	4.08874	0.35610	-1.72048
H	5.08904	0.24557	-2.13453
C	1.74236	-0.13949	-1.89538
H	1.13599	-0.16996	-2.79830
C	1.61168	1.18886	-1.15206
H	1.86638	2.01785	-1.81577
C	2.39838	-0.03170	0.91562
C	2.58110	1.13799	0.03042
H	2.65679	2.08751	0.55405
C	2.36343	0.17313	2.24929
O	0.26784	1.32368	-0.70063
O	0.12344	-1.48357	-0.80475
O	3.14121	-0.18874	-2.39310
O	3.97101	1.03109	-0.65133
O	3.63902	-1.87921	-0.07178

C	-0.17923	2.59577	-0.48097
C	-0.38100	-2.83821	-0.75363
H	-0.17274	-3.32539	-1.71515
H	0.13184	-3.40008	0.03535
C	-1.60915	2.63312	-0.10174
C	-2.18190	3.88354	0.17940
C	-2.39212	1.47008	-0.02918
C	-3.52605	3.96907	0.53026
H	-1.56793	4.77546	0.11962
C	-3.73710	1.56353	0.32145
H	-1.95273	0.50400	-0.24618
C	-4.30448	2.80977	0.60090
H	-3.96761	4.93643	0.74738
H	-4.34100	0.66313	0.37339
H	-5.35335	2.87829	0.87316
C	-1.86076	-2.77990	-0.48421
C	-2.37592	-3.13824	0.76721
C	-2.74122	-2.33852	-1.48298
C	-3.74823	-3.06412	1.01756
H	-1.69907	-3.47885	1.54614
C	-4.11143	-2.25701	-1.23348
H	-2.34877	-2.06231	-2.45800
C	-4.61774	-2.62140	0.01852
H	-4.13650	-3.35002	1.99060
H	-4.78469	-1.91775	-2.01512
H	-5.68513	-2.56541	0.21087
O	0.54695	3.56870	-0.59658
C	1.51881	-1.40737	-1.04551
H	1.85888	-2.26331	-1.64080
O	2.18960	-0.83033	3.12283
C	2.07652	-0.44154	4.50826
H	1.18824	0.17836	4.65777
H	1.98199	-1.36774	5.07418
H	2.97222	0.09920	4.82627
H	2.46265	1.17306	2.66956
H	4.18079	-1.82835	0.73027

H-IM1C-*E* (Hartree/Particle)

Zero-point correction= 0.427601

Thermal correction to Energy= 0.454246
Thermal correction to Enthalpy= 0.455190
Thermal correction to Gibbs Free Energy= 0.367404
Sum of electronic and thermal Free Energies= -1416.326584

C	2.41751	-1.31162	0.22132
H	2.29261	-2.29615	0.66382
C	4.48782	-0.07900	0.04864
H	5.56329	-0.06878	-0.11822
C	1.97505	0.15301	-1.84569
H	1.22080	0.25601	-2.63390
C	1.81221	1.34939	-0.88114
H	2.23402	2.24873	-1.33501
C	2.02914	-0.18404	1.10406
C	2.44094	1.10615	0.50877
H	2.31567	1.97074	1.15712
C	1.41571	-0.28158	2.29623
O	0.40319	1.52506	-0.66211
O	0.37095	-1.41817	-1.01007
O	3.29394	0.14089	-2.38916
O	3.93919	1.05132	0.28310
O	3.91870	-1.22095	0.01851
C	-0.05397	2.77555	-0.39042
C	-0.06425	-2.79654	-1.05496
H	0.13439	-3.20819	-2.05163
H	0.49753	-3.38672	-0.32056
C	-1.51798	2.79580	-0.15913
C	-2.13589	4.03534	0.06583
C	-2.28281	1.61835	-0.14756
C	-3.50753	4.09668	0.29632
H	-1.53511	4.93826	0.05706
C	-3.65412	1.68651	0.08748
H	-1.80668	0.65970	-0.31581
C	-4.26723	2.92298	0.30807
H	-3.98453	5.05635	0.46816
H	-4.24138	0.77355	0.10056
H	-5.33644	2.97243	0.49071
C	-1.53703	-2.82271	-0.74050
C	-1.98526	-2.47073	0.54183
C	-2.47522	-3.17190	-1.71806

C	-3.34867	-2.46494	0.83693
H	-1.26053	-2.20357	1.30660
C	-3.84076	-3.17615	-1.42144
H	-2.13459	-3.44172	-2.71405
C	-4.27976	-2.81938	-0.14520
H	-3.68531	-2.19302	1.83302
H	-4.55898	-3.45292	-2.18750
H	-5.34080	-2.82081	0.08635
O	0.67905	3.75104	-0.34630
C	1.76507	-1.22446	-1.18027
H	2.19973	-1.98507	-1.83839
O	1.02397	-1.46143	2.80041
C	0.42951	-1.41533	4.11589
H	-0.45275	-0.76965	4.11154
H	0.14055	-2.43906	4.35042
H	1.15783	-1.05611	4.84789
H	1.18978	0.60756	2.88296
H	3.42414	0.96793	-2.87820

SM-*Z*

Zero-point correction= 0.418441 (Hartree/Particle)
Thermal correction to Energy= 0.443439
Thermal correction to Enthalpy= 0.444383
Thermal correction to Gibbs Free Energy= 0.359252
Sum of electronic and thermal Free Energies= -1415.904407

C	2.36969	-1.64902	0.58302
H	2.16771	-2.40909	1.33907
C	4.09934	-0.92835	-0.86010
H	5.12244	-1.14058	-1.16839
C	1.88875	-0.77826	-1.69328
H	1.33317	-0.87987	-2.62696
C	1.84798	0.67108	-1.18126
H	2.25131	1.33447	-1.94768
C	2.26784	-0.24813	1.11390
C	2.72406	0.74944	0.08568
H	2.78673	1.76734	0.46756
C	1.81962	0.03836	2.34098
H	1.51973	-0.74549	3.03493

O	0.48670	1.03800	-0.89583
O	0.11677	-1.76685	-0.23224
O	3.28074	-1.04475	-2.00873
O	4.07047	0.38673	-0.35024
O	3.73972	-1.87343	0.12094
O	1.68521	1.31774	2.78607
C	0.18115	2.35768	-0.92585
C	-0.82215	-2.31704	-1.17511
H	-0.92256	-1.65946	-2.04642
H	-0.44200	-3.28714	-1.52641
C	-1.24125	2.62050	-0.59470
C	-1.71085	3.93894	-0.69464
C	-2.11067	1.59710	-0.18699
C	-3.03909	4.22992	-0.39536
H	-1.03033	4.72273	-1.00874
C	-3.43855	1.89474	0.11241
H	-1.74719	0.58055	-0.09743
C	-3.90417	3.20825	0.00766
H	-3.40036	5.25026	-0.47588
H	-4.10887	1.10029	0.42522
H	-4.93993	3.43612	0.24074
C	-2.15576	-2.48978	-0.49206
C	-2.25608	-3.25106	0.68189
C	-3.31296	-1.91154	-1.02640
C	-3.48953	-3.42913	1.30736
H	-1.36275	-3.70121	1.10535
C	-4.55220	-2.09586	-0.40551
H	-3.24389	-1.31624	-1.93274
C	-4.64221	-2.85326	0.76349
H	-3.55381	-4.02009	2.21635
H	-5.44273	-1.64553	-0.83405
H	-5.60317	-2.99521	1.24912
O	0.99974	3.22280	-1.20278
C	1.35630	1.44227	4.17896
H	1.22992	2.50826	4.37066
H	0.42328	0.91598	4.40612
H	2.16339	1.05005	4.80635
C	1.46880	-1.84330	-0.65184
H	1.67678	-2.82928	-1.08772

H-IM1A-*Z* (Hartree/Particle)

Zero-point correction= 0.427374

Thermal correction to Energy= 0.454302

Thermal correction to Enthalpy= 0.455246

Thermal correction to Gibbs Free Energy= 0.366350

Sum of electronic and thermal Free Energies= -1416.325401

C	2.57695	-1.37545	-0.14232
H	2.66697	-2.37311	0.28016
C	4.05863	-0.42626	-1.83431
H	5.04912	-0.28251	-2.26200
C	1.71526	0.10274	-1.90145
H	1.08969	0.23806	-2.78174
C	1.54312	1.27838	-0.92396
H	1.87093	2.19519	-1.41671
C	2.42372	-0.30798	0.86937
C	2.34302	1.12234	0.38726
H	1.86589	1.74690	1.14827
C	2.41545	-0.65053	2.17483
H	2.51641	-1.68888	2.48783
O	0.13616	1.35451	-0.65389
O	0.24595	-1.46201	-0.84202
O	3.10275	0.19920	-2.42147
O	3.65527	1.63485	0.07417
O	3.95862	-1.20537	-0.83849
O	2.26716	0.25831	3.15107
C	-0.38282	2.59139	-0.42628
C	-0.14796	-2.85084	-0.69464
H	0.04967	-3.36834	-1.64213
H	0.44661	-3.32737	0.09203
C	-1.84337	2.55513	-0.17708
C	-2.49894	3.77042	0.07419
C	-2.57169	1.35525	-0.18720
C	-3.87039	3.78510	0.31228
H	-1.92690	4.69169	0.08036
C	-3.94389	1.37694	0.05255
H	-2.06819	0.41554	-0.38012
C	-4.59376	2.58876	0.30205
H	-4.37538	4.72608	0.50620

H	-4.50390	0.44712	0.04485
H	-5.66332	2.60134	0.48880
C	-1.61205	-2.88968	-0.34932
C	-2.03579	-3.26828	0.92993
C	-2.57150	-2.53177	-1.30815
C	-3.39593	-3.29617	1.24746
H	-1.29715	-3.54473	1.67743
C	-3.92999	-2.55191	-0.99113
H	-2.25015	-2.24172	-2.30486
C	-4.34460	-2.93592	0.28853
H	-3.71275	-3.59616	2.24188
H	-4.66499	-2.27820	-1.74219
H	-5.40242	-2.95843	0.53323
O	0.30097	3.60278	-0.43185
C	2.17772	-0.27064	4.49077
H	2.11064	0.59288	5.15178
H	1.28291	-0.89031	4.59582
H	3.07119	-0.85391	4.73027
C	1.56801	-1.29443	-1.29577
H	1.81971	-2.03812	-2.06266
H	4.21538	1.46920	0.84758

H-IM1B-*Z* (Hartree/Particle)
Zero-point correction= 0.427967
Thermal correction to Energy= 0.454524
Thermal correction to Enthalpy= 0.455468
Thermal correction to Gibbs Free Energy= 0.367906
Sum of electronic and thermal Free Energies= -1416.321977

C	2.14237	-1.67409	0.80311
H	1.62018	-2.08624	1.67355
C	3.99948	-0.84720	-1.63498
H	4.95918	-1.20307	-2.00291
C	1.60484	-1.05422	-1.63572
H	0.96126	-1.26340	-2.48808
C	1.67890	0.43962	-1.32367
H	1.99711	0.98984	-2.21197
C	2.39461	-0.20012	1.00635
C	2.70178	0.61426	-0.19787

H	2.91601	1.65742	0.01535
C	2.35185	0.33923	2.24140
H	2.08085	-0.27057	3.10250
O	0.39337	0.88652	-0.90602
O	-0.08707	-1.82476	-0.07835
O	2.96302	-1.42792	-2.11569
O	4.01778	0.12103	-0.80961
O	3.37982	-2.38503	0.57875
O	2.61536	1.63483	2.48211
C	0.11908	2.21377	-1.08266
C	-0.99221	-2.69336	-0.79793
H	-1.00270	-2.43521	-1.86288
H	-0.63513	-3.72680	-0.69764
C	-1.25232	2.56633	-0.65321
C	-1.66057	3.90281	-0.78601
C	-2.13725	1.61045	-0.12994
C	-2.94365	4.27895	-0.39954
H	-0.96879	4.63298	-1.19132
C	-3.42059	1.99382	0.25301
H	-1.82270	0.57936	-0.02425
C	-3.82444	3.32507	0.11936
H	-3.25796	5.31261	-0.50260
H	-4.10521	1.25233	0.65241
H	-4.82559	3.61968	0.41920
C	-2.36727	-2.54012	-0.20349
C	-2.62396	-2.98953	1.10036
C	-3.40039	-1.9385	-0.93151
C	-3.89030	-2.83944	1.66421
H	-1.82703	-3.45896	1.67076
C	-4.67266	-1.79293	-0.37081
H	-3.20839	-1.58731	-1.94172
C	-4.91836	-2.24098	0.92853
H	-4.07836	-3.19227	2.67396
H	-5.46792	-1.33053	-0.94787
H	-5.90610	-2.12878	1.36579
O	0.93658	2.99136	-1.54545
C	2.49382	2.05175	3.85782
H	2.74188	3.11260	3.87409
H	1.46976	1.90406	4.21186
H	3.19458	1.49676	4.48789

C	1.27489	-1.97808	-0.43726
H	1.48055	-3.01007	-0.74442
H	3.98785	-2.11758	1.28498

H-IM1C-*Z* (Hartree/Particle)

Zero-point correction= 0.427649

Thermal correction to Energy= 0.454322

Thermal correction to Enthalpy= 0.455266

Thermal correction to Gibbs Free Energy= 0.367052

Sum of electronic and thermal Free Energies= -1416.326209

C	2.39127	-1.54807	0.52192
H	2.26507	-2.38050	1.21006
C	4.48268	-0.58115	-0.20414
H	5.52817	-0.69740	-0.48423
C	1.75040	-0.62520	-1.79538
H	0.89888	-0.65494	-2.48519
C	1.78175	0.78501	-1.16377
H	2.19108	1.49957	-1.88127
C	2.18557	-0.21025	1.11789
C	2.57644	0.85035	0.15751
H	2.58615	1.85706	0.56644
C	1.74936	-0.01991	2.37606
H	1.50880	-0.86743	3.01597
O	0.42475	1.13552	-0.84489
O	0.23130	-1.85496	-0.31754
O	2.97531	-0.88123	-2.48117
O	4.02451	0.61254	-0.21372
O	3.85775	-1.64339	0.12383
O	1.55877	1.20420	2.88706
C	0.08177	2.45102	-0.87056
C	-0.58803	-2.76235	-1.09144
H	-0.63707	-2.42656	-2.13362
H	-0.12514	-3.75733	-1.07017
C	-1.35307	2.66104	-0.56558
C	-1.84605	3.97473	-0.57504
C	-2.21386	1.59051	-0.27565
C	-3.18907	4.21533	-0.29834
H	-1.17198	4.79392	-0.80030

C	-3.55654	1.83841	0.00018
H	-1.83402	0.57582	-0.26513
C	-4.04503	3.14787	-0.01097
H	-3.56900	5.23194	-0.30662
H	-4.22014	1.00846	0.22194
H	-5.09240	3.33693	0.20416
C	-1.96189	-2.79325	-0.47645
C	-2.17371	-3.45983	0.73913
C	-3.03764	-2.14071	-1.09093
C	-3.43774	-3.47383	1.32833
H	-1.34380	-3.97007	1.22071
C	-4.30668	-2.15765	-0.50515
H	-2.88121	-1.62347	-2.03367
C	-4.50746	-2.82285	0.70610
H	-3.59106	-3.99537	2.26851
H	-5.13529	-1.65559	-0.99569
H	-5.49299	-2.83814	1.16204
O	0.88431	3.33626	-1.12231
C	1.07815	1.24695	4.24813
H	0.97659	2.30270	4.49704
H	0.10669	0.75106	4.32312
H	1.79815	0.77312	4.92084
C	1.57513	-1.76831	-0.76829
H	1.89659	-2.70253	-1.24279
H	3.06985	-0.21994	-3.18363

Cartesian coordinates and total energies for all of the calculated structures (B3LYP/def2-TZVP//B3LYP/6-31G** [SMD = water])

SM-*E*

Zero-point correction= 0.415727 (Hartree/Particle)
Thermal correction to Energy= 0.439948
Thermal correction to Enthalpy= 0.440892
Thermal correction to Gibbs Free Energy= 0.361442
Sum of electronic and thermal Free Energies= -1416.415020

C	2.56005	-1.28933	0.18131
H	2.44709	-2.27143	0.63827
C	4.24995	0.09924	-0.72050
H	5.31530	0.14203	-0.94396
C	2.12028	0.20823	-1.75065
H	1.66065	0.33508	-2.73241
C	1.79791	1.39581	-0.82770
H	2.15913	2.31486	-1.29117
C	2.16445	-0.16477	1.09825
C	2.53558	1.16529	0.50931
H	2.38523	1.99923	1.19584
C	1.50857	-0.28882	2.25675
O	0.37674	1.48380	-0.63271
O	0.39921	-1.33178	-1.01009
O	3.55732	0.25294	-1.94582
O	3.95501	1.15636	0.16590
O	3.98106	-1.15443	-0.13853
C	-0.13194	2.69834	-0.31527
C	-0.02916	-2.70951	-0.95275
H	0.21578	-3.20529	-1.90064
H	0.50164	-3.23060	-0.14701
C	-1.60371	2.66778	-0.12607
C	-2.26022	3.87768	0.14645
C	-2.33902	1.47486	-0.20350
C	-3.63946	3.89485	0.33549
H	-1.68261	4.79358	0.20633
C	-3.71879	1.49798	-0.01014
H	-1.83318	0.53905	-0.40853
C	-4.36975	2.70522	0.25786
H	-4.14504	4.83237	0.54437
H	-4.28374	0.57283	-0.06634
H	-5.44511	2.71926	0.40775
C	-1.51355	-2.73430	-0.70009
C	-2.01139	-2.45336	0.58098
C	-2.41462	-3.00805	-1.73584
C	-3.38593	-2.44852	0.82049
H	-1.31508	-2.24171	1.38855
C	-3.79113	-3.00897	-1.49745

H	-2.03503	-3.22457	-2.73087
C	-4.27888	-2.72799	-0.21941
H	-3.76110	-2.23493	1.81703
H	-4.48021	-3.22804	-2.30775
H	-5.34855	-2.73164	-0.03147
O	0.56062	3.69984	-0.20186
C	1.80075	-1.18048	-1.16300
H	2.19778	-1.93227	-1.85718
O	1.09226	-1.49670	2.72933
C	0.63079	-1.48771	4.09072
H	-0.18486	-0.76858	4.21863
H	0.26744	-2.49438	4.29871
H	1.44929	-1.24344	4.77549
H	1.25285	0.57774	2.86514

IM1A-*E*

Zero-point correction= 0.451602 (Hartree/Particle)

Thermal correction to Energy= 0.478804

Thermal correction to Enthalpy= 0.479748

Thermal correction to Gibbs Free Energy= 0.393833

Sum of electronic and thermal Free Energies= -1493.288498

C	2.15620	-1.52050	-0.09062
H	1.99920	-2.48707	0.38499
C	3.89074	-0.31530	-1.16128
H	4.93835	-0.35239	-1.45397
C	1.69977	-0.00794	-2.00624
H	1.18576	0.15010	-2.95501
C	1.54618	1.20999	-1.08021
H	1.94659	2.09343	-1.57932
C	1.93689	-0.36161	0.84114
C	2.34577	0.93140	0.20950
H	2.32949	1.78257	0.88938
C	1.40878	-0.42270	2.07032
O	0.15972	1.41137	-0.77697
O	-0.07722	-1.39454	-1.11372
O	3.12105	-0.08070	-2.30818
O	3.74854	0.80636	-0.25745
O	3.56269	-1.50865	-0.51559
C	-0.22567	2.66742	-0.43499
C	-0.62691	-2.73046	-1.09060
H	-0.56357	-3.16668	-2.09518
H	-0.04154	-3.35789	-0.40740
C	-1.67644	2.75487	-0.14011
C	-2.21394	4.01599	0.16007
C	-2.50652	1.62296	-0.14535
C	-3.56990	4.14414	0.44828
H	-1.56319	4.88360	0.16303

C	-3.86187	1.75728	0.14843
H	-2.09311	0.64776	-0.37247
C	-4.39461	3.01517	0.44336
H	-3.98402	5.12086	0.67760
H	-4.49991	0.87910	0.14899
H	-5.45166	3.11599	0.67047
C	-2.05750	-2.64540	-0.62835
C	-2.34307	-2.33435	0.70970
C	-3.11586	-2.85017	-1.52087
C	-3.66426	-2.23009	1.14483
H	-1.52411	-2.17675	1.40699
C	-4.44013	-2.75331	-1.08559
H	-2.90099	-3.08867	-2.55906
C	-4.71634	-2.44107	0.24701
H	-3.87494	-1.99231	2.18334
H	-5.25299	-2.91909	-1.78640
H	-5.74497	-2.36719	0.58748
O	0.55431	3.60662	-0.38315
C	1.31400	-1.36023	-1.37768
H	1.59606	-2.14702	-2.08793
O	0.96912	-1.58597	2.61132
C	0.55924	-1.50323	3.98865
H	-0.23763	-0.76304	4.11002
H	0.18666	-2.49199	4.25610
H	1.40880	-1.24372	4.62788
H	1.28803	0.47050	2.68169
O	5.43419	0.75542	1.57119
H	5.38522	-0.10321	2.03290
H	4.70193	0.76498	0.79513
H	6.32010	0.81142	1.16481

IM1B-*E*

Zero-point correction= 0.452107 (Hartree/Particle)

Thermal correction to Energy= 0.479052

Thermal correction to Enthalpy= 0.479996

Thermal correction to Gibbs Free Energy= 0.394624

Sum of electronic and thermal Free Energies= -1493.287884

C	-2.44719	0.62059	0.39346
H	-2.51874	1.59303	0.87692
C	-3.89357	-1.10667	-0.47654
H	-4.94348	-1.34800	-0.63178
C	-1.85613	-0.71330	-1.62151
H	-1.45263	-0.71876	-2.63472
C	-1.23582	-1.82853	-0.76397
H	-1.42488	-2.79252	-1.23838
C	-1.79465	-0.42870	1.24267

C	-1.91396	-1.78824	0.62119
H	-1.55572	-2.59080	1.26546
C	-1.14044	-0.20772	2.38972
O	0.17832	-1.61331	-0.65124
O	-0.44843	1.13844	-0.92636
O	-3.26851	-1.04578	-1.73211
O	-3.33054	-2.06791	0.36501
O	-3.84891	0.18523	0.16326
C	0.95411	-2.69789	-0.40357
C	-0.31221	2.57748	-0.94746
H	-0.62057	2.95650	-1.92960
H	-0.96873	3.01945	-0.18829
C	2.39184	-2.35229	-0.28371
C	3.30890	-3.39802	-0.09745
C	2.84552	-1.02524	-0.33982
C	4.66717	-3.11923	0.02758
H	2.94770	-4.41966	-0.05349
C	4.20570	-0.75239	-0.20979
H	2.13918	-0.21571	-0.47848
C	5.11659	-1.79652	-0.02777
H	5.37473	-3.93002	0.16919
H	4.55225	0.27558	-0.24807
H	6.17594	-1.58000	0.07221
C	1.12606	2.92159	-0.66376
C	1.65165	2.73023	0.62311
C	1.96060	3.41291	-1.67414
C	2.98809	3.02558	0.89196
H	1.00715	2.35054	1.41188
C	3.29787	3.71609	-1.40527
H	1.56027	3.56027	-2.67360
C	3.81414	3.52107	-0.12271
H	3.38444	2.87737	1.89216
H	3.93451	4.10125	-2.19629
H	4.85285	3.75769	0.08840
O	0.49891	-3.82708	-0.29492
C	-1.79087	0.69753	-1.00541
H	-2.38278	1.36465	-1.64432
O	-0.98460	1.03613	2.90803
C	-0.36273	1.08458	4.20519
H	0.63016	0.62556	4.17465
H	-0.27185	2.14033	4.46053
H	-0.98451	0.57738	4.94941
H	-0.68442	-1.02417	2.94733
O	-5.29094	1.94276	-0.82124
H	-5.43452	1.79026	-1.77423
H	-4.67787	1.16171	-0.42447
H	-6.16492	1.92266	-0.38781

IM1C-*E*

Zero-point correction= 0.451970 (Hartree/Particle)
Thermal correction to Energy= 0.478979
Thermal correction to Enthalpy= 0.479923
Thermal correction to Gibbs Free Energy= 0.394427
Sum of electronic and thermal Free Energies= -1493.287041

C	2.36055	-0.99501	0.75844
H	2.27317	-1.95669	1.26060
C	4.05587	0.49139	0.03508
H	5.13217	0.61935	-0.06438
C	2.05896	0.34513	-1.31530
H	1.73973	0.37752	-2.35732
C	1.52818	1.55130	-0.52176
H	1.87729	2.47114	-0.99302
C	1.75428	0.13690	1.53764
C	2.08628	1.45913	0.91497
H	1.77834	2.31431	1.51622
C	0.96230	0.01532	2.60915
O	0.09526	1.51771	-0.52895
O	0.40025	-1.29929	-0.70715
O	3.51494	0.52427	-1.31607
O	3.54006	1.56555	0.76124
O	3.80162	-0.75055	0.61804
C	-0.55193	2.69939	-0.36397
C	0.07805	-2.70641	-0.63084
H	0.48168	-3.21840	-1.51314
H	0.53981	-3.14422	0.26198
C	-2.02695	2.54692	-0.38738
C	-2.81197	3.70511	-0.27891
C	-2.64370	1.29177	-0.50698
C	-4.20070	3.60903	-0.29407
H	-2.32566	4.66983	-0.18468
C	-4.03409	1.20206	-0.51825
H	-2.03969	0.39597	-0.58575
C	-4.81278	2.35776	-0.41350
H	-4.80561	4.50648	-0.21229
H	-4.50797	0.22955	-0.60677
H	-5.89604	2.28371	-0.42377
C	-1.41969	-2.84464	-0.56568
C	-2.10182	-2.52698	0.61837
C	-2.15181	-3.26348	-1.68275
C	-3.49176	-2.62841	0.68228
H	-1.53646	-2.20308	1.48862
C	-3.54324	-3.37145	-1.61928
H	-1.62849	-3.50882	-2.60306
C	-4.21522	-3.05284	-0.43732
H	-4.01067	-2.38469	1.60466
H	-4.10038	-3.70249	-2.49071
H	-5.29657	-3.13926	-0.38531

O	0.03675	3.75988	-0.21517
C	1.79014	-1.03371	-0.67994
H	2.34066	-1.78383	-1.26252
O	0.59414	-1.19703	3.10091
C	-0.06769	-1.16247	4.37762
H	-0.95563	-0.52363	4.33577
H	-0.36358	-2.18845	4.59691
H	0.61243	-0.80085	5.15527
H	0.55556	0.88681	3.12034
O	4.73228	-0.95397	-2.91405
H	4.21873	-0.32689	-2.22977
H	5.11212	-1.70381	-2.41726
H	5.47533	-0.45695	-3.30671

TS1A-*E*

Zero-point correction= 0.449486 (Hartree/Particle)

Thermal correction to Energy= 0.477104

Thermal correction to Enthalpy= 0.478048

Thermal correction to Gibbs Free Energy= 0.391205

Sum of electronic and thermal Free Energies= -1493.282872

C	2.25140	-1.34916	-0.28913
H	2.21712	-2.36912	0.08358
C	3.90237	-0.15366	-1.52841
H	4.94567	0.04173	-1.75425
C	1.61908	0.29534	-2.01480
H	1.07810	0.51791	-2.93275
C	1.45322	1.41585	-0.97200
H	1.79877	2.35583	-1.40471
C	2.03758	-0.31063	0.76308
C	2.27225	1.10409	0.30141
H	2.02600	1.82788	1.08283
C	1.60663	-0.58264	2.00635
O	0.06122	1.50764	-0.65185
O	-0.01274	-1.27197	-1.19219
O	3.04850	0.35153	-2.38144
O	3.65978	1.30115	-0.10822
O	3.66734	-1.18890	-0.78148
C	-0.41551	2.72935	-0.28923
C	-0.49131	-2.63786	-1.27273
H	-0.44770	-2.97286	-2.31556
H	0.15672	-3.28738	-0.67268
C	-1.86458	2.69928	0.02123
C	-2.49372	3.90915	0.35321
C	-2.60612	1.50757	-0.00283
C	-3.85275	3.92691	0.65437
H	-1.91182	4.82412	0.37037

C	-3.96500	1.53175	0.30360
H	-2.12207	0.57200	-0.25560
C	-4.58915	2.73870	0.63034
H	-4.33811	4.86399	0.90804
H	-4.53450	0.60780	0.28855
H	-5.64878	2.75375	0.86690
C	-1.90161	-2.67127	-0.74949
C	-2.14393	-2.50012	0.62188
C	-2.98488	-2.85190	-1.61689
C	-3.44874	-2.50887	1.11460
H	-1.30461	-2.36310	1.29917
C	-4.29216	-2.86897	-1.12409
H	-2.80218	-2.98279	-2.68005
C	-4.52598	-2.69530	0.24152
H	-3.62686	-2.37852	2.17792
H	-5.12483	-3.01552	-1.80558
H	-5.54141	-2.71018	0.62635
O	0.29221	3.72270	-0.23451
C	1.35167	-1.13163	-1.51990
H	1.64364	-1.82742	-2.31594
O	1.31797	-1.83562	2.41584
C	0.99138	-1.97021	3.81385
H	0.14040	-1.33326	4.07174
H	0.72858	-3.01700	3.96381
H	1.85493	-1.71199	4.43378
H	1.44255	0.21512	2.72935
O	5.16205	0.50654	1.91731
H	4.48041	0.11984	2.48974
H	4.25303	1.03859	0.66332
H	5.60545	-0.26857	1.53838

TS1B-*E*

Zero-point correction= 0.448753 (Hartree/Particle)

Thermal correction to Energy= 0.476665

Thermal correction to Enthalpy= 0.477609

Thermal correction to Gibbs Free Energy= 0.389327

Sum of electronic and thermal Free Energies= -1493.283349

C	-2.41474	0.67552	0.47301
H	-2.26363	1.64164	0.95855
C	-3.90624	-1.38964	-0.57345
H	-4.98520	-1.47675	-0.64989
C	-1.86370	-0.69735	-1.58600
H	-1.50525	-0.69291	-2.61359
C	-1.23566	-1.82203	-0.75942
H	-1.42731	-2.78311	-1.23992
C	-1.81209	-0.44778	1.27860

C	-1.88786	-1.78632	0.63059
H	-1.55403	-2.60740	1.26114
C	-1.20723	-0.30040	2.46933
O	0.16932	-1.59666	-0.65669
O	-0.46397	1.13573	-0.91948
O	-3.30266	-1.02619	-1.67775
O	-3.34563	-2.07986	0.37311
O	-3.84957	0.41177	0.38721
C	0.95841	-2.69086	-0.46072
C	-0.30468	2.56763	-1.04351
H	-0.53405	2.87152	-2.07199
H	-1.01048	3.07186	-0.37194
C	2.39232	-2.33803	-0.34613
C	3.31757	-3.38320	-0.19927
C	2.83613	-1.00645	-0.36790
C	4.67478	-3.09860	-0.07864
H	2.96343	-4.40807	-0.18180
C	4.19554	-0.72856	-0.24260
H	2.12378	-0.19772	-0.47694
C	5.11462	-1.77158	-0.09932
H	5.38902	-3.90815	0.03302
H	4.53500	0.30237	-0.25404
H	6.17336	-1.55087	-0.00249
C	1.11337	2.91914	-0.67781
C	1.56466	2.73134	0.63785
C	1.99992	3.42125	-1.63690
C	2.87980	3.04006	0.98420
H	0.88084	2.34155	1.38771
C	3.31582	3.73902	-1.28966
H	1.65774	3.56467	-2.65826
C	3.75850	3.54663	0.02027
H	3.21900	2.89297	2.00535
H	3.99338	4.13204	-2.04185
H	4.78065	3.79317	0.29207
O	0.50540	-3.82171	-0.38928
C	-1.81345	0.70848	-0.95705
H	-2.41473	1.37190	-1.58940
O	-1.05948	0.90401	3.05675
C	-0.39601	0.89187	4.33650
H	0.61533	0.48682	4.23976
H	-0.34851	1.93057	4.66232
H	-0.96921	0.30275	5.05825
H	-0.79071	-1.15449	3.00116
O	-4.99954	2.11074	-1.27853
H	-5.39065	1.51201	-1.93323
H	-4.28278	1.08507	-0.22466
H	-5.76477	2.49512	-0.82395

TS1C-*E*

Zero-point correction= 0.448680 (Hartree/Particle)

Thermal correction to Energy= 0.476364

Thermal correction to Enthalpy= 0.477308

Thermal correction to Gibbs Free Energy= 0.389921

Sum of electronic and thermal Free Energies= -1493.284430

C	2.33105	-0.92009	0.73858
H	2.28062	-1.88622	1.23378
C	4.12330	0.58639	0.29761
H	5.17394	0.73884	0.06995
C	2.01826	0.40205	-1.39536
H	1.54820	0.40116	-2.38276
C	1.50253	1.59678	-0.56954
H	1.84706	2.52549	-1.02646
C	1.71478	0.19432	1.51372
C	2.01414	1.50840	0.88606
H	1.70572	2.36997	1.47456
C	0.97401	0.06706	2.62513
O	0.07174	1.55084	-0.56924
O	0.43269	-1.28727	-0.73442
O	3.43937	0.65401	-1.53344
O	3.49669	1.64464	0.75288
O	3.79781	-0.64331	0.61149
C	-0.59328	2.72734	-0.41926
C	0.15060	-2.70655	-0.75391
H	0.50040	-3.13191	-1.70209
H	0.68954	-3.19976	0.06399
C	-2.06433	2.54584	-0.41765
C	-2.87117	3.69185	-0.34692
C	-2.65629	1.27409	-0.47263
C	-4.25762	3.56687	-0.33590
H	-2.40363	4.66940	-0.30273
C	-4.04432	1.15582	-0.45643
H	-2.03524	0.38781	-0.52169
C	-4.84509	2.29928	-0.38987
H	-4.87999	4.45454	-0.28424
H	-4.49857	0.17060	-0.49323
H	-5.92658	2.20311	-0.37867
C	-1.33510	-2.89055	-0.59353
C	-1.94901	-2.59354	0.63279
C	-2.12385	-3.33459	-1.66079
C	-3.32789	-2.73794	0.78587
H	-1.34021	-2.25037	1.46541
C	-3.50426	-3.48745	-1.50706
H	-1.65423	-3.56281	-2.61383
C	-4.10852	-3.18712	-0.28477
H	-3.79364	-2.50839	1.73972
H	-4.10560	-3.83721	-2.34097
H	-5.18112	-3.30648	-0.16321

O	-0.01869	3.79818	-0.30045
C	1.81219	-0.97420	-0.71844
H	2.38897	-1.71859	-1.28015
O	0.66833	-1.13755	3.14750
C	-0.04904	-1.10805	4.39821
H	-0.98922	-0.56038	4.28698
H	-0.25387	-2.14732	4.65343
H	0.56385	-0.64790	5.17855
H	0.56818	0.94001	3.13415
O	4.61774	-1.25873	-2.82982
H	3.88318	-0.09304	-2.06639
H	5.04059	-1.76571	-2.11925
H	5.35969	-0.86718	-3.31655

IM2A-*E*

Zero-point correction= 0.425093 (Hartree/Particle)

Thermal correction to Energy= 0.450906

Thermal correction to Enthalpy= 0.451850

Thermal correction to Gibbs Free Energy= 0.369135

Sum of electronic and thermal Free Energies= -1416.832611

C	2.71574	-0.90223	-0.27293
H	2.91806	-1.93951	-0.02288
C	4.07291	0.48925	-1.72396
H	5.04503	0.80991	-2.09221
C	1.68699	0.74642	-1.77180
H	1.05573	0.95842	-2.63260
C	1.37397	1.71145	-0.61335
H	1.59402	2.73087	-0.93471
C	2.39954	-0.05530	0.90708
C	2.18332	1.41901	0.67048
H	1.64640	1.85905	1.51802
C	2.29768	-0.57298	2.14751
O	-0.03326	1.57723	-0.37116
O	0.40692	-1.12820	-1.02239
O	3.05732	1.08481	-2.23300
O	3.43634	2.10521	0.46548
O	4.05596	-0.44936	-0.86847
C	-0.69462	2.68287	0.06444
C	0.18809	-2.56304	-1.04345
H	0.47390	-2.94349	-2.03192
H	0.81492	-3.04710	-0.28714
C	-2.14427	2.43747	0.25134
C	-2.93754	3.49995	0.71118
C	-2.73038	1.19068	-0.01787
C	-4.30451	3.31693	0.90002
H	-2.47413	4.45884	0.91604

C	-4.09898	1.01412	0.17387
H	-2.12058	0.36855	-0.37300
C	-4.88613	2.07417	0.63193
H	-4.91607	4.14001	1.25576
H	-4.54883	0.04830	-0.03387
H	-5.95236	1.93224	0.78045
C	-1.26858	-2.81347	-0.76283
C	-1.68465	-3.27808	0.49059
C	-2.23109	-2.55679	-1.75018
C	-3.03978	-3.49012	0.75406
H	-0.94292	-3.47513	1.25989
C	-3.58584	-2.76274	-1.48770
H	-1.91418	-2.19958	-2.72637
C	-3.99217	-3.23163	-0.23410
H	-3.35079	-3.85529	1.72831
H	-4.32295	-2.56705	-2.26076
H	-5.04594	-3.39917	-0.03186
O	-0.13245	3.74670	0.27170
C	1.70171	-0.74182	-1.41557
H	2.03800	-1.31090	-2.29235
O	2.50991	-1.87440	2.41229
C	2.38969	-2.25703	3.79784
H	1.38322	-2.04162	4.16748
H	2.57465	-3.33032	3.83120
H	3.13405	-1.73321	4.40412
H	2.01617	0.05557	2.99152
H	4.02215	1.84200	1.19172

IM2B-*E*

Zero-point correction= 0.424677 (Hartree/Particle)

Thermal correction to Energy= 0.450620

Thermal correction to Enthalpy= 0.451564

Thermal correction to Gibbs Free Energy= 0.368498

Sum of electronic and thermal Free Energies= -1416.832419

C	2.32114	-1.40047	0.27636
H	1.83709	-2.10048	0.96425
C	4.08874	0.35610	-1.72048
H	5.08904	0.24557	-2.13453
C	1.74236	-0.13949	-1.89538
H	1.13599	-0.16996	-2.79830
C	1.61168	1.18886	-1.15206
H	1.86638	2.01785	-1.81577
C	2.39838	-0.03170	0.91562
C	2.58110	1.13799	0.03042
H	2.65679	2.08751	0.55405
C	2.36343	0.17313	2.24929

O	0.26784	1.32368	-0.70063
O	0.12344	-1.48357	-0.80475
O	3.14121	-0.18874	-2.39310
O	3.97101	1.03109	-0.65133
O	3.63902	-1.87921	-0.07178
C	-0.17923	2.59577	-0.48097
C	-0.38100	-2.83821	-0.75363
H	-0.17274	-3.32539	-1.71515
H	0.13184	-3.40008	0.03535
C	-1.60915	2.63312	-0.10174
C	-2.18190	3.88354	0.17940
C	-2.39212	1.47008	-0.02918
C	-3.52605	3.96907	0.53026
H	-1.56793	4.77546	0.11962
C	-3.73710	1.56353	0.32145
H	-1.95273	0.50400	-0.24618
C	-4.30448	2.80977	0.60090
H	-3.96761	4.93643	0.74738
H	-4.34100	0.66313	0.37339
H	-5.35335	2.87829	0.87316
C	-1.86076	-2.77990	-0.48421
C	-2.37592	-3.13824	0.76721
C	-2.74122	-2.33852	-1.48298
C	-3.74823	-3.06412	1.01756
H	-1.69907	-3.47885	1.54614
C	-4.11143	-2.25701	-1.23348
H	-2.34877	-2.06231	-2.45800
C	-4.61774	-2.62140	0.01852
H	-4.13650	-3.35002	1.99060
H	-4.78469	-1.91775	-2.01512
H	-5.68513	-2.56541	0.21087
O	0.54695	3.56870	-0.59658
C	1.51881	-1.40737	-1.04551
H	1.85888	-2.26331	-1.64080
O	2.18960	-0.83033	3.12283
C	2.07652	-0.44154	4.50826
H	1.18824	0.17836	4.65777
H	1.98199	-1.36774	5.07418
H	2.97222	0.09920	4.82627
H	2.46265	1.17306	2.66956
H	4.18079	-1.82835	0.73027

IM2C-*E*

Zero-point correction= 0.424809 (Hartree/Particle)

Thermal correction to Energy= 0.450616

Thermal correction to Enthalpy= 0.451560

Thermal correction to Gibbs Free Energy= 0.369016

Sum of electronic and thermal Free Energies= -1416.834022

C	2.41751	-1.31162	0.22132
H	2.29261	-2.29615	0.66382
C	4.48782	-0.07900	0.04864
H	5.56329	-0.06878	-0.11822
C	1.97505	0.15301	-1.84569
H	1.22080	0.25601	-2.63390
C	1.81221	1.34939	-0.88114
H	2.23402	2.24873	-1.33501
C	2.02914	-0.18404	1.10406
C	2.44094	1.10615	0.50877
H	2.31567	1.97074	1.15712
C	1.41571	-0.28158	2.29623
O	0.40319	1.52506	-0.66211
O	0.37095	-1.41817	-1.01007
O	3.29394	0.14089	-2.38916
O	3.93919	1.05132	0.28310
O	3.91870	-1.22095	0.01851
C	-0.05397	2.77555	-0.39042
C	-0.06425	-2.79654	-1.05496
H	0.13439	-3.20819	-2.05163
H	0.49753	-3.38672	-0.32056
C	-1.51798	2.79580	-0.15913
C	-2.13589	4.03534	0.06583
C	-2.28281	1.61835	-0.14756
C	-3.50753	4.09668	0.29632
H	-1.53511	4.93826	0.05706
C	-3.65412	1.68651	0.08748
H	-1.80668	0.65970	-0.31581
C	-4.26723	2.92298	0.30807
H	-3.98453	5.05635	0.46816
H	-4.24138	0.77355	0.10056
H	-5.33644	2.97243	0.49071
C	-1.53703	-2.82271	-0.74050
C	-1.98526	-2.47073	0.54183
C	-2.47522	-3.17190	-1.71806
C	-3.34867	-2.46494	0.83693
H	-1.26053	-2.20357	1.30660
C	-3.84076	-3.17615	-1.42144
H	-2.13459	-3.44172	-2.71405
C	-4.27976	-2.81938	-0.14520
H	-3.68531	-2.19302	1.83302
H	-4.55898	-3.45292	-2.18750
H	-5.34080	-2.82081	0.08635
O	0.67905	3.75104	-0.34630
C	1.76507	-1.22446	-1.18027
H	2.19973	-1.98507	-1.83839
O	1.02397	-1.46143	2.80041
C	0.42951	-1.41533	4.11589

H	-0.45275	-0.76965	4.11154
H	0.14055	-2.43906	4.35042
H	1.15783	-1.05611	4.84789
H	1.18978	0.60756	2.88296
H	3.42414	0.96793	-2.87820

TS2A-*E*

Zero-point correction= 0.422913 (Hartree/Particle)

Thermal correction to Energy= 0.448838

Thermal correction to Enthalpy= 0.449782

Thermal correction to Gibbs Free Energy= 0.366966

Sum of electronic and thermal Free Energies= -1416.829549

C	-2.48006	0.98489	-0.17987
H	-2.78582	1.99301	0.07895
C	-3.96724	-0.33298	-2.04339
H	-4.84825	-0.70053	-2.57543
C	-1.56548	-0.60811	-1.84000
H	-0.85086	-0.75038	-2.64986
C	-1.30921	-1.64374	-0.73058
H	-1.51477	-2.64024	-1.12393
C	-2.43191	0.01900	0.85474
C	-2.17100	-1.44253	0.53083
H	-1.66231	-1.92521	1.37236
C	-2.73110	0.36465	2.15283
O	0.08710	-1.53348	-0.40903
O	-0.25086	1.21264	-0.98326
O	-2.86860	-0.92684	-2.44082
O	-3.40253	-2.12473	0.24261
O	-4.07472	0.56070	-1.19029
C	0.72218	-2.66679	-0.00999
C	0.05522	2.61503	-1.21403
H	-0.05191	2.82067	-2.28578
H	-0.65411	3.24266	-0.66450
C	2.16223	-2.44430	0.26338
C	2.92650	-3.53869	0.69684
C	2.76761	-1.18862	0.09903
C	4.28327	-3.37843	0.96390
H	2.44904	-4.50461	0.82005
C	4.12585	-1.03492	0.36850
H	2.18092	-0.34182	-0.23636
C	4.88390	-2.12662	0.80039
H	4.87214	-4.22641	1.29886
H	4.59051	-0.06227	0.24083
H	5.94219	-2.00246	1.00942
C	1.46275	2.86838	-0.74972
C	1.70851	3.43207	0.50874

C	2.54879	2.51503	-1.56328
C	3.01697	3.64502	0.94679
H	0.87034	3.70816	1.14290
C	3.85785	2.72351	-1.12617
H	2.36462	2.08244	-2.54305
C	4.09340	3.28974	0.13048
H	3.19574	4.08822	1.92190
H	4.69211	2.45378	-1.76686
H	5.11163	3.45848	0.46828
O	0.14790	-3.73878	0.10305
C	-1.56444	0.84948	-1.37629
H	-1.91147	1.48510	-2.19715
O	-3.08953	1.57970	2.50252
C	-3.32517	1.81559	3.91706
H	-2.40154	1.64801	4.47433
H	-3.63234	2.85686	3.99286
H	-4.11934	1.15605	4.27199
H	-2.65090	-0.37607	2.94968
H	-3.99214	-1.99705	1.00116

TS2B-*E*

Zero-point correction= 0.423283 (Hartree/Particle)

Thermal correction to Energy= 0.449084

Thermal correction to Enthalpy= 0.450028

Thermal correction to Gibbs Free Energy= 0.367366

Sum of electronic and thermal Free Energies= -1416.831076

C	2.33403	-1.35935	0.16172
H	1.87986	-2.08868	0.83981
C	3.99533	0.55342	-1.90156
H	4.94709	0.45704	-2.42522
C	1.64637	-0.00727	-1.92454
H	0.97860	-0.00567	-2.78443
C	1.50829	1.27707	-1.10951
H	1.74361	2.14676	-1.72484
C	2.43987	-0.01652	0.85842
C	2.45523	1.19235	0.07463
H	2.59968	2.12461	0.61188
C	2.62472	0.10444	2.20188
O	0.15768	1.36585	-0.64956
O	0.09734	-1.43642	-0.83306
O	3.00089	-0.01078	-2.51227
O	3.95899	1.19400	-0.82397
O	3.63738	-1.81475	-0.25293
C	-0.30713	2.61711	-0.35866
C	-0.37237	-2.80383	-0.82080
H	-0.18513	-3.24655	-1.80795

H	0.17980	-3.38499	-0.07323
C	-1.73639	2.61282	0.02490
C	-2.32972	3.83970	0.36152
C	-2.49827	1.43393	0.05061
C	-3.67363	3.88628	0.72051
H	-1.73175	4.74409	0.33747
C	-3.84289	1.48831	0.41060
H	-2.04250	0.48592	-0.20849
C	-4.43097	2.71122	0.74494
H	-4.13132	4.83553	0.98002
H	-4.43031	0.57573	0.42762
H	-5.47955	2.74927	1.02416
C	-1.84341	-2.79616	-0.50096
C	-2.30843	-3.23518	0.74438
C	-2.76692	-2.32591	-1.44632
C	-3.67333	-3.21193	1.04098
H	-1.59810	-3.59915	1.48188
C	-4.12997	-2.29504	-1.15015
H	-2.41379	-1.98771	-2.41682
C	-4.58586	-2.73999	0.09519
H	-4.02238	-3.56008	2.00859
H	-4.83677	-1.93291	-1.89085
H	-5.64752	-2.72330	0.32328
O	0.40450	3.60563	-0.42061
C	1.48077	-1.31545	-1.12548
H	1.81414	-2.13596	-1.77198
O	2.62095	-0.95193	3.00490
C	2.71254	-0.68135	4.42475
H	1.82468	-0.13873	4.75754
H	2.76228	-1.65466	4.91034
H	3.61642	-0.10623	4.63889
H	2.75890	1.08051	2.66781
H	4.20524	-1.81978	0.53225

TS2Ca-*E*

Zero-point correction= 0.422473 (Hartree/Particle)

Thermal correction to Energy= 0.448430

Thermal correction to Enthalpy= 0.449374

Thermal correction to Gibbs Free Energy= 0.366225

Sum of electronic and thermal Free Energies= -1416.826859

C	2.63631	-0.65184	-0.24085
H	2.80068	-1.70581	-0.02842
C	4.35332	1.09984	-0.26520
H	5.40840	1.27150	-0.50066
C	1.51362	0.98563	-1.86805
H	0.64726	1.01567	-2.53877

C	1.16482	1.86237	-0.64418
H	1.37872	2.91143	-0.85278
C	2.06709	0.09085	0.93156
C	1.78801	1.43986	0.67332
H	1.61867	2.12102	1.50325
C	2.01396	-0.38809	2.21781
O	-0.25308	1.69775	-0.39943
O	0.51658	-1.09806	-1.23693
O	2.68371	1.45786	-2.52310
O	3.65558	2.02054	0.18361
O	4.00699	-0.14618	-0.50287
C	-0.95827	2.77800	0.03814
C	0.43511	-2.49263	-1.60929
H	0.41692	-2.57575	-2.70254
H	1.32016	-3.02460	-1.23978
C	-2.39705	2.46930	0.21013
C	-3.24793	3.50451	0.62692
C	-2.91581	1.18625	-0.02786
C	-4.60685	3.25926	0.80254
H	-2.83532	4.49099	0.80800
C	-4.27647	0.94793	0.15201
H	-2.25944	0.38538	-0.34734
C	-5.12178	1.98129	0.56564
H	-5.26379	4.06115	1.12396
H	-4.67690	-0.04432	-0.02967
H	-6.18173	1.79069	0.70403
C	-0.81621	-3.06865	-1.00114
C	-0.90771	-3.23133	0.38953
C	-1.90348	-3.43350	-1.80270
C	-2.06565	-3.75247	0.96530
H	-0.06615	-2.94858	1.01713
C	-3.06292	-3.96135	-1.22791
H	-1.83994	-3.30587	-2.87997
C	-3.14597	-4.12009	0.15636
H	-2.12590	-3.87707	2.04250
H	-3.89899	-4.24478	-1.86040
H	-4.04622	-4.52990	0.60474
O	-0.43867	3.86060	0.25386
C	1.77210	-0.48808	-1.50715
H	2.27447	-0.95945	-2.35830
O	2.30572	-1.63389	2.49701
C	2.16484	-2.05479	3.88396
H	1.11851	-1.97215	4.18255
H	2.48993	-3.09271	3.90735
H	2.80328	-1.43678	4.51745
H	1.68137	0.25397	3.03366
H	2.52283	2.37347	-2.79655

TS2Cb-*E*

Zero-point correction= 0.422297 (Hartree/Particle)
Thermal correction to Energy= 0.448344
Thermal correction to Enthalpy= 0.449288
Thermal correction to Gibbs Free Energy= 0.365881
Sum of electronic and thermal Free Energies= -1416.825781

C	1.95324	-1.42707	0.22470
H	1.84162	-2.39741	0.69948
C	4.51016	-0.43623	-0.09572
H	5.59577	-0.37500	-0.22074
C	1.82073	-0.04168	-1.95200
H	1.02777	0.11082	-2.69290
C	1.84093	1.19165	-1.02539
H	2.31906	2.02959	-1.53631
C	1.97156	-0.26475	1.02611
C	2.52860	0.93628	0.32829
H	2.49235	1.83398	0.94327
C	1.69794	-0.25626	2.36930
O	0.46578	1.50952	-0.73866
O	0.08394	-1.46874	-1.05835
O	3.08524	-0.20854	-2.58213
O	3.97986	0.75320	0.08488
O	3.93806	-1.53447	-0.12086
C	0.14959	2.79740	-0.46891
C	-0.44295	-2.80834	-1.22758
H	-0.38329	-3.08564	-2.28614
H	0.16762	-3.51401	-0.65174
C	-1.29144	2.97496	-0.17847
C	-1.76199	4.27519	0.06141
C	-2.17867	1.88742	-0.12983
C	-3.10846	4.48664	0.34410
H	-1.06735	5.10717	0.02265
C	-3.52402	2.10618	0.15691
H	-1.81754	0.88169	-0.31013
C	-3.99017	3.40271	0.39262
H	-3.47083	5.49317	0.52718
H	-4.20775	1.26422	0.19786
H	-5.03997	3.56842	0.61498
C	-1.86762	-2.81230	-0.74136
C	-2.15198	-2.51357	0.60009
C	-2.92141	-3.10842	-1.61250
C	-3.46891	-2.51115	1.05831
H	-1.33757	-2.28229	1.28174
C	-4.24084	-3.11602	-1.15198
H	-2.70764	-3.33431	-2.65363
C	-4.51675	-2.81475	0.18259
H	-3.67915	-2.27928	2.09824
H	-5.05043	-3.35079	-1.83661
H	-5.54179	-2.81714	0.54119

O	0.96672	3.67709	-0.47207
C	1.50260	-1.35771	-1.21806
H	1.90712	-2.18760	-1.80233
O	1.24437	-1.32133	2.99204
C	0.99256	-1.19191	4.41839
H	0.28403	-0.37990	4.59221
H	0.56599	-2.14411	4.72733
H	1.93341	-1.00670	4.93986
H	1.80772	0.65942	2.95047
H	3.23275	0.56223	-3.15097

PA-*E*

Zero-point correction= 0.422308 (Hartree/Particle)

Thermal correction to Energy= 0.449362

Thermal correction to Enthalpy= 0.450307

Thermal correction to Gibbs Free Energy= 0.364684

Sum of electronic and thermal Free Energies= -1416.842129

C	-1.99722	1.19860	0.43013
H	-1.98625	2.09645	1.04188
C	-3.81374	0.71352	-2.47425
H	-4.49627	0.53430	-3.31479
C	-1.51772	0.08255	-1.82168
H	-0.65578	0.06527	-2.48919
C	-1.53627	-1.24351	-1.04067
H	-1.79367	-2.06143	-1.71457
C	-2.43720	0.02207	0.95782
C	-2.49839	-1.27349	0.15588
H	-2.19861	-2.10529	0.80349
C	-2.88417	-0.03481	2.30710
O	-0.18619	-1.40760	-0.56319
O	0.03151	1.48810	-0.63523
O	-2.64983	0.10066	-2.73474
O	-3.80137	-1.50936	-0.37607
O	-4.09214	1.37540	-1.49641
C	0.28960	-2.67124	-0.43062
C	0.51052	2.84970	-0.76903
H	0.34336	3.17636	-1.80317
H	-0.05311	3.50885	-0.09977
C	1.70875	-2.69682	0.00033
C	2.30207	-3.94429	0.24724
C	2.46104	-1.52137	0.15280
C	3.63421	-4.01489	0.64576
H	1.71298	-4.84665	0.12512
C	3.79473	-1.59906	0.54805
H	2.00703	-0.55687	-0.04016
C	4.38168	-2.84295	0.79581

H	4.09048	-4.98075	0.83815
H	4.37462	-0.68800	0.65878
H	5.42110	-2.89973	1.10466
C	1.97386	2.85730	-0.42124
C	2.41487	3.36028	0.80886
C	2.91238	2.32334	-1.31660
C	3.77158	3.33620	1.13922
H	1.69201	3.77370	1.50704
C	4.26752	2.29213	-0.98597
H	2.57734	1.93549	-2.27487
C	4.69932	2.79973	0.24358
H	4.10300	3.73390	2.09380
H	4.98665	1.88044	-1.68783
H	5.75479	2.78176	0.49854
O	-0.39292	-3.66056	-0.64919
C	-1.36756	1.32569	-0.92447
H	-1.74872	2.20706	-1.44476
O	-2.93544	1.00438	3.04803
C	-3.38369	0.90124	4.43660
H	-2.54547	1.22883	5.05033
H	-4.22569	1.58545	4.53143
H	-3.67133	-0.12564	4.66148
H	-3.19389	-0.98864	2.74001
H	-4.40062	-1.70248	0.36088

PB-*E*

Zero-point correction= 0.422603 (Hartree/Particle)

Thermal correction to Energy= 0.449516

Thermal correction to Enthalpy= 0.450461

Thermal correction to Gibbs Free Energy= 0.365138

Sum of electronic and thermal Free Energies= -1416.846480

C	2.66351	-0.91916	-0.26083
H	2.53312	-1.94137	0.11029
C	3.50942	1.96915	-2.10568
H	4.17979	2.17213	-2.95056
C	1.37695	0.79171	-1.70977
H	0.50209	0.83881	-2.35827
C	1.08022	1.66091	-0.47630
H	1.28752	2.71281	-0.67103
C	2.44422	0.04512	0.89757
C	1.77363	1.22124	0.78054
H	1.66247	1.87640	1.64157
C	2.97895	-0.22951	2.18393
O	-0.34129	1.51271	-0.21469
O	0.36277	-1.20442	-0.95426
O	2.45027	1.28392	-2.55790

O	3.71396	2.33575	-0.96721
O	3.96411	-0.76448	-0.82857
C	-1.01163	2.60111	0.25106
C	0.14865	-2.59295	-1.29208
H	0.24350	-2.70960	-2.37939
H	0.90782	-3.22105	-0.81121
C	-2.45359	2.32354	0.45206
C	-3.26890	3.36850	0.91319
C	-3.00923	1.05937	0.19834
C	-4.62854	3.15070	1.11798
H	-2.82869	4.34068	1.10622
C	-4.37007	0.84815	0.40729
H	-2.38157	0.25059	-0.15676
C	-5.17989	1.89086	0.86588
H	-5.25791	3.96000	1.47420
H	-4.79599	-0.13122	0.21280
H	-6.24025	1.72205	1.02763
C	-1.22986	-2.98031	-0.82624
C	-1.40841	-3.71539	0.35191
C	-2.35819	-2.57930	-1.55633
C	-2.69082	-4.05021	0.79263
H	-0.53838	-4.02885	0.92274
C	-3.64114	-2.90864	-1.11613
H	-2.22707	-2.01399	-2.47520
C	-3.80895	-3.64615	0.06008
H	-2.81613	-4.62497	1.70548
H	-4.50763	-2.60015	-1.69358
H	-4.80657	-3.90822	0.39993
O	-0.46329	3.66922	0.47130
C	1.62438	-0.69110	-1.37145
H	1.97606	-1.19349	-2.27967
O	3.62804	-1.30854	2.38906
C	4.16816	-1.61620	3.71501
H	3.69612	-2.54866	4.02141
H	5.24025	-1.74893	3.57743
H	3.94589	-0.80374	4.40633
H	2.84181	0.47056	3.01026
H	4.61399	-1.07153	-0.17844

PCa-*E*

Zero-point correction= 0.422985 (Hartree/Particle)

Thermal correction to Energy= 0.449855

Thermal correction to Enthalpy= 0.450799

Thermal correction to Gibbs Free Energy= 0.365180

Sum of electronic and thermal Free Energies= -1416.855438

C	-2.61074	-0.03569	-0.24450

H	-2.68006	0.86470	0.36864
C	-4.77201	0.72571	-0.91570
H	-5.72647	0.37321	-1.32555
C	-1.02958	-1.07102	-1.97965
H	-0.23650	-0.80790	-2.68837
C	-0.43988	-2.01788	-0.92246
H	-0.36166	-3.02388	-1.34665
C	-2.23956	-1.20671	0.63360
C	-1.24918	-2.08596	0.33829
H	-0.99076	-2.86871	1.04654
C	-2.91264	-1.41754	1.87257
O	0.88537	-1.56160	-0.55784
O	-0.49422	0.96366	-0.85428
O	-2.12007	-1.72155	-2.62052
O	-4.52363	1.87775	-0.62792
O	-3.93194	-0.31702	-0.78677
C	1.80762	-2.50364	-0.22737
C	-0.65503	2.39652	-0.96160
H	-0.76261	2.65772	-2.02247
H	-1.56344	2.71544	-0.43739
C	3.12439	-1.90770	0.10113
C	4.17351	-2.77266	0.44824
C	3.33756	-0.52022	0.07746
C	5.42523	-2.25389	0.76784
H	3.99750	-3.84265	0.46454
C	4.59212	-0.00803	0.39978
H	2.52815	0.14960	-0.18795
C	5.63541	-0.87188	0.74424
H	6.23575	-2.92406	1.03592
H	4.75302	1.06537	0.38297
H	6.61191	-0.46871	0.99506
C	0.56269	3.05177	-0.36638
C	0.51443	3.63529	0.90523
C	1.77428	3.05823	-1.07314
C	1.65430	4.22024	1.46187
H	-0.42095	3.63350	1.45850
C	2.91616	3.63715	-0.51726
H	1.81776	2.61371	-2.06389
C	2.85705	4.22035	0.75243
H	1.60285	4.67415	2.44707
H	3.84707	3.64297	-1.07657
H	3.74349	4.67646	1.18322
O	1.55196	-3.69769	-0.20788
C	-1.59012	0.22300	-1.37536
H	-2.09781	0.77834	-2.17201
O	-3.84185	-0.62847	2.23334
C	-4.54003	-0.82132	3.50731
H	-4.13060	-1.68449	4.03113
H	-4.39255	0.10086	4.06731
H	-5.58972	-0.95970	3.25260

H	-2.64028	-2.25543	2.51735
H	-1.76486	-2.47597	-3.11425

PCb-*E*

Zero-point correction= 0.422872 (Hartree/Particle)

Thermal correction to Energy= 0.449687

Thermal correction to Enthalpy= 0.450632

Thermal correction to Gibbs Free Energy= 0.365249

Sum of electronic and thermal Free Energies= -1416.849344

C	1.51195	-1.69294	0.08791
H	1.30568	-2.49975	0.78586
C	4.98422	0.23157	-0.47102
H	5.83904	0.19929	-1.15712
C	1.10194	-0.58844	-2.17642
H	0.22200	-0.37859	-2.79542
C	1.51035	0.71578	-1.47033
H	1.85525	1.44877	-2.20228
C	2.33690	-0.68718	0.46213
C	2.60210	0.52053	-0.40916
H	2.68033	1.42037	0.20689
C	2.90172	-0.66810	1.78303
O	0.32609	1.20121	-0.81151
O	-0.65666	-1.58513	-0.83911
O	2.18081	-1.09337	-2.95253
O	3.85312	0.39596	-1.15608
O	5.07363	0.12594	0.73996
C	0.17869	2.54284	-0.66754
C	-1.33610	-2.84494	-0.63151
H	-1.29936	-3.43052	-1.55821
H	-0.82842	-3.41361	0.15688
C	-1.10433	2.90130	-0.01624
C	-1.37550	4.25993	0.20668
C	-2.03763	1.92750	0.37361
C	-2.56898	4.64102	0.81382
H	-0.64820	5.00421	-0.09864
C	-3.22993	2.31566	0.98053
H	-1.83264	0.87771	0.20067
C	-3.49683	3.66965	1.20133
H	-2.77661	5.69248	0.98509
H	-3.94966	1.55978	1.27907
H	-4.42727	3.96824	1.67477
C	-2.75687	-2.54060	-0.23774
C	-3.04740	-2.09578	1.06037
C	-3.79878	-2.66032	-1.16508
C	-4.35628	-1.77603	1.42349
H	-2.24432	-2.00536	1.78702

C	-5.11097	-2.34764	-0.80213
H	-3.58025	-3.00258	-2.17303
C	-5.39119	-1.90288	0.49145
H	-4.57098	-1.43839	2.43312
H	-5.91174	-2.44982	-1.52854
H	-6.41098	-1.65979	0.77482
O	1.02257	3.34309	-1.04113
C	0.72606	-1.70901	-1.19223
H	0.90310	-2.66151	-1.70894
O	3.03416	-1.74808	2.44142
C	3.51469	-1.71677	3.82536
H	3.70595	-0.68796	4.12911
H	2.72950	-2.17553	4.42442
H	4.42096	-2.32008	3.84077
H	3.16353	0.27369	2.26228
H	2.43821	-0.40891	-3.58855

SM-*Z*

Zero-point correction= 0.415663 (Hartree/Particle)

Thermal correction to Energy= 0.439897

Thermal correction to Enthalpy= 0.440841

Thermal correction to Gibbs Free Energy= 0.361175

Sum of electronic and thermal Free Energies= -1416.414990

C	2.36969	-1.64902	0.58302
H	2.16771	-2.40909	1.33907
C	4.09934	-0.92835	-0.86010
H	5.12244	-1.14058	-1.16839
C	1.88875	-0.77826	-1.69328
H	1.33317	-0.87987	-2.62696
C	1.84798	0.67108	-1.18126
H	2.25131	1.33447	-1.94768
C	2.26784	-0.24813	1.11390
C	2.72406	0.74944	0.08568
H	2.78673	1.76734	0.46756
C	1.81962	0.03836	2.34098
H	1.51973	-0.74549	3.03493
O	0.48670	1.03800	-0.89583
O	0.11677	-1.76685	-0.23224
O	3.28074	-1.04475	-2.00873
O	4.07047	0.38673	-0.35024
O	3.73972	-1.87343	0.12094
O	1.68521	1.31774	2.78607
C	0.18115	2.35768	-0.92585
C	-0.82215	-2.31704	-1.17511
H	-0.92256	-1.65946	-2.04642
H	-0.44200	-3.28714	-1.52641

C	-1.24125	2.62050	-0.59470
C	-1.71085	3.93894	-0.69464
C	-2.11067	1.59710	-0.18699
C	-3.03909	4.22992	-0.39536
H	-1.03033	4.72273	-1.00874
C	-3.43855	1.89474	0.11241
H	-1.74719	0.58055	-0.09743
C	-3.90417	3.20825	0.00766
H	-3.40036	5.25026	-0.47588
H	-4.10887	1.10029	0.42522
H	-4.93993	3.43612	0.24074
C	-2.15576	-2.48978	-0.49206
C	-2.25608	-3.25106	0.68189
C	-3.31296	-1.91154	-1.02640
C	-3.48953	-3.42913	1.30736
H	-1.36275	-3.70121	1.10535
C	-4.55220	-2.09586	-0.40551
H	-3.24389	-1.31624	-1.93274
C	-4.64221	-2.85326	0.76349
H	-3.55381	-4.02009	2.21635
H	-5.44273	-1.64553	-0.83405
H	-5.60317	-2.99521	1.24912
O	0.99974	3.22280	-1.20278
C	1.35630	1.44227	4.17896
H	1.22992	2.50826	4.37066
H	0.42328	0.91598	4.40612
H	2.16339	1.05005	4.80635
C	1.46880	-1.84330	-0.65184
H	1.67678	-2.82928	-1.08772

IM1A-*Z*

Zero-point correction= 0.451733 (Hartree/Particle)

Thermal correction to Energy= 0.478782

Thermal correction to Enthalpy= 0.479726

Thermal correction to Gibbs Free Energy= 0.393928

Sum of electronic and thermal Free Energies= -1493.288021

C	2.01337	-1.79275	0.38681
H	1.82366	-2.55025	1.14738
C	3.68587	-1.19073	-1.17693
H	4.67863	-1.44097	-1.54629
C	1.43901	-0.87343	-1.84751
H	0.82589	-0.93403	-2.74714
C	1.51269	0.57055	-1.32623
H	1.90940	1.21752	-2.10979
C	2.03263	-0.39697	0.93665
C	2.45499	0.59306	-0.10659

H	2.61019	1.60152	0.27151
C	1.71277	-0.10242	2.20385
H	1.42349	-0.88034	2.90868
O	0.19924	1.00975	-0.95303
O	-0.28473	-1.77582	-0.28338
O	2.79503	-1.20930	-2.25635
O	3.76495	0.15795	-0.65324
O	3.34299	-2.08911	-0.16476
O	1.70336	1.16950	2.67189
C	-0.03869	2.34657	-0.98252
C	-1.31664	-2.22327	-1.18462
H	-1.44581	-1.50690	-2.00396
H	-1.01122	-3.18522	-1.62009
C	-1.42350	2.68498	-0.57512
C	-1.83510	4.02256	-0.67858
C	-2.31437	1.71320	-0.09370
C	-3.12832	4.38330	-0.31024
H	-1.13860	4.76600	-1.05047
C	-3.60656	2.08108	0.27527
H	-1.99467	0.68228	-0.00183
C	-4.01511	3.41312	0.16623
H	-3.44562	5.41784	-0.39429
H	-4.29396	1.32702	0.64558
H	-5.02355	3.69559	0.45313
C	-2.60399	-2.37808	-0.41479
C	-2.66346	-3.22689	0.70040
C	-3.76009	-1.69579	-0.81085
C	-3.85624	-3.38860	1.40416
H	-1.77049	-3.75919	1.01574
C	-4.95919	-1.86260	-0.11129
H	-3.72152	-1.03291	-1.67087
C	-5.00853	-2.70746	0.99862
H	-3.88971	-4.04850	2.26618
H	-5.85005	-1.33102	-0.43263
H	-5.93819	-2.83677	1.54486
O	0.80889	3.16108	-1.31574
C	1.44698	1.30016	4.08136
H	1.42553	2.36998	4.28974
H	0.48132	0.85507	4.34085
H	2.24300	0.82710	4.66482
C	1.02884	-1.92519	-0.78993
H	1.15328	-2.91640	-1.24521
O	5.58530	0.46423	0.99887
H	6.45467	0.46175	0.55589
H	5.57720	-0.28369	1.62598
H	4.81293	0.31180	0.27162

IM1B-*Z*

Zero-point correction= 0.451598 (Hartree/Particle)

Thermal correction to Energy= 0.478723
Thermal correction to Enthalpy= 0.479668
Thermal correction to Gibbs Free Energy= 0.393655
Sum of electronic and thermal Free Energies= -1493.288589

C	2.31291	-1.26807	0.24254
H	2.29755	-2.09011	0.95795
C	3.81332	-0.21404	-1.33245
H	4.82560	-0.27771	-1.72704
C	1.53025	-0.35988	-1.93725
H	0.91490	-0.49305	-2.82764
C	1.34292	1.04162	-1.33530
H	1.58524	1.79222	-2.08878
C	2.09989	0.07806	0.86017
C	2.30956	1.17739	-0.14221
H	2.27464	2.17422	0.29309
C	1.78475	0.23908	2.15265
H	1.67333	-0.61516	2.81884
O	-0.02225	1.19765	-0.91879
O	0.05170	-1.66048	-0.40286
O	2.91404	-0.41772	-2.38570
O	3.65393	1.02865	-0.71170
O	3.66957	-1.28466	-0.37026
O	1.56316	1.46057	2.69510
C	-0.51193	2.46194	-0.86540
C	-0.87681	-2.31138	-1.29350
H	-1.13424	-1.64909	-2.12774
H	-0.39513	-3.20813	-1.70839
C	-1.92767	2.50548	-0.42564
C	-2.57996	3.74789	-0.42120
C	-2.61626	1.35166	-0.02062
C	-3.91048	3.83431	-0.02065
H	-2.03794	4.63338	-0.73476
C	-3.94730	1.44497	0.38058
H	-2.11139	0.39326	-0.01379
C	-4.59538	2.68314	0.37976
H	-4.41380	4.79592	-0.02070
H	-4.47810	0.55032	0.69071
H	-5.63321	2.75158	0.69160
C	-2.11456	-2.68614	-0.51869
C	-2.01872	-3.50395	0.61697
C	-3.37589	-2.24023	-0.92990
C	-3.16284	-3.86571	1.32715
H	-1.04345	-3.85326	0.94397
C	-4.52529	-2.60901	-0.22426
H	-3.45801	-1.60308	-1.80616
C	-4.42023	-3.42046	0.90659
H	-3.07621	-4.49853	2.20558
H	-5.49835	-2.26036	-0.55747

H	-5.31135	-3.70565	1.45793
O	0.15592	3.44311	-1.15685
C	1.31506	1.46669	4.11240
H	1.13268	2.50628	4.38504
H	0.43429	0.86313	4.35275
H	2.18504	1.08976	4.65909
C	1.35562	-1.52505	-0.93512
H	1.66068	-2.44814	-1.44429
O	5.45172	-1.26995	1.35048
H	6.32848	-1.30172	0.92289
H	4.69195	-1.27153	0.59189
H	5.39887	-0.42167	1.83044

IM1C-*Z*

Zero-point correction= 0.451597 (Hartree/Particle)

Thermal correction to Energy= 0.478754

Thermal correction to Enthalpy= 0.479699

Thermal correction to Gibbs Free Energy= 0.393589

Sum of electronic and thermal Free Energies= -1493.287709

C	2.34358	-0.88393	1.17631
H	2.22002	-1.57973	2.00639
C	4.06880	0.05537	-0.14439
H	5.14055	0.04262	-0.33382
C	1.97059	-0.41680	-1.23905
H	1.58228	-0.74909	-2.20214
C	1.57054	1.03775	-0.94275
H	1.92601	1.68035	-1.74941
C	1.88101	0.50680	1.49270
C	2.23523	1.45624	0.38481
H	2.03385	2.49923	0.62172
C	1.23572	0.82365	2.62179
H	1.02723	0.07489	3.38446
O	0.14065	1.11534	-0.85602
O	0.29285	-1.60006	0.16420
O	3.43089	-0.37497	-1.37708
O	3.67746	1.36601	0.13600
O	3.78240	-0.84554	0.88297
O	0.78737	2.08057	2.87233
C	-0.42657	2.32109	-1.11788
C	-0.38038	-2.46606	-0.77211
H	-0.49284	-1.96785	-1.74173
H	0.23304	-3.36570	-0.92185
C	-1.90223	2.29852	-0.97494
C	-2.61788	3.45227	-1.32998
C	-2.58564	1.17054	-0.49513
C	-4.00478	3.47525	-1.21152

H	-2.08029	4.31909	-1.69833
C	-3.97365	1.20059	-0.37710
H	-2.03420	0.28278	-0.20998
C	-4.68373	2.34970	-0.73536
H	-4.55633	4.36788	-1.48918
H	-4.49959	0.32601	-0.00734
H	-5.76542	2.36897	-0.64263
C	-1.73030	-2.83264	-0.20969
C	-1.82822	-3.46699	1.03767
C	-2.90112	-2.55973	-0.92630
C	-3.07343	-3.81915	1.55684
H	-0.92458	-3.68128	1.60131
C	-4.15026	-2.91970	-0.41138
H	-2.83411	-2.06470	-1.89119
C	-4.23842	-3.54757	0.83213
H	-3.13681	-4.30904	2.52408
H	-5.05049	-2.70642	-0.98019
H	-5.20775	-3.82528	1.23546
O	0.22691	3.30257	-1.43820
C	0.18571	2.26757	4.16469
H	-0.14955	3.30442	4.19856
H	-0.67214	1.59972	4.29243
H	0.91503	2.09121	4.96173
C	1.67114	-1.42218	-0.10127
H	2.13655	-2.37951	-0.37049
O	4.43262	-2.34900	-2.51754
H	5.03491	-2.82786	-1.91706
H	4.96746	-2.02853	-3.26894
H	4.02369	-1.51498	-2.00360

TS1A-*Z*

Zero-point correction= 0.449122 (Hartree/Particle)

Thermal correction to Energy= 0.476843

Thermal correction to Enthalpy= 0.477787

Thermal correction to Gibbs Free Energy= 0.390362

Sum of electronic and thermal Free Energies= -1493.283562

C	2.07964	-1.72952	0.18112
H	1.98551	-2.56724	0.86888
C	3.67090	-1.16483	-1.50285
H	4.69143	-1.18981	-1.87028
C	1.39322	-0.66393	-1.93778
H	0.76875	-0.67696	-2.82939
C	1.44515	0.73826	-1.30911
H	1.81193	1.44665	-2.05321
C	2.10644	-0.39711	0.84682
C	2.38914	0.75043	-0.08828

H	2.34272	1.71759	0.41399
C	1.94663	-0.28237	2.17683
H	1.77172	-1.15873	2.79935
O	0.11702	1.09566	-0.90994
O	-0.22475	-1.75024	-0.40951
O	2.76585	-0.90004	-2.42475
O	3.72649	0.59874	-0.67505
O	3.43155	-1.92011	-0.46217
O	1.96813	0.90922	2.80454
C	-0.19146	2.42166	-0.91809
C	-1.26265	-2.27863	-1.26733
H	-1.43872	-1.60038	-2.10931
H	-0.92464	-3.24366	-1.66839
C	-1.58034	2.68374	-0.47344
C	-2.04742	4.00649	-0.51746
C	-2.42137	1.65834	-0.01412
C	-3.34584	4.29980	-0.11022
H	-1.38858	4.79120	-0.87275
C	-3.71934	1.95900	0.39335
H	-2.06028	0.63793	0.02848
C	-4.18278	3.27657	0.34501
H	-3.70577	5.32302	-0.14721
H	-4.36841	1.16381	0.74626
H	-5.19539	3.50645	0.66244
C	-2.52005	-2.45227	-0.45582
C	-2.53151	-3.30888	0.65500
C	-3.69532	-1.77797	-0.80662
C	-3.69665	-3.48518	1.40018
H	-1.62348	-3.83664	0.93322
C	-4.86692	-1.95990	-0.06560
H	-3.69326	-1.11107	-1.66433
C	-4.86840	-2.81164	1.04019
H	-3.69399	-4.15090	2.25828
H	-5.77379	-1.43555	-0.35200
H	-5.77685	-2.95301	1.61819
O	0.60581	3.28073	-1.26019
C	1.72916	0.86651	4.22523
H	1.79911	1.89632	4.57470
H	0.73063	0.47149	4.43365
H	2.48663	0.25422	4.72325
C	1.06914	-1.81652	-0.96857
H	1.21507	-2.76233	-1.50548
O	5.43649	0.41481	1.27433
H	6.32370	0.40033	0.88285
H	5.29758	-0.49513	1.58065
H	4.40940	0.54992	0.07945

TS1B-*Z*

Zero-point correction= 0.449234 (Hartree/Particle)

Thermal correction to Energy= 0.476933
Thermal correction to Enthalpy= 0.477877
Thermal correction to Gibbs Free Energy= 0.390650
Sum of electronic and thermal Free Energies= -1493.282819

C	2.20403	-1.43041	0.30035
H	1.96414	-2.14654	1.09104
C	3.78214	-0.27675	-1.64573
H	4.78406	-0.55183	-1.95857
C	1.43783	-0.52902	-1.94086
H	0.81240	-0.67404	-2.81971
C	1.34805	0.90190	-1.40639
H	1.60230	1.60818	-2.19851
C	2.14740	-0.01119	0.80427
C	2.34920	1.02589	-0.24959
H	2.42127	2.04176	0.12895
C	1.93397	0.27386	2.10075
H	1.78289	-0.52156	2.82932
O	0.01845	1.14004	-0.94552
O	-0.06781	-1.69574	-0.37372
O	2.82213	-0.69031	-2.43098
O	3.69641	0.76398	-0.87104
O	3.53298	-1.73371	-0.23016
O	1.86382	1.53761	2.56393
C	-0.41327	2.43381	-0.95528
C	-1.03006	-2.32644	-1.24723
H	-1.26825	-1.66778	-2.08974
H	-0.58660	-3.24829	-1.64748
C	-1.80101	2.56981	-0.45711
C	-2.38839	3.84419	-0.48593
C	-2.52651	1.47383	0.03553
C	-3.69189	4.01944	-0.03020
H	-1.81779	4.68380	-0.86753
C	-3.83057	1.65655	0.49041
H	-2.07218	0.49101	0.06696
C	-4.41400	2.92607	0.45756
H	-4.14514	5.00527	-0.05472
H	-4.39087	0.80693	0.86777
H	-5.43088	3.06386	0.81244
C	-2.27313	-2.63497	-0.45313
C	-2.21135	-3.50444	0.64592
C	-3.50460	-2.07052	-0.80483
C	-3.35996	-3.80205	1.37807
H	-1.25923	-3.94708	0.92519
C	-4.65908	-2.37356	-0.07663
H	-3.55948	-1.39324	-1.65264
C	-4.58788	-3.23814	1.01682
H	-3.30044	-4.47710	2.22679
H	-5.60956	-1.93349	-0.36352

H	-5.48306	-3.47382	1.58471
O	0.28875	3.35496	-1.33890
C	1.69009	1.66524	3.98879
H	1.61793	2.73372	4.19066
H	0.77153	1.16474	4.30883
H	2.55016	1.24533	4.51868
C	1.24704	-1.65254	-0.89024
H	1.50537	-2.60263	-1.37444
O	5.31380	-1.08931	1.62858
H	5.72691	-0.31561	1.21422
H	4.21105	-1.53395	0.48655
H	4.73708	-0.70111	2.30540

TS1C-*Z*

Zero-point correction= 0.448739 (Hartree/Particle)

Thermal correction to Energy= 0.476414

Thermal correction to Enthalpy= 0.477358

Thermal correction to Gibbs Free Energy= 0.390076

Sum of electronic and thermal Free Energies= -1493.284035

C	-2.39542	0.80158	1.07955
H	-2.36126	1.51460	1.90066
C	-4.17335	-0.30476	-0.05695
H	-5.19875	-0.29707	-0.41339
C	-1.90385	0.35983	-1.36352
H	-1.33683	0.69356	-2.23713
C	-1.50687	-1.08608	-1.00945
H	-1.83114	-1.75303	-1.80971
C	-1.90095	-0.55674	1.42760
C	-2.16299	-1.51814	0.31965
H	-1.94656	-2.55503	0.56193
C	-1.32228	-0.84728	2.60350
H	-1.18897	-0.08138	3.36600
O	-0.08119	-1.13496	-0.87955
O	-0.37690	1.60730	0.12029
O	-3.31045	0.26749	-1.71308
O	-3.62611	-1.49513	0.02001
O	-3.85066	0.69578	0.72500
O	-0.85494	-2.07749	2.88905
C	0.52196	-2.33220	-1.11118
C	0.20352	2.70733	-0.61659
H	0.22963	2.47449	-1.68697
H	-0.42913	3.59372	-0.47417
C	1.99530	-2.25990	-0.97131
C	2.74035	-3.42094	-1.22907
C	2.64918	-1.07662	-0.59388
C	4.12732	-3.39771	-1.11259

H	2.22497	-4.32979	-1.51968
C	4.03744	-1.06046	-0.47851
H	2.07529	-0.18082	-0.38894
C	4.77680	-2.21780	-0.73757
H	4.70174	-4.29638	-1.31373
H	4.54078	-0.14361	-0.18817
H	5.85869	-2.20087	-0.64682
C	1.59411	2.95385	-0.09346
C	1.78404	3.37961	1.22968
C	2.71221	2.76343	-0.91362
C	3.06820	3.60892	1.72194
H	0.92090	3.53061	1.87220
C	4.00045	3.00087	-0.42465
H	2.57256	2.43345	-1.93943
C	4.18004	3.42149	0.89433
H	3.20338	3.93877	2.74781
H	4.85940	2.85743	-1.07347
H	5.17990	3.60513	1.27633
O	-0.10543	-3.33853	-1.40249
C	-0.29691	-2.23768	4.20863
H	0.05826	-3.26642	4.26350
H	0.53929	-1.54820	4.35634
H	-1.06250	-2.06888	4.97141
C	-1.73691	1.36604	-0.19354
H	-2.24249	2.29977	-0.46785
O	-4.28148	2.60172	-2.28743
H	-4.77723	2.83227	-1.48618
H	-4.96648	2.47071	-2.96166
H	-3.67472	1.18773	-1.95718

IM2A-*Z*

Zero-point correction= 0.424685 (Hartree/Particle)

Thermal correction to Energy= 0.450697

Thermal correction to Enthalpy= 0.451641

Thermal correction to Gibbs Free Energy= 0.368629

Sum of electronic and thermal Free Energies= -1416.833956

C	2.57695	-1.37545	-0.14232
H	2.66697	-2.37311	0.28016
C	4.05863	-0.42626	-1.83431
H	5.04912	-0.28251	-2.26200
C	1.71526	0.10274	-1.90145
H	1.08969	0.23806	-2.78174
C	1.54312	1.27838	-0.92396
H	1.87093	2.19519	-1.41671
C	2.42372	-0.30798	0.86937
C	2.34302	1.12234	0.38726

H	1.86589	1.74690	1.14827
C	2.41545	-0.65053	2.17483
H	2.51641	-1.68888	2.48783
O	0.13616	1.35451	-0.65389
O	0.24595	-1.46201	-0.84202
O	3.10275	0.19920	-2.42147
O	3.65527	1.63485	0.07417
O	3.95862	-1.20537	-0.83849
O	2.26716	0.25831	3.15107
C	-0.38282	2.59139	-0.42628
C	-0.14796	-2.85084	-0.69464
H	0.04967	-3.36834	-1.64213
H	0.44661	-3.32737	0.09203
C	-1.84337	2.55513	-0.17708
C	-2.49894	3.77042	0.07419
C	-2.57169	1.35525	-0.18720
C	-3.87039	3.78510	0.31228
H	-1.92690	4.69169	0.08036
C	-3.94389	1.37694	0.05255
H	-2.06819	0.41554	-0.38012
C	-4.59376	2.58876	0.30205
H	-4.37538	4.72608	0.50620
H	-4.50390	0.44712	0.04485
H	-5.66332	2.60134	0.48880
C	-1.61205	-2.88968	-0.34932
C	-2.03579	-3.26828	0.92993
C	-2.57150	-2.53177	-1.30815
C	-3.39593	-3.29617	1.24746
H	-1.29715	-3.54473	1.67743
C	-3.92999	-2.55191	-0.99113
H	-2.25015	-2.24172	-2.30486
C	-4.34460	-2.93592	0.28853
H	-3.71275	-3.59616	2.24188
H	-4.66499	-2.27820	-1.74219
H	-5.40242	-2.95843	0.53323
O	0.30097	3.60278	-0.43185
C	2.17772	-0.27064	4.49077
H	2.11064	0.59288	5.15178
H	1.28291	-0.89031	4.59582
H	3.07119	-0.85391	4.73027
C	1.56801	-1.29443	-1.29577
H	1.81971	-2.03812	-2.06266
H	4.21538	1.46920	0.84758

IM2B-*Z*

Zero-point correction= 0.425179 (Hartree/Particle)

Thermal correction to Energy= 0.450904

Thermal correction to Enthalpy= 0.451849

Thermal correction to Gibbs Free Energy= 0.369620
Sum of electronic and thermal Free Energies= -1416.830152

C	2.14237	-1.67409	0.80311
H	1.62018	-2.08624	1.67355
C	3.99948	-0.84720	-1.63498
H	4.95918	-1.20307	-2.00291
C	1.60484	-1.05422	-1.63572
H	0.96126	-1.26340	-2.48808
C	1.67890	0.43962	-1.32367
H	1.99711	0.98984	-2.21197
C	2.39461	-0.20012	1.00635
C	2.70178	0.61426	-0.19787
H	2.91601	1.65742	0.01535
C	2.35185	0.33923	2.24140
H	2.08085	-0.27057	3.10250
O	0.39337	0.88652	-0.90602
O	-0.08707	-1.82476	-0.07835
O	2.96302	-1.42792	-2.11569
O	4.01778	0.12103	-0.80961
O	3.37982	-2.38503	0.57875
O	2.61536	1.63483	2.48211
C	0.11908	2.21377	-1.08266
C	-0.99221	-2.69336	-0.79793
H	-1.00270	-2.43521	-1.86288
H	-0.63513	-3.72680	-0.69764
C	-1.25232	2.56633	-0.65321
C	-1.66057	3.90281	-0.78601
C	-2.13725	1.61045	-0.12994
C	-2.94365	4.27895	-0.39954
H	-0.96879	4.63298	-1.19132
C	-3.42059	1.99382	0.25301
H	-1.82270	0.57936	-0.02425
C	-3.82444	3.32507	0.11936
H	-3.25796	5.31261	-0.50260
H	-4.10521	1.25233	0.65241
H	-4.82559	3.61968	0.41920
C	-2.36727	-2.54012	-0.20349
C	-2.62396	-2.98953	1.10036
C	-3.40039	-1.93850	-0.93151
C	-3.89030	-2.83944	1.66421
H	-1.82703	-3.45896	1.67076
C	-4.67266	-1.79293	-0.37081
H	-3.20839	-1.58731	-1.94172
C	-4.91836	-2.24098	0.92853
H	-4.07836	-3.19227	2.67396
H	-5.46792	-1.33053	-0.94787
H	-5.90610	-2.12878	1.36579
O	0.93658	2.99136	-1.54545
C	2.49382	2.05175	3.85782

H	2.74188	3.11260	3.87409
H	1.46976	1.90406	4.21186
H	3.19458	1.49676	4.48789
C	1.27489	-1.97808	-0.43726
H	1.48055	-3.01007	-0.74442
H	3.98785	-2.11758	1.28498

IM2C-*Z*

Zero-point correction= 0.424920 (Hartree/Particle)

Thermal correction to Energy= 0.450724

Thermal correction to Enthalpy= 0.451668

Thermal correction to Gibbs Free Energy= 0.369083

Sum of electronic and thermal Free Energies= -1416.834398

C	2.39127	-1.54807	0.52192
H	2.26507	-2.38050	1.21006
C	4.48268	-0.58115	-0.20414
H	5.52817	-0.69740	-0.48423
C	1.75040	-0.62520	-1.79538
H	0.89888	-0.65494	-2.48519
C	1.78175	0.78501	-1.16377
H	2.19108	1.49957	-1.88127
C	2.18557	-0.21025	1.11789
C	2.57644	0.85035	0.15751
H	2.58615	1.85706	0.56644
C	1.74936	-0.01991	2.37606
H	1.50880	-0.86743	3.01597
O	0.42475	1.13552	-0.84489
O	0.23130	-1.85496	-0.31754
O	2.97531	-0.88123	-2.48117
O	4.02451	0.61254	-0.21372
O	3.85775	-1.64339	0.12383
O	1.55877	1.20420	2.88706
C	0.08177	2.45102	-0.87056
C	-0.58803	-2.76235	-1.09144
H	-0.63707	-2.42656	-2.13362
H	-0.12514	-3.75733	-1.07017
C	-1.35307	2.66104	-0.56558
C	-1.84605	3.97473	-0.57504
C	-2.21386	1.59051	-0.27565
C	-3.18907	4.21533	-0.29834
H	-1.17198	4.79392	-0.80030
C	-3.55654	1.83841	0.00018
H	-1.83402	0.57582	-0.26513
C	-4.04503	3.14787	-0.01097
H	-3.56900	5.23194	-0.30662
H	-4.22014	1.00846	0.22194

H	-5.09240	3.33693	0.20416
C	-1.96189	-2.79325	-0.47645
C	-2.17371	-3.45983	0.73913
C	-3.03764	-2.14071	-1.09093
C	-3.43774	-3.47383	1.32833
H	-1.34380	-3.97007	1.22071
C	-4.30668	-2.15765	-0.50515
H	-2.88121	-1.62347	-2.03367
C	-4.50746	-2.82285	0.70610
H	-3.59106	-3.99537	2.26851
H	-5.13529	-1.65559	-0.99569
H	-5.49299	-2.83814	1.16204
O	0.88431	3.33626	-1.12231
C	1.07815	1.24695	4.24813
H	0.97659	2.30270	4.49704
H	0.10669	0.75106	4.32312
H	1.79815	0.77312	4.92084
C	1.57513	-1.76831	-0.76829
H	1.89659	-2.70253	-1.24279
H	3.06985	-0.21994	-3.18363

TS2A-*Z*

Zero-point correction= 0.423507 (Hartree/Particle)

Thermal correction to Energy= 0.449281

Thermal correction to Enthalpy= 0.450226

Thermal correction to Gibbs Free Energy= 0.367612

Sum of electronic and thermal Free Energies= -1416.832836

C	2.40319	-1.43714	-0.04304
H	2.60192	-2.40463	0.40762
C	3.90759	-0.67209	-2.10580
H	4.81483	-0.52458	-2.69548
C	1.56373	-0.09255	-1.93644
H	0.84569	-0.02544	-2.75283
C	1.45967	1.15582	-1.04318
H	1.76889	2.02827	-1.62084
C	2.48320	-0.29494	0.80200
C	2.32718	1.10315	0.23115
H	1.86951	1.75909	0.97808
C	2.83112	-0.51027	2.10834
H	3.00955	-1.51723	2.48525
O	0.06827	1.27601	-0.70418
O	0.10199	-1.53982	-0.71509
O	2.87728	-0.03915	-2.59805
O	3.60582	1.62525	-0.16916
O	3.93275	-1.39660	-1.09435
O	2.93897	0.48616	2.96539

C	-0.41611	2.53269	-0.52247
C	-0.34316	-2.91156	-0.54069
H	-0.21027	-3.44247	-1.49131
H	0.26563	-3.40579	0.22388
C	-1.86605	2.54253	-0.21364
C	-2.48000	3.78164	0.02594
C	-2.62481	1.36285	-0.15987
C	-3.84003	3.83996	0.31717
H	-1.88463	4.68700	-0.01748
C	-3.98562	1.42828	0.13176
H	-2.15376	0.40486	-0.34488
C	-4.59379	2.66371	0.37025
H	-4.31262	4.79930	0.50262
H	-4.56930	0.51405	0.17258
H	-5.65457	2.71037	0.59738
C	-1.79047	-2.88231	-0.13232
C	-2.15958	-3.06489	1.20612
C	-2.78771	-2.64318	-1.08899
C	-3.50299	-3.01540	1.58416
H	-1.39092	-3.24932	1.95174
C	-4.13049	-2.58929	-0.71319
H	-2.50817	-2.50527	-2.13006
C	-4.49011	-2.77655	0.62531
H	-3.77814	-3.16351	2.62414
H	-4.89575	-2.41072	-1.46267
H	-5.53556	-2.74187	0.91717
O	0.28645	3.52796	-0.60898
C	3.20648	0.13740	4.34889
H	3.38607	1.08047	4.86195
H	2.33553	-0.36666	4.77279
H	4.08985	-0.50201	4.40604
C	1.42590	-1.42792	-1.20268
H	1.65342	-2.24432	-1.89611
H	4.17771	1.62228	0.61311

TS2B-*Z*

Zero-point correction= 0.422745 (Hartree/Particle)

Thermal correction to Energy= 0.448684

Thermal correction to Enthalpy= 0.449628

Thermal correction to Gibbs Free Energy= 0.366727

Sum of electronic and thermal Free Energies= -1416.827282

C	2.12050	-1.72980	0.57764
H	1.62284	-2.20454	1.43025
C	3.86858	-0.66348	-2.04210
H	4.71712	-1.06296	-2.60290
C	1.46684	-0.84122	-1.75343

H	0.72625	-0.92630	-2.54790
C	1.56187	0.60581	-1.27407
H	1.89278	1.25661	-2.08378
C	2.45968	-0.28706	0.91520
C	2.51194	0.69097	-0.10252
H	2.86550	1.68415	0.15144
C	2.82818	0.02453	2.20624
H	2.73892	-0.72273	2.99602
O	0.25429	1.01371	-0.84312
O	-0.15196	-1.75766	-0.18235
O	2.73336	-1.20209	-2.40933
O	4.04129	0.21862	-1.18583
O	3.31358	-2.45445	0.23079
O	3.26416	1.21196	2.55464
C	-0.00926	2.35352	-0.88039
C	-1.11059	-2.51722	-0.95097
H	-1.20958	-2.10240	-1.96051
H	-0.74497	-3.54913	-1.03994
C	-1.39125	2.66772	-0.45383
C	-1.80126	4.00985	-0.48663
C	-2.28286	1.67136	-0.02628
C	-3.09322	4.35123	-0.09734
H	-1.10426	4.77160	-0.81813
C	-3.57433	2.02051	0.36189
H	-1.96624	0.63593	0.00514
C	-3.98047	3.35727	0.32660
H	-3.40931	5.38910	-0.12417
H	-4.26300	1.24812	0.68927
H	-4.98824	3.62503	0.62938
C	-2.43602	-2.48148	-0.23571
C	-2.57155	-3.07726	1.02712
C	-3.54721	-1.85919	-0.81643
C	-3.79524	-3.05015	1.69484
H	-1.71406	-3.56357	1.48416
C	-4.77725	-1.83725	-0.15207
H	-3.44966	-1.39475	-1.79388
C	-4.90218	-2.43105	1.10515
H	-3.88911	-3.51579	2.67148
H	-5.63367	-1.35784	-0.61685
H	-5.85647	-2.41482	1.62322
O	0.82393	3.17267	-1.23080
C	3.50704	1.44626	3.96848
H	4.07109	2.37557	4.01953
H	2.54998	1.55085	4.48298
H	4.08701	0.62213	4.38775
C	1.18669	-1.87837	-0.63834
H	1.35963	-2.87498	-1.06084
H	3.93644	-2.36460	0.96789

TS2Ca-*Z*

Zero-point correction= 0.422168 (Hartree/Particle)

Thermal correction to Energy= 0.448177

Thermal correction to Enthalpy= 0.449121

Thermal correction to Gibbs Free Energy= 0.366116

Sum of electronic and thermal Free Energies= -1416.824611

C	2.52720	-1.43417	0.08816
H	2.50957	-2.37107	0.64167
C	4.53158	-0.22559	-0.64363
H	5.56373	-0.37769	-0.97458
C	1.53800	-0.22765	-1.95192
H	0.61523	-0.22270	-2.54372
C	1.51730	1.05565	-1.09237
H	1.89152	1.90379	-1.66734
C	2.26636	-0.24244	0.95764
C	2.22059	0.97096	0.24877
H	2.30873	1.91114	0.78525
C	2.33360	-0.32511	2.32869
H	2.45065	-1.29266	2.81699
O	0.13866	1.29503	-0.71801
O	0.29477	-1.77315	-0.52513
O	2.67975	-0.26275	-2.79933
O	4.08557	0.92407	-0.50926
O	3.92270	-1.36671	-0.41957
O	2.21100	0.73088	3.09463
C	-0.29402	2.58627	-0.65499
C	-0.57506	-2.58301	-1.34827
H	-0.77761	-2.07474	-2.29806
H	-0.06691	-3.53140	-1.56646
C	-1.72716	2.67358	-0.29067
C	-2.31559	3.94576	-0.22302
C	-2.49337	1.52977	-0.01488
C	-3.65975	4.07230	0.11636
H	-1.71397	4.82213	-0.43811
C	-3.83750	1.66408	0.32481
H	-2.03996	0.54693	-0.06421
C	-4.42132	2.93227	0.39052
H	-4.11380	5.05672	0.16789
H	-4.42757	0.77808	0.53739
H	-5.46950	3.03272	0.65550
C	-1.85564	-2.81966	-0.59229
C	-1.87139	-3.68333	0.51247
C	-3.03954	-2.17230	-0.96639
C	-3.04915	-3.89615	1.22851
H	-0.95697	-4.19165	0.80670
C	-4.22253	-2.38795	-0.25356
H	-3.03528	-1.50309	-1.82244
C	-4.22794	-3.24851	0.84596

H	-3.05035	-4.56951	2.08060
H	-5.13649	-1.88711	-0.55893
H	-5.14630	-3.41831	1.40022
O	0.43633	3.53748	-0.87866
C	2.26844	0.51621	4.53267
H	2.16190	1.50304	4.97846
H	1.44477	-0.13272	4.83544
H	3.23140	0.07677	4.79838
C	1.56650	-1.51918	-1.10872
H	1.87908	-2.34532	-1.75736
H	2.65018	0.51703	-3.37362

TS2Cb-*Z*

Zero-point correction= 0.422095 (Hartree/Particle)

Thermal correction to Energy= 0.448158

Thermal correction to Enthalpy= 0.449102

Thermal correction to Gibbs Free Energy= 0.365683

Sum of electronic and thermal Free Energies= -1416.828962

C	1.94328	-1.64299	0.60165
H	1.89587	-2.42139	1.35815
C	4.39691	-1.06917	-0.53289
H	5.43070	-1.15641	-0.88178
C	1.39162	-0.94393	-1.83032
H	0.46255	-0.92687	-2.41195
C	1.66583	0.49434	-1.34660
H	2.06027	1.09260	-2.17064
C	2.20776	-0.31892	0.98588
C	2.62354	0.57428	-0.14492
H	2.75413	1.61008	0.16049
C	2.30826	0.00779	2.31609
H	2.08321	-0.73250	3.08353
O	0.40070	1.02972	-0.91436
O	-0.16425	-1.87480	-0.19397
O	2.47674	-1.41828	-2.61975
O	3.97641	0.17824	-0.59947
O	3.78218	-2.06318	-0.12809
O	2.62499	1.21743	2.70392
C	0.20336	2.36908	-1.01802
C	-1.00679	-2.95939	-0.66428
H	-0.99238	-2.96809	-1.76059
H	-0.60294	-3.91067	-0.29984
C	-1.15698	2.76997	-0.58596
C	-1.48874	4.13286	-0.62255
C	-2.10465	1.82901	-0.15258
C	-2.75691	4.55091	-0.22845
H	-0.74964	4.85126	-0.95988

C	-3.37231	2.25390	0.23852
H	-1.85045	0.77614	-0.12307
C	-3.69942	3.61225	0.20189
H	-3.01132	5.60564	-0.25665
H	-4.10398	1.52358	0.56962
H	-4.68841	3.93958	0.50812
C	-2.39799	-2.72419	-0.14443
C	-2.81810	-3.31066	1.05631
C	-3.27942	-1.88227	-0.83724
C	-4.09828	-3.06417	1.55567
H	-2.13972	-3.96525	1.59697
C	-4.55903	-1.63162	-0.33850
H	-2.96154	-1.42864	-1.77221
C	-4.96987	-2.22307	0.85959
H	-4.41555	-3.52870	2.48454
H	-5.23641	-0.98359	-0.88664
H	-5.96696	-2.03312	1.24574
O	1.06453	3.13323	-1.42595
C	2.61072	1.48098	4.13522
H	2.98527	2.49622	4.24846
H	1.58663	1.40581	4.50484
H	3.26416	0.77071	4.64470
C	1.18282	-1.94181	-0.67341
H	1.42797	-2.94636	-1.02856
H	2.52432	-0.86798	-3.41592

PA-*Z*

Zero-point correction= 0.422895 (Hartree/Particle)

Thermal correction to Energy= 0.449829

Thermal correction to Enthalpy= 0.450773

Thermal correction to Gibbs Free Energy= 0.365232

Sum of electronic and thermal Free Energies= -1416.847468

C	1.79303	-1.49080	0.67631
H	1.70769	-2.26271	1.43790
C	3.59767	-1.76836	-2.23633
H	4.28634	-1.84011	-3.08788
C	1.40905	-0.74467	-1.73764
H	0.54710	-0.73985	-2.40540
C	1.60812	0.68939	-1.21528
H	1.96266	1.32692	-2.02617
C	2.38066	-0.29933	0.98076
C	2.57543	0.82385	-0.02888
H	2.36658	1.78013	0.46130
C	2.86413	-0.17132	2.30453
H	2.74296	-0.98085	3.02621
O	0.29453	1.11218	-0.79599

O	-0.28479	-1.69560	-0.31710
O	2.51681	-1.07469	-2.62073
O	3.89944	0.82288	-0.56106
O	3.80376	-2.26043	-1.14666
O	3.45369	0.90117	2.67515
C	-0.00703	2.43163	-0.89459
C	-0.94498	-2.98262	-0.21410
H	-0.86044	-3.49520	-1.18050
H	-0.45022	-3.59304	0.54918
C	-1.40168	2.72397	-0.48277
C	-1.82199	4.06284	-0.47653
C	-2.29639	1.70765	-0.11195
C	-3.12372	4.38203	-0.09970
H	-1.12416	4.84078	-0.76633
C	-3.59863	2.03343	0.26085
H	-1.97623	0.67276	-0.11863
C	-4.01293	3.36814	0.26901
H	-3.44581	5.41845	-0.09350
H	-4.28872	1.24390	0.54182
H	-5.02827	3.61851	0.56132
C	-2.38261	-2.73157	0.15035
C	-2.83761	-2.94855	1.45670
C	-3.27678	-2.23730	-0.81072
C	-4.16512	-2.68219	1.79869
H	-2.14874	-3.33011	2.20555
C	-4.60203	-1.96432	-0.46983
H	-2.93143	-2.07076	-1.82757
C	-5.04839	-2.18717	0.83668
H	-4.50811	-2.85918	2.81372
H	-5.28764	-1.58581	-1.22209
H	-6.08136	-1.98086	1.10085
O	0.79745	3.26538	-1.28197
C	3.94597	1.03922	4.04564
H	5.02345	1.17874	3.96833
H	3.46665	1.93025	4.44848
H	3.69413	0.15063	4.62410
C	1.11560	-1.77589	-0.63084
H	1.36677	-2.77799	-0.98443
H	4.50097	1.11992	0.13857

PB-*Z*

Zero-point correction= 0.422285 (Hartree/Particle)

Thermal correction to Energy= 0.449332

Thermal correction to Enthalpy= 0.450277

Thermal correction to Gibbs Free Energy= 0.364780

Sum of electronic and thermal Free Energies= -1416.840799

C	2.55542	-1.54717	0.02961
H	2.29930	-2.42957	0.62789
C	3.73674	0.62692	-2.49596
H	4.38688	0.50928	-3.37245
C	1.47414	-0.07289	-1.79402
H	0.58888	-0.03888	-2.42943
C	1.37023	1.10314	-0.81006
H	1.74723	2.02364	-1.25498
C	2.51456	-0.31803	0.93111
C	2.01700	0.87809	0.52445
H	2.00258	1.72559	1.20453
C	3.02342	-0.46296	2.25781
H	3.34494	-1.44390	2.61500
O	-0.04560	1.27995	-0.52759
O	0.24699	-1.69911	-0.49824
O	2.56496	0.02924	-2.75298
O	4.05197	1.22274	-1.48684
O	3.83288	-1.71226	-0.58324
O	3.11029	0.52785	3.05355
C	-0.49237	2.55542	-0.36292
C	-0.69873	-2.35167	-1.37063
H	-0.95650	-1.70285	-2.21565
H	-0.23216	-3.26199	-1.77156
C	-1.93969	2.60747	-0.04957
C	-2.53931	3.86830	0.09271
C	-2.70607	1.44317	0.11584
C	-3.89465	3.96297	0.39592
H	-1.93707	4.76103	-0.03561
C	-4.06152	1.54530	0.42032
H	-2.24368	0.46881	0.01255
C	-4.65650	2.80217	0.56011
H	-4.35712	4.93870	0.50560
H	-4.65123	0.64305	0.54898
H	-5.71321	2.87771	0.79814
C	-1.93428	-2.69160	-0.57728
C	-1.84872	-3.54128	0.53557
C	-3.18344	-2.17803	-0.94516
C	-2.99064	-3.86813	1.26599
H	-0.88342	-3.94549	0.82739
C	-4.33108	-2.51146	-0.21928
H	-3.25790	-1.51814	-1.80513
C	-4.23582	-3.35517	0.88861
H	-2.91222	-4.52718	2.12568
H	-5.29518	-2.11218	-0.52017
H	-5.12554	-3.61405	1.45497
O	0.23856	3.52813	-0.46241
C	3.60725	0.35003	4.41934
H	4.49113	0.98109	4.50062
H	2.81263	0.70588	5.07349
H	3.83957	-0.69943	4.59867

C	1.51587	-1.45022	-1.09478
H	1.75823	-2.21389	-1.84229
H	4.48261	-1.90578	0.10931

PCa-*Z*

Zero-point correction= 0.422871 (Hartree/Particle)

Thermal correction to Energy= 0.449617

Thermal correction to Enthalpy= 0.450562

Thermal correction to Gibbs Free Energy= 0.365432

Sum of electronic and thermal Free Energies= -1416.850161

C	2.91519	-0.29685	-0.69461
H	3.40160	-1.25938	-0.51776
C	5.02231	0.78700	-0.99642
H	5.60836	1.37541	-1.71261
C	0.80390	0.75680	-1.71831
H	-0.18836	0.43622	-2.05388
C	0.60694	1.54307	-0.38770
H	0.79503	2.60147	-0.61250
C	2.58239	0.37280	0.61911
C	1.49932	1.17476	0.74699
H	1.23259	1.58432	1.71735
C	3.39369	0.05134	1.75578
H	4.02796	-0.83358	1.75054
O	-0.74099	1.41879	0.12334
O	0.88800	-1.47496	-0.80706
O	1.42541	1.57073	-2.70217
O	5.42402	0.43394	0.09754
O	3.81422	0.52330	-1.50011
O	3.35121	0.77153	2.80324
C	-1.68096	2.18931	-0.48026
C	0.10782	-2.35438	-1.64444
H	-0.38592	-1.76960	-2.43065
H	0.77108	-3.08155	-2.12868
C	-3.03255	2.01048	0.10083
C	-4.09652	2.71982	-0.47719
C	-3.26822	1.16306	1.19571
C	-5.38465	2.58005	0.03197
H	-3.90323	3.37254	-1.32133
C	-4.56010	1.02794	1.70040
H	-2.44802	0.61874	1.64819
C	-5.61765	1.73375	1.11984
H	-6.20599	3.12852	-0.41796
H	-4.74136	0.37509	2.54827
H	-6.62281	1.62535	1.51581
C	-0.92034	-3.04712	-0.78790
C	-0.89610	-4.43456	-0.61230

C	-1.92357	-2.29736	-0.15588
C	-1.86280	-5.06698	0.17506
H	-0.11923	-5.02142	-1.09505
C	-2.88458	-2.92478	0.63514
H	-1.94654	-1.21897	-0.28467
C	-2.85723	-4.31358	0.80080
H	-1.83530	-6.14529	0.30143
H	-3.65665	-2.33165	1.11714
H	-3.60776	-4.80377	1.41394
O	-1.41270	2.93802	-1.40903
C	4.11398	0.39523	3.99649
H	3.37958	0.25775	4.78881
H	4.67871	-0.51680	3.80519
H	4.76655	1.23992	4.21132
C	1.65059	-0.51443	-1.53086
H	1.94485	-0.89011	-2.51664
H	0.79643	2.27383	-2.92551

PCb-*Z*

Zero-point correction= 0.423131 (Hartree/Particle)

Thermal correction to Energy= 0.449880

Thermal correction to Enthalpy= 0.450824

Thermal correction to Gibbs Free Energy= 0.365828

Sum of electronic and thermal Free Energies= -1416.855652

C	1.17604	-2.19194	0.57937
H	0.91626	-2.90495	1.35717
C	4.72545	0.33223	-1.07583
H	5.61758	-0.11113	-1.53483
C	0.85212	-1.31854	-1.78829
H	0.01531	-1.05797	-2.44719
C	1.48919	-0.01725	-1.27839
H	1.93006	0.53600	-2.11076
C	2.20371	-1.32481	0.77487
C	2.57360	-0.24210	-0.20939
H	2.74372	0.69661	0.32057
C	2.92127	-1.45630	1.99607
H	2.64803	-2.22232	2.72351
O	0.42051	0.75376	-0.69482
O	-0.98558	-1.83971	-0.20676
O	1.82971	-2.10185	-2.46307
O	3.82149	-0.63932	-0.84061
O	4.58377	1.50933	-0.82505
O	3.89731	-0.67926	2.24861
C	0.52367	2.10696	-0.71962
C	-2.05461	-2.51638	-0.91578
H	-1.98641	-2.28241	-1.98478

H	-1.93645	-3.59873	-0.78649
C	-0.66386	2.77335	-0.13202
C	-0.66665	4.17448	-0.05570
C	-1.76890	2.04408	0.33497
C	-1.76368	4.83981	0.48491
H	0.19145	4.72852	-0.42022
C	-2.86524	2.71622	0.87082
H	-1.76925	0.96239	0.27608
C	-2.86373	4.11168	0.94788
H	-1.76283	5.92352	0.54511
H	-3.72127	2.15005	1.22433
H	-3.71942	4.63181	1.36773
C	-3.36175	-2.04279	-0.34260
C	-3.94645	-2.71542	0.73883
C	-3.99062	-0.89945	-0.85447
C	-5.14051	-2.25679	1.29715
H	-3.46445	-3.60343	1.13923
C	-5.18407	-0.43704	-0.29628
H	-3.54436	-0.37536	-1.69529
C	-5.76040	-1.11584	0.78086
H	-5.58776	-2.78901	2.13140
H	-5.66622	0.44605	-0.70477
H	-6.69101	-0.75982	1.21281
O	1.49492	2.68670	-1.18107
C	4.63794	-0.79700	3.50708
H	5.67633	-0.96572	3.22610
H	4.52102	0.16042	4.01269
H	4.23787	-1.61991	4.09854
C	0.31925	-2.20792	-0.64938
H	0.31650	-3.24177	-1.02494
H	2.18767	-1.57534	-3.19401

H_2O

Zero-point correction= 0.020877 (Hartree/Particle)

Thermal correction to Energy= 0.023713

Thermal correction to Enthalpy= 0.024657

Thermal correction to Gibbs Free Energy= 0.003212

Sum of electronic and thermal Free Energies= -76.472595

O	-2.66033	0.76010	0.00000
H	-1.70033	0.76010	0.00000
H	-2.98079	1.66503	0.00000

H_3O^+

Zero-point correction= 0.034762 (Hartree/Particle)

Thermal correction to Energy= 0.037629
Thermal correction to Enthalpy= 0.038573
Thermal correction to Gibbs Free Energy= 0.015632
Sum of electronic and thermal Free Energies= -76.876027

O	0.00008	0.00008	0.09398
H	0.00215	0.91694	-0.25078
H	0.79327	-0.46085	-0.25067
H	-0.79609	-0.45675	-0.25038

(±)-**8**
Zero-point correction= 0.397286 (Hartree/Particle)
Thermal correction to Energy= 0.421894
Thermal correction to Enthalpy= 0.422838
Thermal correction to Gibbs Free Energy= 0.342595
Sum of electronic and thermal Free Energies= -1490.512917

C	-1.73665	-1.42284	-0.79815
H	-2.02026	-2.31592	-1.35751
C	-2.03399	-0.18462	-1.66997
H	-1.50600	-0.23045	-2.62279
C	-1.81951	1.16784	-0.97206
H	-2.17005	1.96402	-1.62982
O	-3.45188	-0.25034	-1.96640
C	-4.23262	-0.19278	-0.78549
O	-4.03993	1.02702	-0.10640
O	-0.42127	1.35234	-0.69565
O	-0.36253	-1.53130	-0.38809
C	-2.57688	-1.30462	0.48471
H	-2.45395	-2.17964	1.12175
C	-2.65287	1.16562	0.32844
H	-2.58070	2.12160	0.84796
C	-2.28495	-0.00745	1.18939
C	-1.70008	0.12253	2.38505
H	-1.52282	1.10093	2.82982
O	-1.25986	-0.94950	3.09831
C	-0.89885	-0.66466	4.46076
H	-1.77883	-0.36918	5.04096
H	-0.48408	-1.58709	4.86780
H	-0.14473	0.12806	4.50326
C	0.49950	-2.13641	-1.23791
C	0.02467	2.63144	-0.61318
O	0.18982	-2.46136	-2.37509
O	-0.70550	3.59857	-0.77359
C	1.47498	2.71605	-0.31296
C	2.24973	1.57512	-0.05595

C	2.06984	3.98667	-0.28004
C	3.60661	1.70760	0.23105
H	1.79230	0.59400	-0.07542
C	3.42716	4.11295	0.00292
H	1.46231	4.86284	-0.47855
C	4.19665	2.97404	0.25938
H	4.20045	0.82171	0.43404
H	3.88530	5.09667	0.02515
H	5.25467	3.07426	0.48218
C	1.83691	-2.35429	-0.63252
C	2.08024	-2.13209	0.73268
C	2.87229	-2.80863	-1.46334
C	3.35346	-2.35713	1.25362
H	1.27769	-1.79599	1.37930
C	4.14230	-3.02877	-0.93719
H	2.67210	-2.98089	-2.51520
C	4.38441	-2.80197	0.42091
H	3.54033	-2.18989	2.30962
H	4.94252	-3.37604	-1.58300
H	5.37479	-2.97525	0.83090
O	-3.96852	-1.29789	0.04881
H	-5.27790	-0.24556	-1.08646

(±)-**22**

Zero-point correction= 0.433879 (Hartree/Particle)

Thermal correction to Energy= 0.457738

Thermal correction to Enthalpy= 0.458682

Thermal correction to Gibbs Free Energy= 0.379905

Sum of electronic and thermal Free Energies= -1342.313712

C	-1.82612	-1.25545	-0.99419
H	-2.16477	-2.09938	-1.60993
C	-2.09452	0.05126	-1.78275
H	-1.58675	0.05454	-2.74875
C	-1.78090	1.33829	-0.99745
H	-2.12249	2.19406	-1.59477
O	-3.51860	0.07102	-2.06806
C	-4.27948	0.09097	-0.87644
O	-4.01403	1.24937	-0.11862
O	-0.38410	1.41275	-0.77437
O	-0.48154	-1.44064	-0.58947
C	-2.67221	-1.18954	0.29102
H	-2.59054	-2.11304	0.86332
C	-2.61460	1.28511	0.30960
H	-2.50462	2.19205	0.90562
C	-2.30889	0.03644	1.08464
C	-1.71452	0.04640	2.28230

H	-1.47770	0.97692	2.79656
O	-1.34089	-1.10111	2.91803
C	-0.94926	-0.93058	4.28953
H	-1.79557	-0.59481	4.89780
H	-0.61405	-1.90802	4.63786
H	-0.12789	-0.21082	4.37187
C	0.40624	-1.87613	-1.61883
C	0.09536	2.72057	-0.44254
C	1.56483	2.63639	-0.10461
C	2.09732	1.50183	0.52231
C	2.40972	3.71766	-0.38407
C	3.45175	1.44905	0.85665
H	1.45168	0.65656	0.73530
C	3.76141	3.67096	-0.03668
H	2.00760	4.59837	-0.87828
C	4.28757	2.53435	0.58197
H	3.85288	0.55620	1.32804
H	4.40453	4.51730	-0.26003
H	5.34096	2.49296	0.84302
C	1.76266	-2.17402	-1.02151
C	1.92005	-2.47663	0.33694
C	2.89121	-2.17375	-1.85241
C	3.18551	-2.76890	0.85345
H	1.05534	-2.47058	0.99180
C	4.15281	-2.47557	-1.33876
H	2.77982	-1.93122	-2.90635
C	4.30492	-2.77198	0.01860
H	3.29410	-2.99713	1.90993
H	5.01771	-2.46993	-1.99578
H	5.28719	-3.00062	0.42140
O	-4.06714	-1.08127	-0.12263
H	-5.33063	0.11527	-1.16210
H	-0.06860	3.40635	-1.28527
H	-0.46221	3.12138	0.41576
H	0.50579	-1.11027	-2.39848
H	-0.00662	-2.77760	-2.09814

Second order perturbation theory analysis of the Fock matrices in the NBO analyses for the DFT calculated structures

(±)-**9** (*E*-siomer)

Threshold for printing: 0.50 kcal/mol

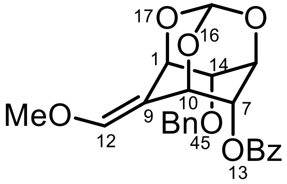

Donor NBO (i)	Acceptor NBO (j)	E(2) kcal/mol	E(j)-E(i) a.u.	F(i,j) a.u.
BD (1) C 1 - O 17	/***. BD*(1) O 14 - C 45	1.89	1.06	0.040
BD (2) C 9 - C 12	/***. BD*(1) C 1 - O 17	4.79	0.51	0.045
BD (2) C 9 - C 12	/***. BD*(1) C 10 - O 16	5.52	0.51	0.048
BD (1) C 10 - O 16	/***. BD*(1) C 7 - O 13	2.11	1.02	0.042

(±)-**10** (*Z*-siomer)

Threshold for printing: 0.50 kcal/mol

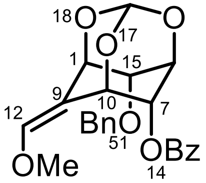

Donor NBO (i)	Acceptor NBO (j)	E(2) kcal/mol	E(j)-E(i) a.u.	F(i,j) a.u.
BD (1) C 1 - O 18	/***. BD*(1) O 15 - C 51	1.81	1.06	0.039
BD (2) C 9 - C 12	/***. BD*(1) C 1 - O 18	5.63	0.51	0.048
BD (2) C 9 - C 12	/***. BD*(1) C 10 - O 17	5.10	0.51	0.046
BD (1) C 10 - O 17	/***. BD*(1) C 7 - O 14	2.09	1.02	0.041

(±)-**8**

Threshold for printing: 0.50 kcal/mol

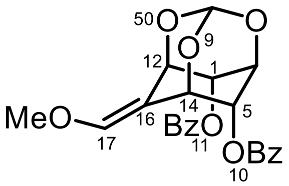

Donor NBO (i)	Acceptor NBO (j)	E(2) kcal/mol	E(j)-E(i) a.u.	F(i,j) a.u.
BD (1) O 9 - C 14	/***. BD*(1) C 5 - O 10	2.08	1.02	0.041
BD (1) C 12 - O 50	/***. BD*(1) C 1 - O 11	1.90	1.02	0.040
BD (2) C 16 - C 17	/***. BD*(1) O 9 - C 14	5.52	0.51	0.048
BD (2) C 16 - C 17	/***. BD*(1) C 12 - O 50	4.77	0.51	0.045

(±)-**22**

Threshold for printing: 0.50 kcal/mol

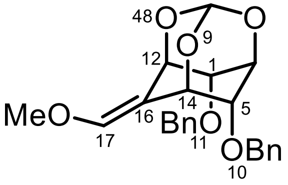

Donor NBO (i)	Acceptor NBO (j)	E(2) kcal/mol	E(j)-E(i) a.u.	F(i,j) a.u.
BD (1) O 9 - C 14	/988. BD*(1) C 5 - O 10	1.99	1.07	0.041
BD (1) C 12 - O 48	/982. BD*(1) C 1 - O 11	1.62	1.07	0.037
BD (2) C 16 - C 17	/994. BD*(1) O 9 - C 14	5.43	0.51	0.047
BD (2) C 16 - C 17	/999. BD*(1) C 12 - O 48	4.71	0.52	0.044
